# Programmable mesoporous carbon architectures from liquefied wood via reactive-emulsion-mediated self-assembly

**DOI:** 10.1038/s41467-026-77269-7

**Published:** 2026-08-29

**Authors:** Yang Li, Kun Zhang, Shenghui Jiao, Guangying Li, Yanhong Liu, Shirui Zou, Rui Teng, Hongjian Fu, Chunhui Ma, Sha Luo, Wei Li, Zaiwang Zhao, Shouxin Liu

**Affiliations:** 1 Key Laboratory of Utilization of Woody Oil Resource, Northeast Forestry University, Harbin, PR China; 2 Inner Mongolia Key Laboratory of Low Carbon Catalysis, College of Energy Materials and Chemistry, College of Chemistry and Chemical Engineering, Inner Mongolia University, Hohhot, PR China; 3 Key Laboratory of Bio-based Material Science and Technology of the Ministry of Education, Northeast Forestry University, Harbin, PR China

**Keywords:** Nanoparticles, Nanoparticles, Mechanical properties

## Abstract

Developing biomass-derived programmable self-assembly systems for precise control of carbon morphology and architecture remains challenging, primarily because of the intrinsic heterogeneity that hinders controllable assembly. Here, we report a biomass-enabled reactive-emulsion-mediated strategy for converting liquefied wood into various mesoporous carbon nanomaterials. This synthesis features the introduction of trioctyl phosphate (TOP) as a hydrophobic swelling agent for composite micelles and an interfacial modifier, shifting the preferred micelle curvature/packing tendency and biasing the assembly pathway from homogeneous aqueous self-assembly toward interface-associated anisotropic organization. This coupled regulation progressively reduces the density of ordered mesochannels within the fibrous framework and promotes the evolution from ordered mesoporous nanofibers to hollow nanofibers and bowl-like architectures. The fabricated hollow carbon nanofiber-based mixed ion-electron thermoelectric generator (MTEG) exhibits a high thermopower of 12.33 mV K^–1^ (across 40 kΩ). This strategy provides a versatile route for constructing biomass-derived mesoporous carbon materials with tunable morphologies and pore architectures.

## Abstract (web summary)

Developing biomass-derived self-assembly systems for controlling carbon morphology remains challenging because of their intrinsic heterogeneity, which hinders controllable assembly. Here, the authors report a biomass-enabled reactive-emulsion-mediated strategy for converting liquefied wood into various mesoporous carbon nanomaterials.

## Introduction

Mesostructure and morphology jointly govern transport pathways, active-site exposure, and interfacial accessibility, determining the performance of mesoporous carbons in adsorption, catalysis, and electrochemical applications^1–6^. Conventional soft-template strategies have enabled substantial progress in mesoporous carbon synthesis; however, topological engineering of mesoporous carbon materials remains constrained by the single-phase characteristic of most solution systems and the limited coordination of micelle assembly and precursor condensation^7–18^. The structural modulation is predominantly restricted to adjustments of the micelle curvature, swelling behavior, or nucleation kinetics^19–24^. These factors are often optimized separately, making it difficult to achieve coupled control over both morphology and mesostructure within a single synthetic system to fabricate multi-mode mesoporous carbon materials.

In emulsion-induced synthesis of mesoporous carbon materials, oil molecules can simultaneously swell the hydrophobic blocks of micelles and construct oil/water interfaces for the self-assembly and growth process of micelles^25–30^. The relative affinity among precursor species, surfactant micelles, oil phase, and solvent can be described by an effective interfacial-energy/interfacial-affinity framework, which is useful for rationalizing whether composite micelles preferentially remain in the bulk aqueous phase or become enriched at oil/water interfaces^31–36^. Although packing-parameter regulation and interfacial micelle assembly have been widely used in soft-template synthesis, most reported systems rely on model synthetic precursors, such as dopamine or other well-defined monomers^37–40^. In these systems, the carbon precursor generally serves mainly as a framework-forming component, whereas the simultaneous programming of external morphology and internal mesopore architecture from a renewable biomass-derived precursor remains rarely explored^41–46^. Therefore, establishing a reactive emulsion platform in which biomass can participate in micelle organization, interfacial localization, and polymerization-induced structural fixation would provide a distinct route beyond conventional homogeneous soft-template or droplet-directed assembly.

Standing out as an important biomass material, wood has garnered considerable interest according to its renewability and rich functional groups^47^. In particular, the conversion of biomass wood into liquid polyhydroxy small molecules (LW) has been demonstrated as an effective method to synthesize mesoporous carbon with diverse morphologies including carbon spheres, gyroids and flakes^48–50^. However, owing to the compositional complexity and rapid polymerization rate, it is difficult to coordinate interfacial participation, template interaction, and polymerization within a controllable soft-template assembly process. The self-assembly involved occurs exclusively confined in a single aqueous-phase environment through homogeneous self-assembly, and strategies for regulating structure remain limited to micelle structure and nucleation kinetics. The simultaneous regulation of multiple mesoporous carbons derived from biomass, particularly spanning hollow carbon fibers, ordered mesoporous fibers, and even less to diverse mesoporous nanospheres within a single synthetic system remains challenging.

Herein, we develop a biomass-enabled reactive-emulsion-mediated self-assembly platform to synthesize mesoporous carbon architectures from liquefied wood. By systematically varying the TOP and ethanol content, we gain coupled control over micelle curvature and interfacial interactions, biasing the self-assembly pathway from homogeneous toward interface-associated anisotropic self-assembly of composite micelles and enabling the evolution of carbon nanofibers from parallel-ordered channels to hollow architectures. Moreover, beyond nanofibers, the same synthetic framework affords concurrent access to diverse mesoporous nanospheres with tailored architectures. Building on the mechanistic insights, we further demonstrate the utility of the resulting hollow carbon nanofibers in MTEGs, exhibiting continuous and reliable power output under steady temperature gradients. This study provides insights for designing biomass-derived mesoporous carbon and establishes a platform that enables the multifunctional utilization of programmable carbon architectures.

## Results

### Formation process

**Fig. 1:**
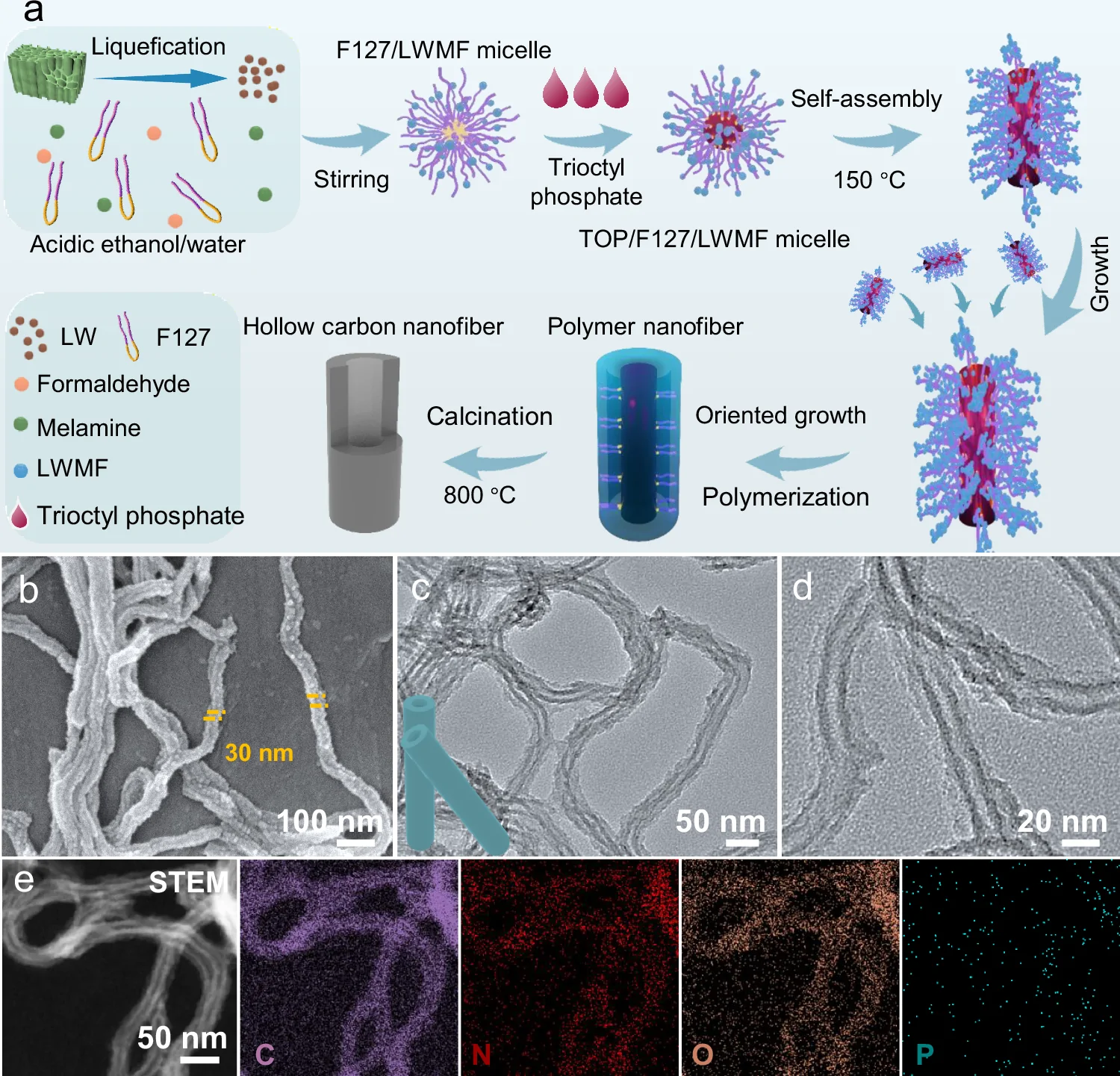
Microstructure characterizations of the hollow carbon nanofibers. **a** Schematic representation of the formation process of the hollow carbon nanofiber. First, the micelle system was formed by stirring at 500 rpm. Then, the composite micelles were proposed to organize at the TOP/water interfacial regions and further assembled into the mesostructured polymer nanofibers. Last, carbonization of the dried nanofibers in N_2_ atmosphere resulted in hollow carbon nanofibers. **b** SEM image. **c**, **d** TEM images with different magnifications. **e** STEM image and EDX element mapping images of the MCNs with hollow structures by the micelle-oriented self-assembly.

Mesoporous carbon nanoparticles (MCNs) with fiber-like morphology and hollow mesostructure were prepared via a reactive-emulsion-mediated micelle self-assembly strategy, in which Pluronic F127 serves as the soft template, trioctyl phosphate (TOP) functions as the swelling agent and interface mediation agent, LW acts as the carbon source and interface regulator, followed by calcination under N_2_ atmosphere (Supplementary Fig. 1a–f). Specifically, hydrophobic TOP is introduced into an aqueous solution containing LW, formaldehyde, and melamine molecules to form a well-defined biphasic mixture. After continuous stirring, the mixture solution turns into a pale-yellow emulsion, accompanied by milky droplets adhering to the container walls, indicative of a stable emulsion in which nanoscale micelles and micron-sized TOP droplets coexist. Optical microscopy image reveals that the TOP droplets are uniform, with an average diameter of ~4.5 μm (Supplementary Fig. 1g). LW and melamine react with formaldehyde, respectively, and further co-polymerize to form LW-melamine-formaldehyde (LWMF) resins^20^. Driven by hydrophobic association and hydrogen bonding, Pluronic F127 coassembles with TOP molecules and LWMF polymers to form TOP/F127/LWMF composite micelles. As the fundamental building blocks for subsequent growth, the formed composite micelles initially assemble into micelle aggregates via synergistic TOP-mediated hydrophobic interactions and intermolecular chemical bonding among carbon source moieties during the hydrothermal process. Afterward, these aggregates serve as the nucleation seeds, promoting the oriented overgrowth of additional composite micelles onto the oil droplet surface via interfacial energy-driven adsorption^49^. As the reaction proceeds, composite micelles migrate toward the interface of oil/water and preferentially assemble at the termini of the growing nanoparticles, undergoing radial overgrowth self-assembly mode alongside the TOP to generate fibrous polymer intermediates. After hydrothermal treatment, the resulting polymer products exhibit well-defined, self-supporting columnar architectures with pronounced surface striations, supporting the presence of directional interfacial interactions and strong hydrogen-bonding between LWMF and the templates (Supplementary Fig. 1d). Notably, directional interactions between composite micelles and TOP droplets promote progressive interfacial adsorption and radial anisotropic overgrowth. After carbonization and removal of the TOP/F127 templates, hollow carbon nanoparticles with uniform fiber-like morphology could be obtained. (Fig. 1a). Time-dependent TEM observations were further performed to track the formation of hollow nanofibers (Supplementary Fig. 2). Early products obtained at 30 min and 60 min show aggregated or partially elongated intermediates, whereas fibrous frameworks with hollow/mesoporous features become evident after 180 min. Prolonging the hydrothermal treatment to 10 h yields well-defined hollow nanofibers, while further extension to 24 h does not induce obvious morphological transformation or coarsening. The corresponding time-dependent SAXS patterns show the gradual development of a broad mesoscale scattering feature during hydrothermal treatment. The broad feature around *q* ≈ 0.7 nm^–1^ corresponds to a characteristic length of approximately 9 nm. No new sharp higher-order peaks or additional peak series appear upon prolonged hydrothermal treatment, indicating that extended polymerization does not induce an independent late-stage ordered mesophase transition. These observations suggest that the hollow fibrous architecture is established through polymerization-coupled micelle/emulsion assembly and subsequently fixed by precursor condensation, rather than being generated by a separate phase transition or by carbonization alone.

### Morphological and structural characterization

**Fig. 2:**
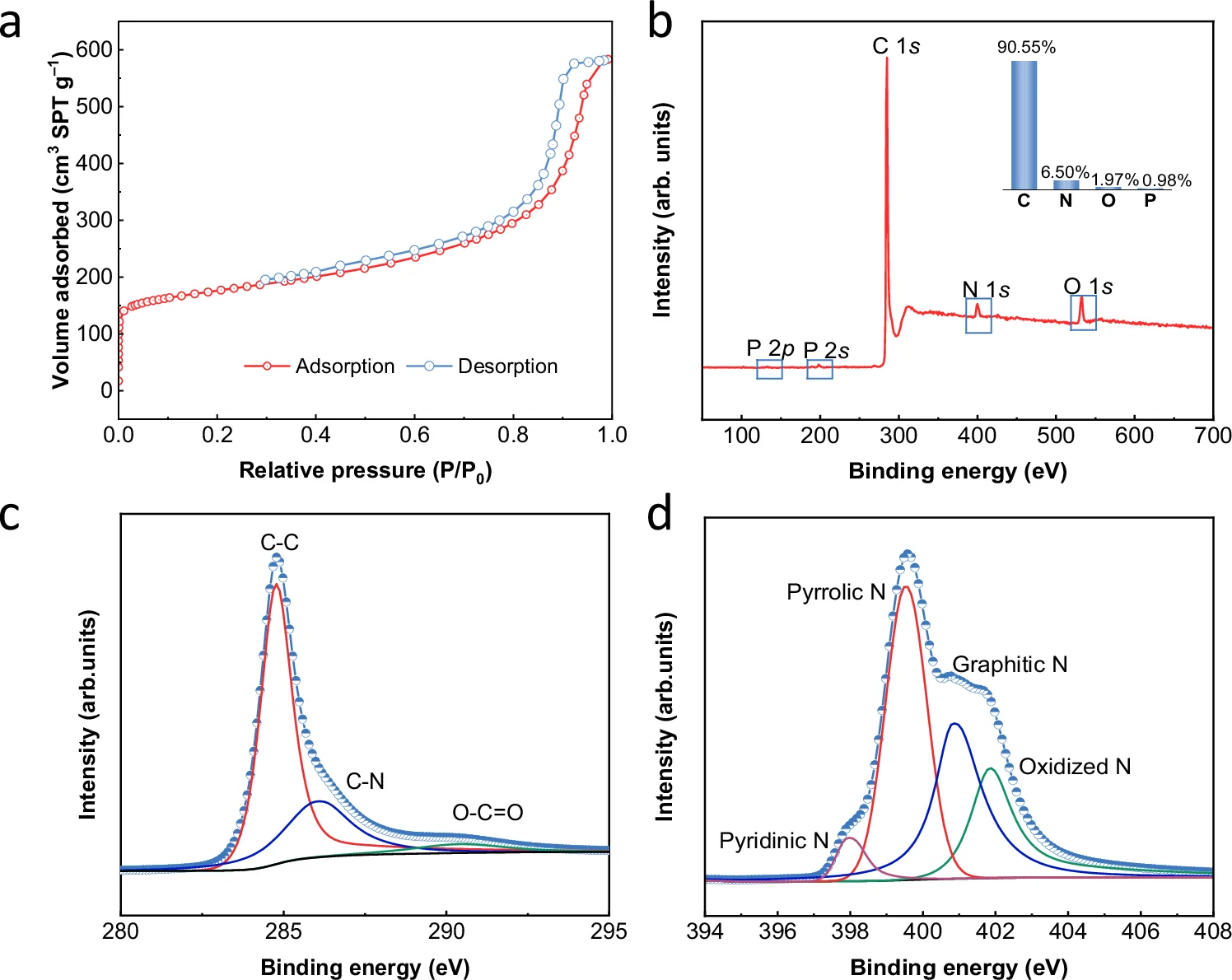
Physicochemical characterizations of the hollow carbon nanofibers. **a** N_2_ isotherms, **b** XPS survey spectrum, and **c**, **d** high-resolution C 1*s* and N 1*s* XPS spectrum of the hollow carbon nanofibers prepared by the emulsion-mediated self-assembly strategy, respectively. Inset **b** is the corresponding element percentages.

Scanning electron microscopy (SEM) images reveal that the as-prepared mesostructured resin polymers exhibit a uniform fibrous morphology, with a representative cross-sectional diameter of 40 nm and striped grooves along the nanofiber surface, which are consistent with the radial growth of composite TOP/F127/LWMF micelles during interfacial self-assembly (Supplementary Fig. 3a, c). Transmission electron microscopy (TEM) images show that the polymeric nanofibers possess well-defined hollow mesochannels of ~12 nm (Supplementary Fig. 3b, d). After carbonization in N_2_ atmosphere, the fibrous morphology is retained while shrinking to a representative outer diameter of 30 nm (Fig. 1b). The hollow interior and porous wall are preserved after carbonization, with a wall thickness of ~8 nm as observed from the magnified TEM image (Fig. 1c, d). Thermogravimetric analysis indicates that the yield of hollow carbon nanofibers is calculated to be ~39% (Supplementary Fig. 4). The element mapping images further reveal a homogenous distribution of C, N, O, and P elements throughout the hollow carbon nanofibers (Fig. 1e). Nitrogen sorption isotherms of hollow carbon nanofibers display a representative type IV curve with a pronounced hysteresis loop and a rapid increase in the adsorption branch at a relative pressure of 0.4–0.9, indicating the copresence of micropore and large mesoporous structure. Meanwhile, the specific surface area and total pore volume can be calculated to be 639 m^2^ g^–1^ and 0.90 cm^3^ g^–1^, respectively (Fig. 2a). The Raman spectrum shows two typical peaks located at 1320 and 1593 cm^–1^, which are assigned to the D-band (disordered *sp*^3^ carbon) and G-band (graphitic *sp*^2^ carbon structures), respectively. The relatively high *I*_D_/*I*_G_ ratio suggests the existence of abundant defects and amorphous structure domains within the carbon framework (Supplementary Fig. 5). This can also be corroborated by the X-ray diffraction (XRD) pattern, in which two diffraction peaks at ~21.3° and 43.8° are assigned to (002) and (100) planes, respectively (Supplementary Fig. 6). The X-ray photoelectron spectroscopy (XPS) displays five peaks located at 284.9, 400.7, 533.3, 132.1, and 198.3 eV, corresponding to C 1*s*, N 1*s*, O 1*s*, P 2*p* and P 2*s*, respectively (Fig. 2b). The N content in hollow carbon nanofibers framework is measured to be ~6.5%. The high-resolution C 1*s* XPS spectrum of the hollow carbon nanofibers can be deconvoluted into distinct components corresponding to C-C (284.7 eV), C-N (286.1 eV) and O-C=O (290.4 eV) (Fig. 2c). Meanwhile, the N 1*s* spectrum can be resolved into four peaks, including the pyridinic N (397.9 eV), pyrrolic N (399.6 eV), graphitic N (400.9 eV) and oxidized N (401.9 eV), proving the formation of uniformly doped hollow N-containing carbon architectures (Fig. 2d).

### Regulation of mesophase structures

**Fig. 3:**
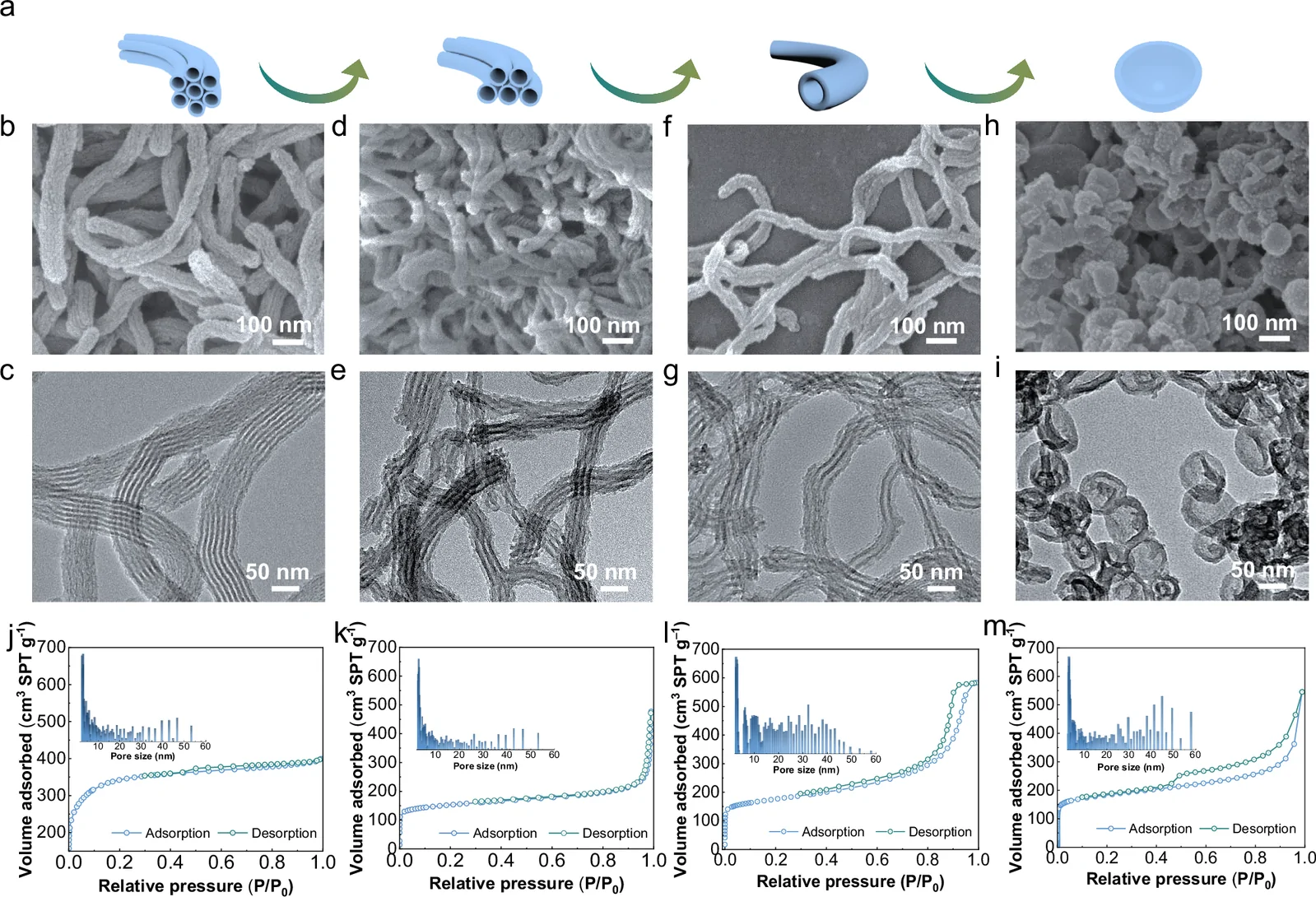
Controllability of the emulsion-mediated micelle self-assembly strategy by tuning the TOP content. **a** Structural models depicting experimentally sampled TOP-dependent morphology/mesostructured evolution of mesoporous carbon nanomaterials from ordered mesochannel nanofibers to hollow nanofibers and ultimately to bowl-like nanospheres, **b**, **d**, **f**, **h** SEM and **c**, **e**, **g**, **i** TEM images of the MCNs-x (*x* = 1, 2, 3, 4) prepared by adjusting the amount of TOP. **b**, **c** 0.05 mL, **d**, **e** 0.1 mL, **f**, **g** 0.2 mL, **h**, **i** 0.3 mL. **j**–**m** The corresponding N_2_ sorption isotherms and distribution histograms of pore sizes.

To further elucidate the formation mechanism of the intriguing hollow fibrous architectures and the feasibility of emulsion-induced self-assembly processes, a series of TOP-dependent control experiments was systematically conducted. The detailed parameter matrix and morphology/structure map are summarized in Supplementary Table 1. In the absence of TOP, three-dimensional carbon nanoparticles with dodecahedral morphology and ordered mesopores were obtained, indicating that the assembly mainly proceeds through a homogeneous aqueous pathway without an oil phase (Supplementary Fig. 7a, b). After TOP was introduced, the system evolved from a single aqueous assembly environment into a reactive emulsion containing oil/water interfaces. Upon gradually increasing the TOP amount from 0.05 to 0.3 mL, the macroscopic product changed from soft and collapsible columns to a self-supporting monolith, and finally to compact blocks deposited at the bottom (Supplementary Fig. 8). Correspondingly, the resulting mesoporous carbon nanomaterials (MCNs) showed a systematic TOP-dependent evolution: the external morphology evolved from one-dimensional nanofibers to bowl-like nanospheres, while the internal architecture changed from ordered mesochannels to hollow structures with a progressive decrease in ordered channel density (Fig. 3a and Supplementary Fig. 9). At 0.05 mL TOP, uniform carbon nanofibers with a diameter of ~80 nm were generated with multiple linear protrusions observed on their surface (Fig. 3b). TEM images further reveal multiple ordered mesoporous channels (~3 nm) packed in a hexagonal arrangement and aligned parallel to the one-dimensional fiber axis, suggesting that homogeneous micelle organization remains dominant under limited TOP swelling (Fig. 3c). As the TOP volume increased to 0.1 mL, the fibrous morphology was retained, but showed a reduced diameter of ~50 nm, accompanied by similar mesopore size and substantial decrease in ordered mesochannels density (Fig. 3d, e). Further increasing the TOP amount to 0.2 mL, the fibrous morphology was well-preserved, while the ordered mesoporous channels underwent a distinct evolution into hollow structures (Fig. 3f, g). When using 0.3 mL TOP in the reaction system, bowl-like nanospheres were produced, suggesting that excessive TOP shifts the balance toward lower-curvature or lamellar-like assemblies (Fig. 3h, i). When the TOP amount reached 0.4 mL, irregular lamellar aggregates were obtained (Supplementary Fig. 10). The specific surface areas of the carbon materials obtained at increasing TOP contents were calculated to be 1235, 566, 639, and 687 m^2^ g^–1^, while corresponding total pore volumes were measured to be 0.62, 0.72, 0.90, and 0.84 cm^3^ g^–1^, respectively (Fig. 3j–m). The NLDFT-derived pore-size distributions reveal hierarchical porosity in these MCNs, including micropores, primary micelle-templated mesopores, and broader larger-pore contributions, which might originate from hollow interiors, interparticle voids, and structural cavities. These results indicate that TOP acts not only as a hydrophobic swelling agent, but also as an interfacial modifier that changes the relative contribution of homogeneous aqueous assembly and interface-associated anisotropic organization. Low TOP content favors ordered mesochannel formation, intermediate TOP content promotes hollow fibrous architectures, and excessive TOP shifts the system toward low-curvature or lamellar-like assemblies that no longer preserve ordered fibrous frameworks.

The solvent also plays a crucial role in determining both interfacial tension, micelle structures, and polymerization kinetics, which in turn influence the micelles self-assembly behavior. To clarify the unique role of the solvent in this system, a series of control experiments was first carried out by replacing ethanol with other organic solvents. Notably, regardless of whether methanol, isopropanol or N,N-dimethylformamide was employed, only irregular aggregates or non-porous spheres were obtained, illustrating the indispensable role of ethanol for establishing well-defined mesostructures (Supplementary Fig. 11). Building on the as-prepared hollow nanofibers, a series of MCNs were prepared with different contents of ethanol in the reaction system (Supplementary Fig. 12). When the ethanol volume was reduced to 5 mL, the resulting MCNs-14 evolved into irregular gyroids with ordered mesoporous channels (Supplementary Figs. 12a and 13a, b). Interestingly, increasing the ethanol volume to 15 mL led to the formation of MCNs-15 with a distinctive braided-like morphology composed of interlacing mesoporous channels, as further confirmed by an enlarged SEM image (Supplementary Figs. 13e, f, and 14). Further increasing the ethanol volume to 20 mL preserved fibrous morphology, while ordered mesoporous channels became more distinct (Supplementary Fig. 13g, h). Once the ethanol volume exceeded 25 mL, the products gradually transformed into mesoporous carbon spheres with increasing particle size and ultimately into solid spheres when the ethanol volume was 40 mL (Supplementary Fig. 13i–n).

**Fig. 4:**
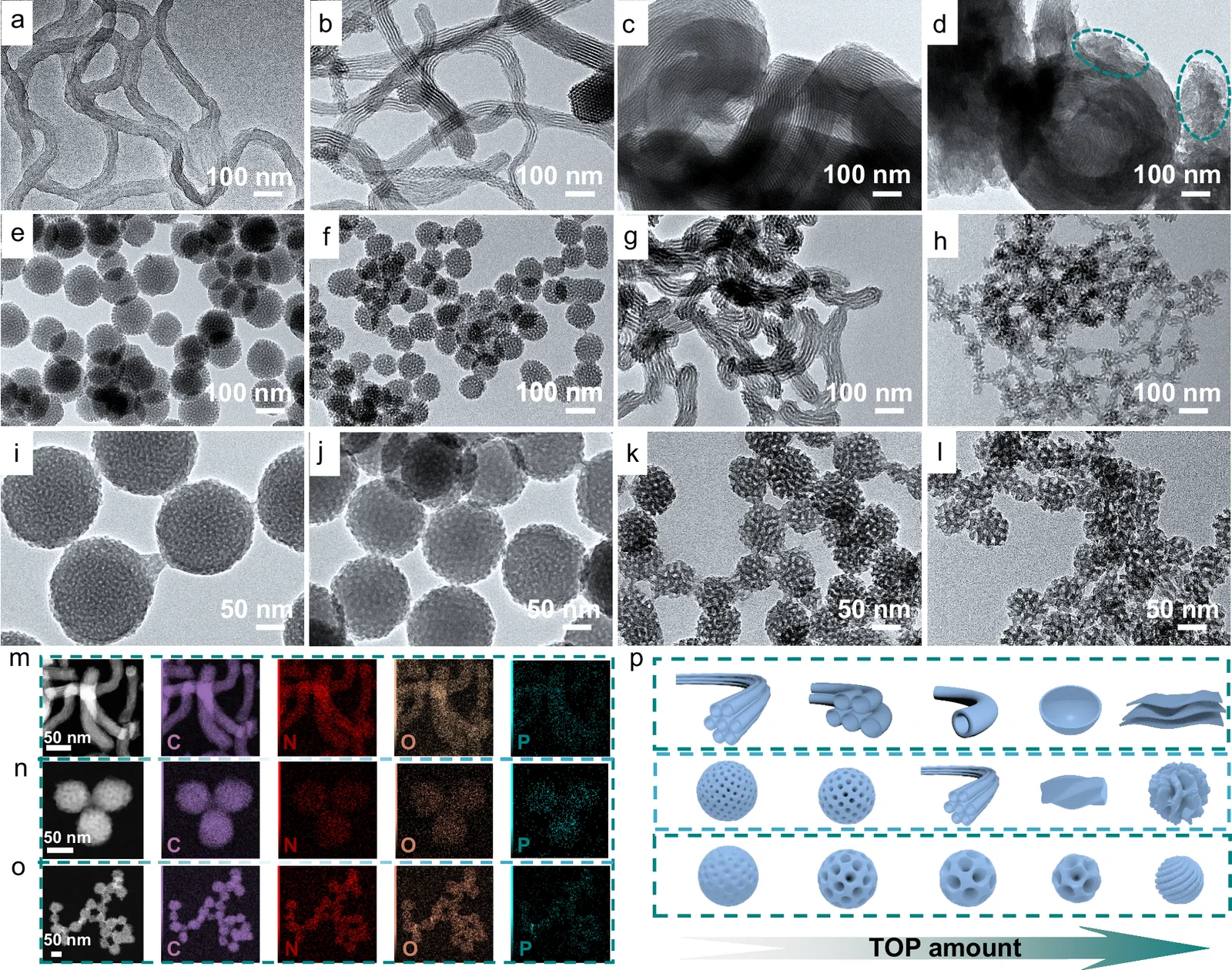
The versatility of the synthetic method. **a**, **e**, **i** TEM images of the MCNs-x (*x* = 8, 9, 10) prepared with 0.05 mL TOP in the reaction system of 5, 20, and 25 mL ethanol. **b**, **f**, **j** TEM images of the MCNs-x (*x* = 11, 12, 13) prepared with 0.1 mL TOP in the reaction system of 5, 20, and 25 mL ethanol. **c**, **g**, **k** TEM images of the MCNs-x (*x* = 14, 16, 17) prepared with 0.2 mL TOP in the reaction system of 5, 20, and 25 mL ethanol. **d**, **h**, **l** TEM images of MCNs-x (*x* = 20, 21, 22) prepared with 0.3 mL TOP in the reaction system of 5, 20, and 25 mL ethanol. **m**–**o** EDX element mappings of the ordered carbon nanorods (MCNs-16), multichambered nanospheres (MCNs-17), and branched nanoclusters (MCNs-22). **p** Structural models of the prepared MCNs.

Based on this, a series of MCNs-x (*x* = 8–24) was synthesized under varied ethanol and TOP contents to systematically investigate the synergistic effect mechanism of ethanol and TOP on product morphology and structure (Fig. 4). In the absence of ethanol, modulation of TOP amount failed to produce well-defined morphologies. Instead, only ill-defined aggregates and solid spheres were obtained, although mesostructural features became gradually discernible with increasing TOP content (Supplementary Fig. 15). This observation suggests that while TOP can partially dissolve the LW and contribute to mesostructure formation, it is insufficient to direct ordered self-assembly without appropriate solvent mediation. At low ethanol content (5 mL), increasing TOP content promoted the formation of highly ordered mesostructures, whereas excessive TOP led to defective structures, which is probably because excessive TOP disrupts the kinetic matching between micelle organization and precursor condensation under low-ethanol conditions (Fig. 4a–d). When the TOP content was fixed at 0.05 mL, increasing the ethanol volume from 10 to 25 mL induced a composition-dependent evolution of products from ordered nanofibers to golf ball-like mesoporous nanospheres (Fig. 4e, i, and Supplementary Fig. 16a). A similar transition was observed at a TOP volume of 0.1 mL, where raspberry-like nanospheres were obtained with increasing ethanol content (Fig. 4f, j, and Supplementary Fig. 16b). Under a TOP volume of 0.2 mL, increasing ethanol content drove a transition from one-dimensional fibers to nanospheres, accompanied by a change in mesopore geometry from cylindrical to spherical pores (Fig. 4g, k, and Supplementary Fig. 16c). Likewise, elevating the ethanol content drove the morphological evolution from bowl-shaped to unique twisted nanoribbons and branched nanoclusters under a TOP volume of 0.3 mL (Fig. 4h, l, and Supplementary Fig. 16d). Element mapping images of the as-prepared ordered nanorods, multichambered nanospheres and the branched nanospheres confirmed the homogenous distribution of C, N, O, and P elements across the skeleton (Fig. 4m–o) and additional SEM images of the MCNs prepared in reaction system containing 20 mL are provided in Supplementary Fig. 17. Further adjusting the TOP volume to 0.4 mL, unique dendritic nanospheres and gear-like nanospheres were generated at ethanol volumes of 20 and 25 mL, respectively (Supplementary Figs. 18 and 19). The evolution of mesostructures reveals that increasing ethanol content improves the solvation of PEO segments and hydroxyl-rich LWMF fragments, thereby favoring more dispersed or isotropic precursor organization. At the same time, ethanol increases the effective polar/interfacial contribution of the composite micelles and shifts the preferred micelle curvature toward higher-curvature organizations. Importantly, this curvature regulation operates within the assembly pathway selected by the interfacial-energy balance, rather than independently determining the assembly locus. In addition to structural regulation, ethanol also plays a critical role in controlling polymerization kinetics. Time-dependent experiments terminated at different hydrothermal durations (20, 40, 60, and 90 min) revealed distinct growth behaviors for hollow nanofibers and multichambered nanospheres (Supplementary Fig. 20). Reaction systems containing lower ethanol content exhibited earlier polymer formation within the same reaction time, indicating that ethanol functions as a proton scavenger to moderate polymerization rates, thereby favoring the development of ordered mesostructures.

At an ethanol volume of 25 mL, a series of carbon nanospheres with diverse morphologies and mesostructures was obtained simply by tuning the TOP content (Fig. 4i–l, Supplementary Figs. 18 and 21). With increasing TOP, the pore size of the as-prepared nanospheres increased, consistent with the role of TOP as an interfacial modifier and swelling agent for hydrophobic domains. Meanwhile, the particle size of nanospheres decreases with increasing TOP volume, reflecting the spontaneous minimization of interfacial energy under enhanced oil–water interactions. Moreover, the regular spherical mesopores exhibited a tendency to transform into worm-like mesopores upon increasing TOP, a phenomenon consistently observed at an ethanol volume of 10 and 20 mL, corresponding to transitions from cylindrical to lamellar mesopores and from typical spherical to cylindrical mesopores, respectively (Fig. 4f, g). All these results demonstrate that the diverse morphologies ranging from nanofibers to nanospheres, together with tunable pore structures, can be achieved within a single reaction system through the synergistic regulation of ethanol and TOP (Fig. 4p). These results define an experimentally sampled EtOH–TOP composition window in which ethanol mainly shifts the system from anisotropic fibrous/hollow architectures toward isotropic or quasi-isotropic nanospheres, while TOP further regulates the internal pore architecture and surface mesostructure within each morphology regime.

Furthermore, the effects of hydrothermal temperature, Pluronic F127 amount, and pH value were also investigated to gain deeper insight into the formation mechanisms of hollow nanofibers (MCNs-3) and multichambered nanospheres (MCNs-17). Optical photograph of the MCNs-3 precursor solution after 40 min hydrothermal treatment shows more pronounced solid formation at 180 °C, suggesting accelerated polymerization at elevated temperatures (Supplementary Fig. 22). For the hollow nanofiber system, increasing hydrothermal temperature from 130 to 150 °C induced a pronounced structure transition from aggregated nanostructures or layer-like structures to uniform fibrous and bowl-shaped architectures, respectively (Supplementary Fig. 23a–f). Further increasing the temperature to 180 °C led to irregular nanoparticles, suggesting that mismatch between the micelle self-assembly process and the polymerization kinetics of the carbon source under excessively high hydrothermal temperature, leading to disruption of the ordered assembly pathway. (Supplementary Fig. 23g–i). A similar temperature-dependent effect was observed for nanospheres, as illustrated in Supplementary Fig. 24. Aggregated nanospheres with poorly defined mesostructures were obtained at relatively low hydrothermal temperatures. Whereas elevated temperature promoted well-defined gear-like mesoporous carbon spheres. This unique structure closely resembles that of MCNs-24, which was prepared by increasing the TOP volume from 0.3 to 0.4 mL under otherwise identical conditions (Supplementary Fig. 18), suggesting that elevated temperature and increased TOP content can lead to similar structural outcomes through distinct but convergent pathways. Notably, the evolution in pore structure suggests that hydrothermal temperature affects the packing geometry of composite micelles. At elevated temperatures, the hydration of PEO segments is reduced, while the effective hydrophobic volume of the PPO/TOP domain increases. This change favors lower-curvature micellar configurations, thereby influencing the resulting mesopore geometry. These temperature-dependent results indicate that hydrothermal temperature affects both the kinetic matching between LWMF polymerization and soft-template assembly. Insufficient temperature slows precursor condensation and weakens framework fixation, whereas excessive temperature accelerates polymerization or disrupts micelle organization.

In addition, the F127/LW mass ratio was also found to exert a pronounced influence on both the morphology and pore structure of products. Only nonporous carbon spheres were obtained without adding Pluronic F127, indicating that Pluronic F127 is indispensable for the formation of mesostructures (Supplementary Fig. 25). The effect of Pluronic F127 was first examined during the fabrication of hollow nanofibers. When the F127 dosage was 0.5 g, the structure of the products gradually transformed from disordered to gyroids with highly ordered mesoporous channels and ultimately to concentric spirals (Supplementary Figs. 26a–d, 27, and 28) with increasing TOP volumes from 0.05 to 0.3 mL. The curved rods observed on the gyroid surface originate from the spiral packing of the cylindrical micelles during homogeneous assembly (Supplementary Fig. 27). At a dosage of 0.8 g, ordered mesoporous structures appeared at TOP of 0.05 mL (Supplementary Fig. 26e). Upon carefully increasing TOP volumes to 0.3 mL, well-defined nanofibers featuring ordered hexagonal mesoporous packed channels were obtained, followed by a gradual decrease in channel number, and the products eventually evolved into nanoflowers composed of nanosheets with ordered mesoporous channels on the edge (Supplementary Figs. 26f–h and 29). At an F127 dosage of 1.5 g, the morphology of products could be varied from ordered nanofibers to hollow nanofibers and to small nanobowls (Supplementary Figs. 26i–l and 30). Interestingly, increasing F127 dosage rendered the nanofibers more tortuous and cross-linked, indicative of enhanced micelle population density and stronger steric interactions during assembly. Subsequently, the effect of Pluronic F127 dosage on the mesoporous nanospheres was investigated. At a F127 dosage of 0.5 g, increasing TOP led to a transition from highly ordered mesoporous nanofibers to nanorods, accompanied by the appearance of small nanoparticles surrounding the nanorods (Supplementary Figs. 31a–d and 32). Increasing Pluronic F127 dosage to 0.8 g, uniform mesoporous nanospheres were initially formed and gradually evolved into worm-like and honeycomb-like particles upon further increasing TOP (Supplementary Figs. 31e–h and 33). At a Pluronic F127 dosage of 1.5 g, diverse mesoporous carbon nanospheres (pinecone-like and interconnected nanospheres) were obtained with increasing TOP, and the particle size decreased progressively (Supplementary Figs. 34 and 35). In general, increasing the F127 dosage at fixed TOP content promotes a mesostructural shift from cylindrical to spherical. Mechanistically, a higher F127 concentration increases the micelle population density and enhances steric repulsion among micelles, which increases the effective polar/interfacial contribution and favors higher-curvature assemblies. In contrast, increasing TOP content swells the PPO-rich hydrophobic domains and increases the effective hydrophobic contribution, driving the mesostructure toward lower-curvature cylindrical, hollow, or lamellar-like configurations. (Supplementary Fig. 36). These results confirm that F127 defines the soft-template assembly window. A sufficient amount of F127 is required to generate composite micelles and preserve ordered mesopores, while the final morphology is further selected by TOP-mediated interfacial affinity and ethanol-regulated solvation/curvature effects.

**Fig. 5:**
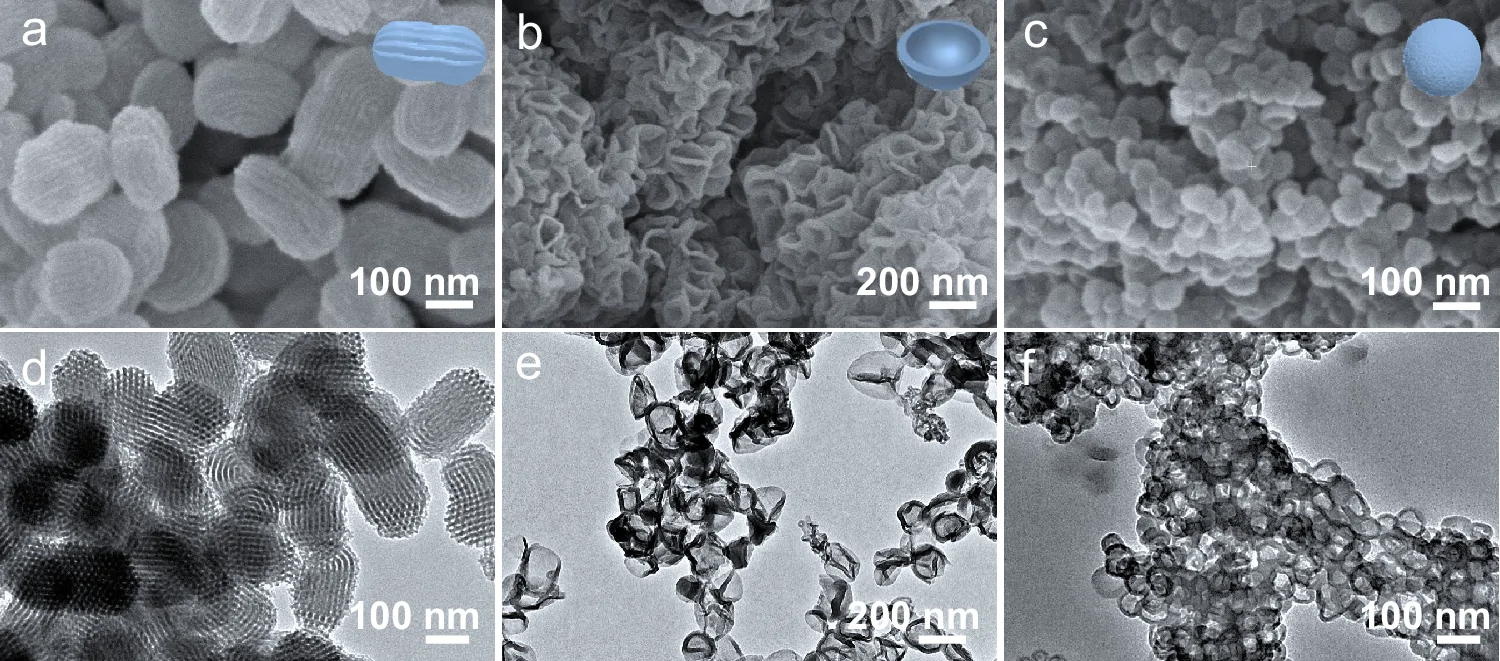
The effects of the copolymer templates for synthesizing MCNs by emulsion-mediated self-assembly strategy. **a**–**c** SEM and **d**–**f** TEM images of the MCNs-x (*x* = 5, 6, 7) synthesized with various amphiphilic triblock copolymers as templates: **a**, **d** worms, F108 + F127; **b**, **e** vesicle-like, P123 + F127; **c**, **f** hollow spheres, P84 + F127. The structural models are provided on the top. The results indicate that changing the copolymer template from F108/F127 to P123/F127 and P84/F127 leads to worm-like particles, vesicle-like nanospheres, and hollow carbon spheres, respectively.

The effect of the triblock copolymer template on morphology and structure was investigated to elucidate the generality of this approach. When Pluronic F108 was employed as the co-template, worm-like carbon nanoparticles with rough surfaces were obtained (Fig. 5a). The TEM image reveals the highly ordered mesoporous structure, indicating that the incorporation of F108 alters the micelle packing behavior while preserving mesoscopic ordering. (Fig. 5d). Upon partial replacement of F127 with P123, uniform vesicle-like nanospheres with well-defined internal cavities were generated (Fig. 5b, e). Further replacing Pluronic F127 with P84, well-defined hollow carbon spheres could be obtained (Fig. 5c, f). These results suggest that, while the self-assembly locus is primarily governed by the interfacial energy at the oil-water interface, the molecular architecture and hydrophobic block length of the triblock copolymer templates modulate the interfacial affinity and curvature of the composite TOP/template/LWMF micelles. Such regulation changes the preferred micelle curvature and induces distinct self-assembly pathways, including homogeneous assembly, vesicle formation, and interfacial overgrowth.

Meanwhile, the importance of HCl concentration on the morphology and mesostructure of hollow nanofibers (MCNs-3) and multichambered nanospheres (MCNs-17) was also investigated, as HCl directly regulates the polymerization kinetics of the carbon source within the self-assembly system. For the hollow nanofiber system (10 mL ethanol, 0.2 mL TOP), a relatively low acid concentration (1.37 M), hollow helical structures were obtained with parallel arrays of ordered mesopores, suggesting that insufficient polymerization kinetics and weakened interaction prevented the formation of continuous straight frameworks (Supplementary Fig. 37a). Increasing HCl concentration to 1.54 M led to the emergence of elongated nanofibers with long-range ordered mesoporous channels (Supplementary Fig. 37b). When the HCl concentration reached 1.71 M, well-defined hollow nanofibers with intact mesostructures were obtained (Supplementary Fig. 37c). Further increasing the HCl concentration to 1.89 M, the fibers became progressively smaller, more tortuous, and partially agglomerated (Supplementary Fig. 37d), implying that excessively accelerated polymerization began to mismatch with the self-assembly. At an even higher acid concentration of 2.06 M, rapid nucleation generated numerous small seed nuclei that failed to incorporate into the continuous nanofiber framework, resulting in shortened hollow nanorods with poorly defined mesostructure (Supplementary Fig. 37e). A similar acid-dependent kinetic effect was observed for the multichambered nanosphere system (25 mL ethanol, 0.2 mL TOP), as shown in Supplementary Fig. 38. With increasing HCl concentration from 1.37 M to 2.06 M, the nanospheres gradually decreased in size and ultimately evolved into agglomerated small spheres with discernible mesostructure. These results confirm that HCl concentration functions as an effective kinetic regulator that governs the channel density and structural integrity of products. Appropriate acid concentration ensures coordinated micelle self-assembly and precursor polymerization, whereas low or high HCl concentration leads to disordered structures or aggregated products, respectively.

To clarify whether the regulatory function originates from the oil phase itself rather than the specific chemistry of TOP, TOP was systematically replaced by trialkyl phosphate oils with different alkyl-chain lengths and hydrophobicity, including triethyl phosphate (TEP), tributyl phosphate (TBP) and triamyl phosphate (TAP), while keeping the remaining synthetic parameters unchanged. For the synthesis of nanofibers, when TEP was employed as the oil phase, the resulting product exhibited rhombic dodecahedral morphology with ordered spherical mesopores, suggesting that the composite micelles preferentially undergo homogeneous self-assembly in the aqueous phase when interface energy is relatively weak. (Supplementary Fig. 39a). Employing TBP with higher hydrophobicity led to the formation of ordered mesoporous nanofibers with tightly packed mesoporous channels aligned along the fiber (Supplementary Fig. 39b). Notably, when TAP with longer alkyl chains was used, the mesoporous channels became more clearly resolved while their number decreased, and the products displayed an apparent tendency toward hollowing, consistent with a shift toward interface-mediated self-assembly and overgrowth (Supplementary Fig. 39c). A similar chain-length-dependent trend was observed for the nanosphere-forming regime. With increasing alkyl-chain length of the oil phase, the particle size of the mesoporous carbon spheres gradually decreased, whereas the mesoporous structure became increasingly pronounced, and a progressive transition toward multi-chambered mesoporous nanocarbon spheres was manifested. The use of different trialkyl phosphates led to distinct mesostructural evolution, which may be attributed to the fact that longer alkyl-chain phosphate oils strengthen PPO/oil association and improve compatibility with the hydrophobic/aromatic fragments of LWMF, thereby increasing the tendency of composite micelles to organize at oil/water interfaces. At the same time, stronger hydrophobic swelling shifts the preferred micelle curvature toward lower-curvature configurations, promoting hollow or multi-chambered architectures. In contrast, shorter-chain oils provide weaker hydrophobic/interfacial interactions and retain a more homogeneous assembly pathway. These oil-phase controls demonstrate that the transition is not caused solely by the chemical identity of TOP, but by the combined hydrophobic swelling ability and interfacial affinity of the oil phase. Longer alkyl-chain phosphate oils strengthen PPO/oil association and interfacial coupling, thereby favoring anisotropic or hollow architectures, whereas shorter-chain oils provide weaker hydrophobic/interfacial interactions and retain a more homogeneous assembly pathway.

The generality of this emulsion-mediated synthetic approach was further examined, in which coconut shell and corn straw were selected as the raw biomass resources and converted into liquefied biomass precursors (Supplementary Fig. 40). Despite the different origins and compositions from wood, regular morphology and mesoporous structures were obtained. Moreover, varying TOP and ethanol in these biomass-derived systems produced structural evolution trends similar to those observed for liquefied wood, supporting the generality of their regulatory roles in emulsion-mediated self-assembly.

### Thermoelectric performance

Ionic thermoelectric systems are often limited by slow ion relaxation and concentration polarization, which tend to produce capacitive responses rather than sustained generator-like output under external loads^51^. Accordingly, building a mixed ion-electron transport architecture that simultaneously maximizes ion-accessible interfacial area and provides a percolated electronic backbone is crucial for converting ionic polarization into continuous, load-matched energy harvesting^52^. In this context, though porous carbon is widely used as conductive scaffolds, their thermoelectric potential remains underexplored as a thermoelectric media, largely due to the limited ability to programmable control multiscale transport pathways and ion/electron coupling within a single material.

**Fig. 6:**
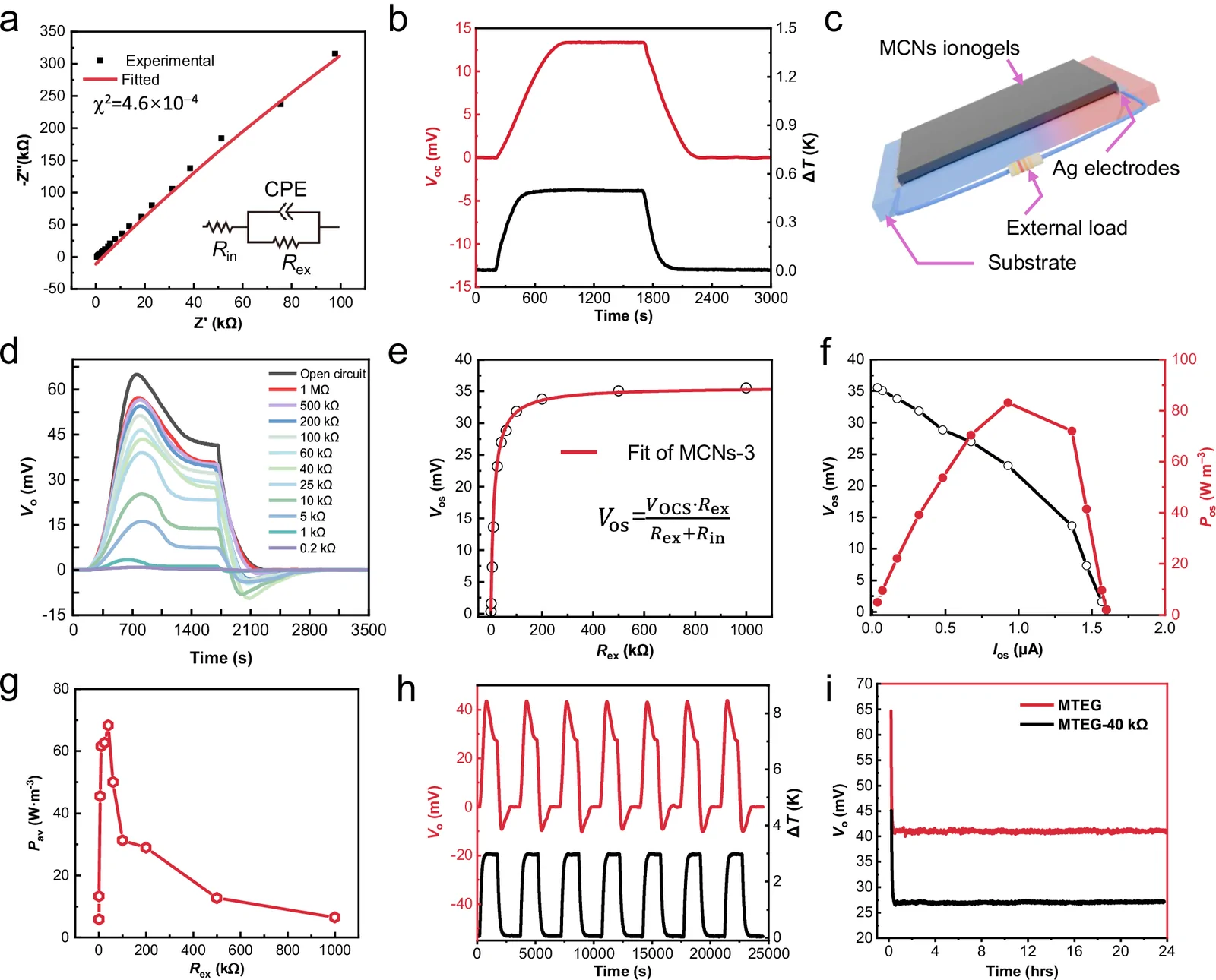
TE performance of MTEGs with hollow carbon nanofibers ionogels. **a** Fitting of Nyquist plots with an equivalent circuit, in which the ionic resistance (${R}_{{{{\rm{in}}}}}$) is in series with a parallel element consisting of an electronic resistance (${R}_{{{{\rm{ex}}}}}$) and a constant phase element (CPE). **b** ${V}_{{{{\rm{oc}}}}}$ response under the temperature of 0.5 K. The heating rate is 0.4 K min^−1^, and the relative humidity (RH) is 70%. **c** Schematic illustration of an MTEG device with Ag-paste electrodes and an external load. **d** Output voltage profiles on the various external loads connected to the MTEG. The temperature gradient is 3 K. External resistances are indicated. **e** Variation of ${V}_{{{{\rm{os}}}}}$ with ${R}_{{{{\rm{ex}}}}}$ for MTEGs (${V}_{{{{\rm{ocs}}}}}$ is the steady open-circuit voltage) and **f** dependences of ${V}_{{{{\rm{os}}}}}$ and steady power density (${P}_{{{{\rm{os}}}}}$) on steady current (${I}_{{{{\rm{os}}}}}$) for the MTEG. **g** Dependences of the average power density (${P}_{{{{\rm{av}}}}}$) with ${R}_{{{{\rm{ex}}}}}$ for the external load connected to MTEGs. **h** Output voltage on an external load of 25 kΩ connected to an MTEG under periodic temperature gradient cycles. **i** Evolution of output voltage on open-circuit and an external load of 40 kΩ connected to an MTEG, and the temperature gradient is 3 K.

The thermoelectric performance of MCNs-based ionogels is governed by the coupled transport of ions and electrons, which is intrinsically influenced by the structural characteristics of the carbon frameworks. To demonstrate their functional potential, four representative architectures, including ordered mesoporous carbon nanofibers (MCNs-1), hollow carbon nanofibers (MCNs-3), golf ball-like nanospheres (MCNs-10) and multichambered nanospheres (MCNs-17) were incorporated into ionogels to construct mixed ion-electron thermoelectric generators (MTEGs) (Fig. 6c). In this framework, an ionic thermodiffusion-driven thermopower is coupled with a percolated electronic pathway provided by the conductive carbon scaffold, and the ionogel supplies a large thermopower via the Soret effect, whereas the percolated electronic pathway provided by carbon network enables charge compensation and transport under a steady temperature gradient, rendering the device behavior analogous to conventional load-matched thermoelectric generators rather than purely capacitive ionic thermoelectric responses^53,54^.

The mixed-conduction characteristics and thermoelectric performance of MCNs-ionogels were systematically evaluated by electrochemical impedance spectroscopy (EIS) and temperature-gradient measurements, including output voltage (${V}_{{{{\rm{o}}}}}$), open-circuit voltage (${V}_{{{{\rm{oc}}}}}$) transients, load-dependent steady output (${V}_{{{{\rm{os}}}}}$), and power density. EIS reveals that the MCNs ionogels exhibit near-linear Nyquist characteristics and can be well fitted by an equivalent-circuit model, confirming the coexistence of ionic and electronic transport pathways in the MCNs ionogels (Fig. 6a, Supplementary Figs. 41 and 42). The extracted transport parameters indicate that the pristine ionogel is essentially ion-conducting (${\sigma }_{{{{\rm{i}}}}}$ = 1.17 S m^−1^, ${\sigma }_{{{{\rm{e}}}}}$ = 6.02 × 10^−15^ S m^−1^), while MCNs-10/17 ionogels remain largely ion-dominated (${\sigma }_{{{{\rm{i}}}}}$ = 1.44  S m^−1^ and 1.10 S m^−1^; ${\sigma }_{{{{\rm{e}}}}}$ = 6.02 × 10^−15^ S m^−1^ and 6.02 × 10^−13^ S m^−1^), respectively. In contrast, MCNs-1 and MCNs-3 ionogels exhibit markedly enhanced electronic conductivities (${\sigma }_{{{{\rm{e}}}}}$ = 7.00 × 10^−7^ S m^−1^ and 4.91 × 10^−7^ S m^−1^) while maintaining moderate ionic conductivities (${\sigma }_{{{{\rm{i}}}}}$ = 0.248 S m^−1^ and 0.361 S m^−1^), thereby establishing a mixed-conduction regime that is essential for continuous power delivery (Supplementary Fig. 43). Under a small temperature gradient (Δ*T* = 0.5 K), all devices generate a stable open-circuit thermovoltage after a short transient (Supplementary Fig. 44), corresponding to the thermopower of 23.4–26.8 mV K^−1^. Upon application and removal of Δ*T*, the ${V}_{{{{\rm{oc}}}}}$ responds promptly and reversibly, consistent with a thermodiffusion-driven ionic thermopower. Notably, the MCNs-3 ionogel exhibits the highest thermopower of 26.8 mV K^−1^, suggesting that abundant ion-accessible interfaces and shortened ion-diffusion pathways provided by the hollow/mesoporous fibrous architecture facilitate ionic thermodiffusion and interfacial polarization (Fig. 6b and Supplementary Fig. 45). When a rectangular thermal gradient (Δ*T* = 3 K) is applied, the output voltage profiles display a pronounced peak followed by a quasi-steady plateau (Fig. 6d), reflecting the competition between rapid ion redistribution and subsequent relaxation enabled by the mixed ion/electron conduction pathway, which is distinct from purely capacitive discharge responses^55^. As expected, decreasing the external load resistance (${R}_{{{{\rm{ex}}}}}$) reduces the steady-state output voltage, and the dependence of ${V}_{{{{\rm{oc}}}}}$ on ${R}_{{{{\rm{ex}}}}}$ follows the voltage-divider relationship (Fig. 6e), from which the internal resistance (${R}_{{{{\rm{in}}}}}$) was extracted. ${R}_{{{{\rm{in}}}}}$ derived from the ${V}_{{{{\rm{os}}}}}-{R}_{{{{\rm{ex}}}}}$ fitting equals 14.01 kΩ for MCNs-1 and 12.52 kΩ for MCNs-3 ionogels, consistent with the corresponding ionic resistances (${R}_{{{{\rm{in}}}}}$ = 14.57 and 10.01 kΩ) derived from EIS fitting (Supplementary Fig. 43), indicating that the device-level internal resistance is primarily governed by ion transport in the ionogel matrix^56^. Component-removal controls show that omitting melamine largely preserves morphology but decreases electronic conductivity (Supplementary Fig. 46), suggesting a partial contribution of N-containing species to electronic transport. In contrast, TOP removal markedly alters particle size, pore architecture, and carbon connectivity, indicating that the P-containing component mainly contributes through its structure-regulating role. The thermoelectric response is therefore attributed to the combined effects of surface chemistry, ion-accessible hollow/mesoporous architecture, and electronic percolation (Supplementary Fig. 47).

By sweeping ${R}_{{{{\rm{ex}}}}}$ from 0.2 to 1000 kΩ and converting the steady-state response to ${I}_{{{{\rm{os}}}}}$ and ${P}_{{{{\rm{os}}}}}$, the power-output curves exhibit a typical arch-shaped dependence (Fig. 6f). For MCNs-3 ionogels, ${V}_{{{{\rm{os}}}}}$ increases from 0.32 mV (0.2 kΩ) to 35.54 mV (1000 kΩ), while ${I}_{{{{\rm{os}}}}}$ decreases from 1.6 to 0.0355 μA; the corresponding power density rises from 1.98 W m^−3^ to a maximum of 83.10 W m^−3^ at ${R}_{{{{\rm{ex}}}}}$ = 25 kΩ and then gradually declines to 4.88 W m^−3^ at 1000 kΩ. For MCNs-1 ionogels, ${V}_{{{{\rm{os}}}}}$ increases from 0.25 mV (0.2 kΩ) to 29.57 mV (1000 kΩ), and ${P}_{{{{\rm{os}}}}}$ peaks at 48.68 W m^−3^ at ${R}_{{{{\rm{ex}}}}}$ = 25 kΩ before decaying to 3.38 W m^−3^ at 1000 kΩ, consistent with the matched-load behavior at the 10^4^ Ω scale (Supplementary Fig. 48). The average power density over a full heating–cooling cycle (${P}_{{{{\rm{av}}}}}$) also exhibits a clear dependence on ${R}_{{{{\rm{ex}}}}}$ (Fig. 6g and Supplementary Fig. 49). MCNs-3 ionogels achieve a maximum ${P}_{{{{\rm{av}}}}}$ of 68.32 W m^−3^ at ${R}_{{{{\rm{ex}}}}}$ = 40 kΩ, while MCNs-1 ionogels deliver a maximum ${P}_{{{{\rm{av}}}}}$ of 63.01 W m^−3^ at ${R}_{{{{\rm{ex}}}}}$ = 25 kΩ. Under an external load of 40 kΩ, MCNs-3 MTEG deliver a steady thermopower of 12.33 mV K^−1^, which is higher than those of many representative electronic, ionic, and hybrid thermoelectric materials summarized in Supplementary Table 2. Beyond instantaneous performance, the MTEGs demonstrate robust operational stability: repeated rectangular thermal cycling over 25,000 s produces reproducible voltage outputs without detectable degradation (Fig. 6h), and stable voltage can be maintained for at least 24 h under a constant gradient when connected to an external load (Fig. 6i).

Structural variations lead to markedly different power outputs; for example, MCNs-10 and MCNs-17 ionogels deliver ${P}_{{{{\rm{av}}}}}$ limited to ~10–12 W m^−3,^ which are lower than that of MCNs-3 ionogels (Supplementary Fig. 50). Moreover, the higher electronic conductivity of MCNs-17 ionogels (${\sigma }_{{{{\rm{e}}}}}$ = 6.02 × 10^−13^ S m^−1^) than MCNs-10 ionogels (${\sigma }_{{{{\rm{e}}}}}$ = 6.02 × 10^−15^ S m^−1^) indicates that reducing particle size and introducing multichambered structures can partially enhance electronic coupling between carbon domains. However, both systems remain predominantly ion-conducting, and the lack of a continuous electronic percolation network severely limits effective charge compensation under external loading. In this context, the reduced particle size of MCNs-17 may facilitate faster ion redistribution under a temperature gradient, yet this advantage is insufficient to overcome the intrinsic limitation imposed by discontinuous electronic pathways.

## Discussion

Based on the above observations and results, we propose a liquefied-wood-enabled emulsion-mediated micelle self-assembly framework for regulating morphologies and architectures in mesoporous carbon. In this system, TOP partitions into the hydrophobic PPO-rich domains of F127-based composite micelles and acts as a hydrophobic swelling agent. Besides, it forms a TOP-rich oil phase and creates oil/solvent interfacial regions for precursor–template organization. F127, TOP, and LWMF species are proposed to form F127/resin/TOP co-assembled micellar domains. The coexistence of nanoscale micelle species and micron-sized TOP-rich droplets provides both bulk solvent domains and TOP-rich oil/solvent interfacial regions for assembly. Depending on the formulation, these composite micelle domains can mainly organize in the continuous ethanol/water solvent phase or become enriched near the TOP-rich oil/solvent interface. To describe the competition between homogeneous aqueous assembly and interface-associated organization, we use an effective interfacial-energy/interfacial-affinity descriptor:

1 $$\Delta {\sigma }_{{{{\rm{eff}}}}}{=\sigma }_{{{{\rm{M}}}}-{{{\rm{S}}}}}^{{{{\rm{eff}}}}}+{\sigma }_{{{{\rm{M}}}}-{{{\rm{O}}}}}^{{{{\rm{eff}}}}}-{\sigma }_{{{{\rm{O}}}}-{{{\rm{S}}}}}^{{{{\rm{eff}}}}}$$

where M denotes the F127/resin/TOP co-assembled micellar domain, O denotes the TOP-rich oil phase, and S denotes the ethanol/water solvent phase. Here, ${\sigma }_{{{{\rm{M}}}}-{{{\rm{S}}}}}^{{{{\rm{eff}}}}}$, ${\sigma }_{{{{\rm{M}}}}-{{{\rm{O}}}}}^{{{{\rm{eff}}}}}$ and ${\sigma }_{{{{\rm{O}}}}-{{{\rm{S}}}}}^{{{{\rm{eff}}}}}$ represent the effective micelle/resin–solvent, micelle/resin–oil and oil–solvent interfacial-energy terms, respectively. This descriptor does not represent the static interfacial tension of a simple binary liquid/liquid interface. Instead, it describes the effective interfacial-energy change associated with the dynamic replacement of the original oil/solvent interface by micelle/resin-containing interfaces during reactive emulsion assembly. In this framework, a positive $\Delta {\sigma }_{{{{\rm{eff}}}}}$ favors spreading or more homogeneous isotropic assembly, whereas a more negative or less positive $\Delta {\sigma }_{{{{\rm{eff}}}}}$ disfavors complete spreading and promotes localized island-like nucleation and anisotropic growth at the interface. Increasing TOP content strengthens PPO–TOP and resin/TOP hydrophobic interactions, thereby improving the compatibility between the F127/resin/TOP micellar domains and the TOP-rich oil phase and decreasing ${\sigma }_{{{{\rm{M}}}}-{{{\rm{O}}}}}^{{{{\rm{eff}}}}}$. At the same time, TOP incorporation increases the hydrophobic character and swelling degree of the micellar domains, making full exposure of PPO/TOP-rich regions to the ethanol/water solvent less favorable and altering ${\sigma }_{{{{\rm{M}}}}-{{{\rm{S}}}}}^{{{{\rm{eff}}}}}$. The relative decrease in ${\sigma }_{{{{\rm{M}}}}-{{{\rm{O}}}}}^{{{{\rm{eff}}}}}$ with respect to ${\sigma }_{{{{\rm{M}}}}-{{{\rm{S}}}}}^{{{{\rm{eff}}}}}$ promotes the enrichment of F127/resin/TOP micellar domains at the TOP-rich interfacial region, thereby facilitating interface-associated anisotropic organization. This interpretation is consistent with the TOP-dependent morphology and pore-architecture evolution. In the absence of TOP, the system lacks a TOP-rich oil phase and mainly follows a homogeneous soft-template assembly pathway in the solvent phase, producing three-dimensional particles with ordered mesopores. Introducing a small amount of TOP creates a reactive emulsion while maintaining a strong contribution from homogeneous micelle organization, leading to ordered mesoporous nanofibers with multiple parallel mesochannels. Increasing TOP content enhances hydrophobic swelling and strengthens the contribution of interface-associated organization, which reduces the density of ordered mesochannels and promotes the formation of hollow carbon nanofibers. At higher TOP content, excessive swelling or an altered oil/micelle balance further changes the interfacial assembly behavior, giving rise to bowl-like nanospheres or irregular lamellar aggregates.

This interfacial-affinity balance contributes to the assembly-locus selection by biasing where micelle organization preferentially occurs, but does not independently determine the final morphology and mesostructure. Micelle packing provides another useful but qualitative framework for understanding the internal pore-architecture evolution. In the present F127/TOP/ethanol/water/LWMF reactive system, a single classical packing parameter $p=V/{a}_{0}{l}_{c}$ cannot be unambiguously calculated because the hydrophobic volume, effective headgroup area, and hydrophobic chain length are all formulation-dependent and dynamically altered during TOP partitioning, ethanol solvation, and resin polymerization. Therefore, the packing-parameter concept is used here as a qualitative micelle-curvature framework rather than a quantitatively predictive parameter. TOP incorporation into PPO-rich domains increases the effective hydrophobic volume and favors lower-curvature micellar organizations. The observed evolution from ordered channels to hollow, curved, and lamellar-like architectures is therefore consistent with a formulation-dependent shift in preferred micelle curvature/packing tendency.

Ethanol regulates the assembly pathway through a coupled solvation–curvature–kinetic effect. Increasing ethanol content improves the compatibility between the oil phase and the surrounding ethanol/water medium, thereby decreasing $\Delta {\sigma }_{{{{\rm{eff}}}}}$. Ethanol also alters ${\sigma }_{{{{\rm{M}}}}-{{{\rm{S}}}}}^{{{{\rm{eff}}}}}$ by changing the solvation environment of both PEO and PPO blocks, as well as hydroxyl-rich LWMF fragments, which modifies the effective micelle/solvent interaction and shifts the preferred organization of F127/resin/TOP composite micelles toward more highly solvated, higher-curvature and more isotropic aggregates. This interpretation is consistent with the ethanol-dependent transition from fibrous architectures with cylindrical mesopores to nanospheres with more spherical pore geometries. Ethanol also moderates the acid-catalyzed condensation of LWMF species, thereby altering the kinetic matching between micelle organization and polymerization-induced structural fixation. At moderate ethanol content, the reduced interfacial penalty, retained hydrophobic segregation, and appropriate polymerization rate allow controlled interfacial nucleation and anisotropic growth. At higher ethanol content, enhanced solvent compatibility, stronger micelle/precursor solvation, and slower condensation collectively weaken interface-associated anisotropic growth and shift the products toward isotropic or quasi-isotropic nanospheres.

**Fig. 7:**
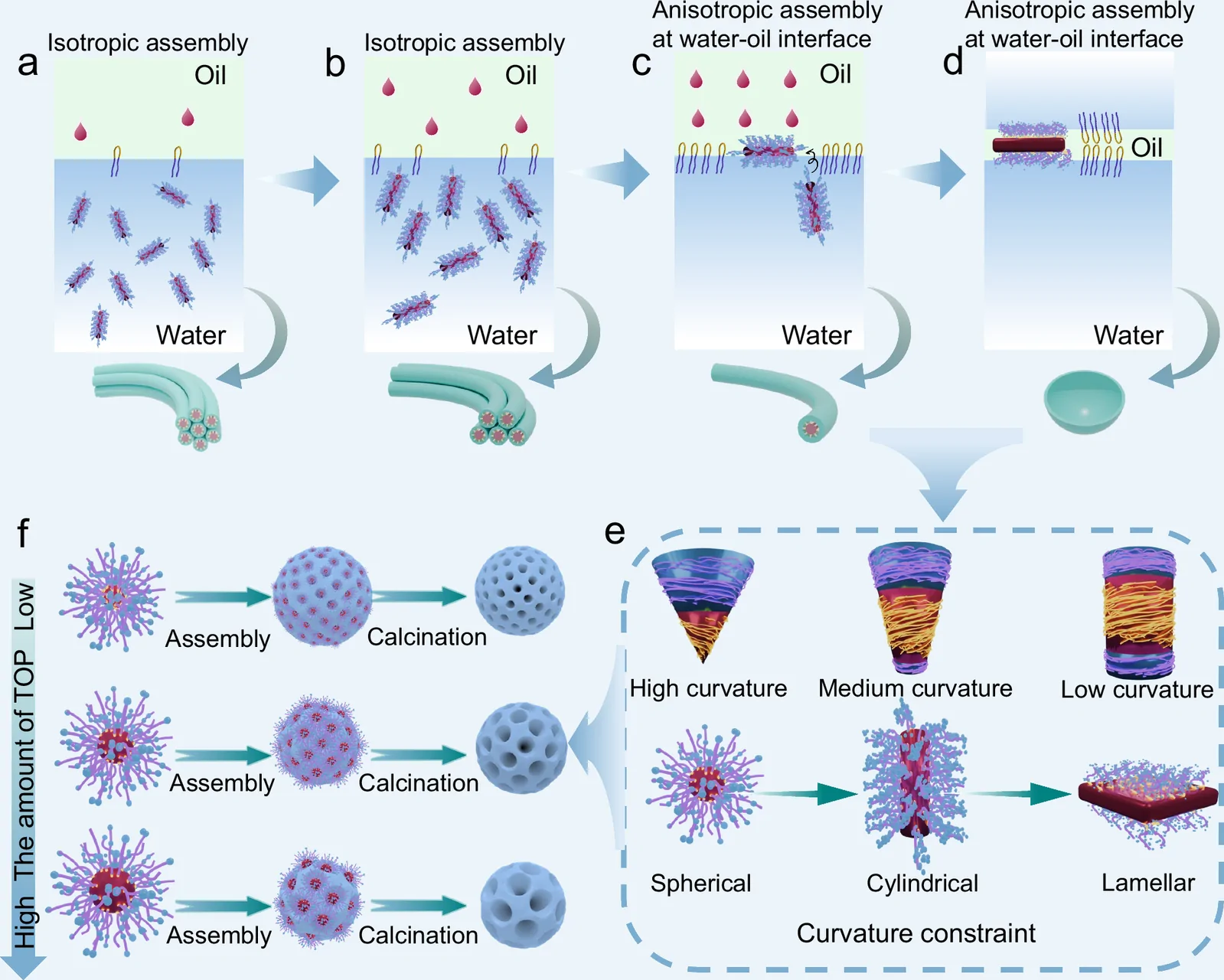
Schematic illustration of the proposed emulsion-mediated self-assembly strategy. **a**–**d** Transformation of micelle self-assembly patterns from isotropic patterns in aqueous to interfacial anisotropic modes. **e** Qualitative micelle-curvature/packing framework used to rationalize the observed pore-architecture evolution. **f** The swelling effect of TOP on the hydrophobic blocks of micelles.

Coarse-grained simulations provide additional support for this coupled mechanism (Supplementary Table 3). For formulation-dependent micelle packing, the classical packing parameter cannot be uniquely calculated in this reactive multicomponent system. A contact-derived packing-tendency proxy and aggregate-shape descriptors were used to compare relative packing tendencies (Supplementary Table 4). The increase in the normalized local packing proxy and aggregate anisotropy under high-TOP conditions supports a shift from more isotropic/high-curvature aggregates toward elongated or lower-curvature organizations (Supplementary Figs. 51 and 52). Interfacial simulation models further show that F127/TOP/LWOH aggregates organize near the TOP-rich oil/water interfacial region, where TOP/PPO-rich domains provide hydrophobic anchoring while PEO/LWOH hydroxyl-rich regions maintain polar contacts with the solvent (Supplementary Table 5). These results support the interpretation that increasing TOP content enhances interfacial coupling and anisotropic organization of F127/resin/TOP micellar domains (Supplementary Fig. 53). In addition, LWOH-removal simulations indicate that liquefied-wood-derived species bridge hydrophobic TOP/PPO-rich domains and polar PEO/ethanol/water-rich environments, supporting the active role of liquefied wood in precursor–template interaction, interfacial organization and framework fixation (Supplementary Fig. 54). Together, these simulations support the mechanism proposed in Fig. 7, in which TOP and ethanol regulate micelle curvature, TOP/water interfaces define favorable assembly loci and liquefied wood couples interfacial assembly with carbon-framework formation.

The morphology/mesostructure maps further clarify the structural windows accessible in this reactive emulsion system (Supplementary Fig. 55). The products can be classified into irregular/particle-like products, fiber/rod-like structures, hollow/curved architectures, and sphere-like architectures. In the TOP–EtOH map, low-EtOH/low-TOP conditions mainly favor fiber/rod-like structures, intermediate TOP promotes hollow/curved architectures, whereas high ethanol content shifts the products toward sphere-like morphologies. These boundaries are empirical transition regions inferred from adjacent experimental points, rather than sharp equilibrium phase boundaries, because the present system involves coupled emulsion formation, micelle organization, precursor condensation, and hydrothermal fixation. Additional TOP–temperature, TOP–F127, and HCl–EtOH maps further show that temperature, template concentration, and polymerization kinetics regulate the accessibility and stability of each structural regime. Additional control experiments further support the coupled mechanism. Variations in hydrothermal temperature, F127 dosage, HCl concentration, surfactant composition, and oil-phase chemistry all alter the final morphology and mesostructure (Supplementary Figs. 23–40), indicating that these parameters regulate the assembly pathway through coupled changes in interfacial energy, micelle curvature, precursor-template interactions, and polymerization kinetics. Therefore, the observed morphology evolution should be interpreted as a cooperative thermodynamic-kinetic process, in which effective interfacial-affinity-biased locus selection and micelle-curvature regulation guide the assembly pathway, while polymerization kinetics fixes or disrupts the evolving mesostructure depending on the kinetic matching condition.

The mixed ion-electron thermoelectric behavior of ionogels is commonly governed by the balance between electronic percolation and ionic transport within the composite network^57^. As summarized in Supplementary Table 6, the superior performance of MCNs-3 cannot be attributed solely to the surface area or pore volume, but rather to the coupling of one-dimensional electronic percolation, hollow/mesoporous ion-accessible interfaces, reduced ion-diffusion pathways, and lower device-level internal resistance. One-dimensional ordered mesoporous carbon nanofibers provide continuous carbon backbones that facilitate long-range electronic connectivity, while their ordered mesoporous channels offer accessible ion-transport pathways along the fibrous framework. Hollow carbon nanofibers retain this one-dimensional electronic connectivity and introduce additional internal cavities and thin mesoporous shells, enabling ion access to both outer and inner carbon surfaces and shortening radial ion-diffusion pathways. This architecture is therefore more effective in coupling ionic polarization with electronic charge compensation under a temperature gradient^58,59^. By contrast, zero-dimensional nanosphere-based architectures, including golf ball-like and multichambered nanospheres, provide abundant local ion-accessible surfaces, internal compartments, and rough external interfaces, which are beneficial for ion accumulation and interfacial polarization^60^. However, their zero-dimensional particle morphology makes long-range electron transport more dependent on discrete interparticle contacts. Such discontinuous electronic pathways are less favorable for forming a percolated carbon network in the ionogel matrix, thereby limiting electronic charge compensation and steady power delivery under external loading^61^. These considerations explain why the hollow carbon nanofiber-based ionogels exhibit the most balanced mixed ion-electron transport among the representative architectures examined.

In summary, we have demonstrated a biomass-derived self-assembly strategy for constructing mesoporous carbons from liquefied wood within a single synthetic system. This approach features the introduction of TOP, which not only expands the hydrophobic blocks of composite micelles but also serves as an interfacial mediator to establish a stable oil-water interface, inducing the composite micelle from homogeneous self-assembly to anisotropic overgrowth, which drives the cylindrical micelles assembled from ordered nanofibers into hollow nanofibers. Furthermore, adjusting the ethanol content provides an additional handle to modulate micelle self-assembly, yielding diversely carbon nanospheres, including bowl-like, gear-like, and dendritic architectures. The resultant hollow carbon nanofibers feature high pore volume (~0.90 cm^3^ g^−1^), abundant N content (~6.5 wt%), and unique hollow architectures. The fabricated hollow carbon nanofiber-based MTEGs deliver a high thermopower of 12.33 mV K^−1^ and a sustained average power density of 68.32 W m^−3^ under an external load of 40 kΩ, while maintaining stable operation over extended thermal cycling, demonstrating reliable device-level energy conversion. This strategy offers inspiration for designing mesoporous carbon materials from biomass with programmable morphology and structure for advanced applications.

## Methods

### Preparation of liquefied wood

Larch sawdust was procured from a local lumber mill situated in Harbin, Heilongjiang Province. Before use, the dried larch sawdust was ground and passed through a 60-mesh sieve. Dried larch sawdust (1.0 g) was dispersed in a mixture of H_3_PO_4_ (85%, 0.2 mL), H_2_SO_4_ (98%, 0.1 mL), and phenol (3 mL) at 60 °C under stirring at 180 rpm. The mixture was then stirred at 120 °C for 2 h and cooled to room temperature. Methanol (10 mL) was added portionwise, and the resulting mixture was filtered. The filtrate was neutralized with 5 M NaOH solution, followed by rotary evaporation at 52 °C to remove methanol and water and obtain LW.

### Synthesis of hollow carbon nanofibers

The hollow carbon nanofibers were synthesized through a reactive emulsion-mediated self-assembly strategy. In a typical process, 0.30 g of LW, 0.20 mL of trioctyl phosphate (99%, TOP), 1.10 g of Pluronic F127, and 0.15 g of melamine were added into a mixed solution containing ethanol (10 mL), water (50 mL), and HCl (37%, 10 mL). The mixture was stirred at 500 rpm for 30 min at room temperature, followed by the addition of 0.76 g of formaldehyde (37%). After stirring for another 60 min to form a homogeneous milk-like emulsion, the mixture was transferred into a 100 mL Teflon-lined stainless-steel autoclave and heated at 150 °C for 10 h. After cooling to room temperature, the polymeric products were collected by filtration and washed with water and ethanol. Finally, the dried polymeric composites were thermally treated at 300 °C and then carbonized at 800 °C under N_2_ atmosphere to obtain hollow carbon nanofibers.

### Fabrication of MCN-based ionogels and mixed ion–electron thermoelectric generators

The MCN-based ionogels were prepared by dispersing the MCNs and sodium dodecyl sulfate in water at a mass ratio of 3:1, followed by sonication for 15 min. A 2 wt% aqueous gelatin solution was prepared by dissolving 0.2 g of gelatin in 9.8 g of water at 60 °C. EMIM:DCA was then added at a gelatin/EMIM:DCA mass ratio of 1:4. After stirring at 60 °C for 10 min, the MCN/SDS dispersion was mixed with the gelatin/ionic-liquid matrix. The resulting mixture was cast into molds and dried at room temperature for 24 h to form MCN-based ionogels. The MCN loading was 0.3 wt%. Mixed ion–electron thermoelectric generators were assembled by painting silver electrodes onto both ends of the ionogel samples, followed by drying at 60 °C before thermoelectric measurements.

## Supplementary information

Supplementary Information

Transparent Peer Review File

## Source data

Source data

## Data Availability

Data supporting the findings of this study are included within the article and Supplementary Information files. Data underlying this study are available from the corresponding authors upon request. Source data are provided with this paper.
