# Supplementary material for: Programmable mesoporous carbon architectures from liquefied wood via reactive-emulsion-mediated self-assembly
Source: Nat Commun. 2026 Aug 29;17:10307. doi: 10.1038/s41467-026-77269-7 (PMC13619605; doi:10.1038/s41467-026-77269-7)
Supplement: Supplementary file 1 [file 41467_2026_77269_MOESM1_ESM.pdf]

Supplementary Information

Programmable mesoporous carbon architectures from liquefied wood via reactive-emulsion-mediated self-assembly

Yang Li[1], Kun Zhang[1], Shenghui Jiao[1], Guangying Li[1], Yanhong Liu[1], Shirui Zou[1], Rui Teng[1], Hongjian Fu[1], Chunhui Ma[1], Sha Luo[1], Wei Li[1]*, Zaiwang Zhao[2]*, Shouxin Liu[1,3]*

[1]Key Laboratory of Utilization of Woody Oil Resource, Northeast Forestry University, 150040, Harbin, P.R. China

[2]College of Energy Materials and Chemistry, Inner Mongolia Key Laboratory of Low Carbon Catalysis, College of Chemistry and Chemical Engineering, Inner Mongolia University, Hohhot 010070, P.R. China

[3]Key Laboratory of Bio-based Material Science and Technology of the Ministry of Education, Northeast Forestry University, 150040, Harbin, P.R. China

*To whom correspondence should be addressed.

Email: liwei19820927@126.com (W. L.);

zwzhao@imu.edu.cn (Z. Z.);

liushouxin@nefu.edu.cn (S. Liu).

Contents

1. Supplementary Methods

    1.1 Chemicals and materials

    1.2 Synthesis process

    1.3 Characterization

    1.4 Application measurements

    1.5 CG simulation of formulation-dependent precursor organization

    1.6 Interfacial CG simulation of TOP-induced localization and anisotropic assembly

    1.7 LWOH-removal simulation for clarifying the role of liquefied wood

2. Morphology and structure regulation

    2.1 Synthesis parameters of MCNs (Tab. 1)

    2.2 Formation process of hollow carbon nanofibers (Fig. 1, 2)

    2.3 Characterization of the hollow carbon nanofibers (Fig. 3–6)

    2.4 Control experiments (Fig. 7–40)

3. Thermoelectric application of MCNs-based ionogels (Fig. 41–50, Tab. 2, 6)

4. Mechanistic analysis and CG simulations (Fig. 51–55, Tab. 3–5)

5. Supplementary References

1 Supplementary Methods

1.1 Chemicals and materials

Larch sawdust was procured from a local lumber mill situated in Harbin, Heilongjiang Province. Before use, the dried larch sawdust was ground and passed through a 60-mesh sieve. Triblock copolymer Pluronic F127 ($PEO_{106}PPO_{70}PEO_{106}$, M_w = 12600), Pluronic F108 ($PEO_{132}PPO_{50}PEO_{132}$, M_w = 14600), Pluronic P123 ($PEO_{20}PPO_{70}PEO_{20}$, M_w = 5800) and Pluronic P84 ($PEO_{19}PPO_{43}PEO_{19}$, M_w = 4200) were purchased from Sigma-Aldrich. Phosphoric acid (H_3PO_4, 85%), hydrochloric acid (HCl, 37%), sulfuric acid (H_2SO_4, 98%), melamine and formaldehyde (37%) were purchased from Tianjin Kermel Chemical Corp. Phenol (AR), trioctyl phosphate (TOP, 99%), triamyl Phosphate (TAP, 99%), tributyl phosphate (TBP, 99%), triethyl phosphate (TEP, 99%), 1-ethyl-3-methylimidazolium dicyanamide (EMIM: DCA, ≥98%), sodium dodecyl sulfate (SDS, ≥99%) and gelatin (Type A, from porcine skin, Bloom 300) were used as received from Sigma-Aldrich. All the reagents were used directly without further purification. Deionized water (18.2 MΩ·cm, Millipore) was used for all experiments. Silver paste (Leitsilber 200) was purchased from Ted Pella Inc., and silicon wafers were purchased from the Guangzhou Fangdao Semiconductor Co., Ltd.

1.2 Synthesis process

Preparation of LW. The LW was prepared according to our previous work[1–3]. Typically, 1 g of dried larch sawdust was dispersed in a mixed solution consisting of 0.2 mL H_3PO_4, 0.1 mL H_2SO_4 and 3 mL phenol at 60 °C under stirring (180 rpm). Following stirring continuously at 120 °C for 2 hours and subsequent cooling to room temperature, 10 mL of methanol was added portionwise into the mixture. The resulting mixture was then filtered to recover the filtrate, and the collected liquid was neutralized with NaOH solution (5 M). Finally, the LW was obtained by rotary evaporation at 52 °C to remove

water and methanol. The LW used in this work is a biomass-derived oligomeric mixture rather than a single molecular compound. As reported in our previous studies, the liquefaction of larch sawdust in phenol under acid catalysis converts the rigid lignocellulosic structure into soluble phenolic-rich oligomers containing abundant hydroxyl and aromatic units[1,3]. These chemical features allow LW to participate in subsequent melamine–formaldehyde co-polymerization and to interact with Pluronic F127 through hydrogen bonding and hydrophobic/aromatic interactions. Therefore, in the present reactive-emulsion-mediated assembly system, LW serves not only as a carbon precursor, but also as an active assembly precursor that contributes to precursor–template interaction, interfacial affinity and polymerization-induced structural fixation. The detailed molecular-weight distribution and spectroscopic characterization of LW, including GPC, FTIR, ^{13}C NMR and ^{31}P NMR analyses, have been reported in our previous studies. GPC analysis revealed a bimodal molecular-weight distribution, with the oligomeric fraction showing a molecular weight of approximately 542 g mol^{-1}. FTIR and ^{13}C NMR analyses confirmed the presence of abundant hydroxyl groups and phenolic/aromatic structures, while quantitative ^{31}P NMR analysis identified aliphatic, guaiacyl, catechol-type, p-hydroxyphenyl and carboxylic acid hydroxyl groups[1–3].

Preparation of hollow carbon nanofibers. The resin polymers with various mesostructures were synthesized via a versatile emulsion-mediated assembly approach, where amphiphilic triblock copolymer Pluronic F127 as the soft-template, TOP molecule as the swelling agent and emulsifier, LW as the interfacial regulator and carbon source in the HCl/ethanol/water/mixture, followed by hydrothermal polymerization and carbonization. In a typical process, 0.30 g of LW, 0.20 mL of TOP, 1.1 g of F127, and 0.15 g of melamine were continuously added into a mixture of ethanol (10 mL), water (50 mL) and HCl (10 mL). The final HCl concentration is 1.71 M. The solution was stirred at

500 rpm for 30 min at room temperature for complete dissolution. Followed by the addition of 0.76 g of formaldehyde, the resulting solution was stirred for another 60 min to form a homogeneous milk-like emulsion. The stirring bar was 2 cm in length. Then the above mixture was placed into a 100 mL teflon-lined autoclave for hydrothermal treatment at 150 °C for 10 h. After the autoclave was cooled, the as-made polymeric composites were collected by filtration and washed with water and ethanol. After the dried polymeric composites were preheated at 300 °C and further carbonized at 800 °C under nitrogen atmosphere with a heating rate of 1 °C min^{-1}, the polymeric composites could be easily converted into hollow carbon nanofibers[4,5].

Fabrication of ionogels. The prepared mesoporous carbon nanoparticles and SDS were dispersed in an aqueous solution at a mass ratio of 3:1 and continuously sonicated with a power of 100 W at an amplitude of 20% for 15 minutes to obtain a homogeneous MCNs/SDS system. An aqueous gelatin solution (2%) was prepared by dissolving 0.2 g of gelatin in water at 60 °C with the total mass of the solution adjusted to 10.0 g. The 1-ethyl-3-methylimidazolium dicyanamide (EMIM):DCA was then added at a mass ratio of gelatin: EMIM:DCA = 1: 4 and stirred at 60 °C for 10 min. Subsequently, the MCNs/SDS dispersion was added to the gelatin/IL matrix to obtain a mesoporous carbon loading of 0.3 wt%. Finally, the prepared mixtures were cast into molds with dimensions of 2×1×0.2 cm^3 and dried at room temperature for 24 h to obtain the ionogels[5].

Control experiments in the emulsion-mediated assembly strategy[6,7].

Change in the TOP amount. The synthetic processes were performed following the standard hollow-carbon-nanofiber synthesis procedure described above except that the amount of TOP was changed from 0.2 mL to 0, to 0.05, to 0.1 and to 0.3 mL. The formed polymer products were collected and calcined at 300 °C for 2 h and subsequently at 800 °C for 2 h.

Change in the template. The synthetic processes were performed following the standard hollow-carbon-nanofiber synthesis procedure described above, except that the template was replaced by 0.5 g F108 + 0.6 g F127, 0.5 g P123 + 0.6 g F127 and 0.5 g P84 + 0.6 g F127, respectively.

Change in the ethanol content. The synthetic processes were conducted following the standard hollow-carbon-nanofiber synthesis procedure described above, except that the ethanol volume fraction in the reaction system was changed from 14, to 7, to 28 and to 36%, which corresponds to the volumes of 5, 20, and 25 mL. Besides, the amount of TOP was set as 0.05, 0.1, 0.2 and 0.3 mL in different fractions of ethanol systems. The resultant polymeric products were collected and calcined at 300 °C for 2 h and subsequently at 800 °C for 2 h. Notably, when the ethanol was 25 mL (36%), a series of carbon nanospheres with various mesostructures was obtained by varying the amount of TOP.

Change in the hydrothermal temperature. The synthetic processes were carried out following the standard hollow-carbon-nanofiber synthesis procedure described above, except that the hydrothermal temperature was changed from 150 °C to 130 and to 180 °C, and the amount of TOP was set as 0.1, 0.2 and 0.3 mL at different temperatures. Additionally, the hydrothermal temperature also changed to 130 and 180 °C when the ethanol volume was 25 mL (36%), and the TOP amount was set as 0.1, 0.2 and 0.3 mL at different temperatures. The polymer products were collected and calcined at 300 °C for 2 h and subsequently at 800 °C for 2 h.

Change in F127 amount. The synthetic processes were carried out following the standard hollow-carbon-nanofiber synthesis procedure described above, except that the F127 amount was changed from 1.1 g to 0.5, to 0.8 and to 1.5 g, and the amount of TOP was set as 0.05, 0.1, 0.2 and 0.3 mL, respectively. Additionally, the F127 amount also changed to 0.5, to 0.8 and to 1.5 g when the ethanol volume was 25 mL (36%), and the TOP amount was set as 0.1, 0.2, and 0.3 mL under different F127 amount

conditions. The polymer products were collected and calcined at 300 °C for 2 h and subsequently at 800 °C for 2 h.

Change in the HCl concentration. The synthetic processes were performed following the standard hollow-carbon-nanofiber synthesis procedure described above, except that the HCl concentration in the reaction system was adjusted from 1.71 M to 1.37, to 1.54, to 1.89, and to 2.06 M. Additionally, when the ethanol volume in the system was 25 mL (36%), the HCl concentration was similarly set as described above. The polymer products were collected and calcined at 300 °C for 2 h and subsequently at 800 °C for 2 h.

Change in the alkyl chain lengths of the oil phase. The synthetic processes were performed following the standard hollow-carbon-nanofiber synthesis procedure described above, except that the TOP was replaced by TAP, TBP and TEP.

The as-prepared carbon nanoparticles were denoted as MCNs-x. The detailed experimental parameters are summarized in the Supplementary Table 1.

1.3 Characterization

Scanning electron microscopy (SEM) images were carried out on a JEOL-JSM-7500 microscope (Japan) with a landing energy of 5 kV. Transmission electron microscopy (TEM) was performed on a JEOL-JEM-F200 (Japan) with an operation voltage of 200 kV equipped with EDS detectors for elemental mapping analysis. Samples used for SEM and TEM analyses were prepared by dropping the MCNs in ethanol on silicon and carbon-coated copper grids , respectively. Wide-angle X-ray diffractometer (XRD) patterns were recorded on X-Pert 3 Powder (Netherlands) with Cu Kα radiation (40 kV, 40 mA). Small-angle X-ray scattering (SAXS) was carried out from an Anton-Paar SAXS system using Cu-Kα radiation. Thermogravimetric analysis (TGA) curves were conducted on TGA

8000 analyzer from 30 to 800 °C in N_2 atmosphere at a heating rate of 10 °C min^{-1}. Raman spectra were collected on a Raman spectrometer (Invia, UK). X-ray photoelectron spectroscopy (XPS) was recorded on Thermo Scientific K-Alpha. All the binding energies were modified using the C 1*s* standard peak at 284.8 eV. N_2 adsorption-desorption isotherms were measured on BELSORP-MAX at 77 K. Before analysis, all samples were degassed under vacuum at 200 °C for 6 h. The specific surface areas were calculated by using the Brunauer-Emmett-Teller (BET) method. The pore-size distributions were obtained from the N_2 adsorption branch using a Non-Local Density Functional Theory (NLDFT) model for carbon materials to evaluate the hierarchical pore structures.

1.4 Application measurements

1.4.1 Thermoelectric properties measurements

Thermovoltage and thermopower were measured using a custom-built measurement system[8,9]. The thermovoltage and temperatures at hot and cold sides were recorded with a Keithley 2000 source meter and type-T thermocouples, respectively, while a UT325 thermometer was employed to verify temperature stability. Both the hot and cold sinks were dimensioned as 4×4 cm^2, with a separation of 2 cm. Two silver electrodes (1 mm in width) were fabricated by directly painting silver paste onto the sample surface, with an interelectrode separation of 1 mm. Subsequently, the silver electrodes were dried on a hot plate at 60 °C for 15 min.

Electrochemical impedance spectroscopy was performed using a Solartron 1470E electrochemical workstation. The measurement was conducted with an AC amplitude of 10 mV, and the frequency was swept from 100 kHz to 0.1 Hz. Before testing, the as-prepared ionogels were sandwiched between two parallel stainless steel electrodes. The ionic and electronic resistances were extracted by analyzing the Nyquist plots with an equivalent circuit. Equivalent-circuit fitting was performed in ZView over all

measured frequency points using complex nonlinear least-squares optimization with Calc-Modulus weighting. The conductivities (σ) were calculated in terms of the resistance (R) values using the equation below,

$$\sigma = \frac{l}{RA} \tag{1}$$

where l is the thickness of the sample, A is the electrode area, and R corresponds to the ionic or electronic resistance. All thermoelectric and conductivity measurements were carried out under ambient conditions with a controlled relative humidity (RH) of 70%.

1.4.2 Fabrication of MTEG Devices

Mixed ion-electron thermoelectric generators (MTEGs) were assembled following the same procedure for thermovoltage measurements. Silver electrodes were painted on both ends of each MCNs-based ionogel sample. The MTEGs were tested under a rectangular-wave thermal cycle with a temperature gradient of 3 K and a duration of 1500 seconds. The average power density (P_{av}) was calculated according to the following equation:

$$P_{av} = \frac{1}{V_{ol} \cdot \Delta t} \int \frac{V_{op}^2(t)}{R_{ex}} dt \tag{2}$$

where R_{ex} is the external resistance, V_{op} is the peak output voltage profile on the external load, V_{ol} is the volume of the ionogel between electrodes, and Δt is the duration of the thermal cycle from heater turn-on to turn-off.

1.5 Coarse-grained simulation of formulation-dependent contact-derived packing tendency and precursor morphology evolution

1.5.1 Model construction and composition-variation design

To support the analysis of precursor-stage organization under different formulation conditions, three formulation-guided coarse-grained (CG) models were constructed, namely P3-high EtOH, P1-base and P2-high TOP. These models were designed to compare the influence of two compositional variables,

TOP content and EtOH content, on the local organization of the F127/TOP/EtOH/LWOH precursor system. The key model names, bead labels and quantitative descriptors used in the CG simulations are defined in Supplementary Table 3 to avoid ambiguity between experimental components, CG residue names and analysis descriptors.

P1-base was used as the reference model, corresponding to the baseline condition with TOP = 0.1 mL and EtOH = 10 mL. P2-high TOP was constructed by increasing the number of TOP molecules from 40 to 120 while keeping the number of ETHX beads unchanged relative to P1-base. P3-high EtOH was constructed by increasing the number of ETHX beads from 9000 to 22500 while keeping the number of TOP molecules unchanged relative to P1-base. The numbers of F127M, LWOH and water beads were kept constant in the three models to allow a controlled comparison of the effects of TOP and EtOH contents.

The EtOH/H_2O ratios in the CG models were mapped according to the corresponding experimental EtOH/H_2O molar ratios, considering that one Martini water bead represents approximately four water molecules. Thus, the ETHX/W ratios in P1-base and P3-high EtOH correspond to the experimental EtOH/H_2O ratios for the 10 mL and 25 mL EtOH formulations, respectively. The TOP variation was represented by a threefold increase in the number of TOP molecules, consistent with the experimental change from 0.1 mL to 0.3 mL TOP.

LWOH was used as a phenolic-rich precursor surrogate to represent hydroxyl-containing aromatic units in the precursor system. The CG models were not intended to reproduce the full experimental composition on an absolute mass basis or to explicitly include all chemical reactions occurring during curing and carbonization. Instead, they were used as comparative models to examine how controlled variations in TOP and EtOH contents affect precursor-stage soft-template organization. The

corresponding CG compositions and key morphology descriptors are summarized in Supplementary Table 4.

1.5.2 Simulation conditions and analysis protocol

All coarse-grained (CG) molecular dynamics simulations were performed using GROMACS with Martini-type CG interaction parameters. The topology files used in the simulations included martini_v3.0.0.itp, martini_v3.0.0_solvents_v1.itp, martini_v3.0.0_ions_v1.itp, F127M_cg.itp, TOP_cg.itp, ETHX_cg.itp and LWOH.itp. F127M was represented by PEO-type polar beads and PPO-type hydrophobic beads. LWOH was used as a phenolic-rich precursor surrogate and was represented by hydrophobic/aromatic carbon beads (LWOH-C) and polar hydroxyl-rich beads (LWOH-OH). ETHX was used as the CG representation of ethanol, and Martini water beads were used as the solvent.

For each CG model, the initial structure was first subjected to energy minimization, followed by stepwise equilibration. A short NVT initialization step was performed with newly generated velocities to stabilize the minimized structure. Subsequent NVT soft-equilibration, gradual relaxation and NPT soft-equilibration steps were then performed before the production simulation. Periodic boundary conditions were applied in all three directions. The production simulation was performed for 15 ns in total, including an initial 2.5 ns run followed by a 12.5 ns continuation run.

For non-bonded interactions, the Verlet cutoff scheme was used. Electrostatic interactions were treated using the reaction-field method with a cutoff of 1.1 nm and a relative dielectric constant of 15. The van der Waals cutoff was set to 1.1 nm. Temperature coupling was performed using the velocity-rescaling thermostat. The main equilibration and production simulations used a time step of 0.01 ps, while the short NVT initialization step used a smaller time step of 0.002 ps to improve stability during the early relaxation stage. The simulations were used to compare the relative precursor-stage

organization under different composition-variation conditions, rather than to explicitly reproduce the polymerization process.

Quantitative analyses were performed using the last three representative frames. Specifically, the frames extracted at 7500, 10000 and 12500 ps from the 12.5 ns continuation trajectory correspond to cumulative simulation times of approximately 10.0, 12.5 and 15.0 ns, respectively. Contact analyses were performed using a 1.0 nm cutoff. The template core was defined using PPO and TOP beads, while the precursor core was defined using PPO, TOP and LWOH-C beads. The largest precursor core was used for shape-descriptor analysis.

1.5.3 Definition of the contact-derived packing-tendency proxy

Because the present system is a multicomponent reactive system rather than a single-component surfactant assembly, the classical molecular packing parameter cannot be directly calculated from uniquely defined hydrophobic volume, headgroup area and chain length. Nevertheless, the physical concept underlying the classical packing parameter of the balance between hydrophobic core volume/association and polar interfacial area/solvation remains useful for rationalizing micelle-curvature evolution. To extend this concept to the present multicomponent system, we defined a contact-derived packing-tendency proxy, p_{contact}, to compare the relative balance between hydrophobic and polar interfacial contributions among different formulations.

$$p_{\text{contact}} = (\text{CLWOH-C–TOP}+\text{CLWOH-C–PPO}+\text{CTOP–PPO})/(\text{CLWOH-OH–PEO}+\text{CLWOH-OH–ETHX}+\text{CTOP–PEO}) \quad (3)$$

where CLWOH-C–TOP, CLWOH-C–PPO and CTOP–PPO represent the hydrophobic contact contributions from the phenolic/aromatic carbon beads of LWOH, TOP and the PPO-rich hydrophobic domains of F127. CLWOH-OH–PEO, CLWOH-OH–ETHX and CTOP–PEO represent the polar

interfacial contact contributions from hydroxyl-rich LWOH beads, PEO segments and ETHX beads. This contact-based definition was used to compare the relative local packing tendency among different composition-variation models. p_{contact} is not equivalent to the classical molecular packing parameter. Instead, it is a contact-derived descriptor that translates the physical balance considered in the classical packing-parameter framework into a form applicable to the present multicomponent reactive emulsion.

A larger p_{contact} indicates a stronger contribution from hydrophobic association relative to polar interfacial solvation, which is expected to favor a higher local packing tendency and lower preferred curvature. Conversely, a smaller p_{contact} indicates stronger polar interfacial solvation relative to hydrophobic association, which is expected to favor a lower local packing tendency and higher preferred curvature. To facilitate comparison with the classical micelle-curvature/packing framework, p_{contact} was further normalized relative to the P1-base formulation to obtain a dimensionless packing-tendency index, p_{local}. The P1-base formulation was used only as an internal reference for normalization. Therefore, the resulting p_{local} values should be interpreted as relative, formulation-dependent indices rather than absolute classical packing parameters. They are used to compare whether TOP or ethanol shifts the precursor aggregates toward relatively higher or lower packing tendencies (Supplementary Fig. 51).

For presentation on a classical-packing-parameter-inspired scale, P1-base was assigned a reference value of p_{local} = 0.40, a value chosen only as an internal normalization convention to represent an intermediate packing-tendency state, which is placed near the commonly discussed $1/3 < p < 1/2$ region for ease of qualitative comparison. The normalized local packing proxy was calculated as:

$$p_{\text{local}} = 0.4 \times \left[p_{\text{contact}} / p_{\text{contact(P1-base)}} \right] \quad (4)$$

1.5.4 Shape descriptors and aggregate-scale compactness descriptor

To evaluate the morphology of precursor aggregates, the largest precursor core was identified from PPO, TOP and LWOH-C beads using a 1.0 nm cutoff. The shape of the largest precursor core was quantified using the eigenvalues of the gyration tensor. The three principal eigenvalues were denoted as $\lambda1$, $\lambda2$ and $\lambda3$, with $\lambda1 \geq \lambda2 \geq \lambda3$. The axis ratio axis1/axis3 was calculated from the square roots of the largest and smallest eigenvalues and was used to describe the elongation degree of the aggregate:

$$\text{axis1}/\text{axis3} = \sqrt{\lambda1}/\sqrt{\lambda3} \tag{5}$$

The relative shape anisotropy κ^2 was used to quantify the deviation from an isotropic spherical shape. A lower axis1/axis3 and κ^2 indicate a more isotropic or high-curvature aggregate, whereas higher values indicate a more elongated and anisotropic precursor assembly. Additional shape descriptors, including axis2/axis3, asphericity and acylindricity, were also calculated and are shown in Supplementary Fig. 52. In addition, a convex-hull-based aggregate-scale p_{eff} was calculated as an auxiliary compactness descriptor. The template core was defined as PPO+TOP, while the precursor core was defined as PPO+TOP+LWOH-C. The aggregate-scale p_{eff} was calculated as:

$$p_{\mathrm{eff}} = V_{\mathrm{core}}/(A_{\mathrm{hull}}\, R_{\mathrm{core90}}) \tag{6}$$

where V_{core} is the effective core volume, A_{hull} is the convex-hull surface area and R_{core90} is the 90th percentile distance of core beads from the core center. This descriptor reflects the geometric compactness of the aggregate and is simultaneously affected by core volume, surface area and aggregate dimension. Therefore, p_{eff} was used only as a supplementary aggregate-scale compactness descriptor rather than as a direct substitute for the classical molecular packing parameter.

1.5.5 Results and interpretation

The normalized local packing proxy showed a clear composition-dependent trend among the three CG models. As summarized in Supplementary Table 4, p_{local} increased from 0.295 ± 0.019 for P3-

highEtOH to 0.400 ± 0.010 for P1-base and 0.538 ± 0.024 for P2-highTOP. The lower p_{local} value in P3-highEtOH was associated with a higher polar contact contribution, whereas the higher p_{local} value in P2-highTOP was associated with an increased hydrophobic contact contribution. These results indicate that the contact-defined packing-tendency proxy was sensitive to the changes in EtOH and TOP contents.

The morphology descriptors further supported the variation in precursor aggregate organization. P3-highEtOH showed the lowest axis1/axis3 value of 1.217 ± 0.071 and the lowest κ^2 value of 0.0167 ± 0.0092, indicating a more isotropic and high-curvature precursor aggregate. P1-base exhibited intermediate values, with axis1/axis3 = 1.352 ± 0.079 and κ^2 = 0.0284±0.0098. P2-highTOP showed the highest anisotropy, with axis1/axis3 = 1.400 ± 0.073 and κ^2 = 0.0447 ± 0.0174, suggesting a tendency toward more elongated and lower-curvature precursor assemblies.

The representative CG snapshots shown in Supplementary Fig. 51 are consistent with these quantitative descriptors. P3-highEtOH exhibited a more compact and high-curvature local aggregate morphology, P1-base showed an intermediate organization, and P2-highTOP displayed a more elongated precursor aggregate. These results support the use of the contact-derived p_{local} and shape descriptors to compare precursor-stage organization under different composition-variation conditions.

The supplementary quantitative analyses shown in Supplementary Fig. 52 further substantiate the contact-derived packing interpretation and the aggregate morphology descriptors. The hydrophobic and polar contact proxies shown in Supplementary Fig. 52a decompose the numerator and denominator of p_{local}, confirming that the increase in p_{local} for P2-highTOP originates from enhanced hydrophobic contacts, whereas P3-highEtOH is associated with stronger polar contact contributions. The convex-hull-based compactness descriptors shown in Supplementary Fig. 52b provide an

aggregate-scale geometric cross-check of the local packing tendency for both template and precursor cores. The additional shape descriptors shown in Supplementary Fig. 52c-e, including axis2/axis3, asphericity and acylindricity, further support the morphology evolution inferred from axis1/axis3 and κ^2.

It should be noted that the CG simulations describe precursor-stage soft-template organization rather than the final carbonized mesoporous structure. Processes such as M/F condensation, curing, carbonization, template removal and framework shrinkage were not explicitly simulated. Therefore, the CG results are interpreted as comparative mechanistic evidence for precursor-stage organization, rather than as a direct reproduction of the final mesoporous carbon morphology.

1.6 Actual-ratio long-box TOP-rich/water interfacial simulations for validating TOP-induced interfacial localization and anisotropic organization

1.6.1 Model construction and simulation protocol

To further validate the proposed transition from homogeneous hydrated association to TOP-rich/water interfacial organization, actual-ratio long-box interfacial CG models were constructed using the same molecular compositions as the corresponding P-series simulations. The low-TOP model, denoted as $Ea1_{TOP40}$, contained 16 F127M molecules, 40 TOP molecules, 9000 ETHX beads, 36538 water beads and 120 LWOH molecules. The high-TOP model, denoted as $Ea2_{TOP120}$, contained 16 F127M molecules, 120 TOP molecules, 9000 ETHX beads, 36538 water beads and 120 LWOH molecules. Thus, the two models differed only in the TOP content, allowing a controlled comparison of TOP-content-dependent interfacial organization.

The simulation box was constructed with a reduced interfacial area and elongated z dimension of $10\times10\times60$ nm to increase the effective TOP surface density and stabilize a TOP-rich slab. The

F127/LWOH components were initially placed near the TOP-rich/water interfacial region to examine interfacial adsorption, segmental partitioning and anisotropic organization. After energy minimization, NVT equilibration and z-only semi-isotropic NPT equilibration, 20 ns production simulations were performed. The x and y dimensions were kept fixed to maintain the interfacial area, while the z dimension was allowed to relax during NPT and production simulations. The last 2 ns of each trajectory were used for quantitative analysis.

1.6.2 Analysis descriptors

TOP-rich slab stability was evaluated by calculating the number of TOP clusters and the fraction of TOP beads in the largest TOP cluster using a 0.8 nm cutoff. Segment-resolved contact analysis was performed between PEO, PPO or LWOH and TOP, water or ETHX using the same cutoff. The normalized contact density was obtained by dividing the total number of contacts by the number of corresponding beads. z-directional normalized density profiles were calculated over the final 2 ns to examine the spatial distributions of TOP, PEO, PPO and LWOH along the interface normal. Shape and orientation descriptors were calculated from the eigenvalues and eigenvectors of the gyration tensor.

The axis1/axis3 ratio and relative shape anisotropy κ^2 were used to describe elongation and shape anisotropy, respectively. The orientation parameter S was calculated relative to the z-axis, which corresponds to the normal direction of the TOP-rich/water interface. S = -0.5 represents ideal in-plane alignment, S = 0 represents random orientation and S = 1 represents perpendicular alignment.

1.6.3 TOP-slab stability and segment-selective interfacial anchoring

The quantitative descriptors summarized in Supplementary Table 5 first confirm that both actual-ratio long-box models maintained a stable TOP-rich slab over the final 2 ns. In both $Ea1_{TOP40}$ and $Ea2_{TOP120}$, the number of TOP clusters remained 1 and the largest TOP-cluster fraction remained 1.0.

Therefore, the difference between the two systems mainly reflects the TOP-content-dependent interfacial coupling strength rather than the presence or absence of a TOP-rich slab.

The normalized contact densities summarized in Supplementary Table 5 and plotted in Supplementary Fig. 53 show that increasing the TOP content from 40 to 120 molecules substantially enhanced the segment-selective anchoring of F127. The normalized PPO-TOP contact density increased from 2.27 to 8.48 contacts per bead, whereas the normalized PEO-TOP contact density increased from 0.51 to 0.97 contacts per bead. Therefore, PPO-TOP contacts were much stronger than PEO-TOP contacts in both models, confirming that TOP preferentially anchors the PPO hydrophobic segment rather than uniformly interacting with the whole F127 chain. Meanwhile, the normalized PPO-water contact density decreased sharply from 1.33 to 0.081 contacts per bead, indicating that TOP enrichment effectively excludes PPO from the aqueous region and drives PPO toward the TOP-rich slab.

1.6.4 Spatial distribution and anisotropic interfacial organization

The z-directional normalized density profiles shown in Supplementary Fig. 53e,f further confirm the TOP-content-dependent interfacial localization. In $Ea1_{TOP40}$, the mean z positions of PEO and PPO were 34.69 and 34.71 nm, respectively, which were below the TOP-rich slab centered at 35.63 nm. In $Ea2_{TOP120}$, the corresponding mean z positions shifted to 36.17 and 36.15 nm, respectively, close to the TOP-rich slab centered at 35.96 nm.

The shape and orientation descriptors summarized in Supplementary Table 5 and shown in Supplementary Fig. 53i,j further support the TOP-induced anisotropic interfacial organization. The aggregate axis1/axis3 ratio increased from 1.96 in $Ea1_{TOP40}$ to 2.30 in $Ea2_{TOP120}$, while the relative shape anisotropy kappa2 increased from 0.141 to 0.272. More importantly, the aggregate orientation

parameter shifted from -0.275 to -0.476, approaching the theoretical value of -0.5 for ideal in-plane alignment. A similar trend was observed for F127 chains: the F127 orientation parameter shifted from -0.071 in $Ea1_{TOP40}$ to -0.441 in $Ea2_{TOP120}$. These results demonstrate that TOP enrichment enhances PPO-TOP anchoring and induces in-plane anisotropic growth of the aggregate at TOP-rich/water interface.

It should be noted that the long-box interfacial models were designed to validate interfacial adsorption and anisotropic organization under actual formulation ratios. Because the F127/LWOH components were initially placed near the TOP-rich/water interfacial region, these simulations are interpreted as direct evidence for interfacial coupling, segmental partitioning and anisotropic organization, rather than as a measurement of the long-range migration kinetics from a far-away bulk aqueous phase.

The correspondence between the conclusions, Supplementary Table 5 and Supplementary Fig. 53 is therefore as follows: TOP-cluster descriptors support slab stability and contact-density descriptors support PPO-selective anchoring ; z-density profiles support spatial localization; and shape/orientation descriptors support in-plane anisotropic organization.

1.7 Functional role of LWOH in TOP-rich/water interfacial organization

To clarify the functional role of LW in precursor-stage interfacial organization, an LWOH-removal control simulation was performed by removing LWOH from the equilibrated actual-ratio TOP120 long-box interfacial configuration while keeping F127M, TOP, ETHX and water unchanged (Supplementary Fig. 54).

After LWOH removal, the TOP-rich slab remained stable, with one TOP cluster and a largest TOP-cluster fraction of 1.0. The F127/TOP contacts were also retained. The normalized PPO–TOP contact

density was 9.173 contacts per bead after LWOH removal, comparable to that in the LWOH-containing TOP120 model, 8.484 contacts per bead. The normalized PEO−TOP contact density was 0.803 contacts per bead after LWOH removal, slightly lower than that in the LWOH-containing model, 0.974 contacts per bead.

In the LWOH-containing TOP120 model, LWOH simultaneously contacted the TOP-rich and water-rich environments, with normalized LWOH–TOP and LWOH–water contact densities of 3.477 and 3.314 contacts per bead, respectively. This simultaneous association indicates that LW is primarily located in the TOP-rich/water interfacial transition region rather than being fully partitioned into either phase. Removing LWOH also increased the water exposure of F127. The normalized PPO−water contact density increased from 0.081 contacts per bead in the LWOH-containing model to 0.805 contacts per bead after LWOH removal, while the PEO–water contact density increased from 3.309 to 4.241 contacts per bead. These results suggest that LW contributes to stabilizing the local interfacial environment and suppressing the re-exposure of the PPO-rich domain to water, although TOP–PPO hydrophobic anchoring itself can still be maintained.

The shape and orientation descriptors further reveal that LW influences the mode of interfacial organization. The F127 axis1/axis3 ratio remained relatively high after LWOH removal, 3.717, compared with 4.076 in the LWOH-containing model, indicating that the assembly remained anisotropic. However, the orientation parameter changed markedly. In the LWOH-containing model, the F127 orientation parameter was -0.441, close to the value expected for in-plane interfacial alignment. After LWOH removal, this value shifted to 0.918, indicating a strong tendency toward alignment along the interface normal. This result shows that LW is important not only for precursor incorporation but also for maintaining the in-plane interfacial organization of the soft-template

assembly.

Taken together, these results demonstrate that LW can serve as a carbonizable amphiphilic interfacial precursor that bridges the TOP/PPO-rich domain and the PEO/EtOH/water-rich polar environment, stabilizes the local interfacial environment and promotes the in-plane interfacial organization of F127.

2 Supplementary Figures and Tables

Supplementary Table 1. Synthesis parameters of the control experiments

Sample	TOP amount (mL)	Ethanol (mL)	Synthesized temperature (°C)	F127 amount (g)	HCl amount (mL)	Morphology and Structure
MCNs-0	0	10	150	1.1	10	Dodecahedron
MCNs-1	0.05	10	150	1.1	10	Ordered nanofibers
MCNs-2	0.1	10	150	1.1	10	Ordered nanofibers
MCNs-3	0.2	10	150	1.1	10	Hollow nanofibers
MCNs-4	0.3	10	150	1.1	10	Bowl-like nanospheres
MCNs-5	0.2	10	150	0.5+0.6 F108	10	Worm-like nanoparticles
MCNs-6	0.2	10	150	0.5+0.6 P123	10	Vesicle-like nanospheres
MCNs-7	0.2	10	150	0.5+0.6 P84	10	Hollow spheres
MCNs-8	0.05	5	150	1.1	10	Ordered nanofibers
MCNs-9	0.05	20	150	1.1	10	Mesoporous nanospheres
MCNs-10	0.05	25	150	1.1	10	Golf ball-like nanospheres
MCNs-11	0.1	5	150	1.1	10	Ordered nanofibers
MCNs-12	0.1	20	150	1.1	10	Mesoporous nanospheres
MCNs-13	0.1	25	150	1.1	10	Raspberry-like nanospheres
MCNs-14	0.2	5	150	1.1	10	Irregular
MCNs-15	0.2	15	150	1.1	10	Curved nanoribbons
MCNs-16	0.2	20	150	1.1	10	Ordered nanorods
MCNs-17	0.2	25	150	1.1	10	Multichambered nanospheres
MCNs-18	0.2	30	150	1.1	10	Smooth nanospheres
MCNs-19	0.2	40	150	1.1	10	Solid nanospheres
MCNs-20	0.3	5	150	1.1	10	Amorphous
MCNs-21	0.3	20	150	1.1	10	Multi-chamber nanospheres
MCNs-22	0.3	25	150	1.1	10	Branched nanospheres
MCNs-23	0.4	20	150	1.1	10	Dendritic nanospheres
MCNs-24	0.4	25	150	1.1	10	Gear-like nanospheres
MCNs-25	0.05	10	130	1.1	10	Agglomerated particles
MCNs-26	0.05	10	180	1.1	10	Ordered nanofibers
MCNs-27	0.2	10	130	1.1	10	Agglomerated particles
MCNs-28	0.2	10	180	1.1	10	Ordered nanofibers
MCNs-29	0.3	10	130	1.1	10	Nanosheets
MCNs-30	0.3	10	180	1.1	10	Blocky nanoparticles
MCNs-31	0.05	25	130	1.1	10	Mesoporous nanospheres

MCNs-32	0.05	25	180	1.1	10	Mesoporous nanospheres
MCNs-33	0.2	25	130	1.1	10	Mesoporous nanospheres
MCNs-34	0.2	25	180	1.1	10	Mesoporous nanospheres
MCNs-35	0.3	25	130	1.1	10	Plum blossom-like nanospheres
MCNs-36	0.3	25	180	1.1	10	Gear-like nanospheres
MCNs-37	0.2	10	150	0	10	Solid nanospheres
MCNs-38	0.2	25	150	0	10	Solid nanospheres
MCNs-39	0.05	10	150	0.5	10	Amorphous
MCNs-40	0.05	10	150	0.8	10	Ordered nanofibers
MCNs-41	0.05	10	150	1.5	10	Ordered nanofibers
MCNs-42	0.1	10	150	0.5	10	Ordered nanoparticles
MCNs-43	0.1	10	150	0.8	10	Ordered nanofibers
MCNs-44	0.1	10	150	1.5	10	Ordered nanofibers
MCNs-45	0.2	10	150	0.5	10	Gyroid-like
MCNs-46	0.2	10	150	0.8	10	nanofibers
MCNs-47	0.2	10	150	1.5	10	Hollow nanofibers
MCNs-48	0.3	10	150	0.5	10	Concentric layered nanospheres
MCNs-49	0.3	10	150	0.8	10	Flake-like nanospheres
MCNs-50	0.3	10	150	1.5	10	Bowl-like nanospheres
MCNs-51	0.05	25	150	0.5	10	Ordered nanofibers
MCNs-52	0.05	25	150	0.8	10	Mesoporous nanospheres
MCNs-53	0.05	25	150	1.5	10	Mesoporous nanospheres
MCNs-54	0.1	25	150	0.5	10	Ordered nanorods
MCNs-55	0.1	25	150	0.8	10	Worm-like nanoparticles
MCNs-56	0.1	25	150	1.5	10	Mesoporous nanospheres
MCNs-57	0.2	25	150	0.5	10	Paperclip-like nanorods
MCNs-58	0.2	25	150	0.8	10	Small nanoparticles
MCNs-59	0.2	25	150	1.5	10	Pinecone-like nanoparticles
MCNs-60	0.3	25	150	0.5	10	Ordered nanoparticles
MCNs-61	0.3	25	150	0.8	10	Honeycomb-like nanoparticles
MCNs-62	0.3	25	150	1.5	10	Adhered nanospheres
MCNs-63	0.2	10	150	1.1	8	Helical hollow nanorods
MCNs-64	0.2	10	150	1.1	9	Ordered nanofibers
MCNs-65	0.2	10	150	1.1	11	Twisted nanofibers
MCNs-66	0.2	10	150	1.1	12	Amorphous
MCNs-67	0.2	25	150	1.1	8	Mesoporous nanospheres
MCNs-68	0.2	25	150	1.1	9	Mesoporous nanospheres
MCNs-69	0.2	25	150	1.1	11	Mesoporous nanospheres
MCNs-70	0.2	25	150	1.1	12	Mesoporous nanospheres

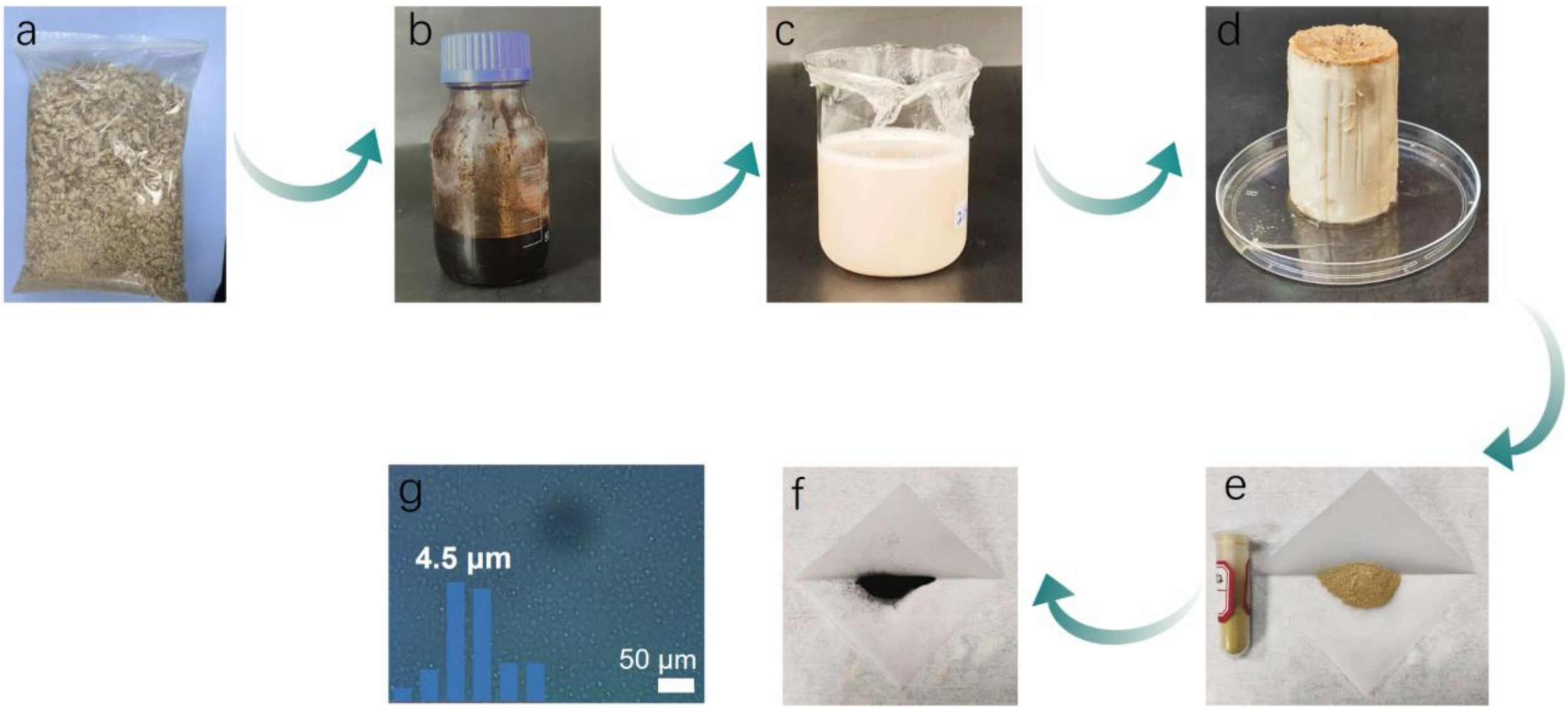

Supplementary Fig. 1. Photographs of the formation process: (a) The biomass larch sawdust. (b) Liquefied wood. (c) Mixture after stirring at 500 rpm. (d) Resin polymers after hydrothermal reaction. (e) Dried polymer powder. (f) Hollow carbon fibers. (g) Optical micrograph of the mixture solution

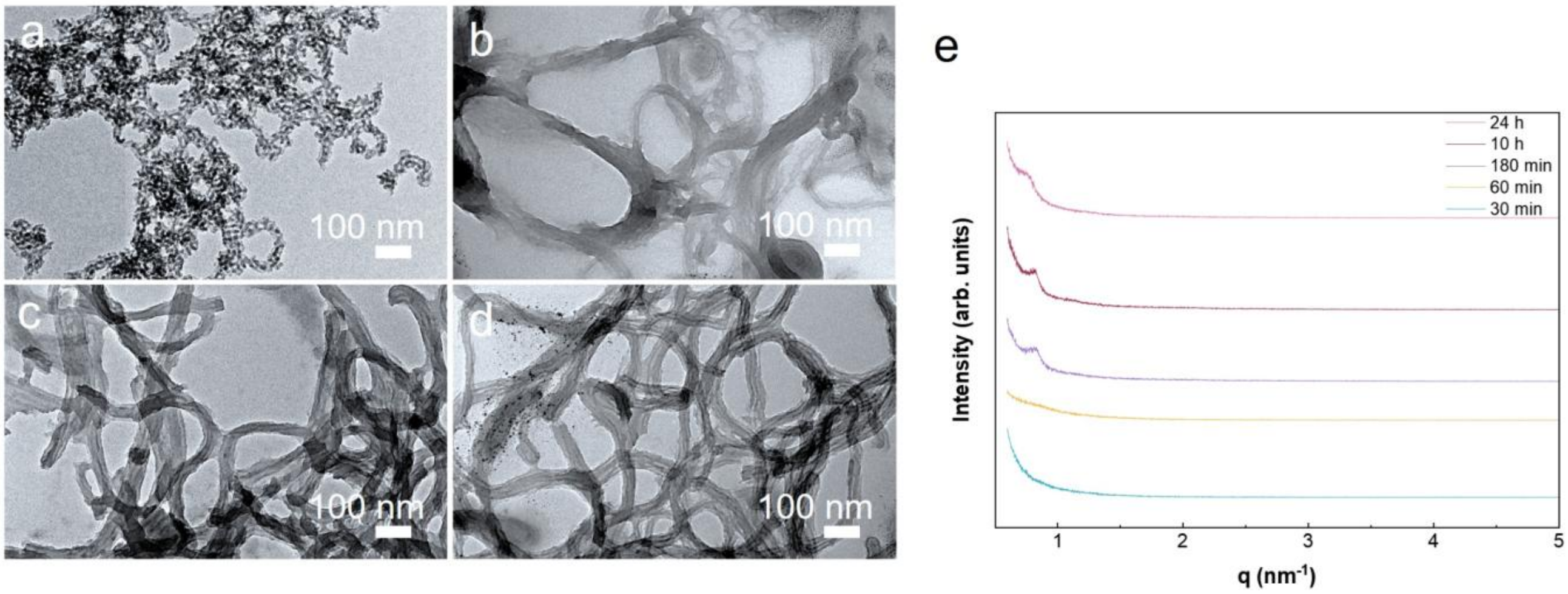

Supplementary Fig. 2. TEM image of polymeric precursor of MCNs-3 at different hydrothermal times. (a) 30 min, (b) 60 min, (c) 180 min, (d) 24 h, (e) SAXS patterns of the corresponding products collected after 30 min, 60 min, 180 min, 10 h and 24 h of hydrothermal treatment. The absence of newly emerged sharp higher-order peaks upon prolonged hydrothermal treatment indicates that extended polymerization does not induce an additional independent ordered mesophase transition.

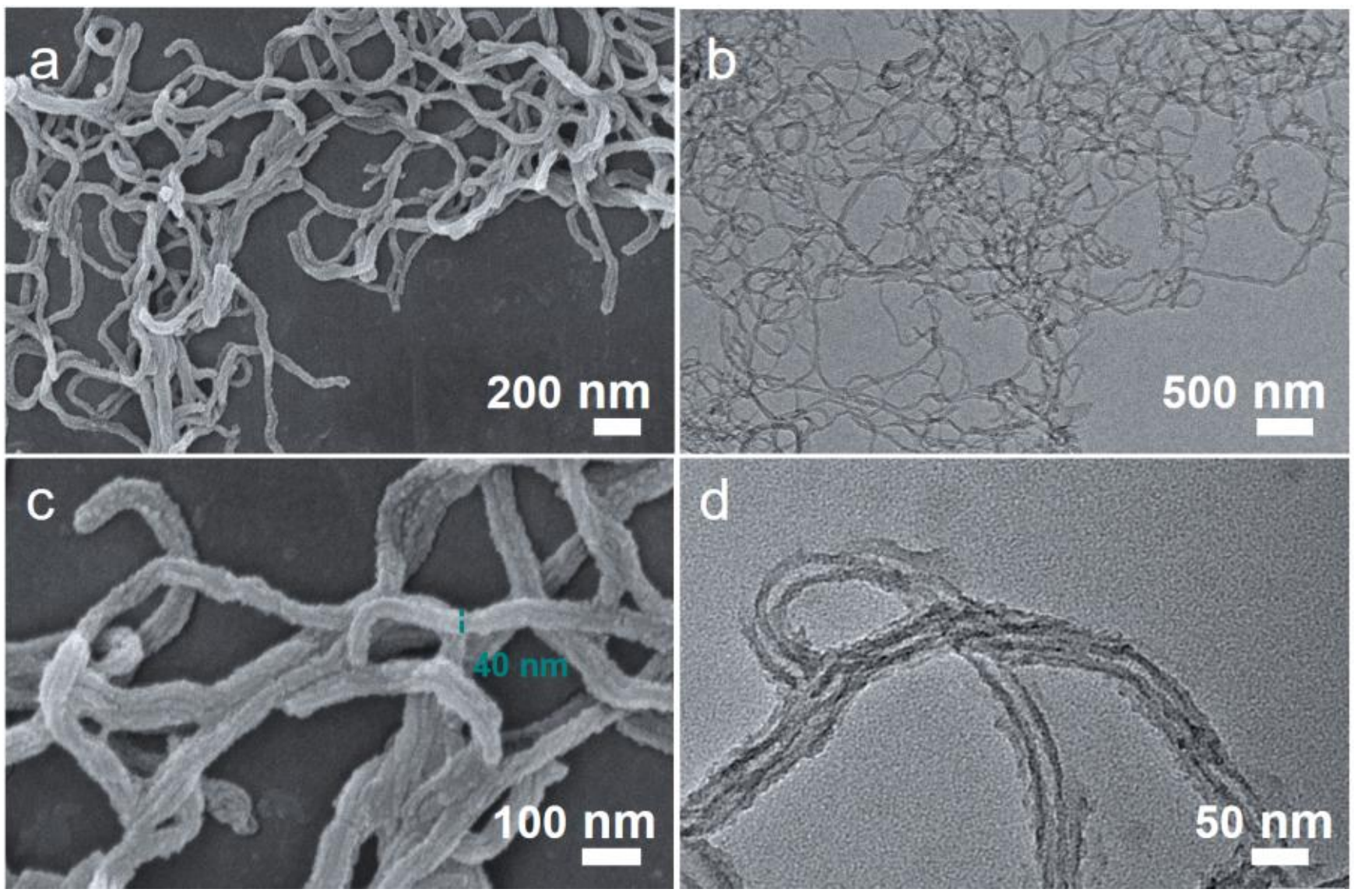

Supplementary Fig. 3. Structural characterization of the as-made resin polymers. (a, c) SEM images and (b, d) TEM images of the as-made resin polymers prepared by the emulsion-mediated self-assembly approach. The as-made resin polymers were prepared by using F127 as the soft-template, TOP as the swelling agent and interface mediation agent, and LW and melamine as the carbon source and nitrogen source.

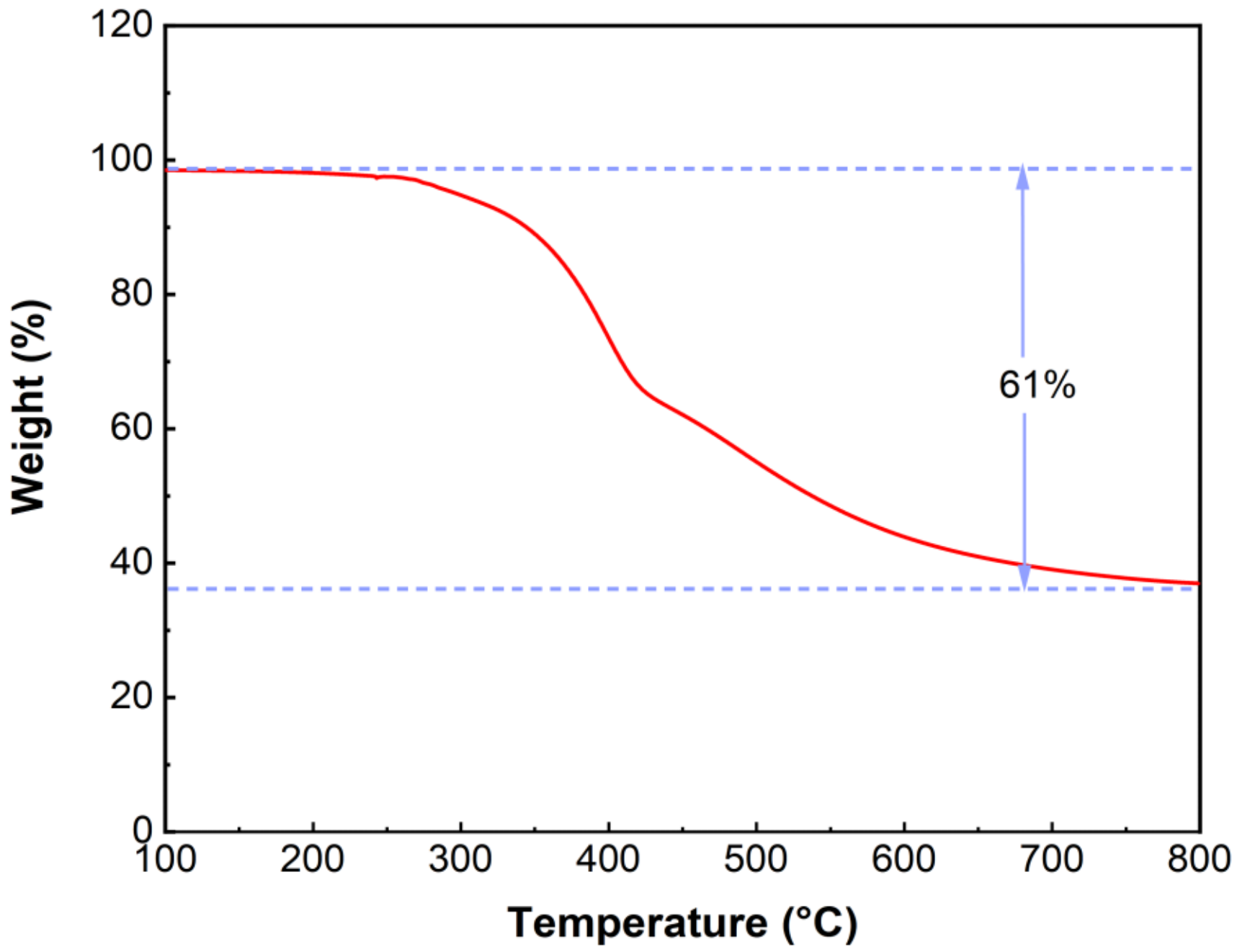

Supplementary Fig. 4. TGA curve of polymeric nanofibers. The polymeric hollow nanofibers were prepared by the emulsion-mediated self-assembly strategy. The result implies that the F127 can be totally removed before 400 °C and the carbon yield is ~39% at 800 °C.

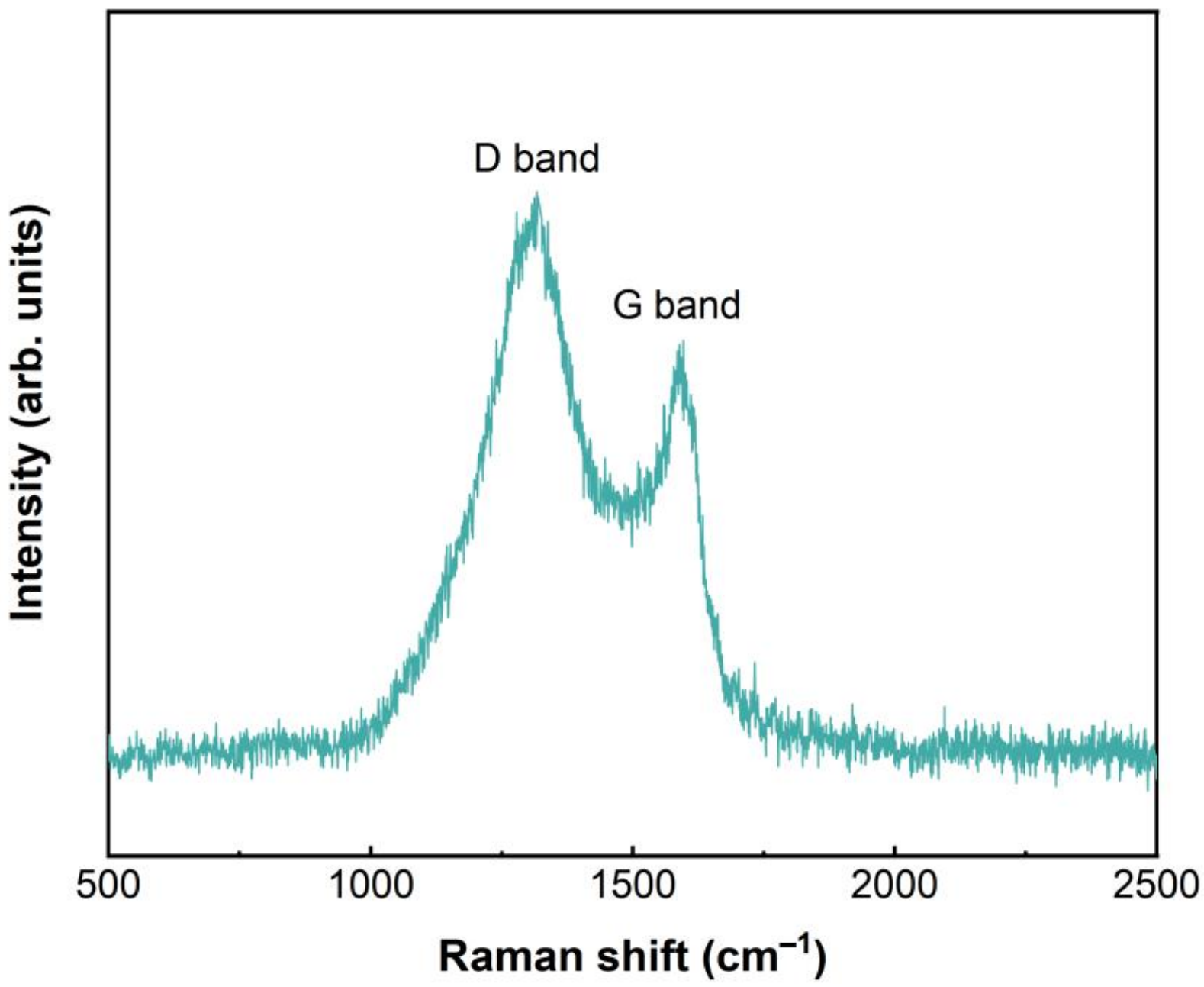

Supplementary Fig. 5. Raman spectrum of the hollow carbon nanofibers. Raman spectrum of the carbon hollow nanofibers by the emulsion-mediated self-assembly strategy. The spectrum shows a typical D-band (defect-induced) at 1320 cm^{-1} and G-band (crystalline graphite) at 1593 cm^{-1}.

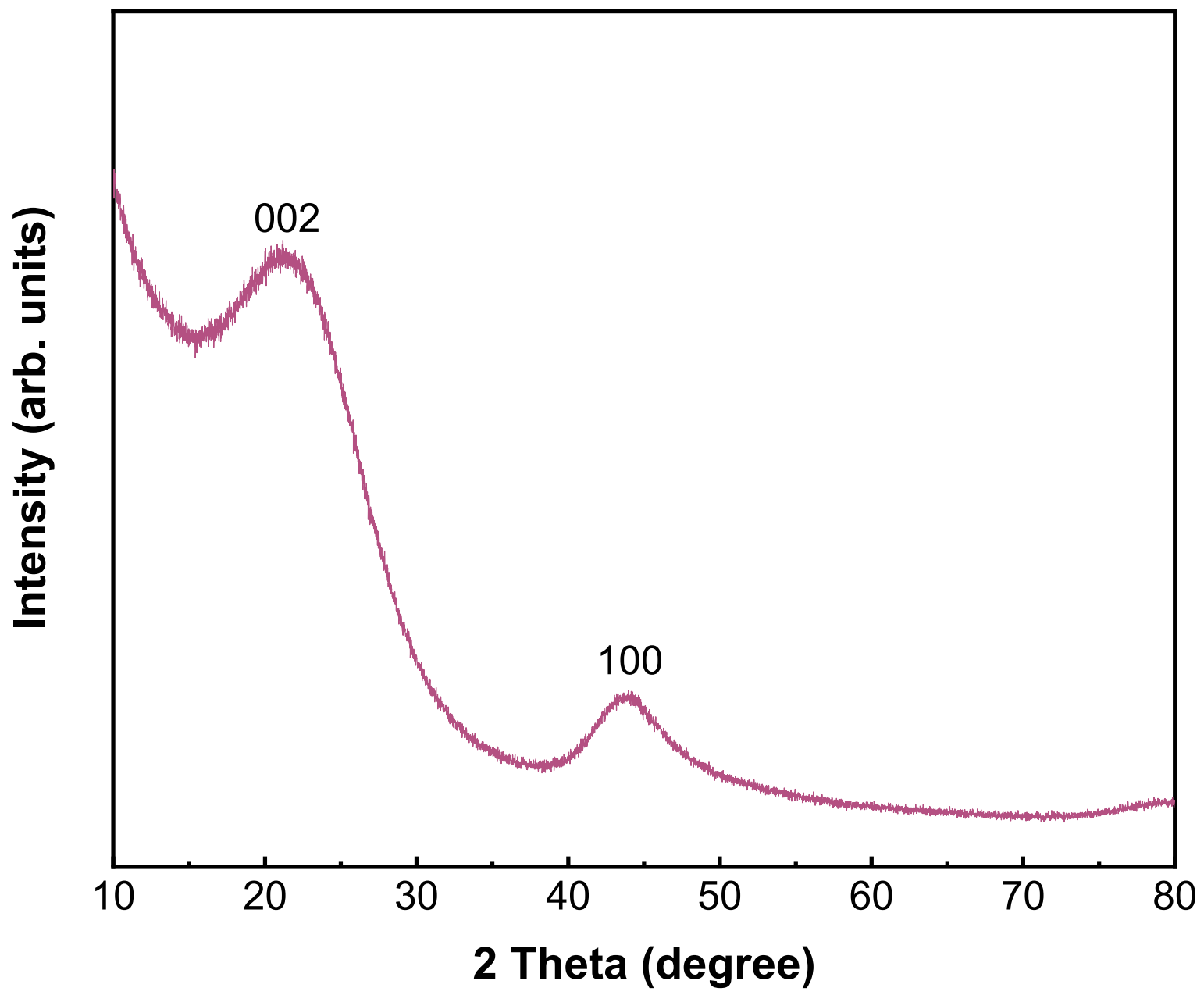

Supplementary Fig. 6. XRD pattern of the hollow carbon nanofibers. The XRD pattern of the hollow carbon nanofibers prepared by the emulsion-mediated self-assembly strategy. Two diffraction peaks at 21.3° and 43.8° are ascribed to (002) and (100) planes, respectively.

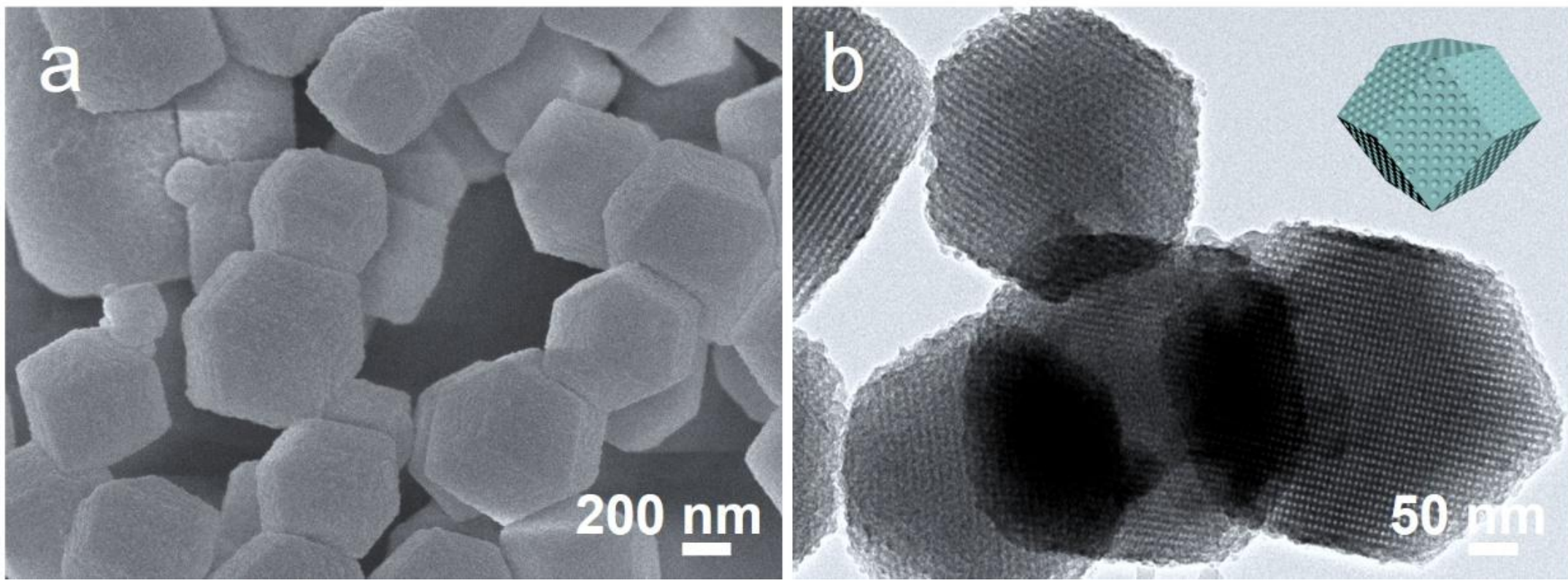

Supplementary Fig. 7. Morphology and mesostructure of MCNs-0 prepared without TOP. (a) SEM image of MCNs-0 showing the dodecahedral morphology. (b) TEM image showing the ordered mesostructure of MCNs-0; inset, corresponding 3D structural model.

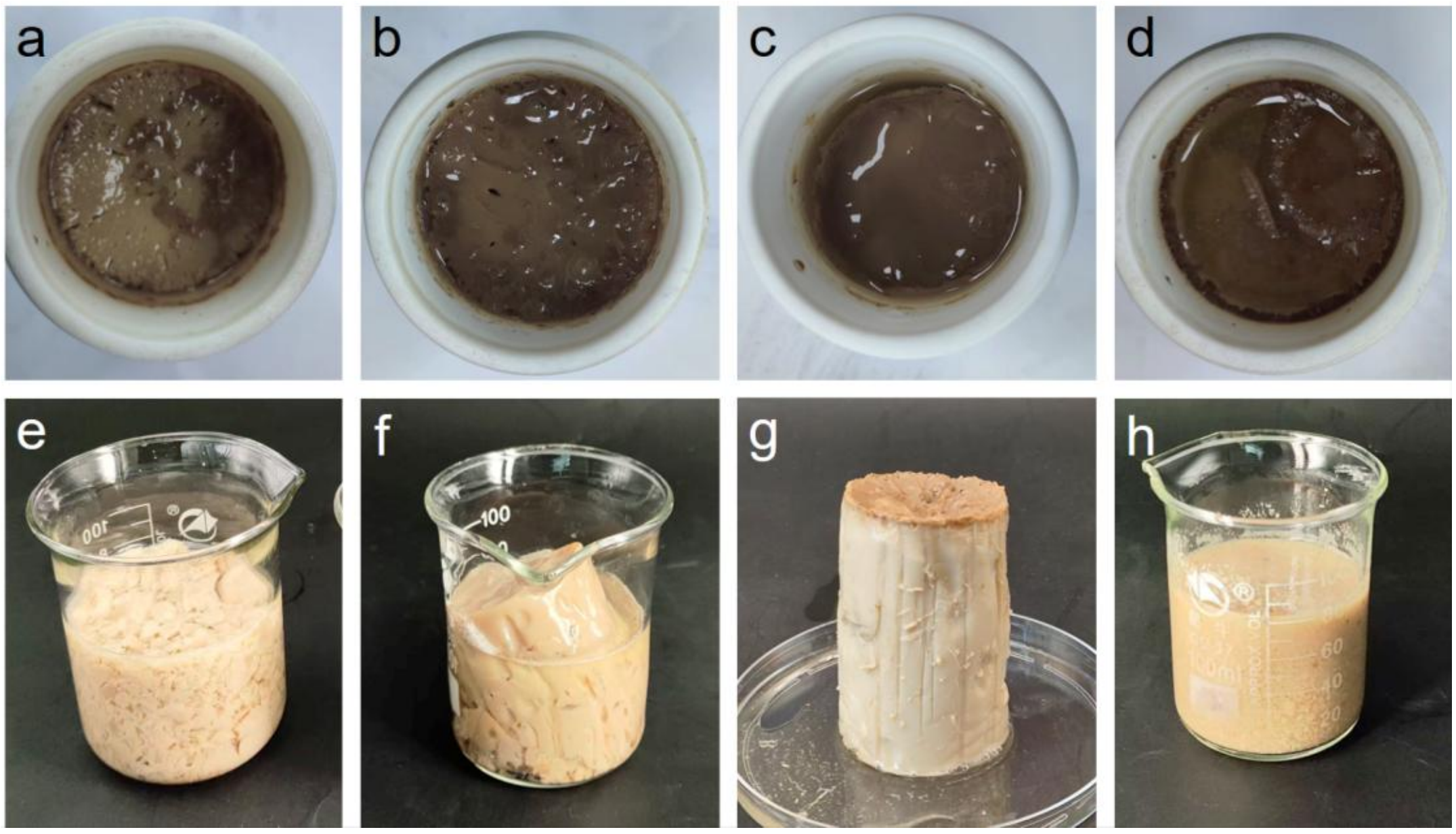

Supplementary Fig. 8. Photograph of the product inside the reactor after hydrothermal treatment with different TOP containing. (a, e) 0.05 mL, (b, f) 0.1 mL, (c, g) 0.2 mL and (d, h) 0.3 mL. The results indicate that the polymeric precursor inside the reactor from gelatinous, gradually formed self-supporting columns with increasing TOP, yet excessive TOP led to the formation of suspended particles.

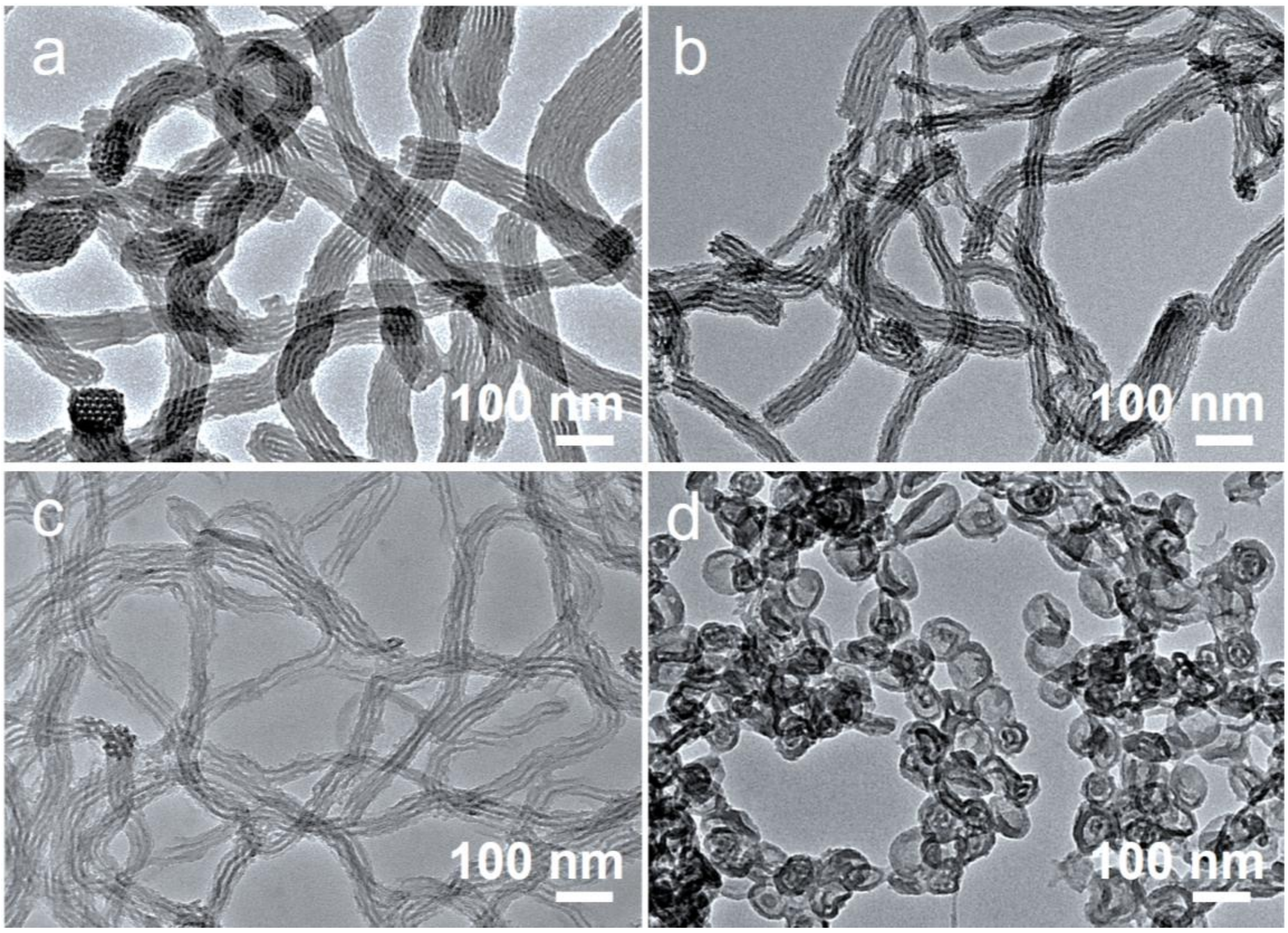

Supplementary Fig. 9. TEM images of the as-prepared MCNs. The TEM images of the MCNs-x (x=1, 2, 3, 4**)** were prepared with different amounts of TOP by the emulsion-mediated self-assembly strategy. (a) 0.05 mL, (b) 0.1 mL, (c) 0.2 mL and (d) 0.3 mL. The results indicate that as the TOP content increases, the number of mesoporous channels in MCNs decreases from tens to several, and to one mesoporous channel. Finally, the structure transforms into nanobowls.

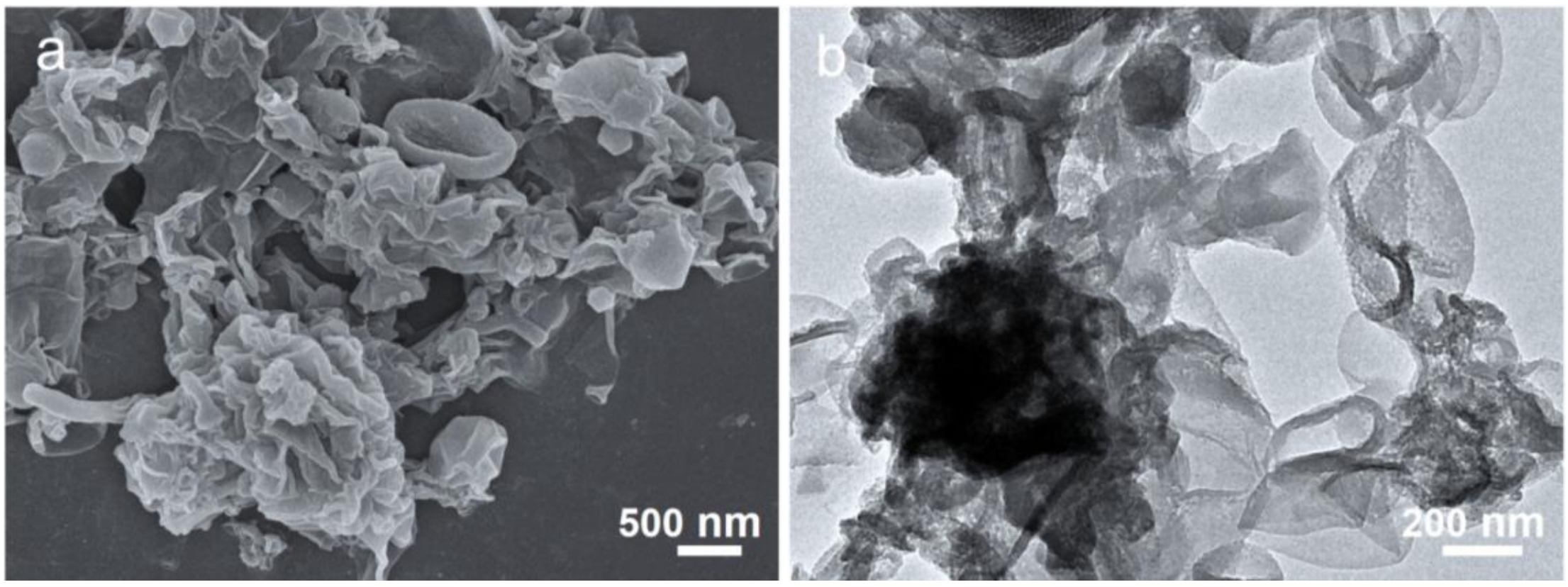

Supplementary Fig. 10. SEM and TEM images of the product prepared with 0.4 mL of TOP. (a) SEM image and (b) TEM image of the product prepared with 0.4 mL TOP, showing the formation of irregular nanosheet-like structures.

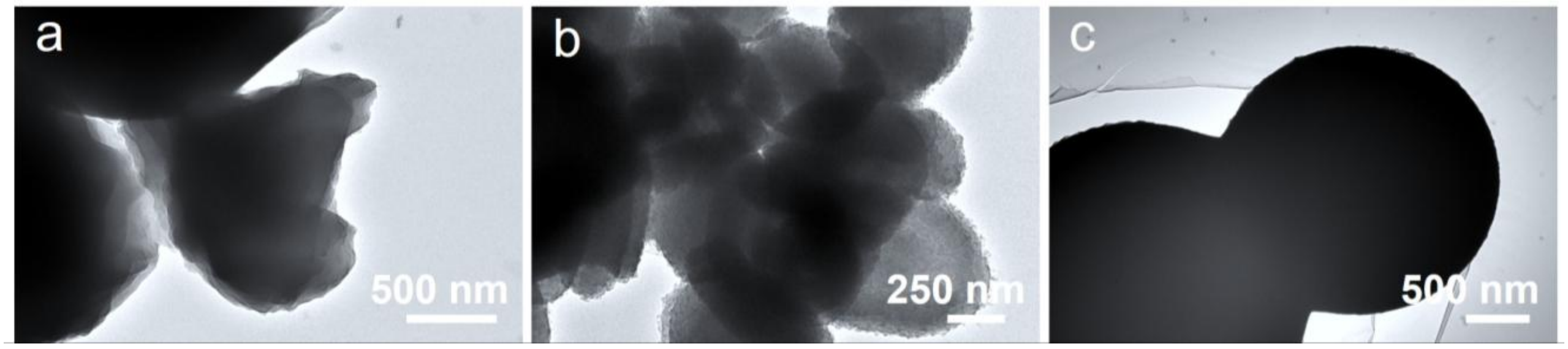

Supplementary Fig. 11. Structural characterization of the as-prepared carbons with different alcohols added by emulsion-mediated assembly approach. (a) TEM images of the carbon materials prepared with methanol as the solvent. (b) TEM images of the carbon materials prepared with isopropanol as the solvent. (c) TEM images of the carbon materials prepared with N, N-dimethylformamide as the solvent. The process was performed similarly to the Supplementary Methods section except the ethanol was replaced. The results show that no regular and ordered structure was observed when the cosolvent was replaced from ethanol to methanol, to isopropanol and to N, N-dimethylformamide.

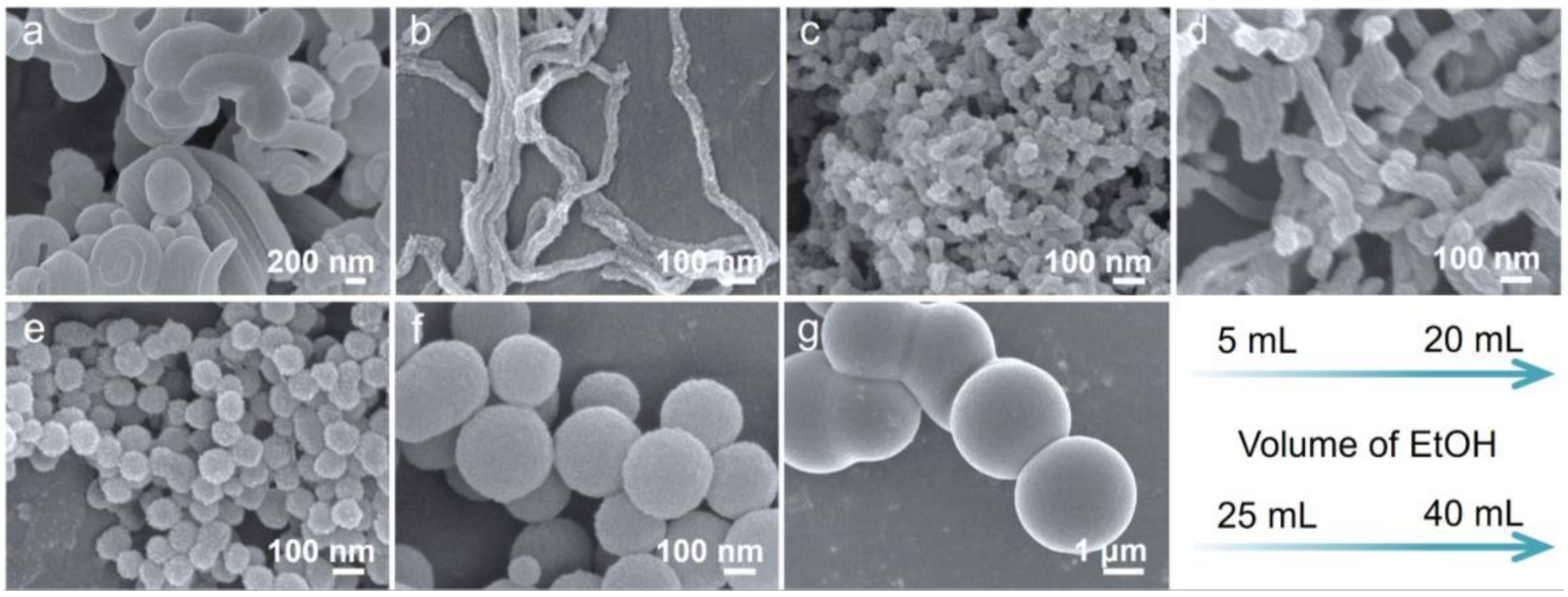

Supplementary Fig. 12. SEM images of the MCN-x (x=14, 3, 15, 16, 17, 18, 19) prepared by the emulsion-mediated assembly approach in different volumes of ethanol system with 0.2 mL TOP. (a) 5 mL, (b) 10 mL, (c) 15 mL, (d) 20 mL, (e) 25 mL, (f) 30 mL, (g) 40 mL. The results indicate that the morphology of the products varied from nanofibers to twisted nanoparticles, to short nanofibers and finally to nanospheres as the ethanol volumes changed from 10 mL to 40 mL.

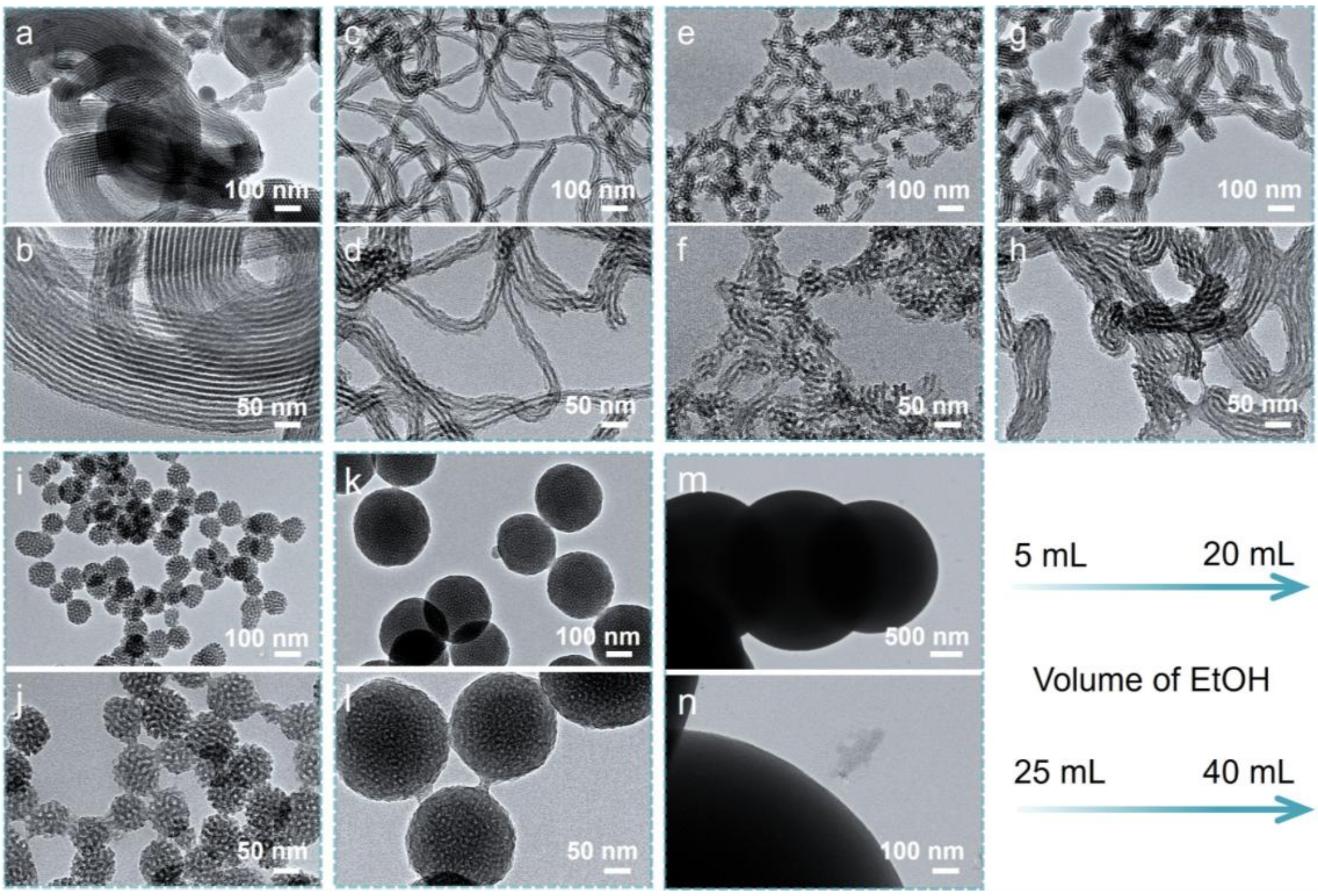

Supplementary Fig. 13. TEM images of MCNs-x (x=14, 3, 15, 16, 17, 18, 19) prepared by the emulsion-mediated assembly approach in different volumes of ethanol system with 0.2 mL TOP. (a, b) 5 mL, (c, d) 10 mL, (e, f) 15 mL, (g, h) 20 mL, (i, j) 25 mL, (k, l) 30 mL, (m, n) 40 mL. The results indicate that when the ethanol content was below 20 ml, strip-like mesoporous channels could form, while spherical mesopores appeared when the ethanol content was excessive.

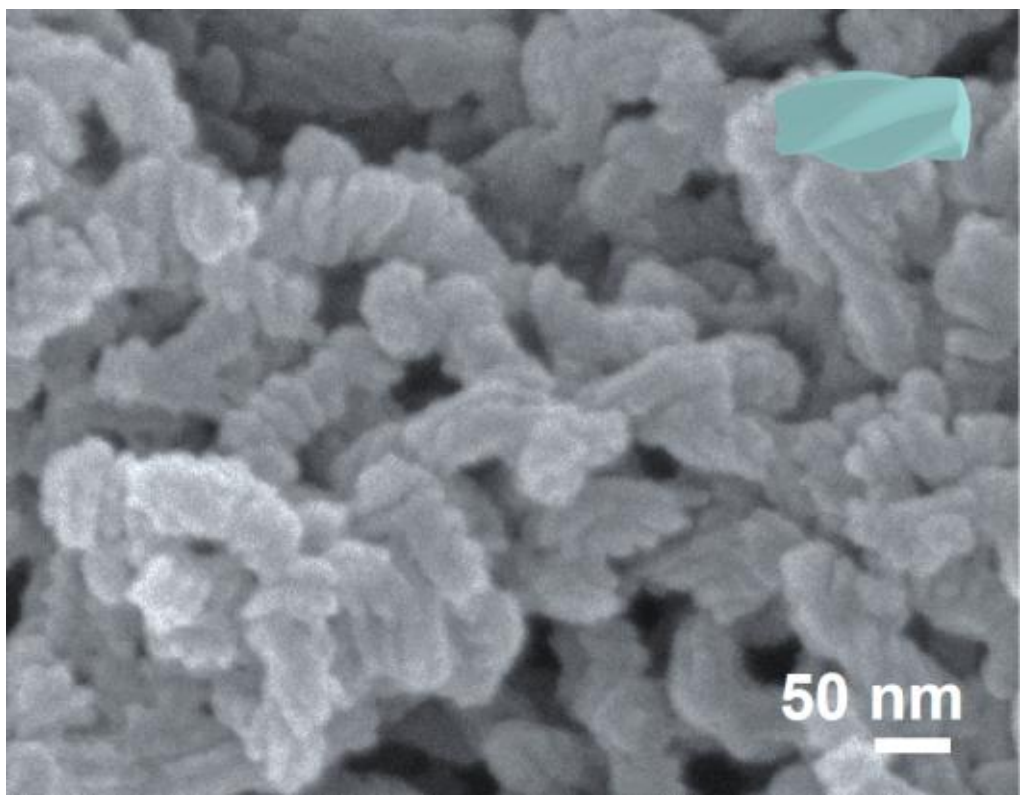

Supplementary Fig. 14. Enlarged SEM image of MCNs-15 prepared by the emulsion-mediated assembly approach with 15 mL of ethanol. Inset is the structural model. The result indicates that MCNs-15 exhibited an interesting braided-like morphology.

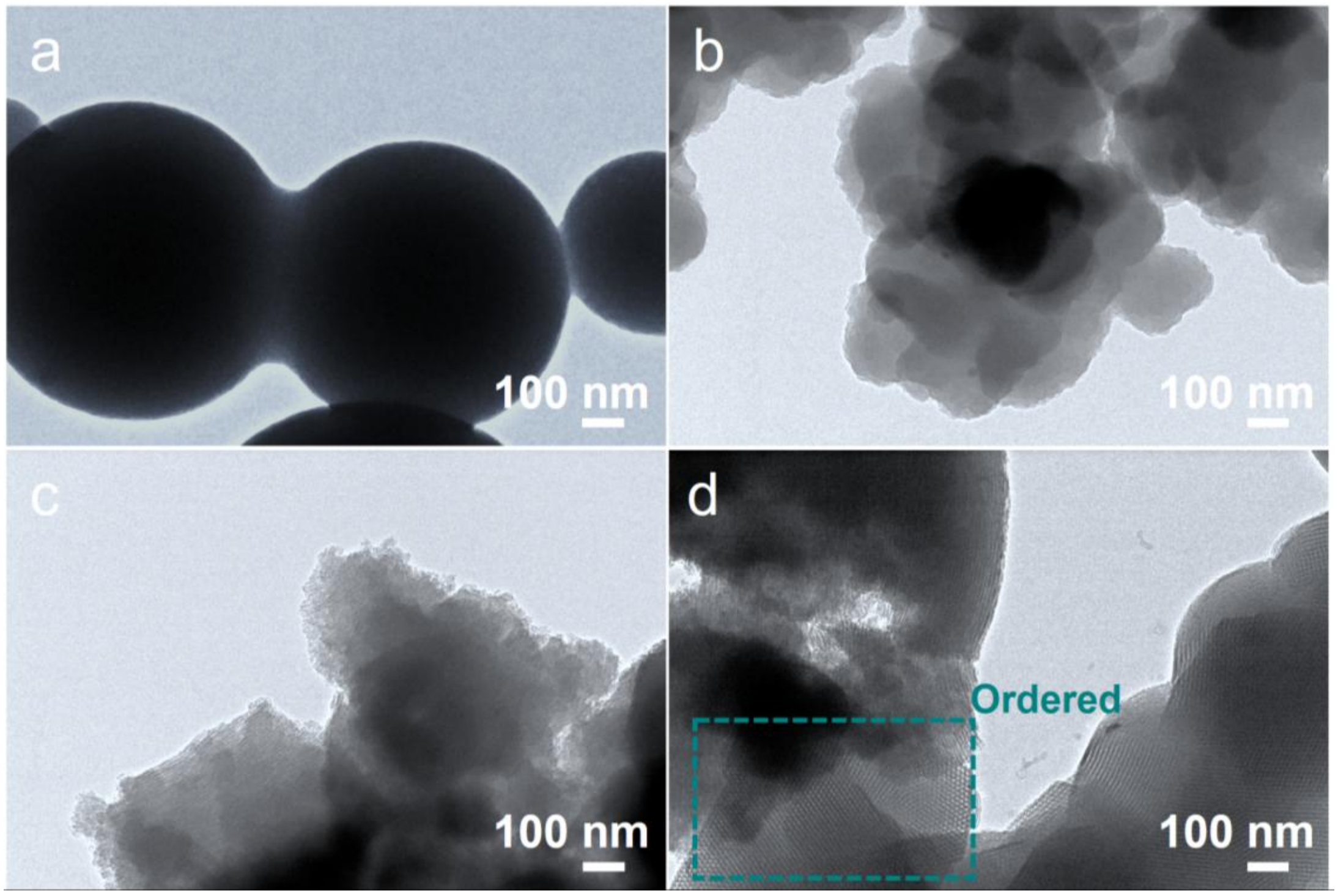

Supplementary Fig. 15. TEM images of MCNs prepared by the emulsion-mediated assembly approach with different additions of TOP in an ethanol-free system. (a) 0 mL, (b) 0.1 mL, (c) 0.2 mL, (d) 0.3 mL. These results indicate that in the absence of ethanol, it is difficult to obtain well-defined nanoparticles merely by adjusting TOP content, while TOP could promote the formation of ordered structures within a certain range.

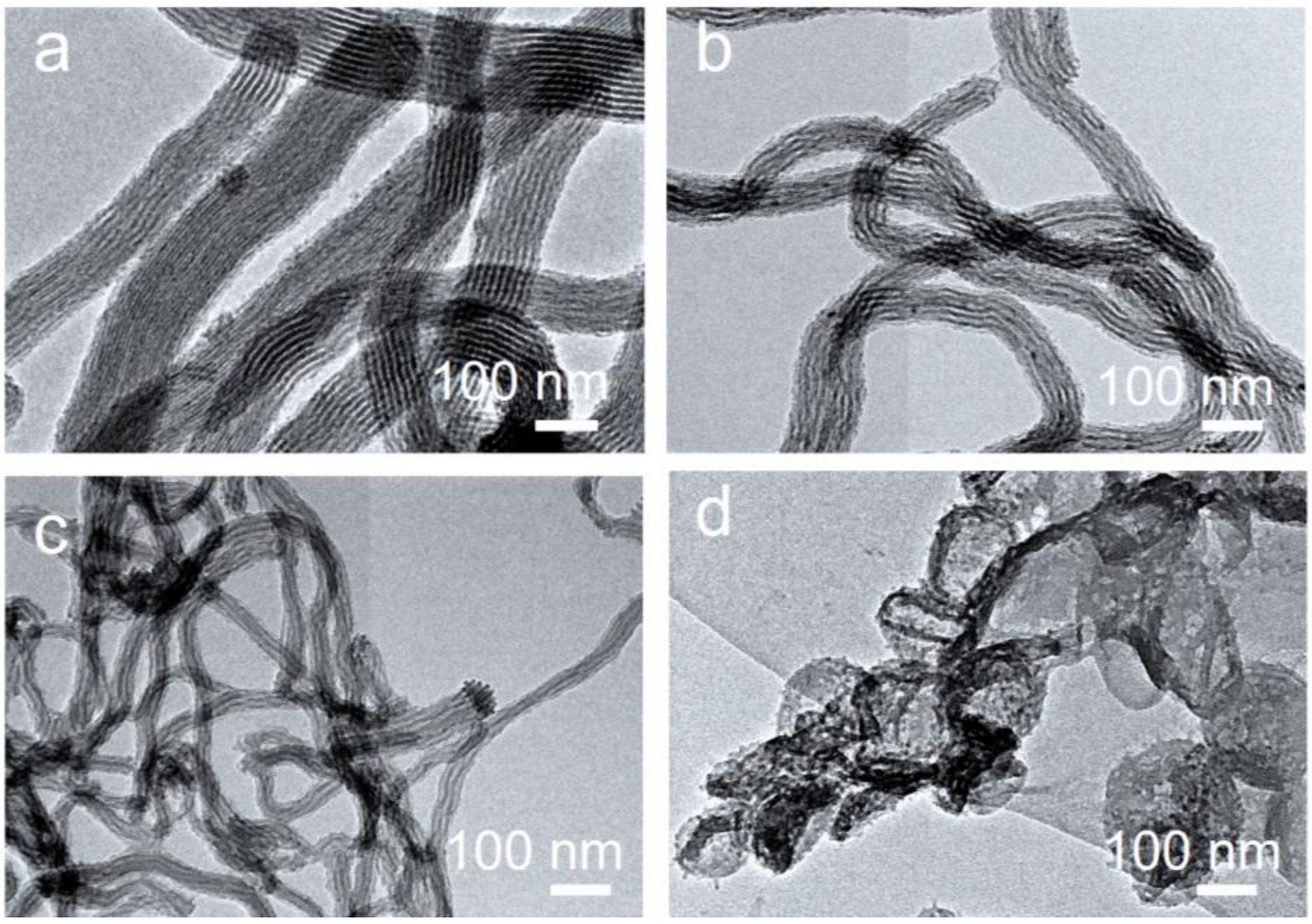

Supplementary Fig. 16. TEM images of MCN-x (x=1, 2, 3, 4) prepared by the emulsion-mediated assembly approach with various TOP content in the reaction system containing 10 mL ethanol. (a) 0.05 mL, (b) 0.1 mL, (c) 0.2 mL and (d) 0.3 mL.

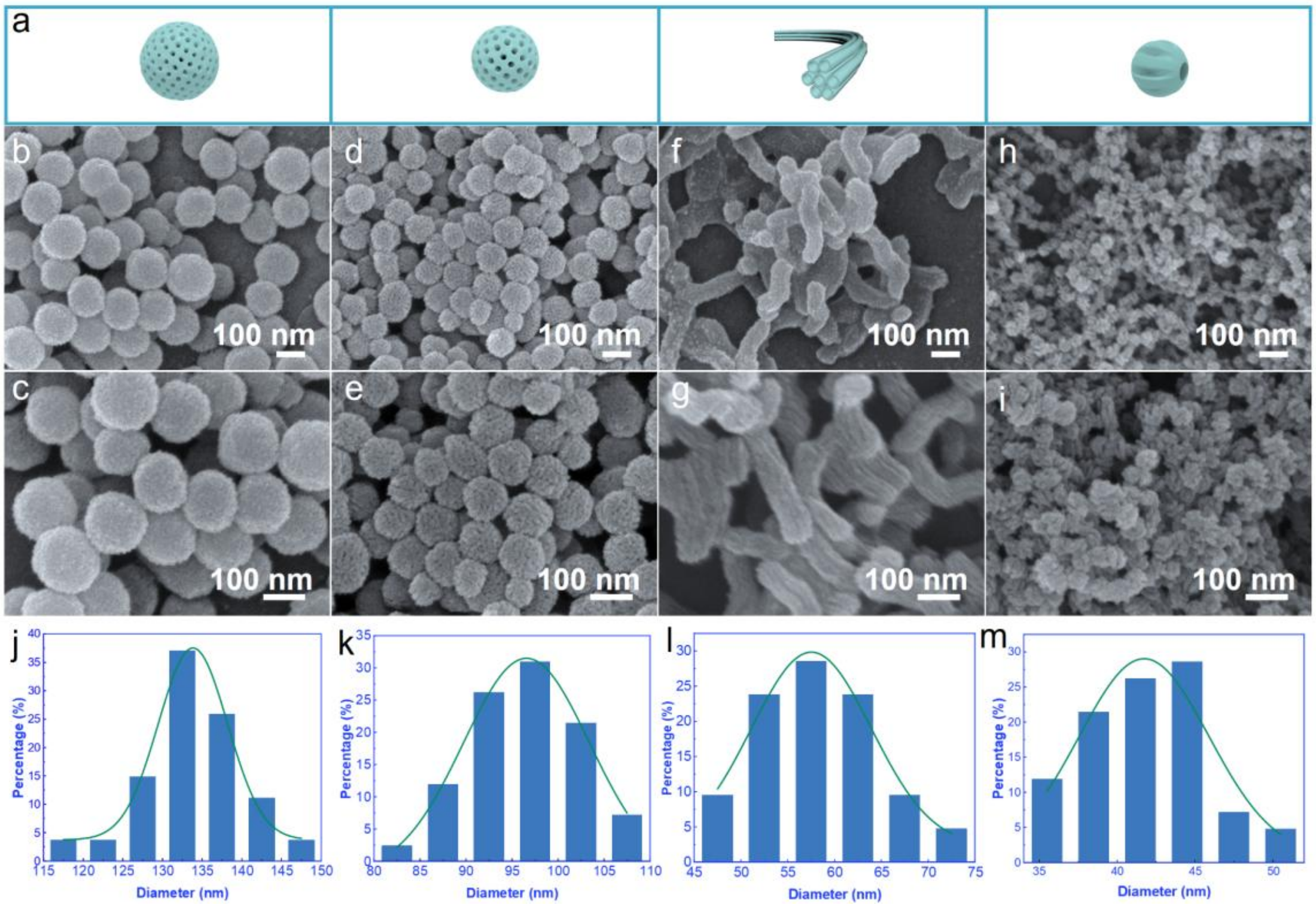

Supplementary Fig. 17. Morphology characterization of the MCNs-x (x=9, 12, 16, 21) prepared by the emulsion-mediated assembly approach with different volumes of TOP in reaction system containing 20 mL of ethanol. The synthesis was performed similarly to the Supplementary Method section except the volume of ethanol was 20 mL and the TOP contents and were varied. (a) Structural models, SEM images of (b, c) 0.05 mL; (d, e) 0.1 mL, (f, g) 0.2 mL, (h, i) 0.3 mL. (j–m) The corresponding particle-size distribution. The results indicate that as the ethanol is 20 mL, increasing the TOP leads to the morphology of MCNs-x from nanospheres to ordered nanorods and multi-chamber nanospheres. Particle-size distributions were obtained from 40 individual particles for each sample.

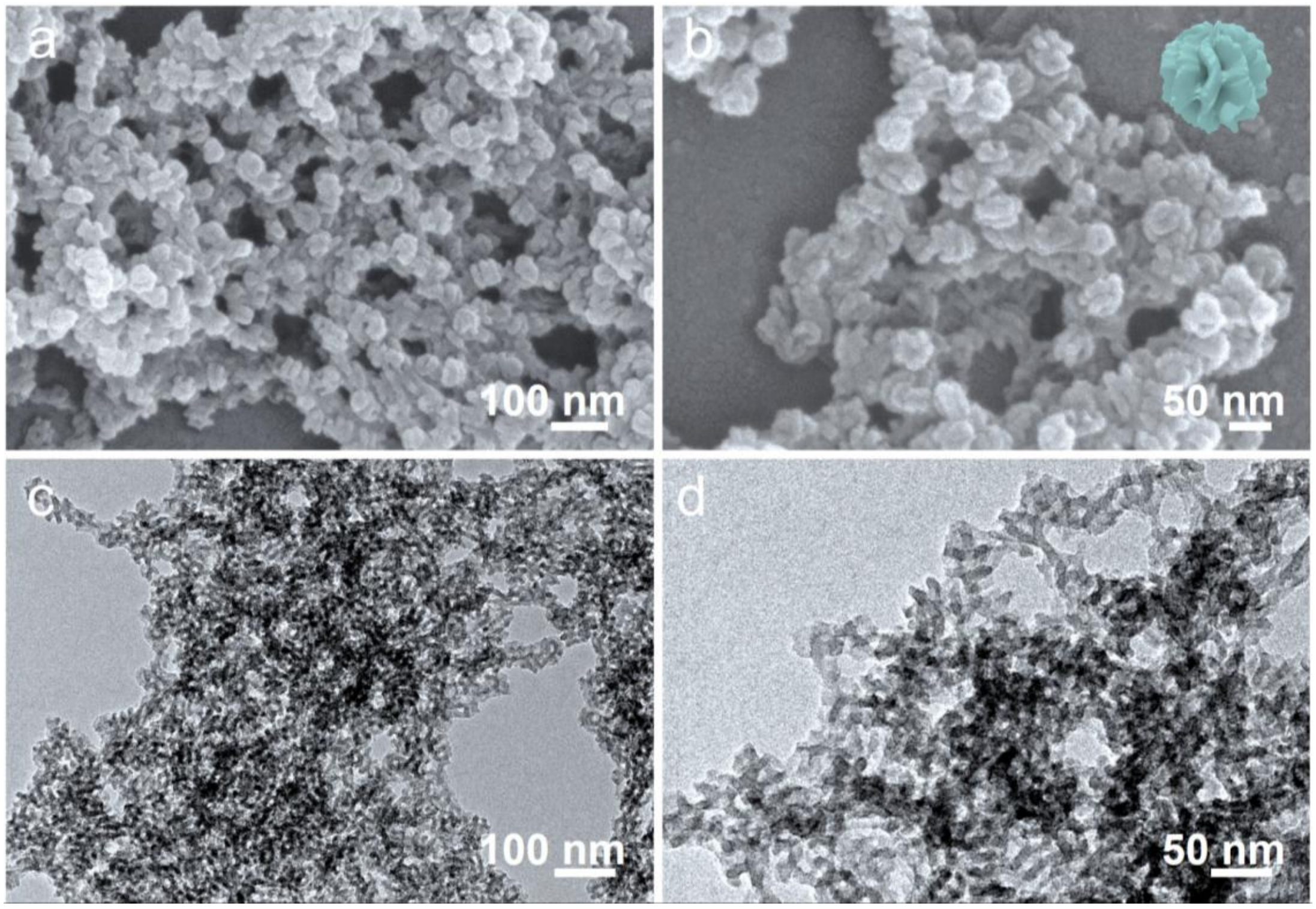

Supplementary Fig. 18. Morphology and mesostructure of dendritic nanospheres (MCNs-23). MCNs-23 were prepared by the emulsion-mediated assembly approach using 0.4 mL TOP and 20 mL ethanol. (a, b) SEM images at different magnifications showing the dendritic nanosphere morphology; inset in (b), corresponding structural model. (c, d) TEM images at different magnifications showing the mesoporous structure of MCNs-23.

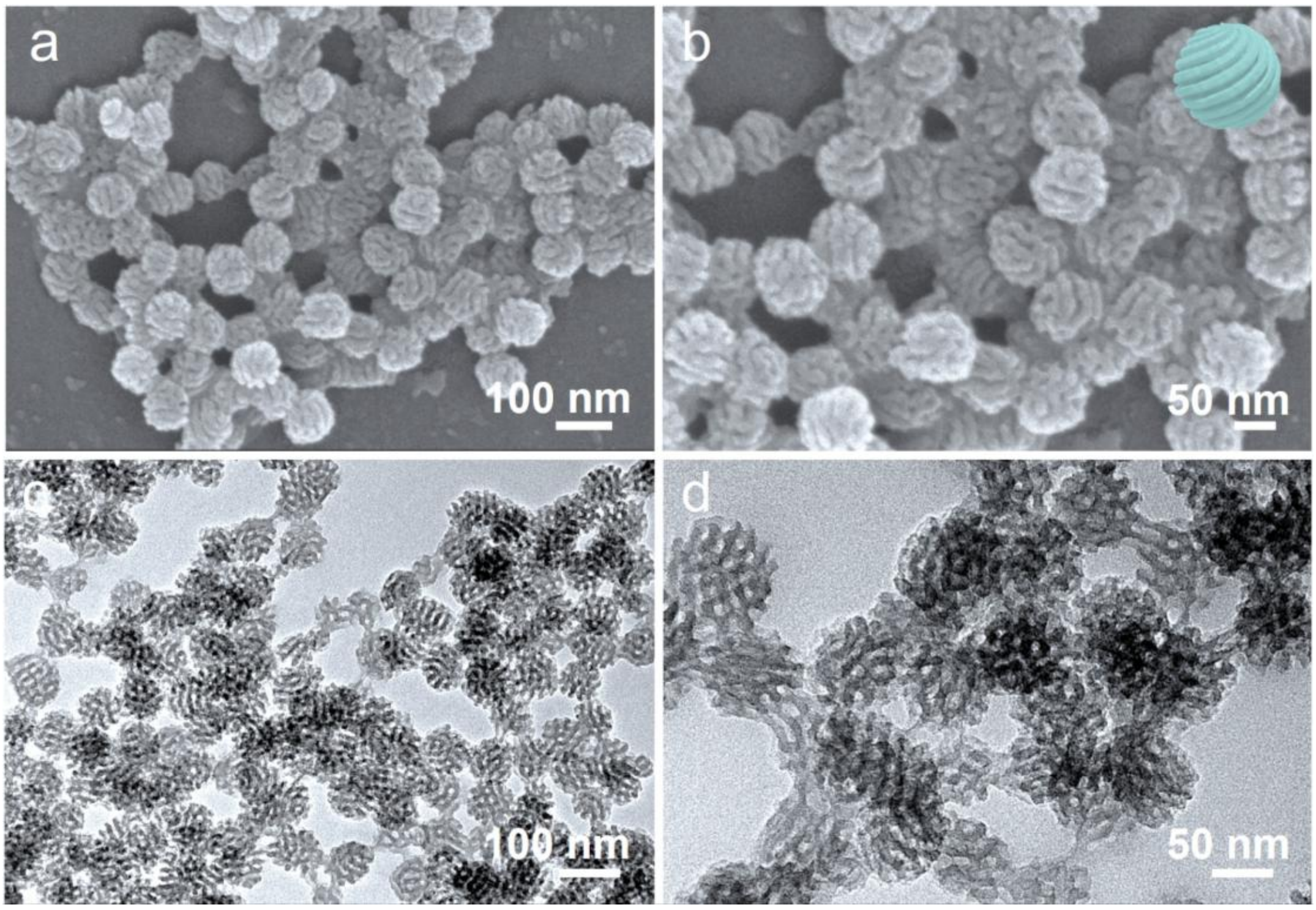

Supplementary Fig. 19. Morphology and mesostructure of gear-like carbon nanospheres (MCNs-24). MCNs-24 were prepared by the emulsion-mediated assembly approach using 0.4 mL TOP and 25 mL ethanol. (a, b) SEM images at different magnifications showing the uniform gear-like morphology; inset in (b), corresponding structural model. (c, d) TEM images at different magnifications showing the well-defined mesoporous structure of MCNs-24.

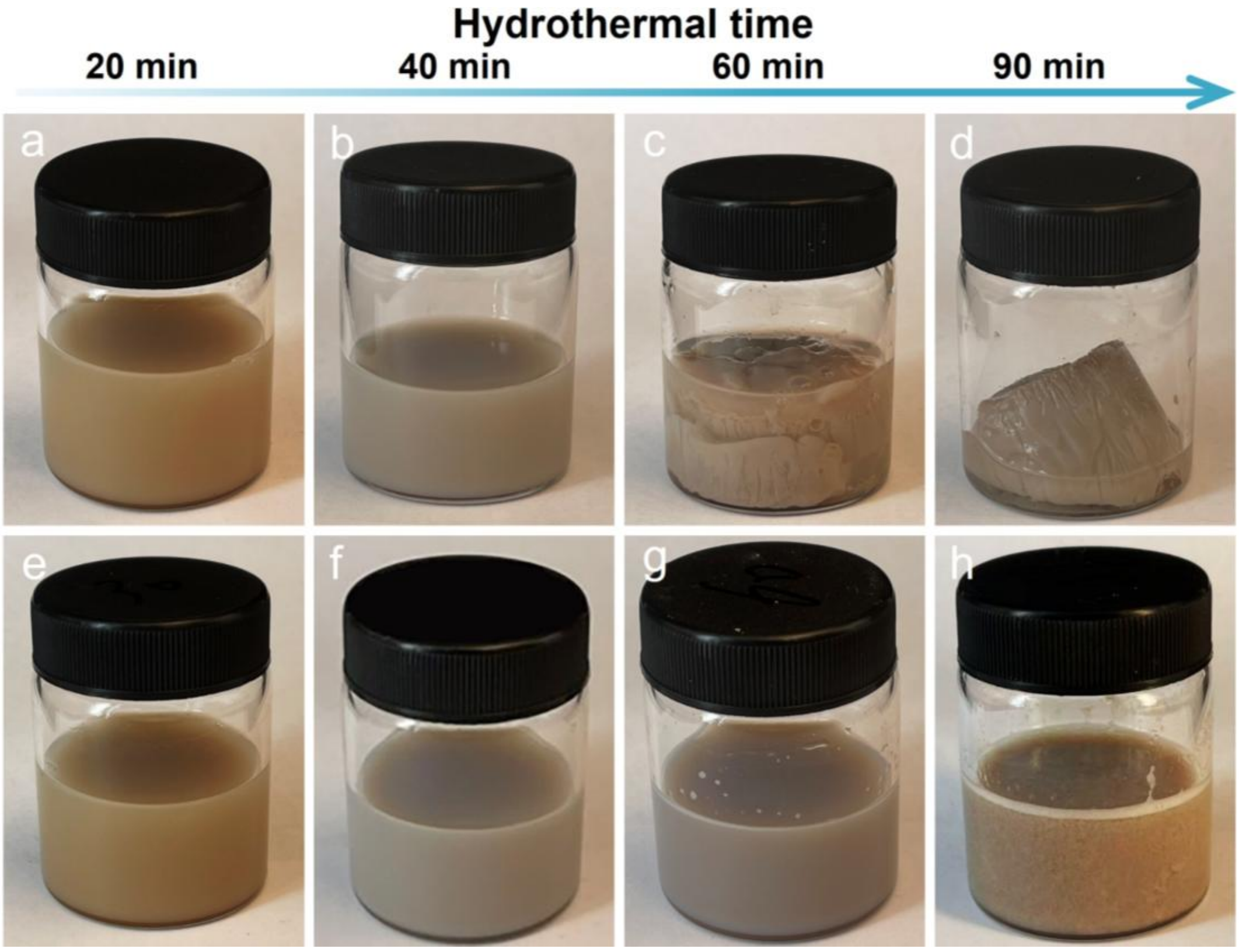

Supplementary Fig. 20. Photography illustration of the solution color change with different reaction times at 20, 40, 60 and 90 min in the reaction of growing polymeric precursors of nanofibers (MCNs-3, a–d) and nanospheres (MCNs-17, e–h) by emulsion-mediated assembly approach. The results indicate that the reaction system containing less ethanol produced the polymer product earlier in the same hydrothermal treatment time.

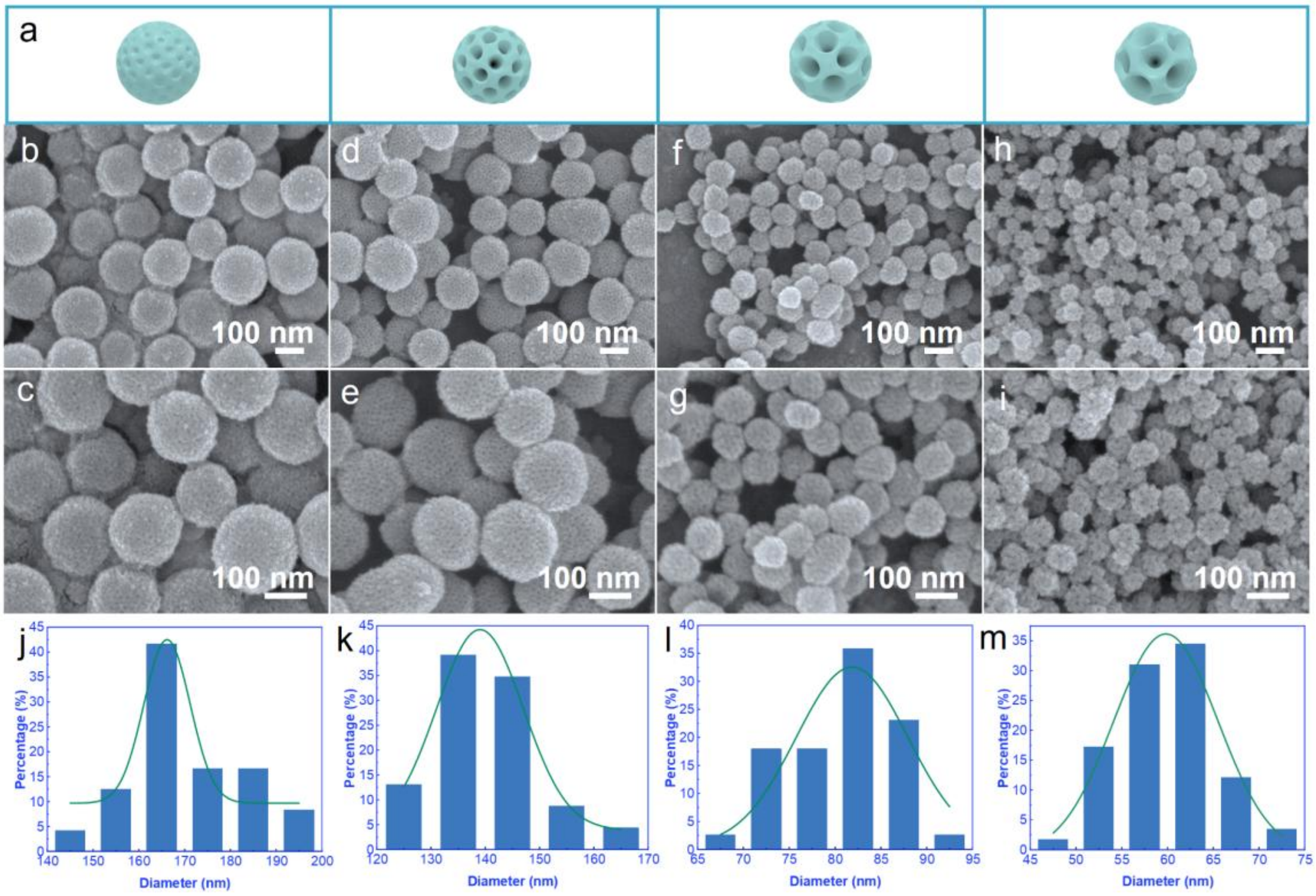

Supplementary Fig. 21. SEM images of the MCNs-x (x=10, 13, 17, 22) prepared by the emulsion-mediated assembly approach with different volumes of TOP in reaction system containing 25 mL ethanol. (a) Structural models, (b, c) 0.05 mL, (d, e) 0.1 mL, (f, g) 0.2 mL, (h, i) 0.3 mL. (j–m) The corresponding particle-size distributions. The synthesis was performed similarly to the Supplementary Method section except the volume of ethanol was 25 mL and the TOP contents were varied. The results indicate that by simply increasing the TOP volumes, golf ball-like nanospheres, raspberry-like nanospheres, multichambered nanospheres and branched nanoclusters were obtained. Particle-size distributions were obtained from 40 individual particles for each sample.

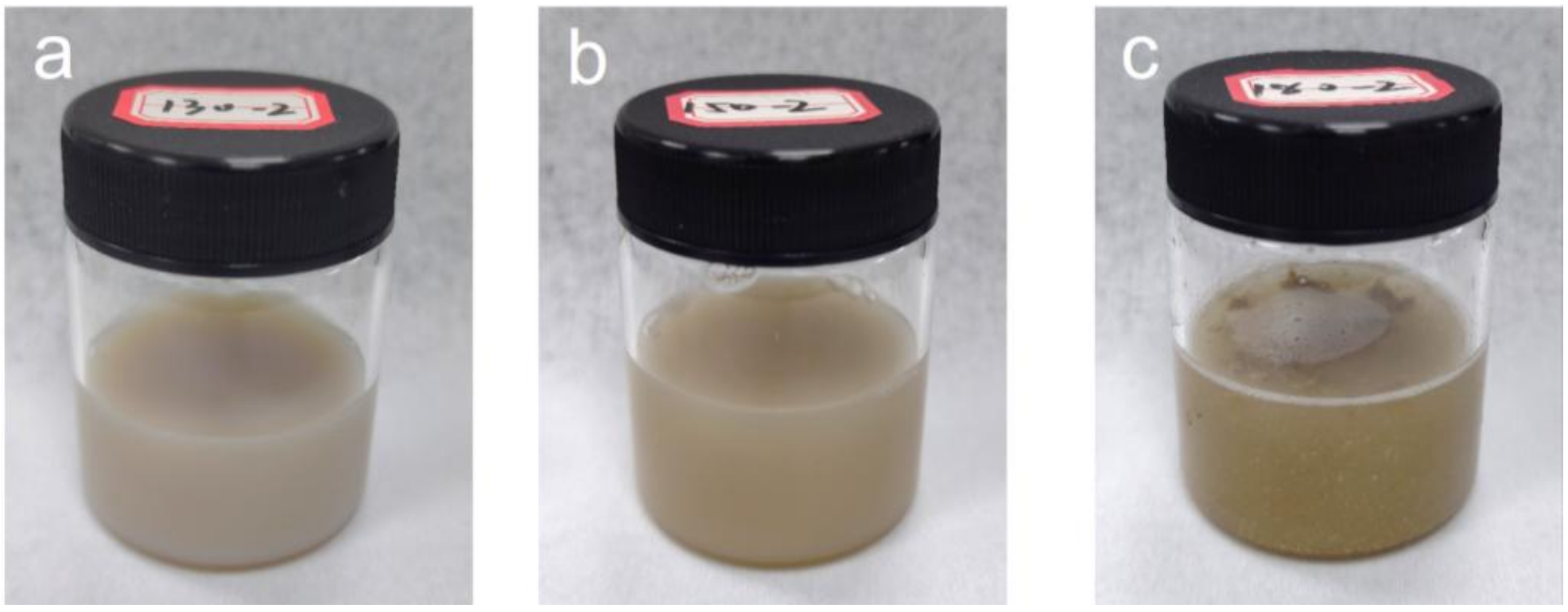

Supplementary Fig. 22. Photograph of the precursor solution after hydrothermal treatment for 40 min. (a) 130 °C, (b) 150 °C, (c)180 °C.

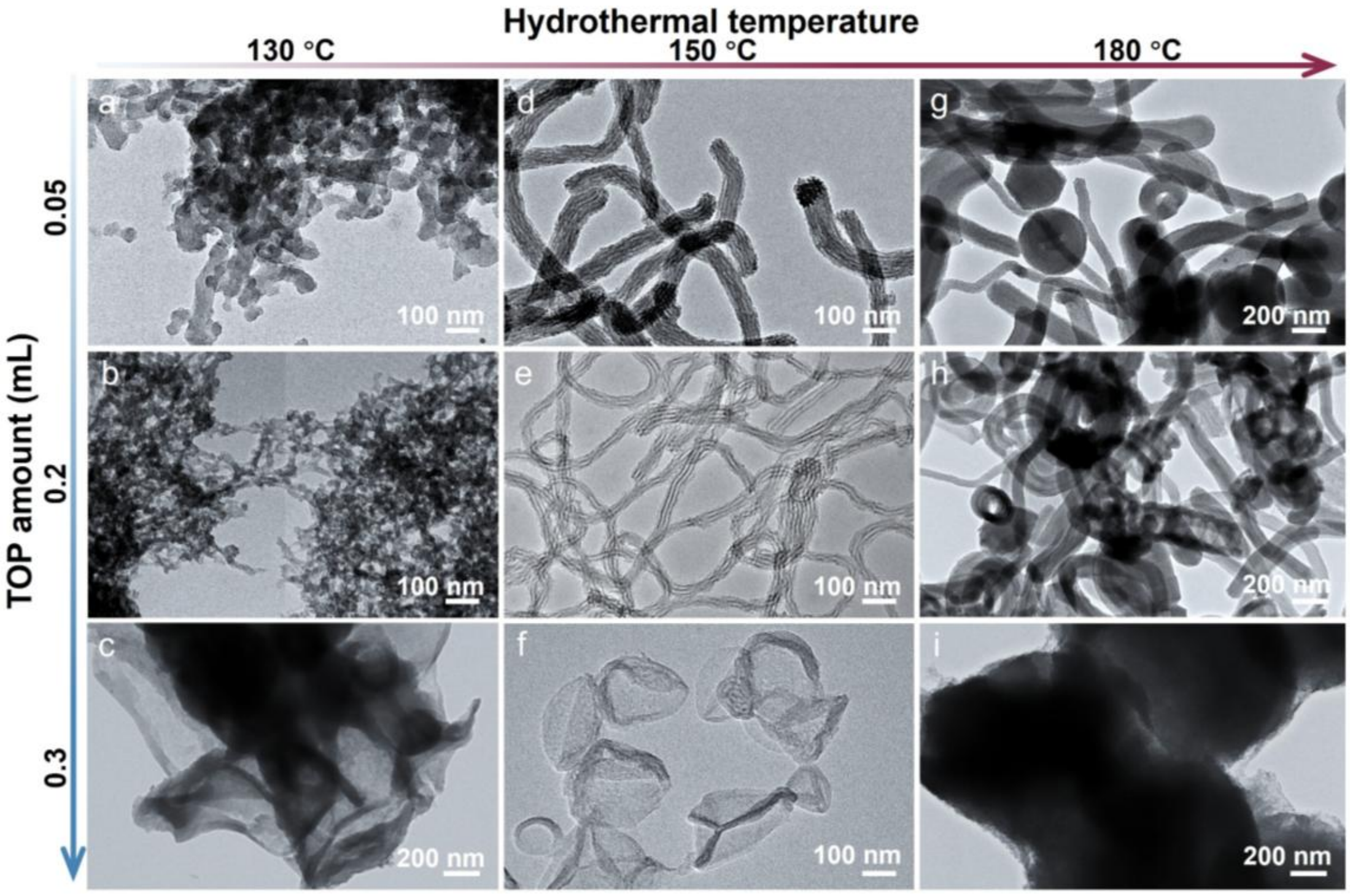

Supplementary Fig. 23. TEM images of the MCNs-x (x=1, 3, 4, 25–30) prepared by the emulsion-mediated assembly approach at different hydrothermal temperature with different additions of TOP. (a–c) 130 °C, (d–f) 150 °C, (g–i) 180 °C. The synthesis was performed similarly to the Supplementary Method section except the volume of ethanol was 10 mL and the TOP volumes and temperatures were varied.

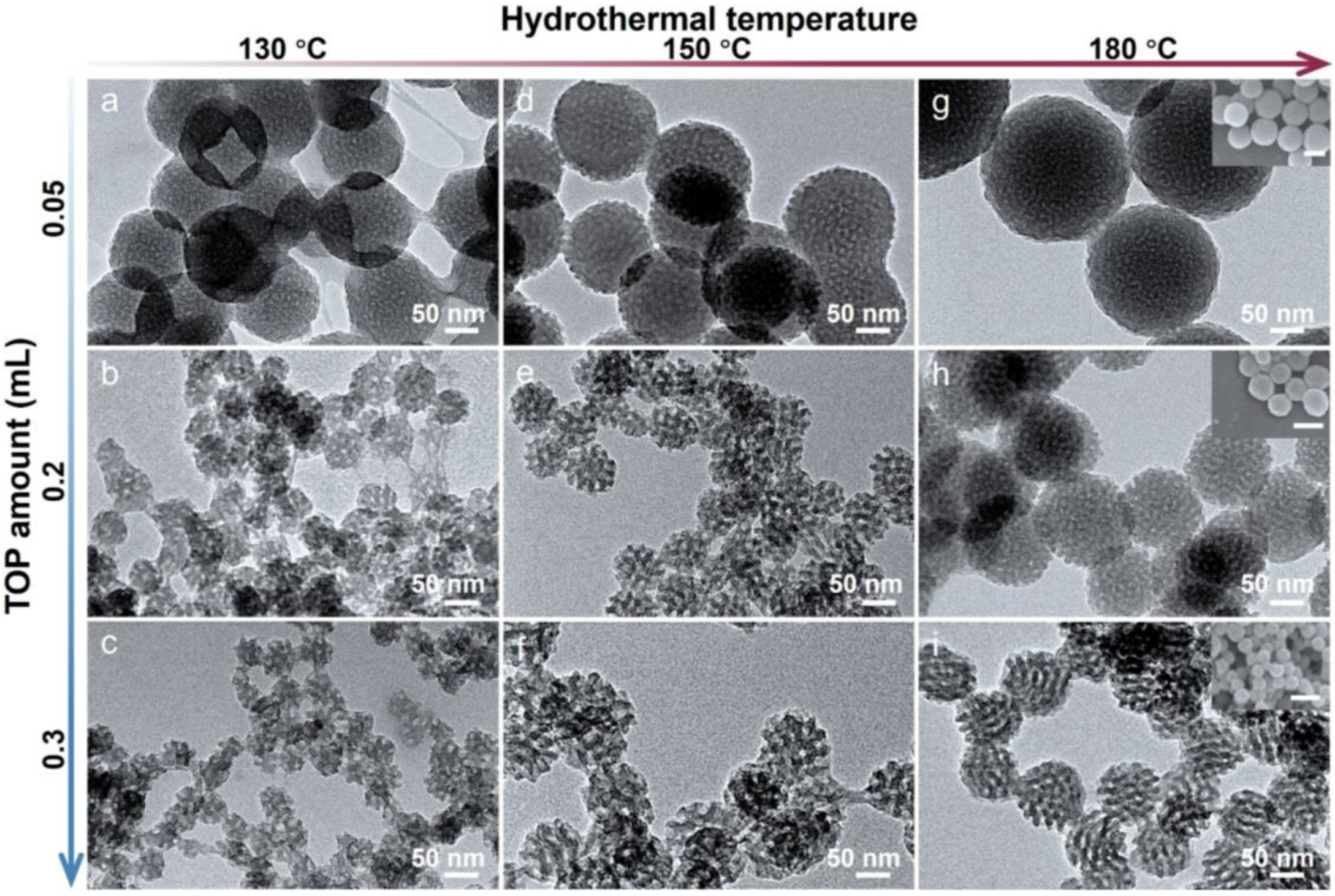

Supplementary Fig. 24. TEM images of the MCNs-x (x=10, 17, 22, 31–36) prepared by the emulsion-mediated assembly approach at different hydrothermal temperatures with different additions of TOP. (a–c) 130 °C, (d–f) 150 °C, (g–i) 180 °C. The synthesis was performed similarly to the Supplementary Method section except the volume of ethanol was 25 mL and the TOP volumes and temperatures were varied. The insets in (g–i) are the corresponding SEM images, with a scale bar of 200 nm.

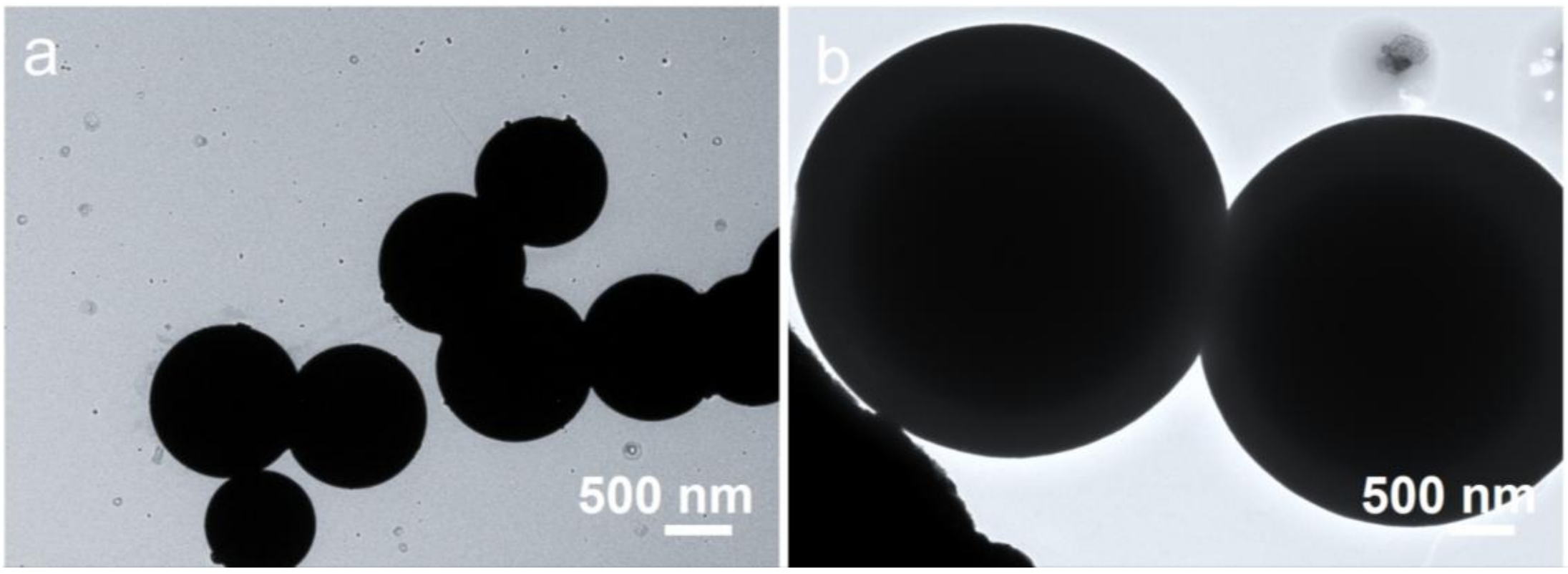

Supplementary Fig. 25. TEM images of the MCNs-x (x=37, 38) prepared by the emulsion-mediated assembly approach with 0.2 mL TOP in different ethanol content systems without adding Pluronic F127. (a) 10 mL, (b) 25 mL. It could be seen that only nonporous carbon spheres are formed without adding Pluronic F127, indicating that Pluronic F127 plays an essential role in the formation of mesostructure.

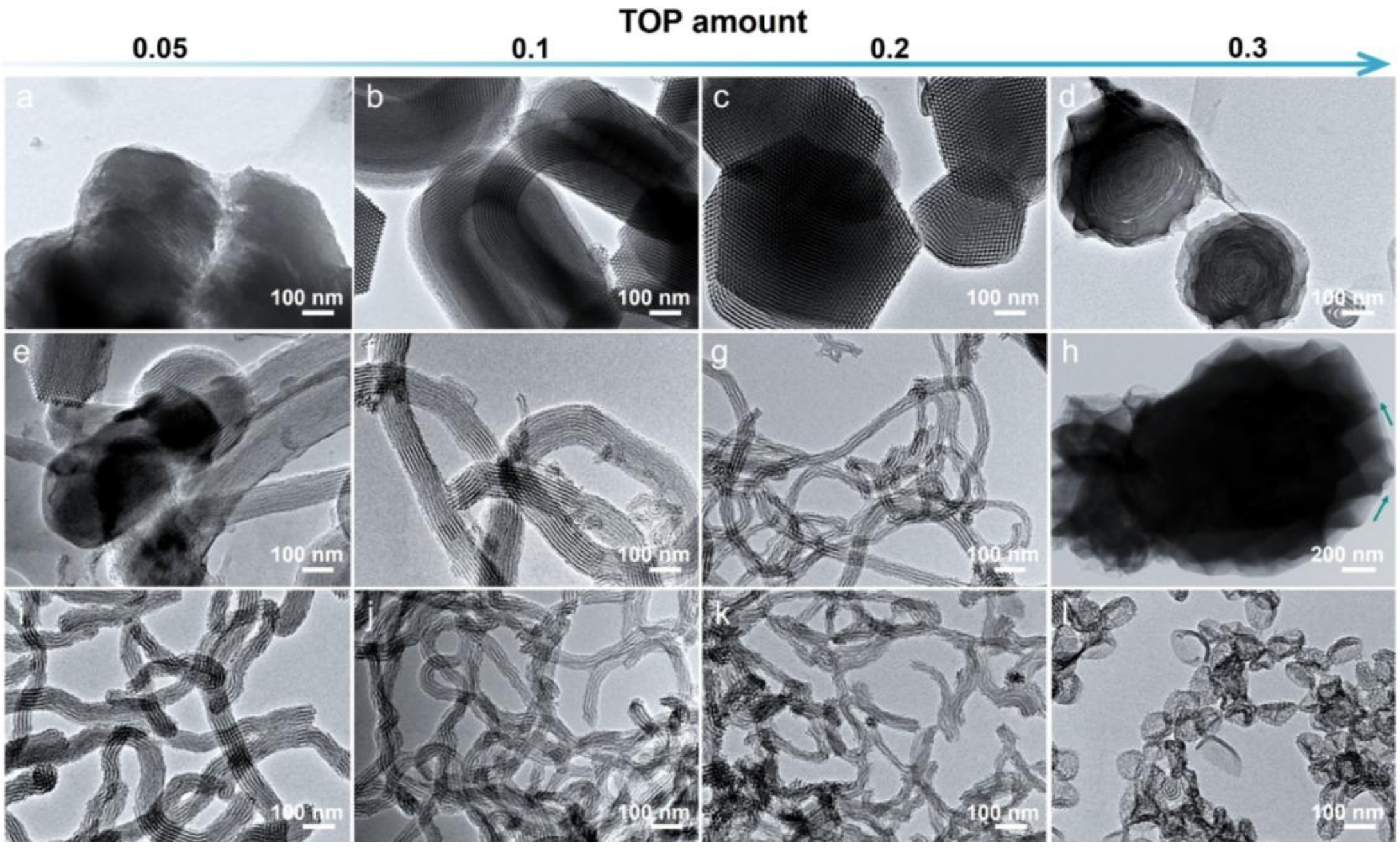

Supplementary Fig. 26. TEM images of the MCNs-x (x=39–50) synthesized in reaction system containing 10 mL ethanol with various F127 amounts and different TOP amounts. (a–d) 0.5 g F127, (e–h) 0.8 g F127, and (i–l) 1.5 g F127. The synthesis was performed similarly to the Supplementary Method section except the volume of ethanol was 10 mL and the TOP volumes and F127 amount were varied.

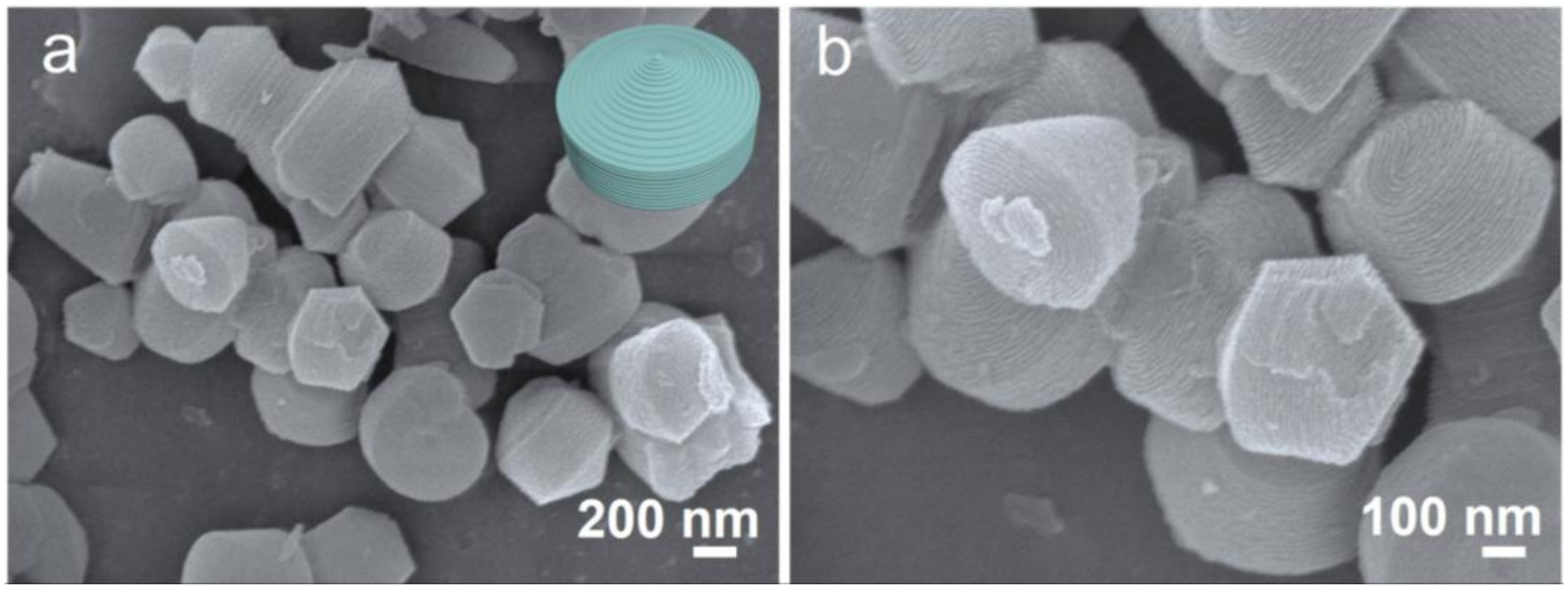

Supplementary Fig. 27. Gyroid-like morphology of MCNs-45. MCNs-45 were synthesized using 10 mL ethanol, 0.2 mL TOP and 0.5 g F127. (a) Low-magnification and (b) high-magnification SEM images showing the gyroid-like morphology with ordered curved features on the particle surface; inset in (a), corresponding structural model.

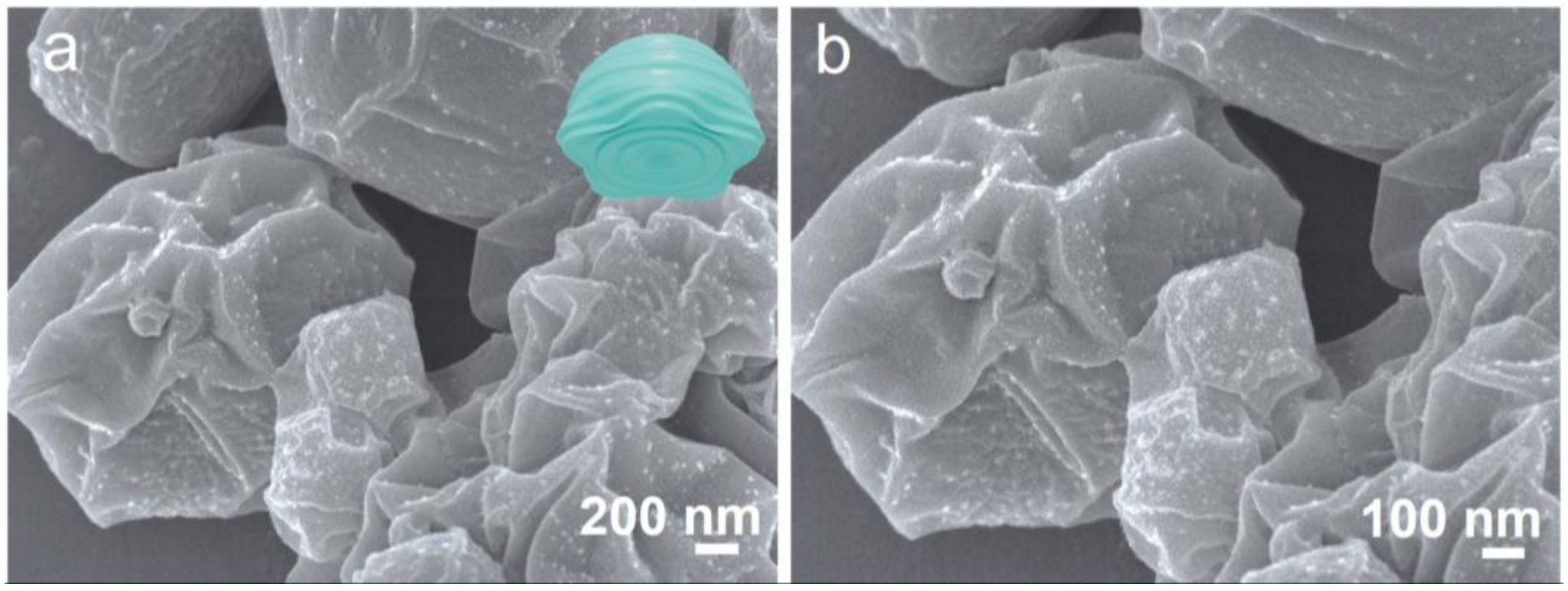

Supplementary Fig. 28. Concentric layered morphology of MCNs-48. MCNs-48 were synthesized using 10 mL ethanol, 0.3 mL TOP and 0.5 g F127. (a) Low-magnification and (b) high-magnification SEM images showing the concentric layered morphology; inset in (a), corresponding structural model.

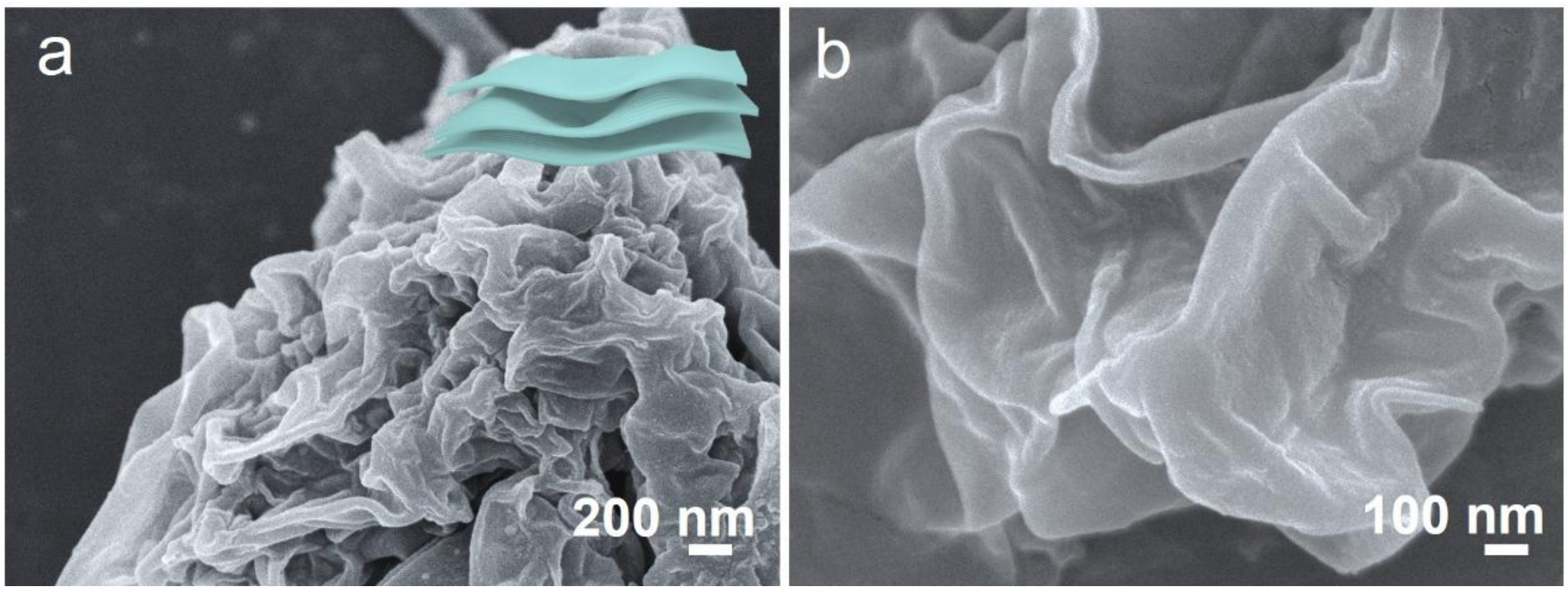

Supplementary Fig. 29. Flake-like morphology of MCNs-49. MCNs-49 were synthesized using 10 mL ethanol, 0.3 mL TOP and 0.8 g F127. (a) Low-magnification and (b) high-magnification SEM images showing the flake-like morphology; inset in (a), corresponding structural model.

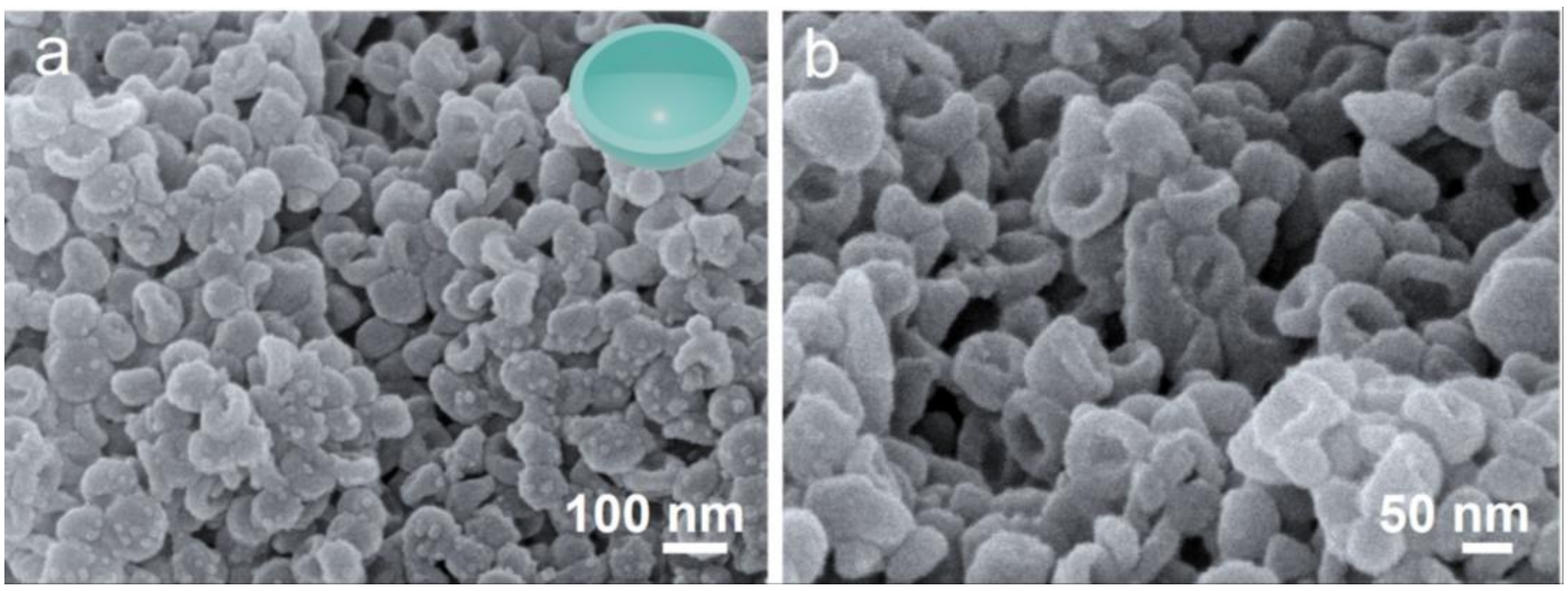

Supplementary Fig. 30. Bowl-like nanosphere morphology of MCNs-50. MCNs-50 were synthesized using 10 mL ethanol, 0.3 mL TOP and 1.5 g F127. (a) Low-magnification and (b) high-magnification SEM images showing the bowl-like nanosphere morphology; inset in (a), corresponding structural model.

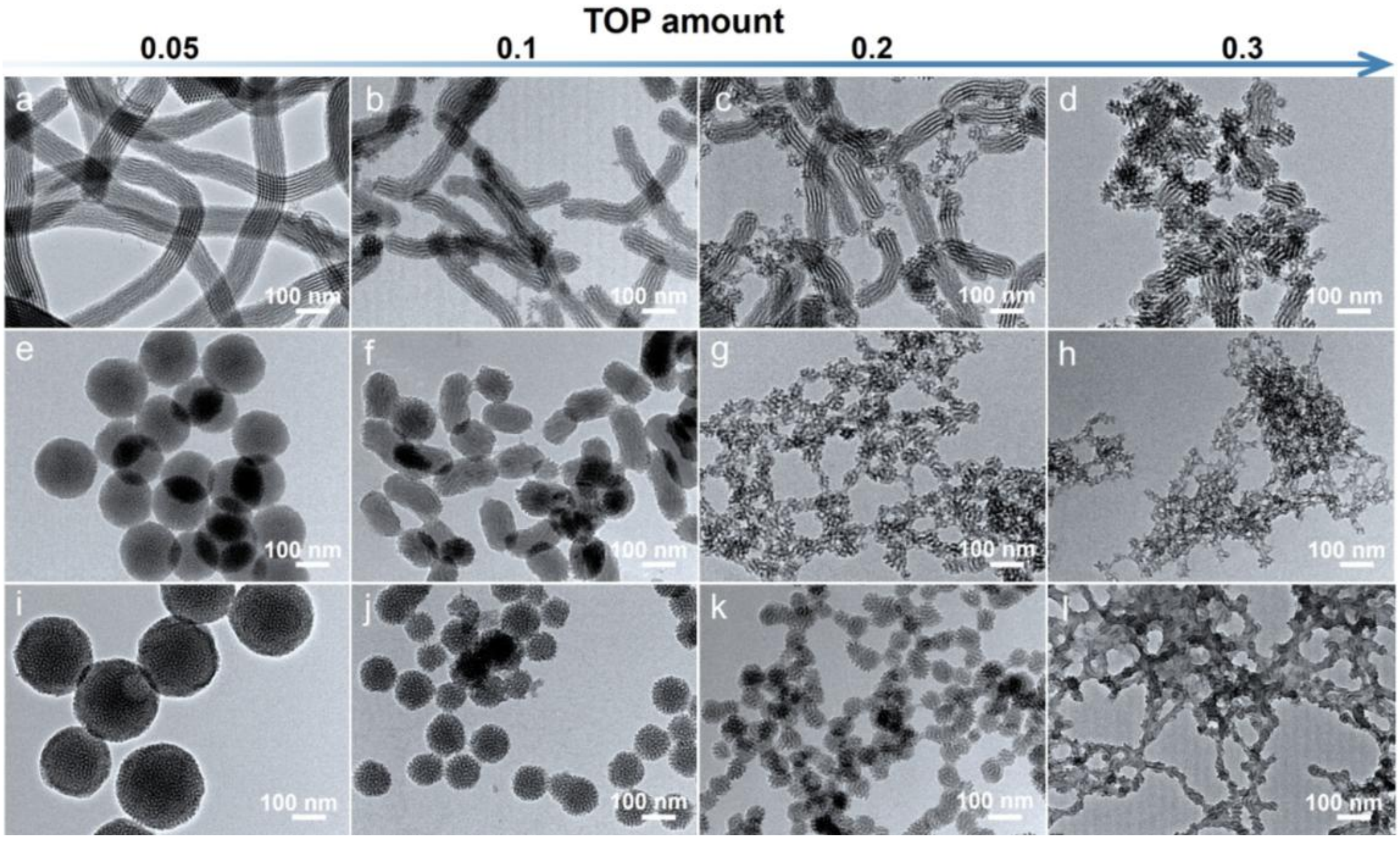

Supplementary Fig. 31. TEM images of the MCNs-x (x=51–62) synthesized in reaction system containing 25 mL ethanol with various F127 amounts and different TOP amounts. (a–d) 0.5 g F127, (e–h) 0.8 g F127, and (i–l) 1.5 g F127. The synthesis was performed similarly to the Supplementary Method section except the volume of ethanol was 25 mL and the TOP volumes and F127 amounts were varied.

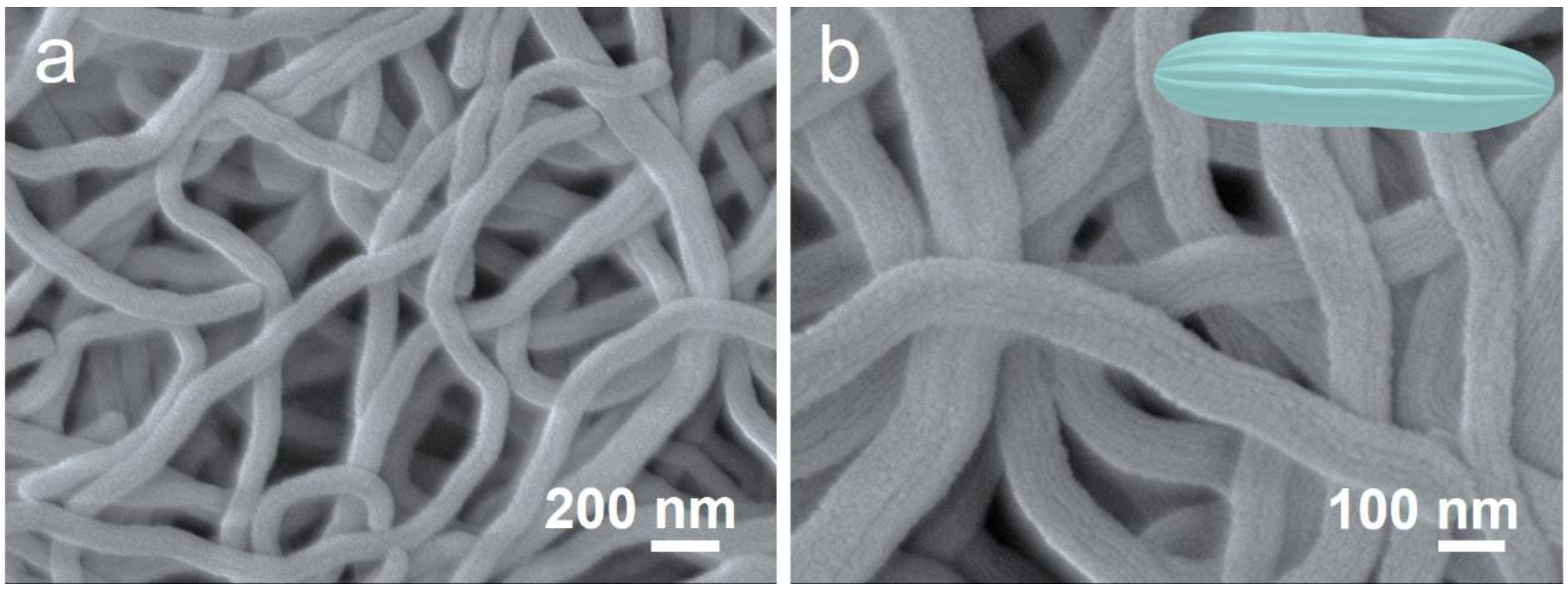

Supplementary Fig. 32. Fiber-like morphology of MCNs-51. MCNs-51 were synthesized using 25 mL ethanol, 0.05 mL TOP and 0.5 g F127. (a) Low-magnification and (b) high-magnification SEM images showing the fiber-like morphology and rough surfaces decorated with small, raised particles originating from the packing and growth of cylindrical micelles; inset in (b), corresponding structural model.

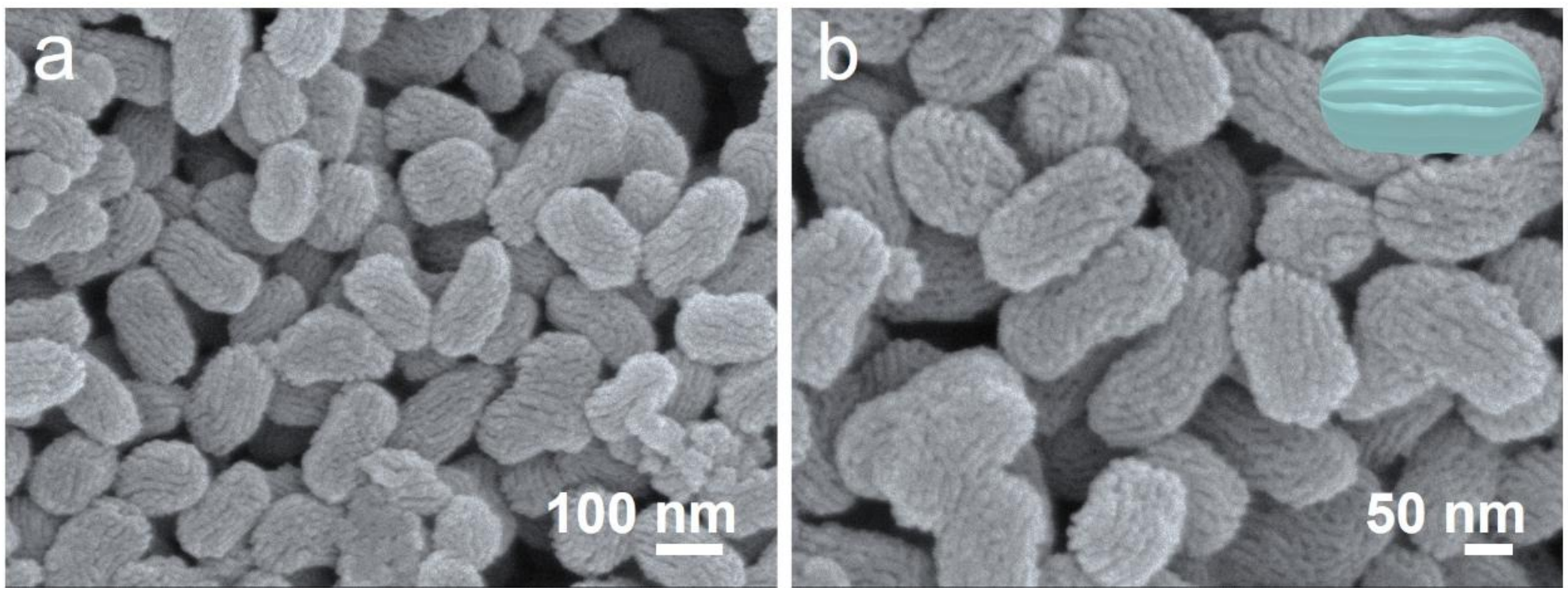

Supplementary Fig. 33. Worm-like morphology of MCNs-55. MCNs-55 were synthesized using 25 mL ethanol, 0.1 mL TOP and 0.8 g F127. (a) Low-magnification and (b) high-magnification SEM images showing the worm-like morphology and rough surfaces of MCNs-55; inset in (b), corresponding structural model.

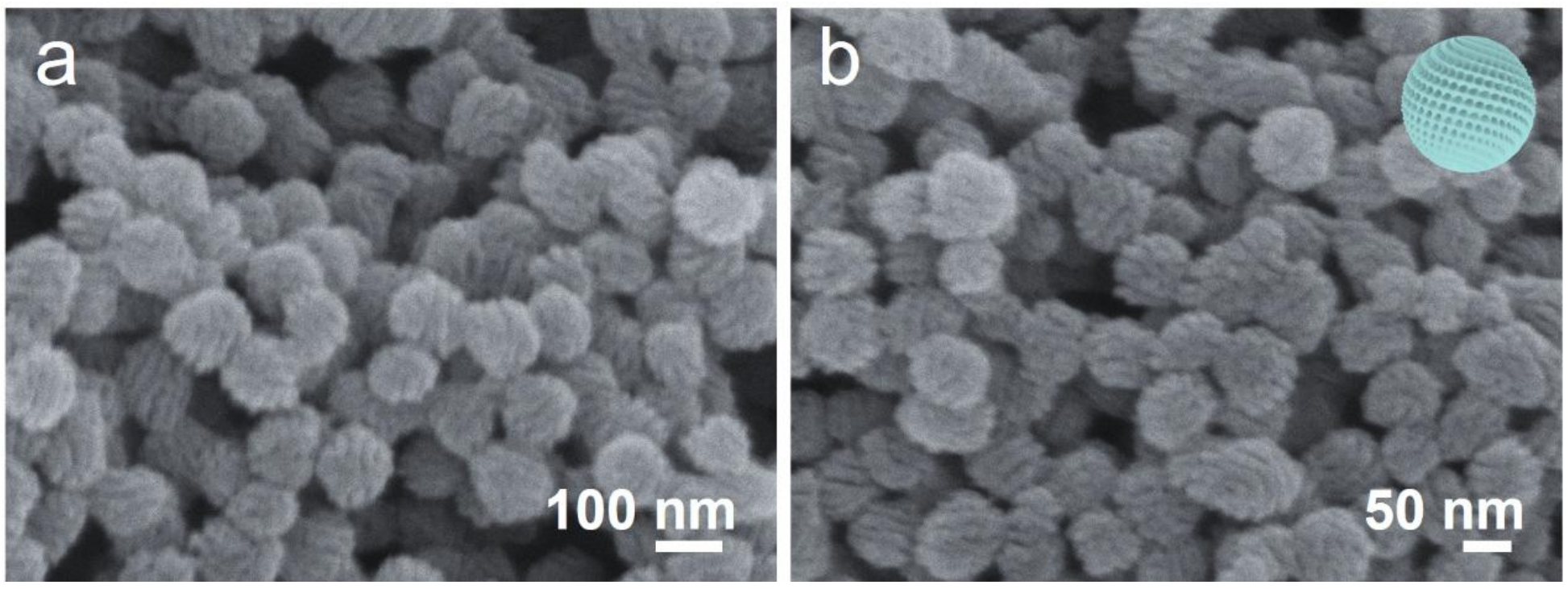

Supplementary Fig. 34. Pinecone-like nanosphere morphology of MCNs-59. MCNs-59 were synthesized using 25 mL ethanol, 0.2 mL TOP and 1.5 g F127. (a) Low-magnification and (b) high-magnification SEM images showing the pinecone-like nanosphere morphology with raised surface features; inset in (b), corresponding structural model.

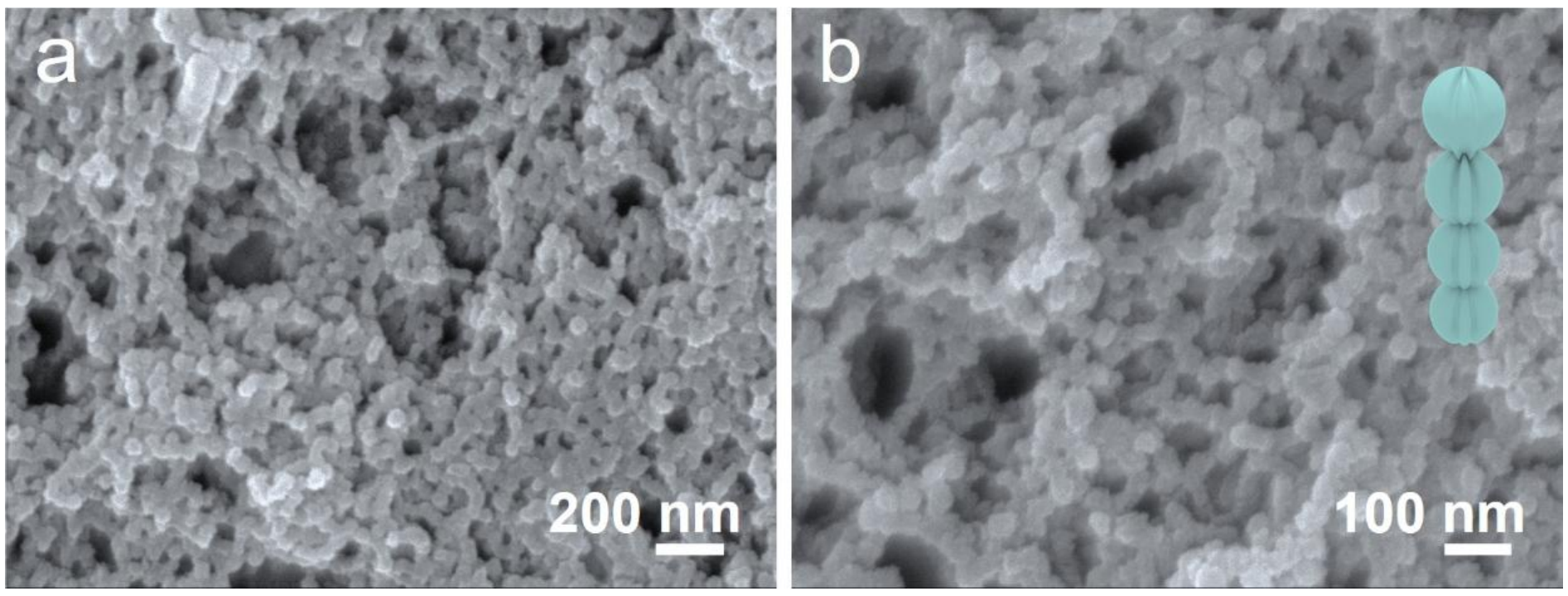

Supplementary Fig. 35. Interconnected nanosphere morphology of MCNs-62. MCNs-62 were synthesized using 25 mL ethanol, 0.3 mL TOP and 1.5 g F127. (a) Low-magnification and (b) high-magnification SEM images showing the interconnected nanosphere morphology; inset in (b), corresponding structural model.

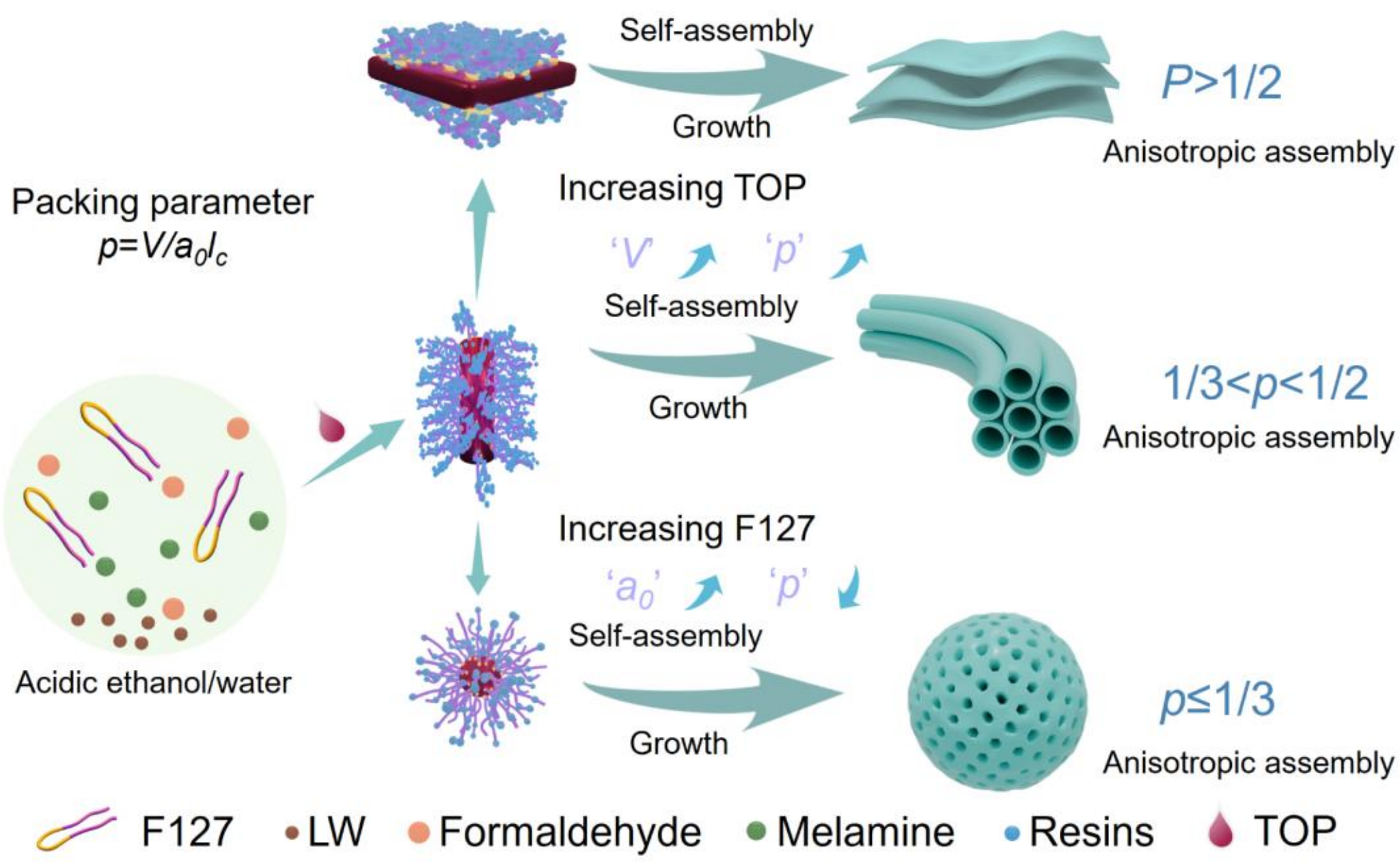

Supplementary Fig. 36. Schematic illustration of the mechanism of the F127-controlled micelle self-assembly strategy. The results indicate that by rationally adjusting the amounts of Pluronic F127 and TOP, it is possible to regulate the micelle stacking parameters, thereby determining the mesostructure.

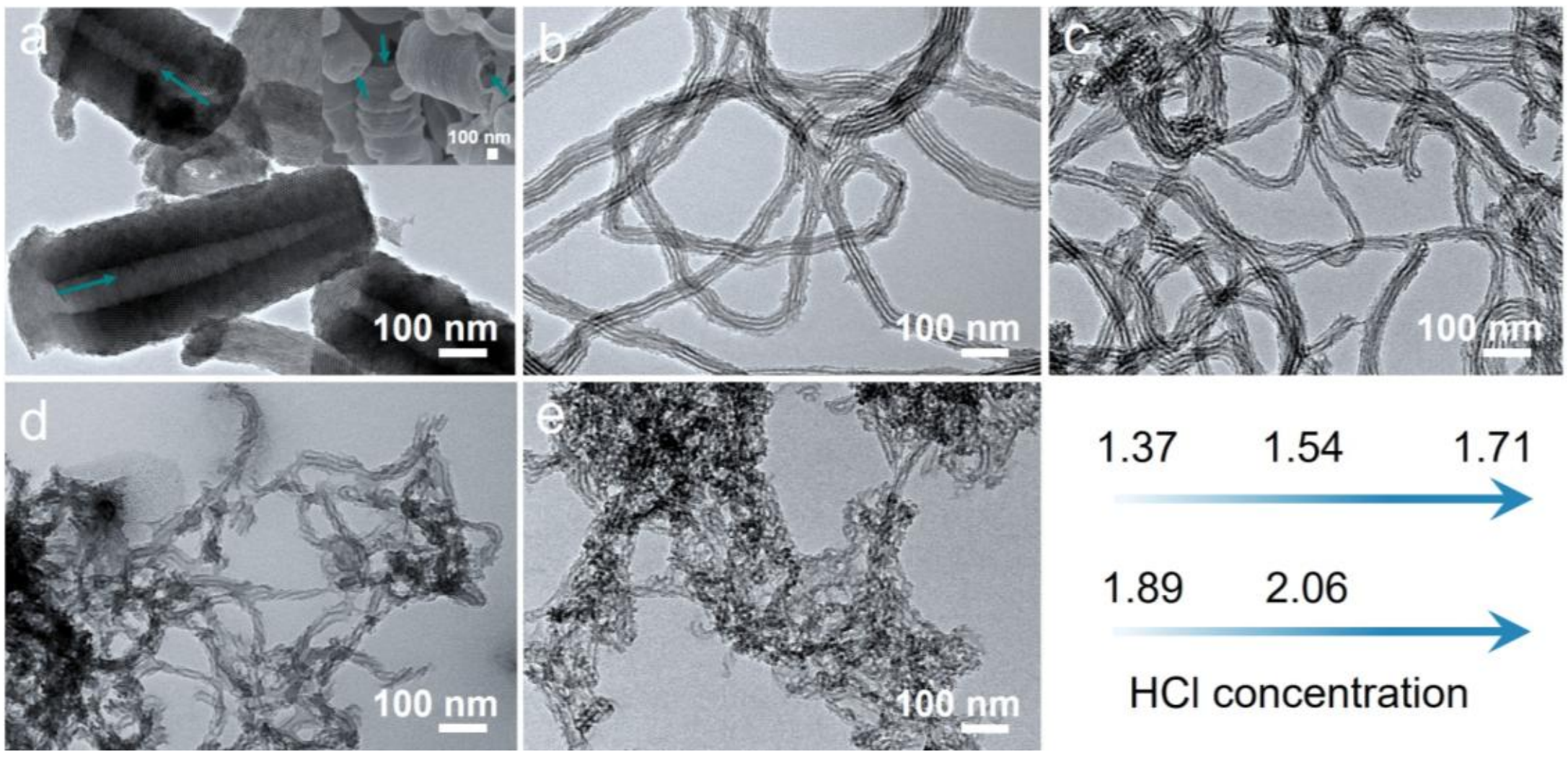

Supplementary Fig. 37. TEM images of MCNs-x (x=63–66) synthesized in reaction system containing 10 mL of ethanol and 0.2 mL TOP at different HCl concentrations. The synthesis was performed similarly to those described in the Supplementary Method section, except that the HCl concentration was varied. (a) 1.37 M, (b) 1.54 M, (c) 1.71 M, (d) 1.89 M, (e) 2.06 M. The results indicate that with increasing acid concentration, the products evolve from hollow helical structures to well-defined hollow nanofibers and further to shortened/agglomerated hollow nanorods with diminished mesostructural definition.

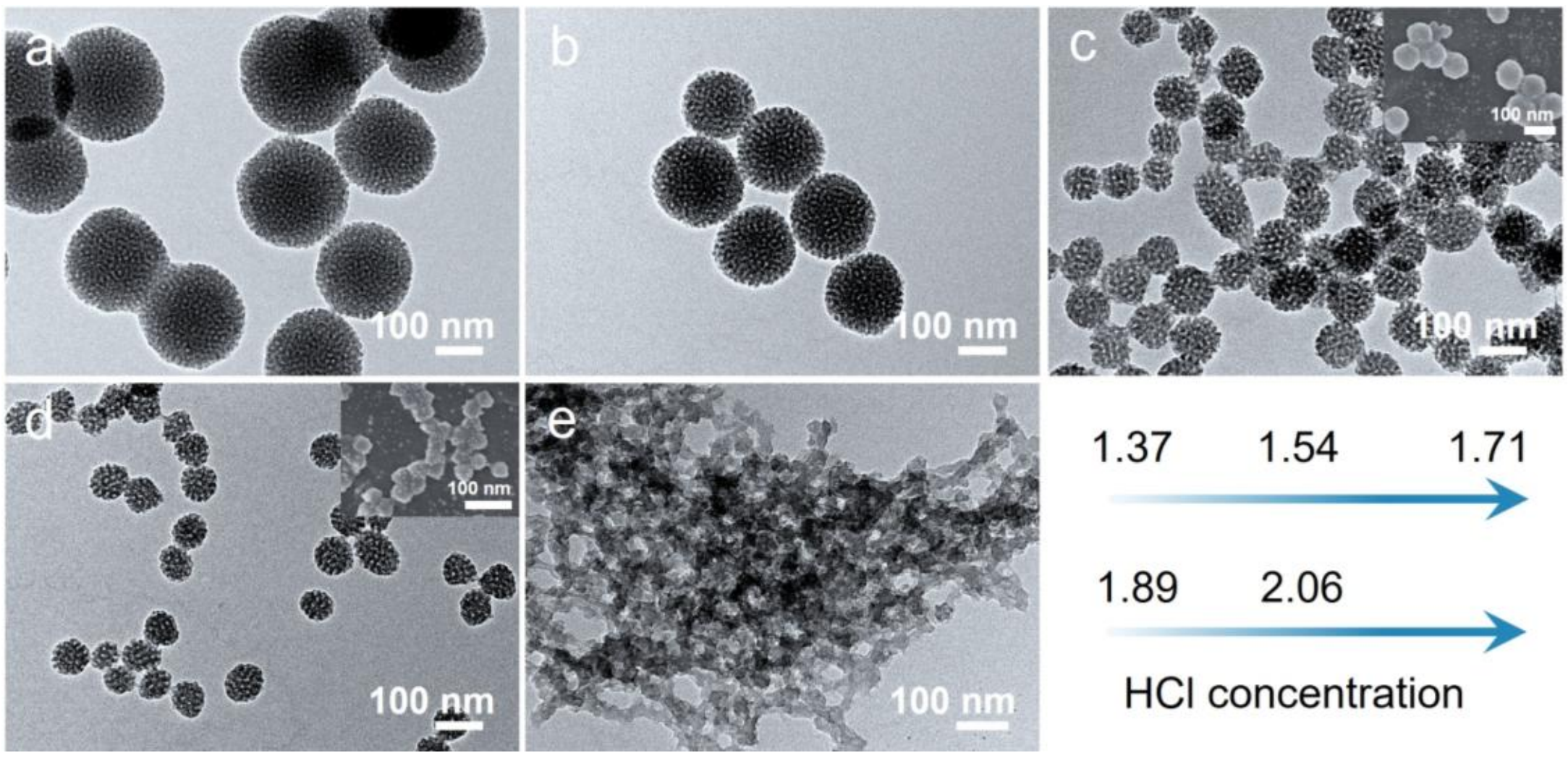

Supplementary Fig. 38. TEM images of MCNs-x (x=67–71) synthesized in reaction system containing 25 mL ethanol and 0.2 mL TOP at different HCl concentrations. The synthesis was performed similarly to those described in the Supplementary Method section, except that the HCl concentration was varied. (a) 1.37 M, (b) 1.54 M, (c) 1.71 M, (d) 1.89 M, (e) 2.06 M. With increasing acid concentration, the particle size decreases progressively, and the products evolve toward densely aggregated particles.

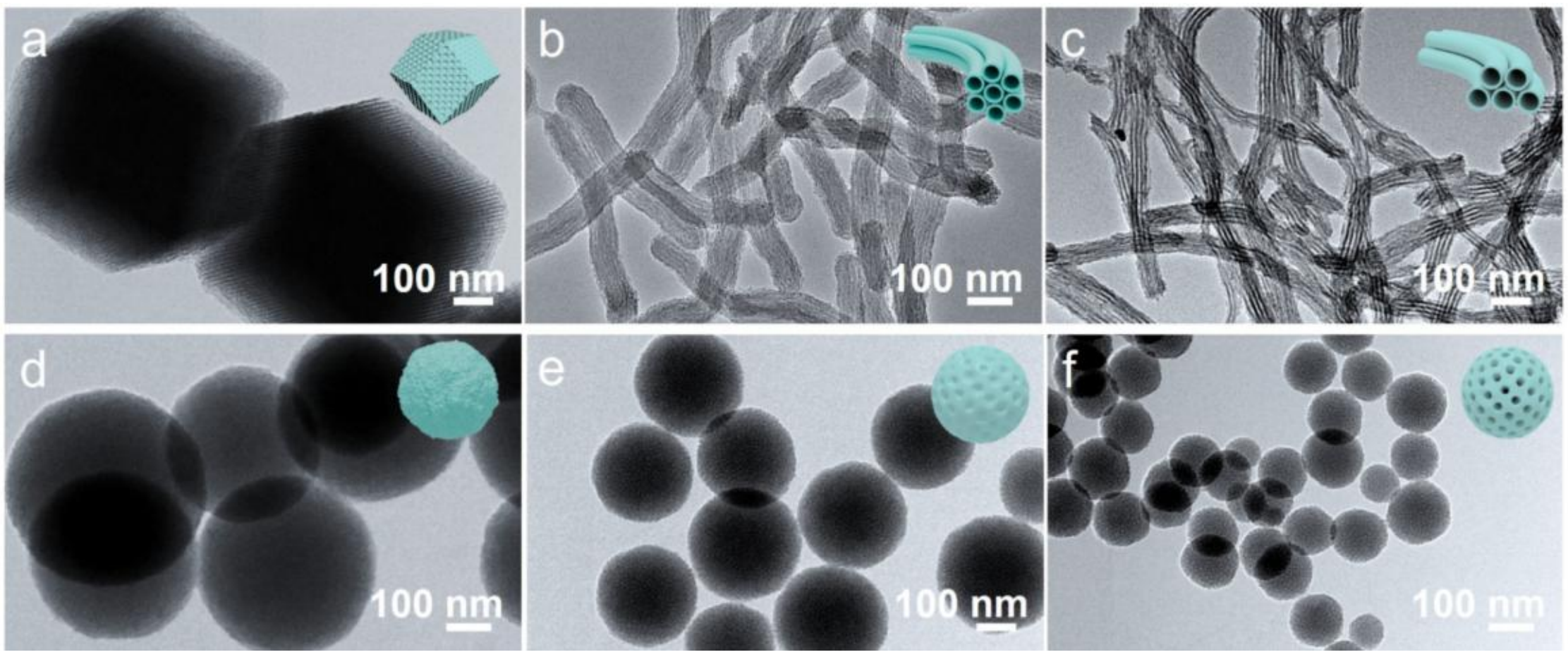

Supplementary Fig. 39. TEM images of mesoporous carbon nanomaterials synthesized by replacing TOP with trialkyl phosphate oils of different alkyl-chain lengths and hydrophobicity. Oil phase: (a, d) TEP, (b, e) TBP and (c, f) TAP. For the fiber-forming regime (a–c), increasing alkyl-chain length induced a transition from particles with ordered spherical mesopores to ordered mesoporous nanofibers with progressively reduced channel number and a tendency toward hollowing. For the nanosphere-forming regime (d–f), increasing alkyl-chain length decreased particle size and enhanced mesostructural definition, accompanied by an evolution toward multichambered mesoporous nanospheres.

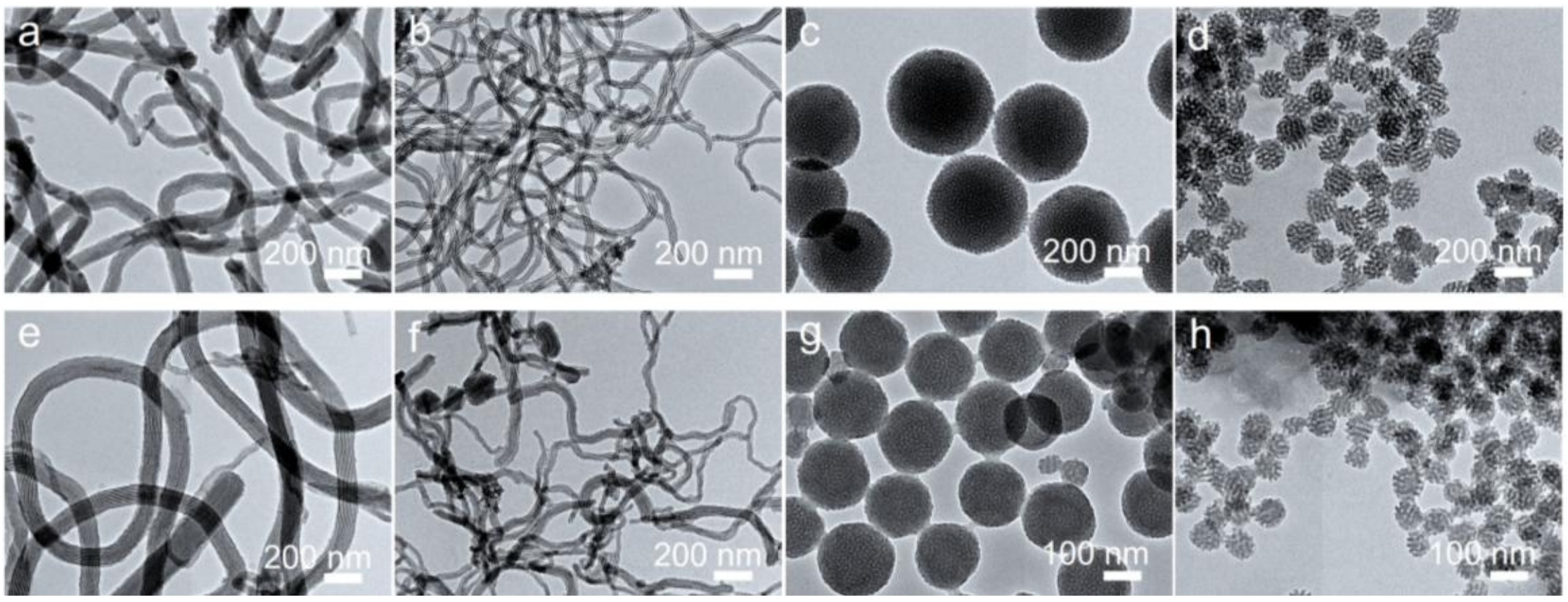

Supplementary Fig. 40. TEM images of MCNs synthesized from coconut shell (a–d) and corn straw (e–h). The synthesis condition was similar to the nanofibers and nanospheres. (a, e) 10 mL ethanol and 0.05 mL TOP. (b, f) 10 mL ethanol and 0.2 mL TOP. (c, g) 25 mL ethanol and 0.05 mL TOP. (d, h) 25 mL ethanol and 0.2 mL TOP.

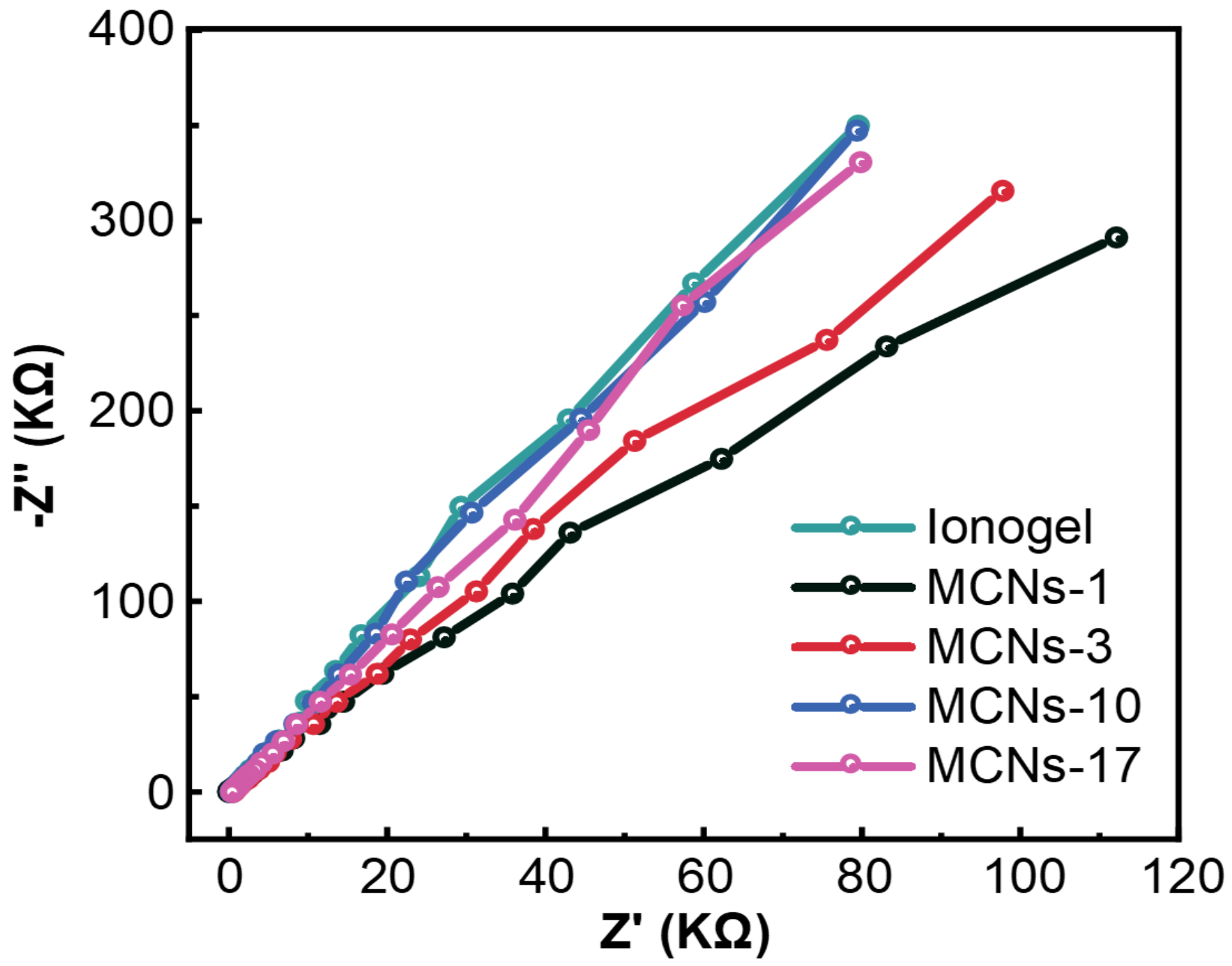

Supplementary Fig. 41. Nyquist plots of MCNs ionogels.

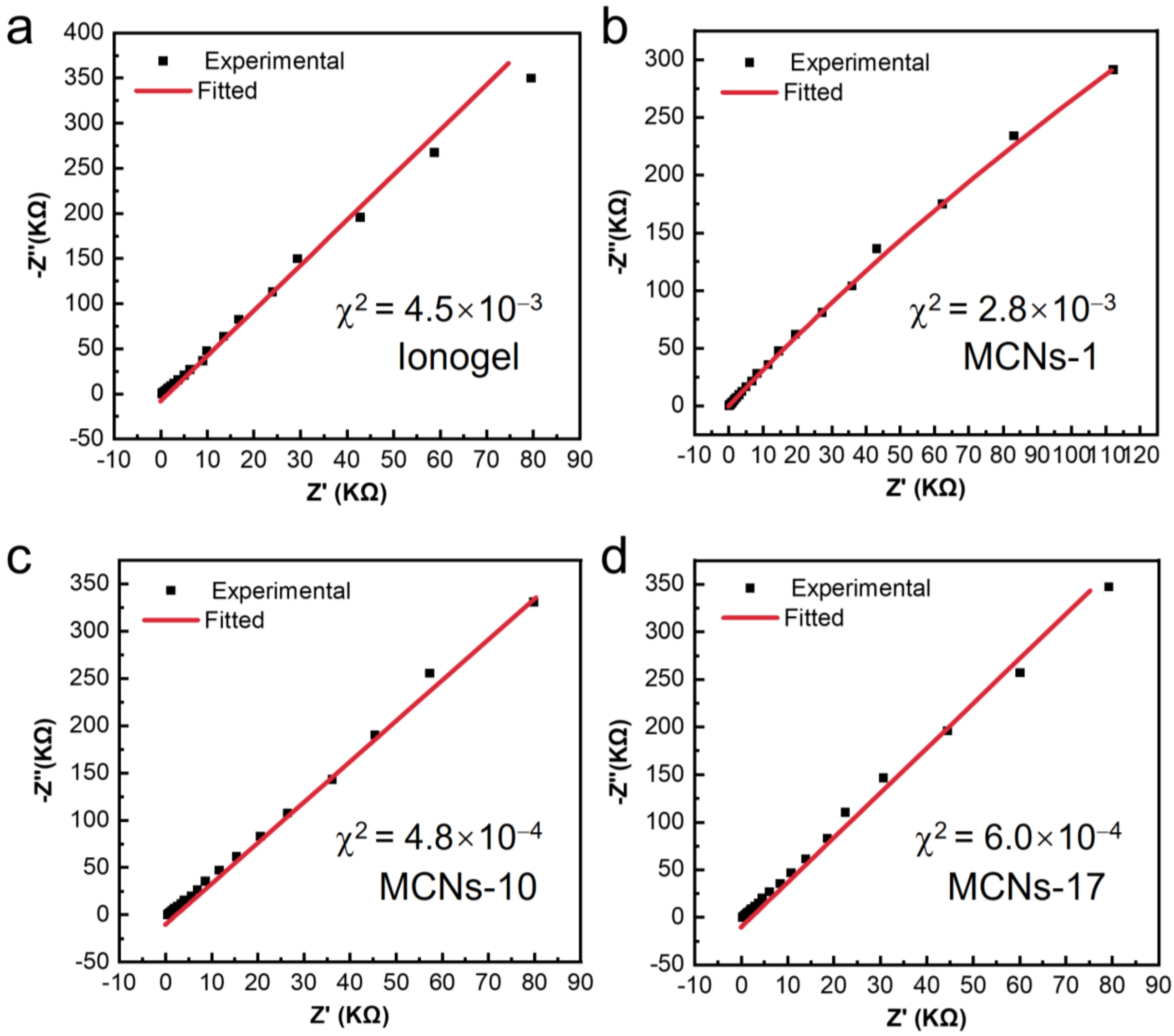

Supplementary Fig. 42. Fitting of Nyquist plots with an equivalent circuit for MCNs-x ionogels. (a) Pure ionogel, (b) MCNs-1 ionogels, (c) MCNs-10 ionogels and (d) MCNs-17 ionogels. The experimental EIS spectra were fitted using the equivalent circuit R_{in}–(R_{ex} ‖ CPE) with the impedance function is $Z(\omega) = R_{in} + [R_{ex}^{-1} + Q(j\omega)^n]^{-1}$, where R_{in} and R_{ex} represent the ionic and electronic resistances, respectively, Q and n are the CPE coefficient and exponent, j is the imaginary unit, and $\omega=2\pi f$. The corresponding χ^2 values obtained from the ZView fitting are indicated in each panel.

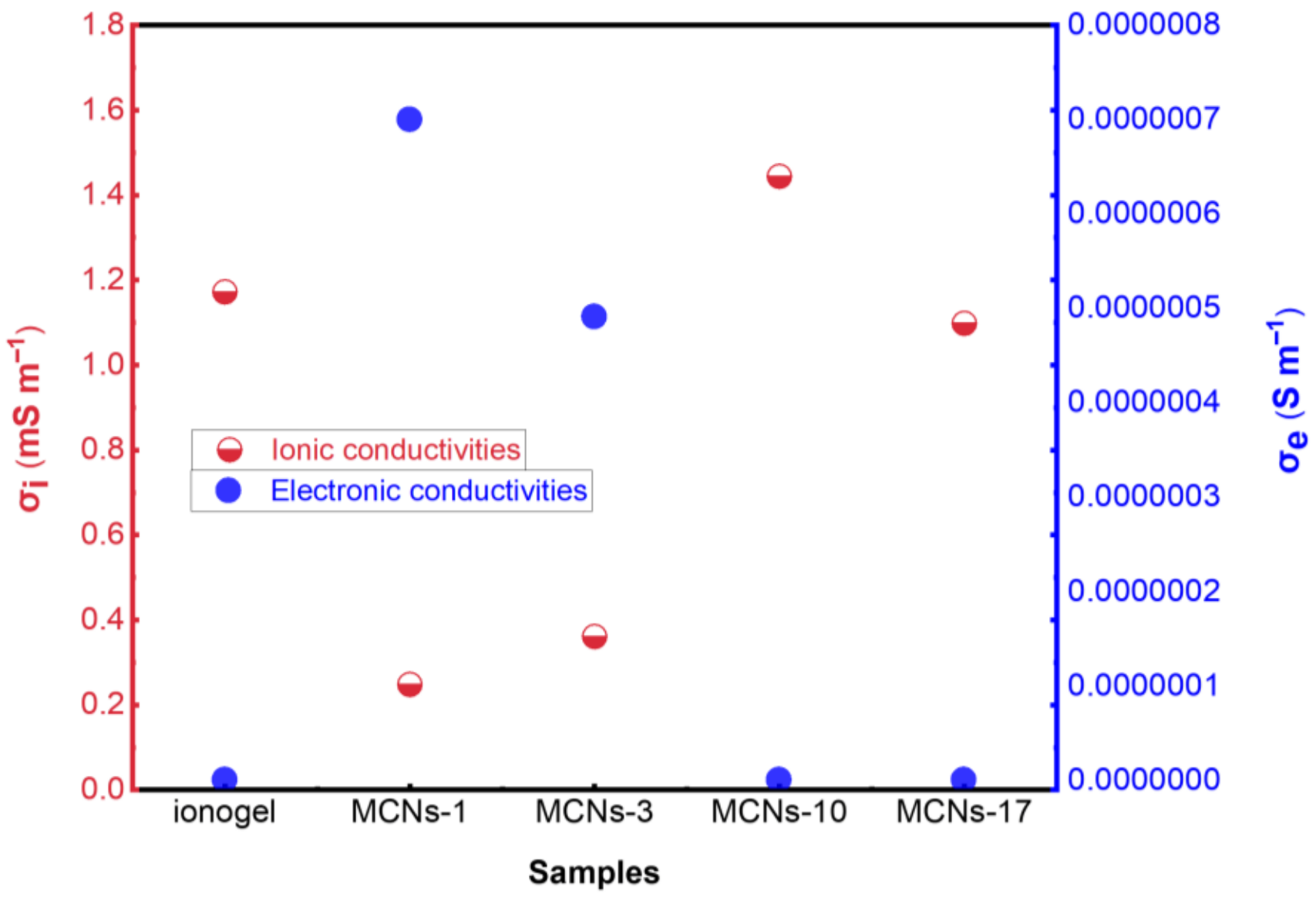

Supplementary Fig. 43. Variations of the ionic and electronic conductivities of MCNs ionogels.

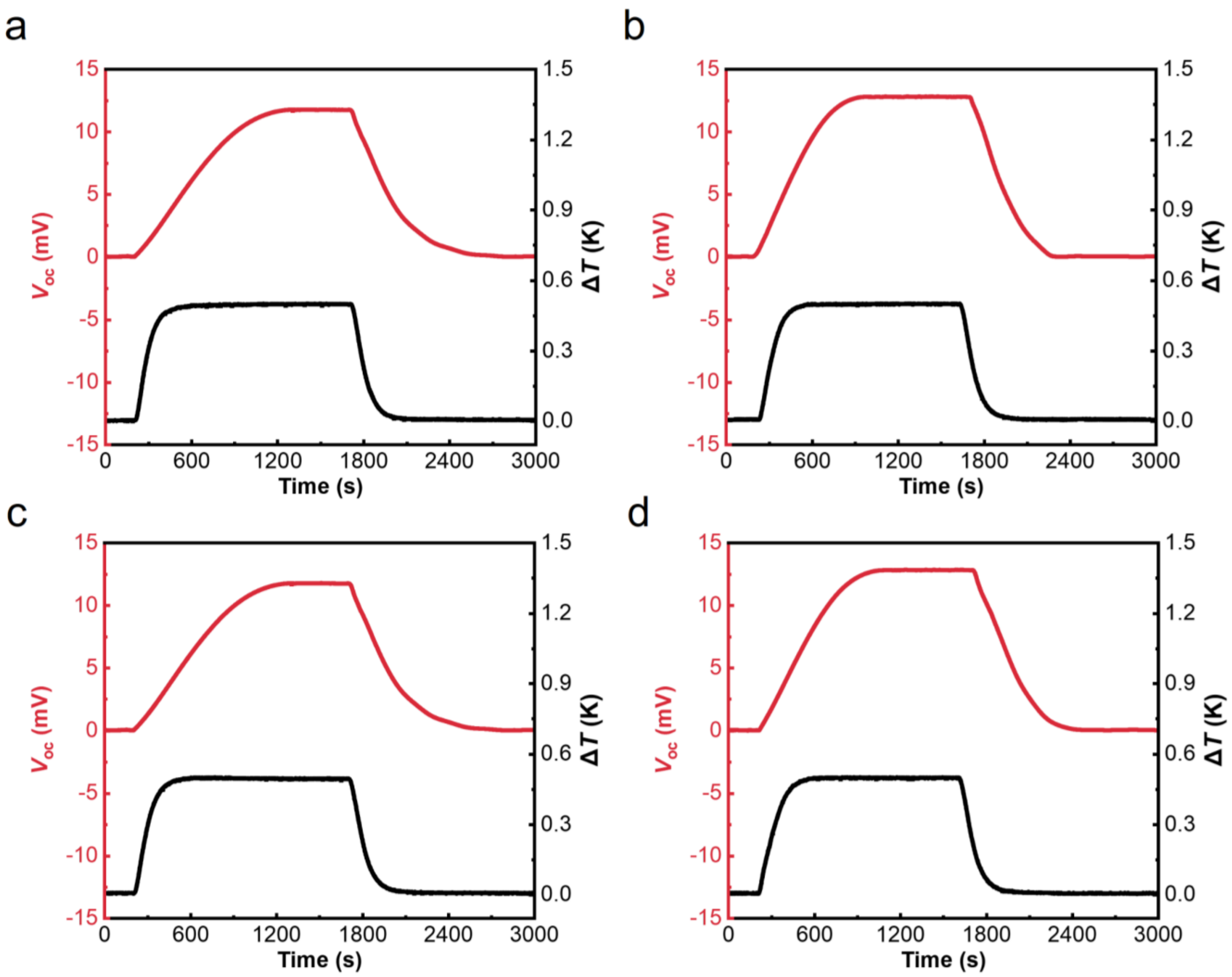

Supplementary Fig. 44. Open-circuit voltage (V_{oc}) profiles of ionogels with different mesostructured MCNs under the temperature gradient of about 0.5 K. (a) Without addition of MCNs. (b) MCNs-1, ordered mesoporous carbon nanofibers. (c) MCNs-10, golf ball-like nanospheres. (d) MCNs-17, multichambered nanospheres. The heating rate is 0.4 K min^{-1}, and the relative humidity (RH) is 70%.

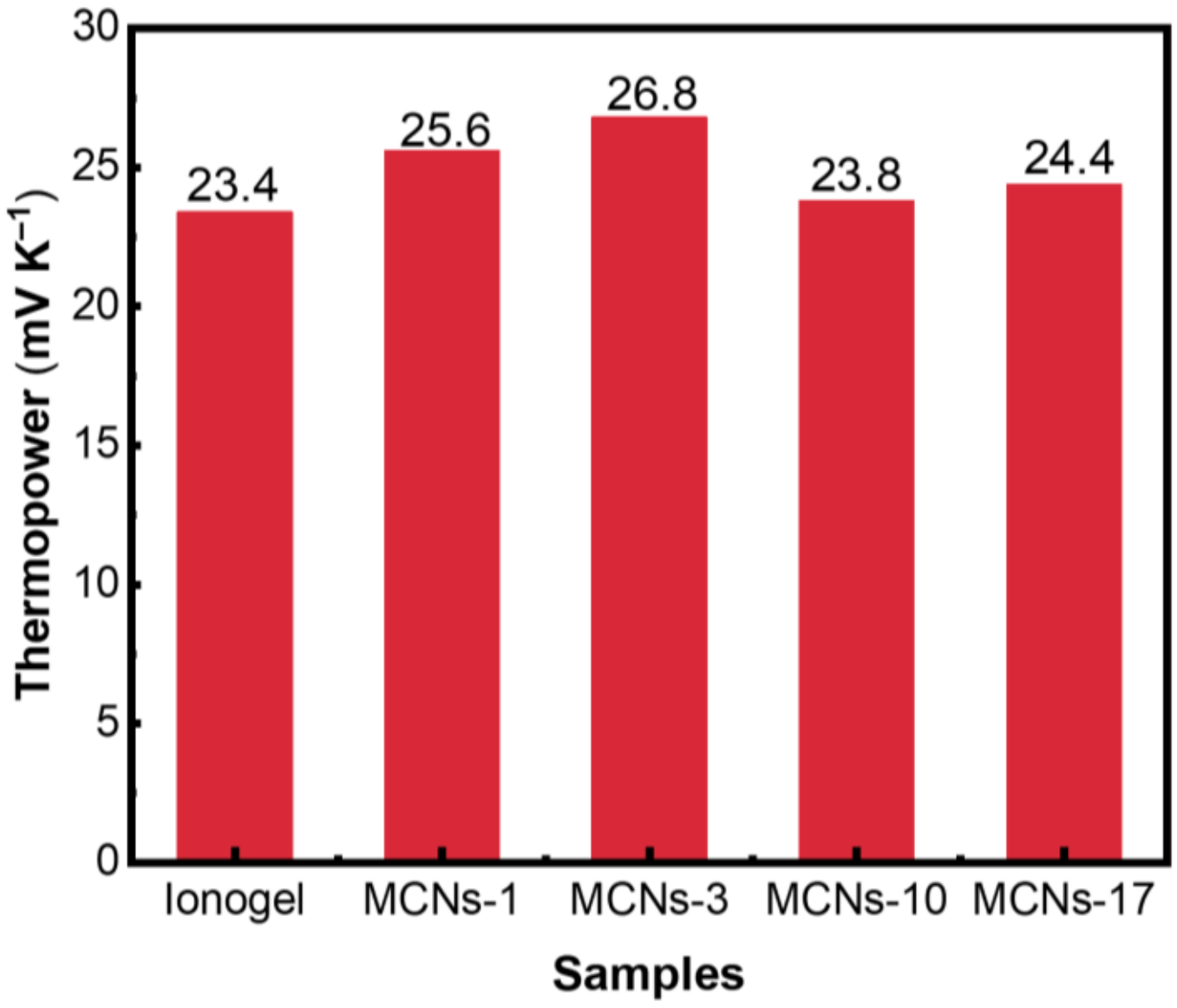

Supplementary Fig. 45. Thermopower of MCNs-x ionogels under the temperature gradient of 0.5 K without external loading. The results indicate that when a temperature gradient of 0.5 K was applied, the MCNs-3 exhibited the highest thermopower of 26.8 mV K^{-1}.

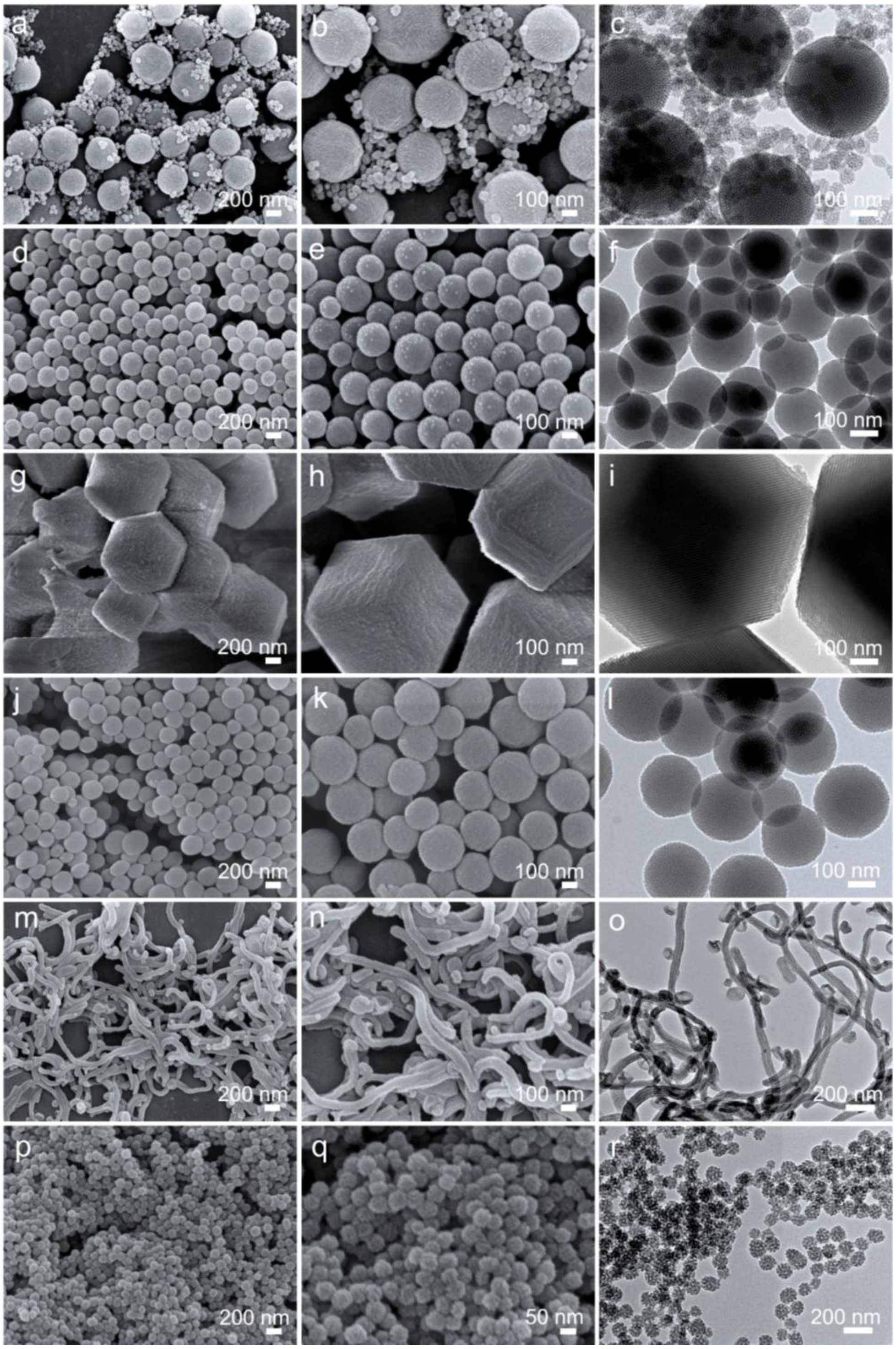

Supplementary Fig. 46. SEM and TEM images of MCNs prepared with similar process of hollow nanofibers (a–c, g–i and m–o) and multichambered nanospheres (d–f, j–l and p–r). The prepared process was performed similarly except (a–f) without adding melamine and TOP, (g–l) without adding TOP and (m–r) without adding melamine.

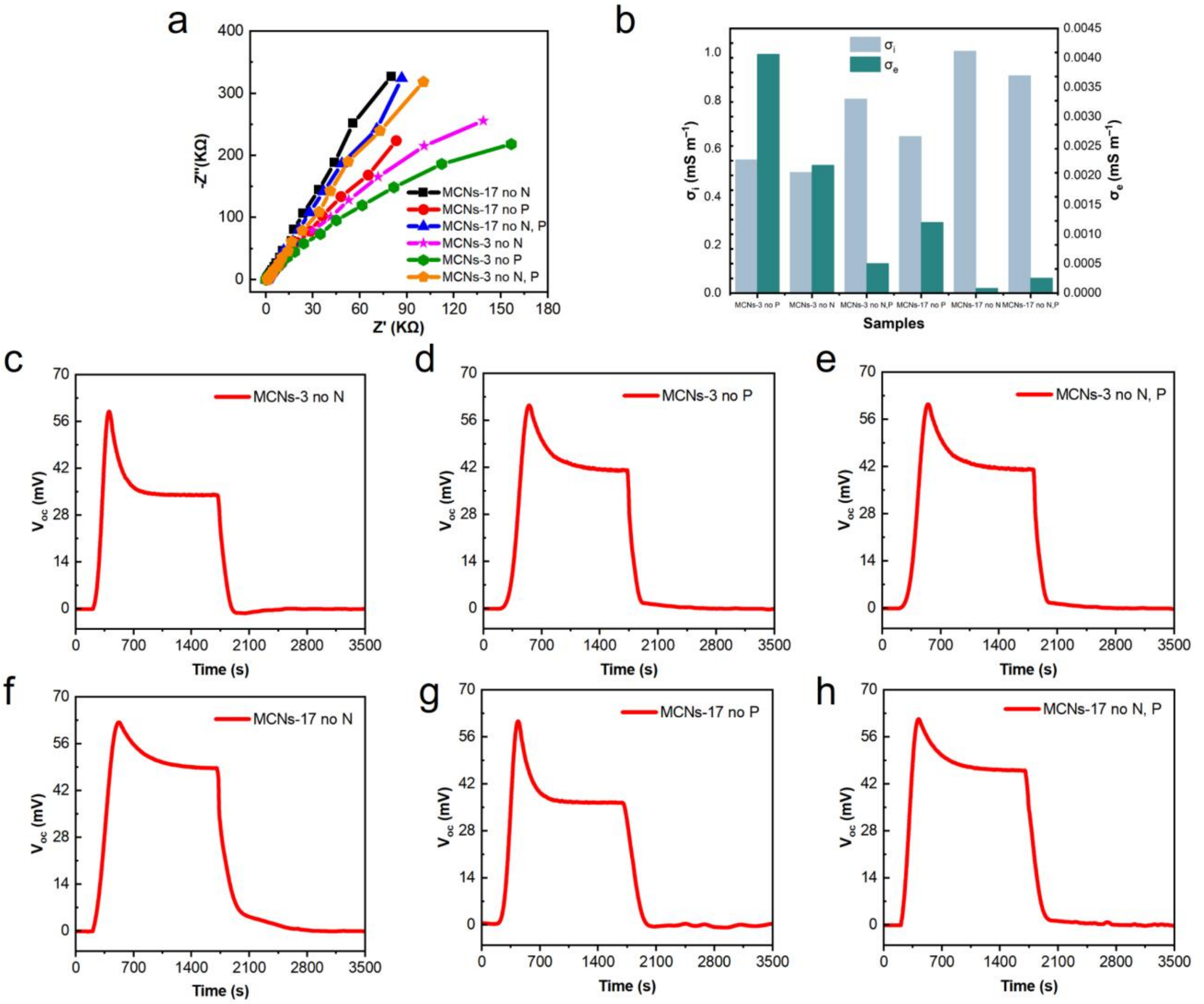

Supplementary Fig. 47. Electrochemical performance of the samples in N/P control experiments.

(a) Nyquist plots. (b) Variations of the ionic and electronic conductivities of MCNs ionogels. (c–h) Open-circuit voltage (V_{oc}) response under the temperature of 3 K. The heating rate is 0.4 K min^{-1}, and the relative humidity (RH) is 70%.

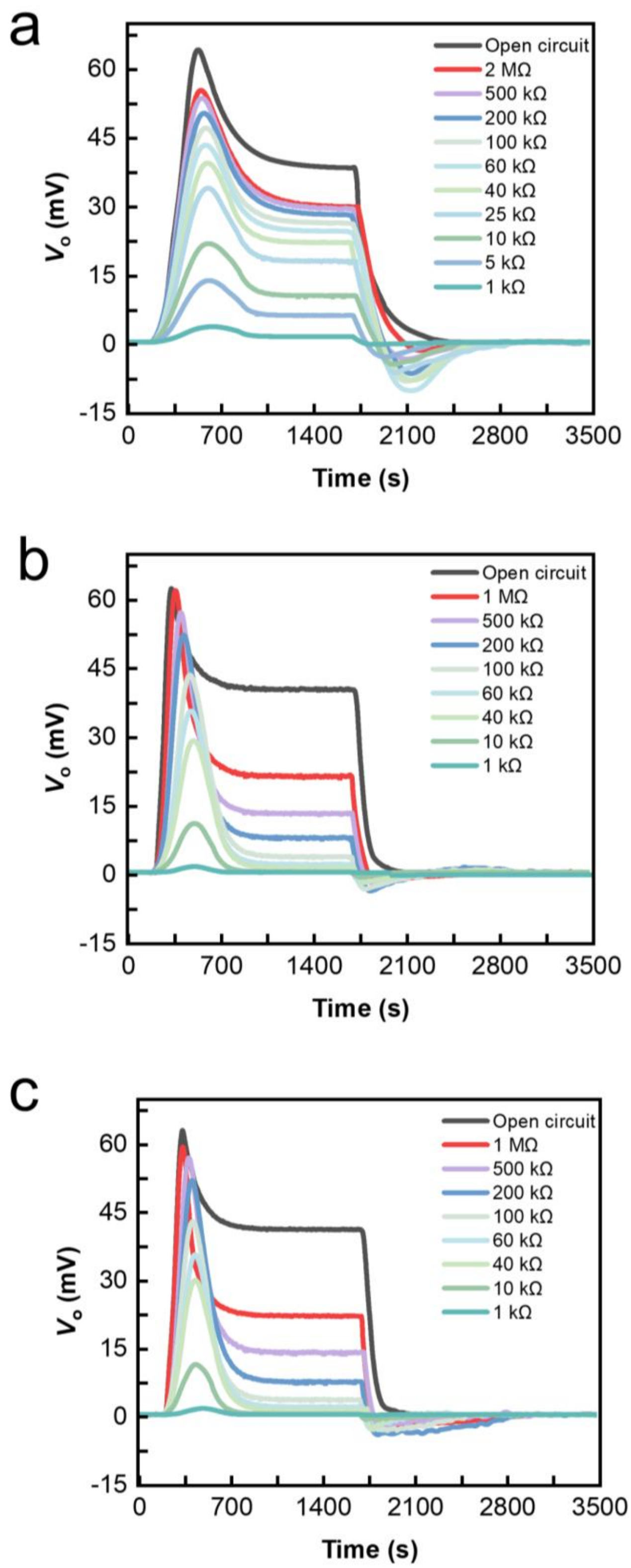

Supplementary Fig. 48. Output voltage profiles (V_o) on the external load connected to an MTEG with various MCNs loading. (a) MCNs-1, (b) MCNs-10, (c) MCNs-17. The temperature gradient is 3 K. External resistances are indicated.

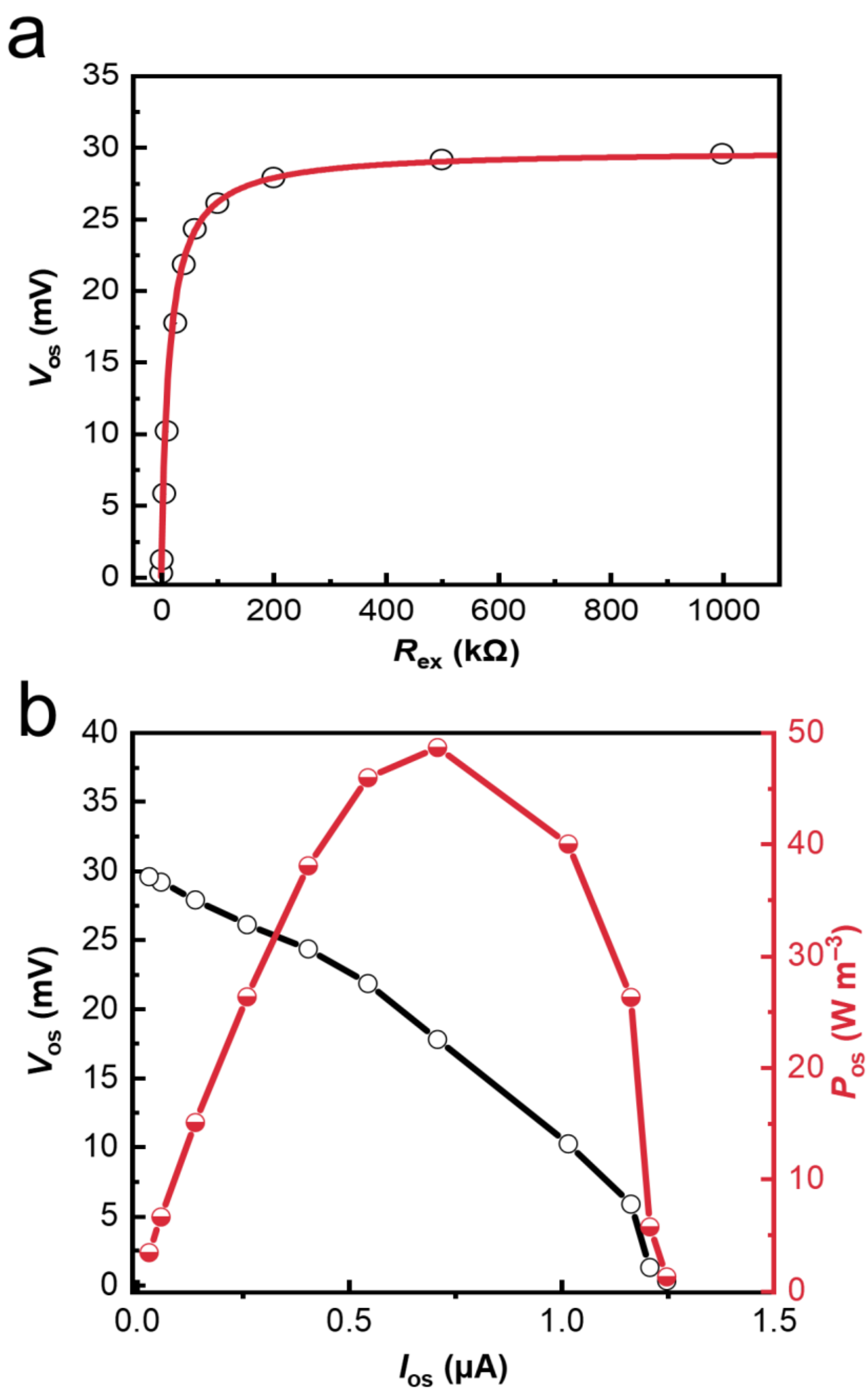

Supplementary Fig. 49. Performance of MTEGs with MCNs-1 ionogels under a steady temperature gradient. (a) Variations of the steady output voltage with external resistance (R_{ex}) for MTEGs. (b) Dependences of steady-state output voltage profile on the external load (V_{os}) and steady power density (I_{os}) on steady current for the MTEGs.

Supplementary Table 2. Comparison of the steady thermovoltage of the ionogels with the MCNs-3 loading of 0.3 wt% in this work with the thermopowers (*S*) of some representative mixed ion-electron Conductor materials, electronic materials, inorganic ionic materials, ionogels, at room temperature.

Materials	Steady-State Thermopower/ (mV/K)	Continuous power supply	External Load Resistance (kΩ)	Reference
**Mixed ion-electron Conductor materials**				
MCNs-3/ionogel	12.33	yes	40	This work
CNT/ionogel	0.05	yes	1	[10]
rGO/ionogel	7	yes	25	[11]
**Electronic TE materials**				
Bi_2Te_3	0.21	yes	-	[12]
PbTe	0.33	yes	-	[13]
Sb/Sb_2Te_3	0.54	yes	-	[14]
Iodine-doped Pentacene	0.07	yes	-	[15]
PEDOT: Tos/Au	0.021	yes	-	[16]
Lignin/PAN-derived electrospun carbon nanofibres	0.01	no	-	[17]
**Inorganic ionic materials**				
$RuIn_3$	0.4	no	-	[18]
$IrIn_3$	0.02	no	-	[18]
$LaCoO_3$	0.5	no	-	[19]
CdS	-0.14	no	-	[20]
Cu_2S	0.15	no	-	[20]
MnS	0.3	no	-	[20]
**Ionogels**				
PVA/IL ionic hydrogels	-	no	2	[21]
NaCl–PMSC Ionogel	-	no	-	[22]
PEO-IL Ionogels	-	no	200	[23]
Cellulose-based ionogel	-	no	0.01	[24]
Cellulose-based ionogel	6	yes	-	[25]

CNT: Carbon Nanotubes; rGO: Reduced Graphene Oxide; PEDOT:Tos: Poly(3,4-ethylenedioxythiophene):p-toluenesulfonate; PVA: Poly(vinyl alcohol); PMSC: Poly(methanesulfonic acid cellulose); PEO: Poly(ethylene oxide); PAM-P407: Polyacrylamide-Poloxamer 407.

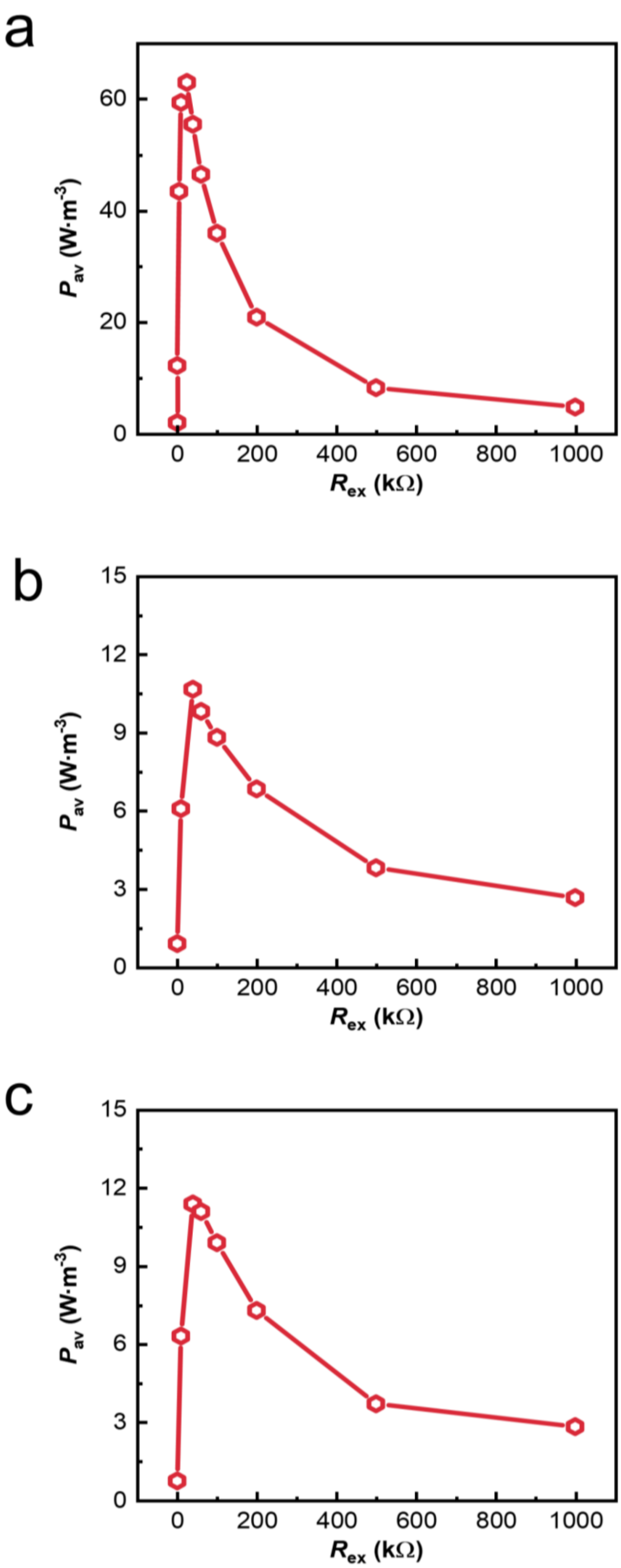

Supplementary Fig. 50. Variations of the average power density with external resistance for the external load connected to MTEGs with the loadings of (a) MCNs-1, (b) MCNs-10, (c) MCNs-17.

Supplementary Table 3. Explanation of key terms and abbreviations used in the CG simulations.

Term/abbreviation	Meaning in this work	Use and interpretation
F127	Pluronic F127, a PEO-PPO-PEO triblock copolymer.	Soft-template polymer in precursor self-assembly.
F127M	Residue/model name of the Martini CG representation of F127 in topology and coordinate files.	Not a new chemical component; discussion can refer to F127.
PEO	Hydrophilic poly(ethylene oxide) blocks of F127, represented by E-type beads.	Forms the polar corona and interacts with water, ETHX and hydroxyl-rich LW sites.
PPO	Hydrophobic poly(propylene oxide) block of F127, represented by P-type beads.	Forms the hydrophobic domain and is preferentially anchored by TOP-rich regions.
TOP	Hydrophobic TOP component in the experimental formulation and CG model.	Forms TOP-rich domains/slabs and tunes the hydrophobic interfacial environment.
EtOH/ETHX	EtOH denotes ethanol in the experiment; ETHX denotes the Martini CG ethanol bead.	Represents ethanol-related polar solvent effects in the CG models.
W	Martini water bead; one W bead represents approximately four water molecules.	Aqueous solvent in all CG models.
LW	Liquefied wood, treated as a polyhydroxyl-containing phenolic precursor/carbon source.	General term for precursor chemistry and interfacial role.
LWOH	Residue/model name of the CG representation of LW containing aromatic carbon-rich beads and hydroxyl-rich polar beads.	Used in topology/coordinate files and bead-level contact analysis; equivalent to the modeled LW component.
LW-C	Aromatic/carbon-rich beads of the CG LW model.	Hydrophobic/aromatic contact contributions, e.g., LW-C-TOP and LW-C-PPO.
LW-OH	Hydroxyl-rich polar beads of the CG LW model.	Polar contact contributions, e.g., LW-OH-PEO and LW-OH-ETHX.

P-series models	Formulation-guided CG models including P3-highEtOH, P1-base and P2-highTOP.	Compare local packing tendency and precursor morphology under different EtOH/TOP contents.
Ea-series models	Actual-ratio long-box TOP-rich/water interfacial models.	Validate interfacial localization, PPO anchoring and anisotropic organization.
TOP-rich slab	Continuous TOP-rich interfacial domain identified in the long-box models.	Stability is evaluated by TOP cluster number and largest TOP-cluster fraction.
Aggregate	F127/LW-containing precursor aggregate or selected bead group used for shape/orientation analysis.	Evaluates elongation, anisotropy and interfacial orientation.
C_A-B or N(A-B)	Number of bead-bead contacts between groups A and B within the selected cutoff.	C or N denotes contact count, not carbon; contacts/bead is normalized by bead number.
p_{local}	Contact-derived local packing proxy normalized relative to P1-base.	Relative descriptor of local packing tendency, not an absolute classical packing parameter.
p_{eff}	Convex-hull-based aggregate-scale compactness descriptor.	Supplementary geometric support for aggregate compactness.
axis1/axis3	Axis ratio calculated from eigenvalues of the gyration tensor.	Higher values indicate a more elongated aggregate or chain configuration.
kappa2 (κ^2)	Relative shape anisotropy calculated from the gyration tensor.	Higher values indicate stronger deviation from an isotropic spherical shape.
S	Orientation parameter calculated relative to the interface normal.	S=-0.5 ideal in-plane alignment; S=0 random; S=1 perpendicular alignment.

Supplementary Table 4. Formulation mapping, coarse-grained composition and key morphology descriptors of P3-highEtOH, P1-base and P2-highTOP systems.

Model	P3-highEtOH	P1-base	P2-highTOP
Experimental condition	TOP=0.1 mL, EtOH=25 mL	TOP=0.1 mL, EtOH=10 mL	TOP=0.3 mL, EtOH=10 mL
F127M	16	16	16
TOP	40	40	120
ETHX	22500	9000	9000
LWOH	120	120	120
W	36538	36538	36538
Calibrated p_{local}	0.295 ± 0.019	0.400 ± 0.010	0.538 ± 0.024
axis1/axis3	1.217 ± 0.071	1.352 ± 0.079	1.400 ± 0.073
κ^2	0.0167 ± 0.0092	0.0284 ± 0.0098	0.0447 ± 0.0174

Normalized p_{local} was obtained by normalizing the contact-derived local packing proxy to P1-base and assigning P1-base a reference value of 0.40. The axis1/axis3 and κ^2 values describe the shape anisotropy of the largest precursor core. All values are reported as mean ± standard deviation over the last three representative frames.

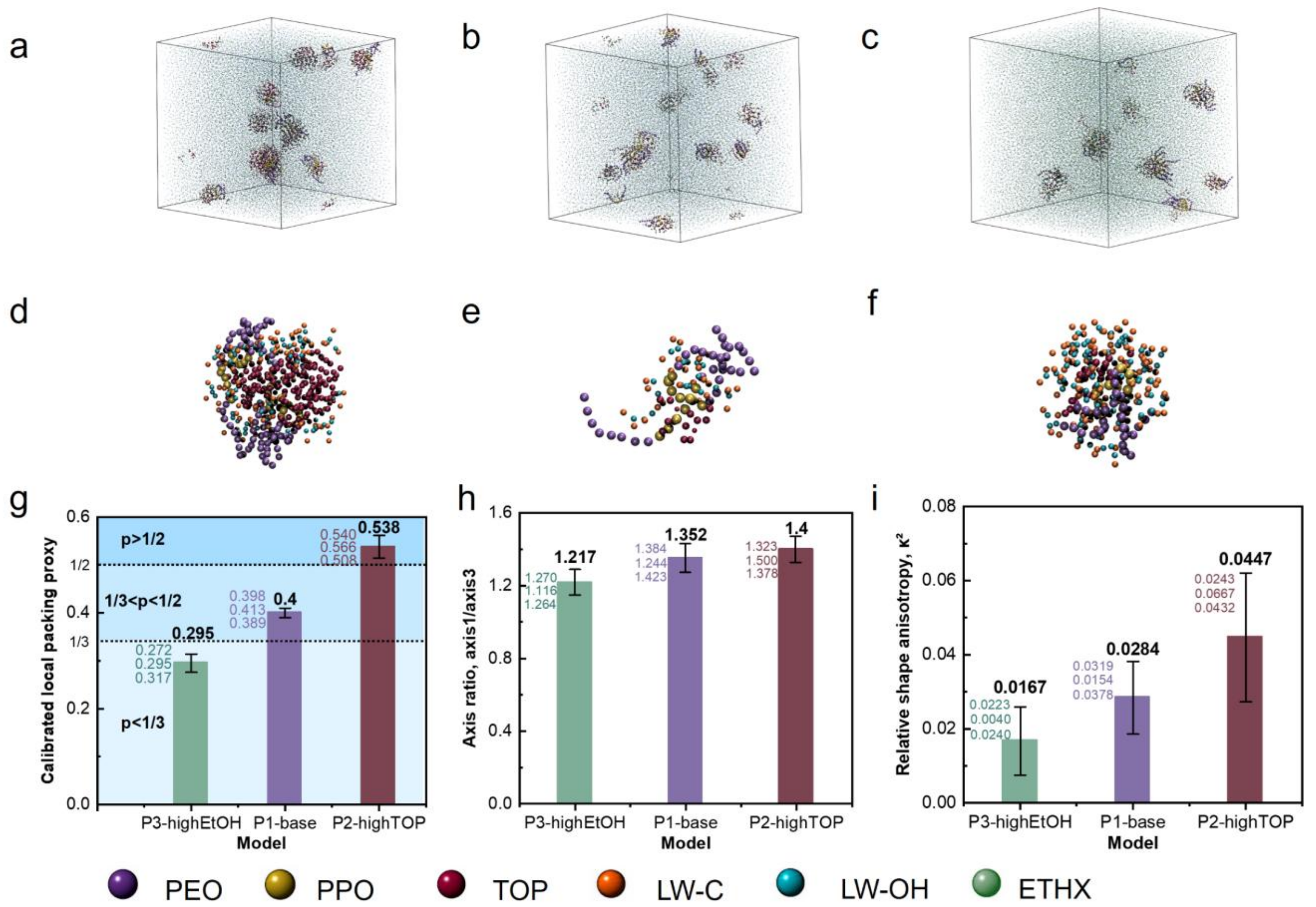

Supplementary Fig. 51. CG simulation of composition-dependent local packing tendency and precursor morphology evolution. (a–c) Representative full-system snapshots of P3-highEtOH, P1-base and P2-highTOP, respectively. Water molecules were omitted for clarity. (d–f) Representative local precursor aggregates extracted from the corresponding systems. (g) Normalized contact-derived packing-tendency proxy, p_{local}. The shaded background regions schematically indicate the commonly discussed classical packing-parameter ranges only for qualitative reference; the plotted p_{local} values should not be interpreted as absolute classical p values or strict regime assignments. (h) Axis ratio axis1/axis3 of the largest precursor core. (i) Relative shape anisotropy κ^2 of the largest precursor core. Error bars represent the standard deviation over the last three representative frames. Individual points represent values calculated from three trajectory frames sampled at cumulative simulation times of 10.0, 12.5 and 15.0 ns. Bars indicate the mean, and error bars indicate the standard deviation (SD; n=3 sampled trajectory frames per system). The sampled frames are temporal observations from a single production trajectory.

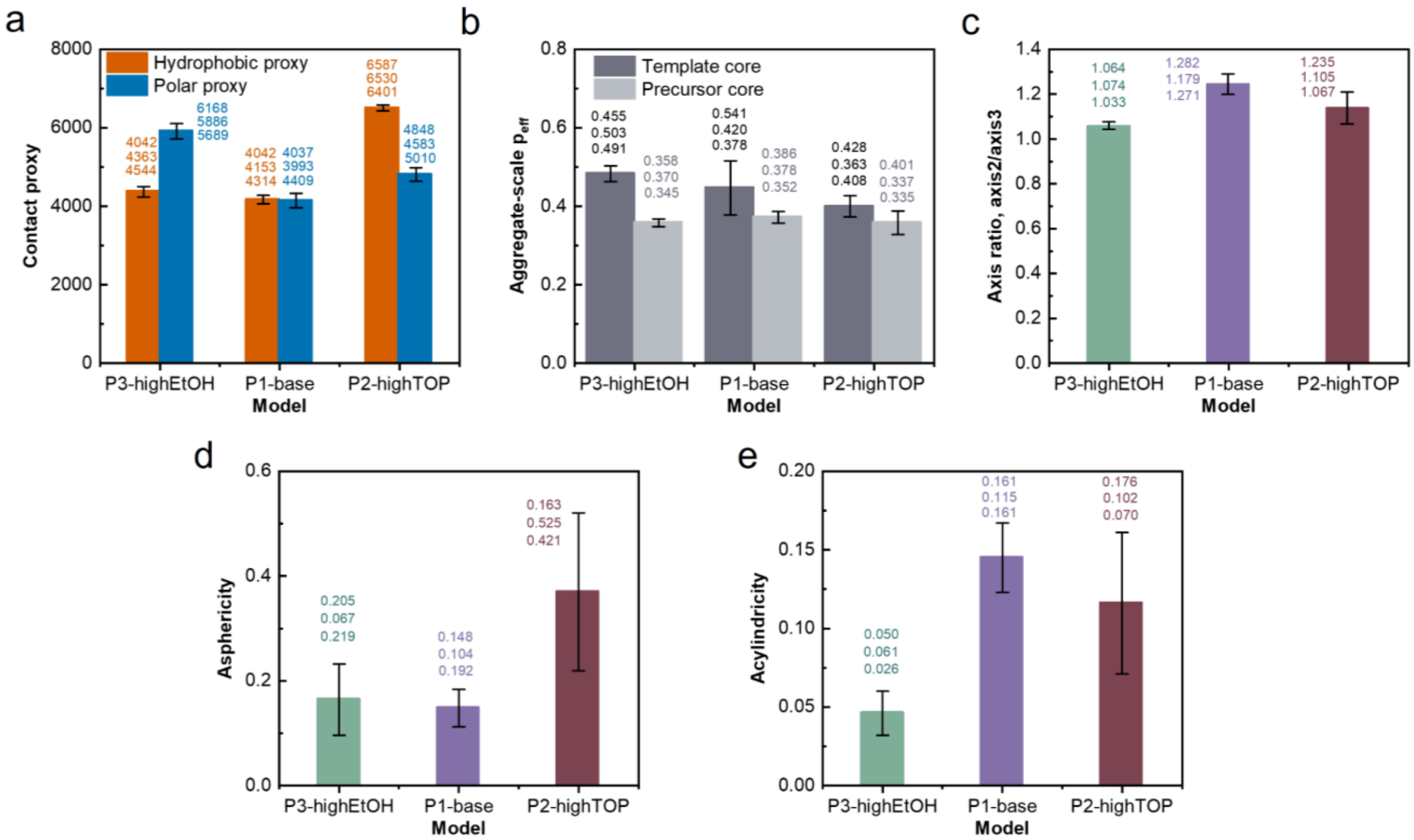

Supplementary Fig. 52. Supplementary quantitative analysis of contact-derived packing contributions, aggregate-scale compactness descriptors and additional shape parameters in P3-highEtOH, P1-base and P2-highTOP systems. (a) Hydrophobic and polar contact proxies are used to define the local packing tendency. The hydrophobic proxy was calculated from LWOH-C–TOP, LWOH-C–PPO and TOP–PPO contacts, whereas the polar proxy was calculated from LWOH-OH–PEO, LWOH-OH–ETHX and TOP–PEO contacts. (b) Convex-hull-based aggregate-scale compactness descriptors, p_{eff}, were calculated for template and precursor cores. The template core was defined as PPO+TOP, while the precursor core was defined as PPO+TOP+LWOH-C. (c–e) Supplementary shape descriptors of the largest precursor core, including axis2/axis3, asphericity and acylindricity. Error bars represent the standard deviation over the last three representative frames. Individual points represent values calculated from three trajectory frames sampled at cumulative simulation times of 10.0, 12.5 and 15.0 ns. Bars indicate the mean, and error bars indicate the standard deviation (SD; n = 3 sampled trajectory frames per system). The sampled frames are temporal observations from a single production trajectory.

Supplementary Table 5. Quantitative descriptors from actual-ratio long-box TOP-rich/water interfacial CG simulations.

Descriptor	$Ea1_{TOP40}$	$Ea2_{TOP120}$
$n_{TOP\ clusters}$	1.0	1.0
Largest TOP-cluster fraction	1.0	1.0
PEO-TOP contacts/bead	0.507	0.974
PPO-TOP contacts/bead	2.270	8.484
LWOH-TOP contacts/bead	1.220	3.477
PEO-water contacts/bead	3.438	3.309
PPO-water contacts/bead	1.327	0.081
LWOH-water contacts/bead	2.728	3.314
TOP z_{mean} (nm)	35.63	35.96
PEO z_{mean} (nm)	34.69	36.17
PPO z_{mean} (nm)	34.71	36.15
LWOH z_{mean} (nm)	34.01	36.46
Aggregate axis1/axis3	1.96 ± 0.17	2.30 ± 0.26
Aggregate κ^2	0.141 ± 0.028	0.272 ± 0.072
Aggregate S	-0.275 ± 0.075	-0.476 ± 0.031
F127 axis1/axis3	2.87 ± 0.18	4.08 ± 0.57
F127 κ^2	0.398 ± 0.069	0.446 ± 0.063
F127 S	-0.071 ± 0.095	-0.441 ± 0.093

The values were averaged over the final 2 ns of the 20 ns production simulations. The normalized contact density was calculated by dividing the total number of contacts by the number of corresponding beads. The TOP-rich slab was identified using a 0.8 nm cutoff for TOP bead clustering. The descriptors listed in Supplementary Table 5 correspond to the structural snapshots, density profiles, contact-density plots and shape/orientation analyses shown in Supplementary Fig. 53.

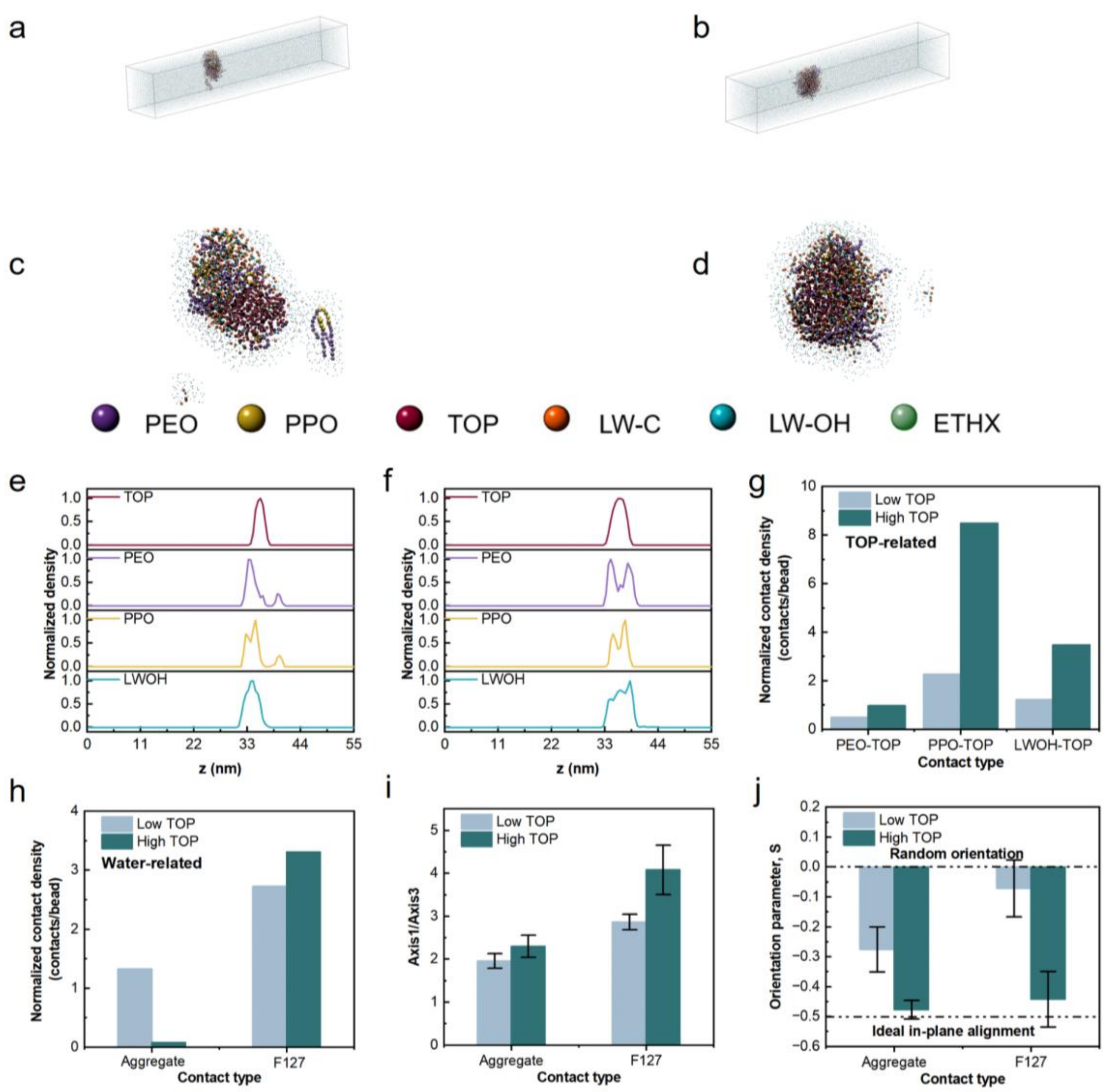

Supplementary Fig. 53. Actual-ratio long-box TOP-rich/water interfacial CG simulations validating TOP-content-dependent interfacial localization and anisotropic organization. (a, b) Representative full-system snapshots of $Ea1_{TOP40}$ and $Ea2_{TOP120}$, respectively. (c, d) Local interfacial views of the corresponding systems. Water beads were omitted or shown only as a dilute background for clarity. The representative CG snapshots shown in Supplementary Fig. 53a-d are consistent with the quantitative descriptors summarized in Supplementary Table 5, showing stronger interfacial coupling in $Ea2_{TOP120}$ than in $Ea1_{TOP40}$. (e, f) z-directional normalized density profiles of TOP, PEO, PPO and LWOH in $Ea1_{TOP40}$ and $Ea2_{TOP120}$. (g) Normalized contact densities between PEO, PPO or LWOH and TOP. (h) Normalized water-contact densities. (i) Axis1/axis3 ratios of the F127/LWOH aggregate and F127 chains. (j) Orientation parameter S of the aggregate and F127 chains relative to the normal interface. S=-0.5 indicates ideal in-plane alignment, S = 0 indicates random orientation and S=1 indicates perpendicular alignment. Error bars represent the standard deviation calculated over the final 2 ns of the corresponding production trajectories. Individual points represent values calculated from ten trajectory frames sampled over the final 2 ns of the corresponding production trajectory. Bars indicate the mean, and error bars indicate the standard deviation (SD; n = 10 sampled trajectory frames per system). The sampled frames are temporal observations from a single production trajectory.

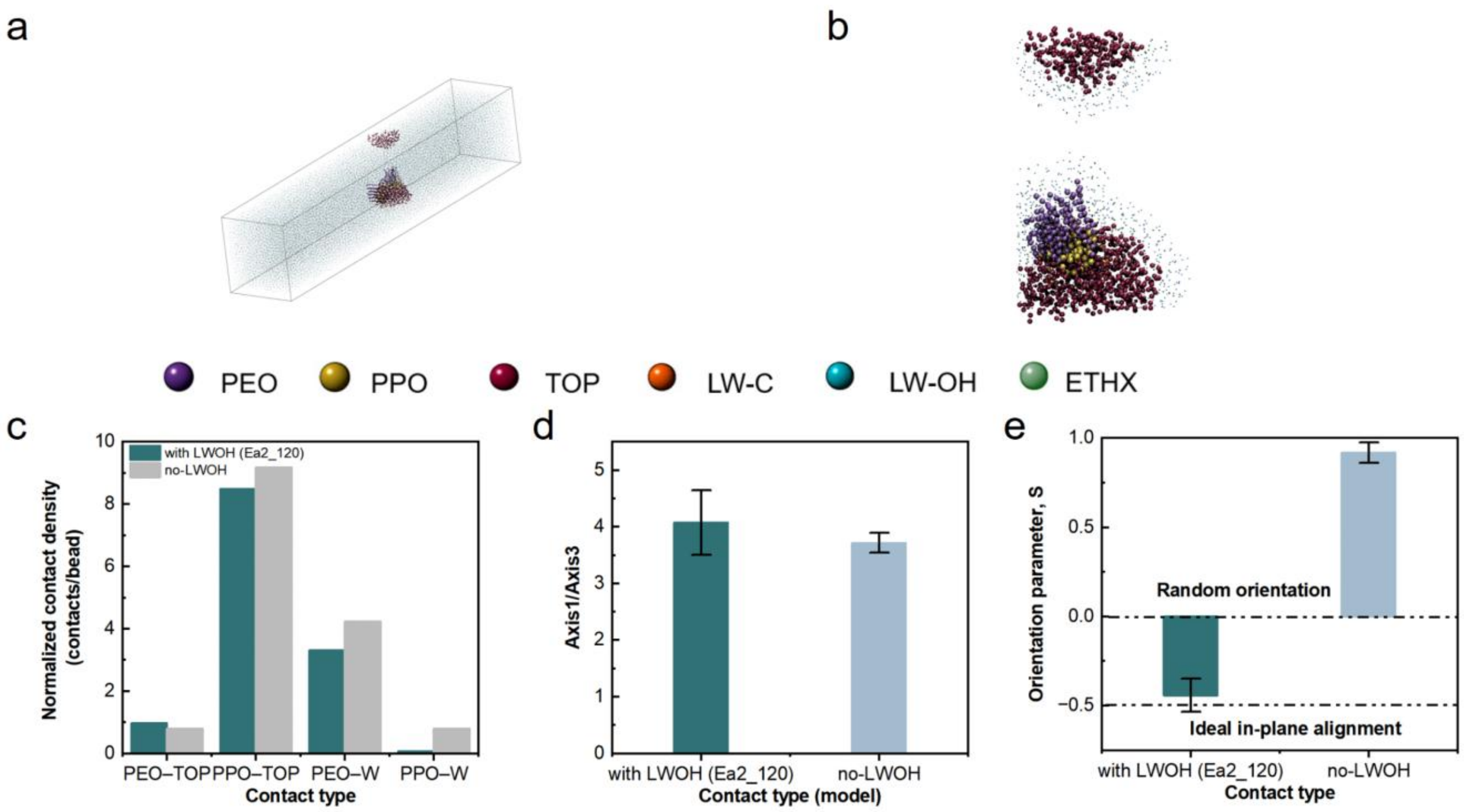

Supplementary Fig. 54. LWOH-removal control simulation clarifying the functional role of LW in TOP-rich/water interfacial organization. (a, b) Representative final configurations of the LWOH-containing TOP120 model and the LWOH-removal model, respectively. In the LWOH-removal model, LWOH was removed from the equilibrated TOP120 interfacial configuration and the trajectory was continued to examine the retained interfacial organization. Water and ETHX beads are omitted for clarity. (c) Normalized contact densities of PEO–TOP, PPO–TOP, PEO–water and PPO–water. LWOH removal retains F127–TOP contacts but increases the water exposure of F127, especially the PPO segment. (d) F127 axis1/axis3 ratio in the LWOH-containing and LWOH-removal models. (e) F127 orientation parameter S relative to the normal interface. S = -0.5 corresponds to ideal in-plane alignment, S = 0 to random orientation and S = 1 to perpendicular alignment. Error bars represent the standard deviation over the final 2 ns of the corresponding production trajectories. Individual points represent values calculated from ten trajectory frames sampled over the final 2 ns of the corresponding production trajectory. Bars indicate the mean, and error bars indicate the standard deviation (SD; n = 10 sampled trajectory frames per system). The sampled frames are temporal observations from a single production trajectory.

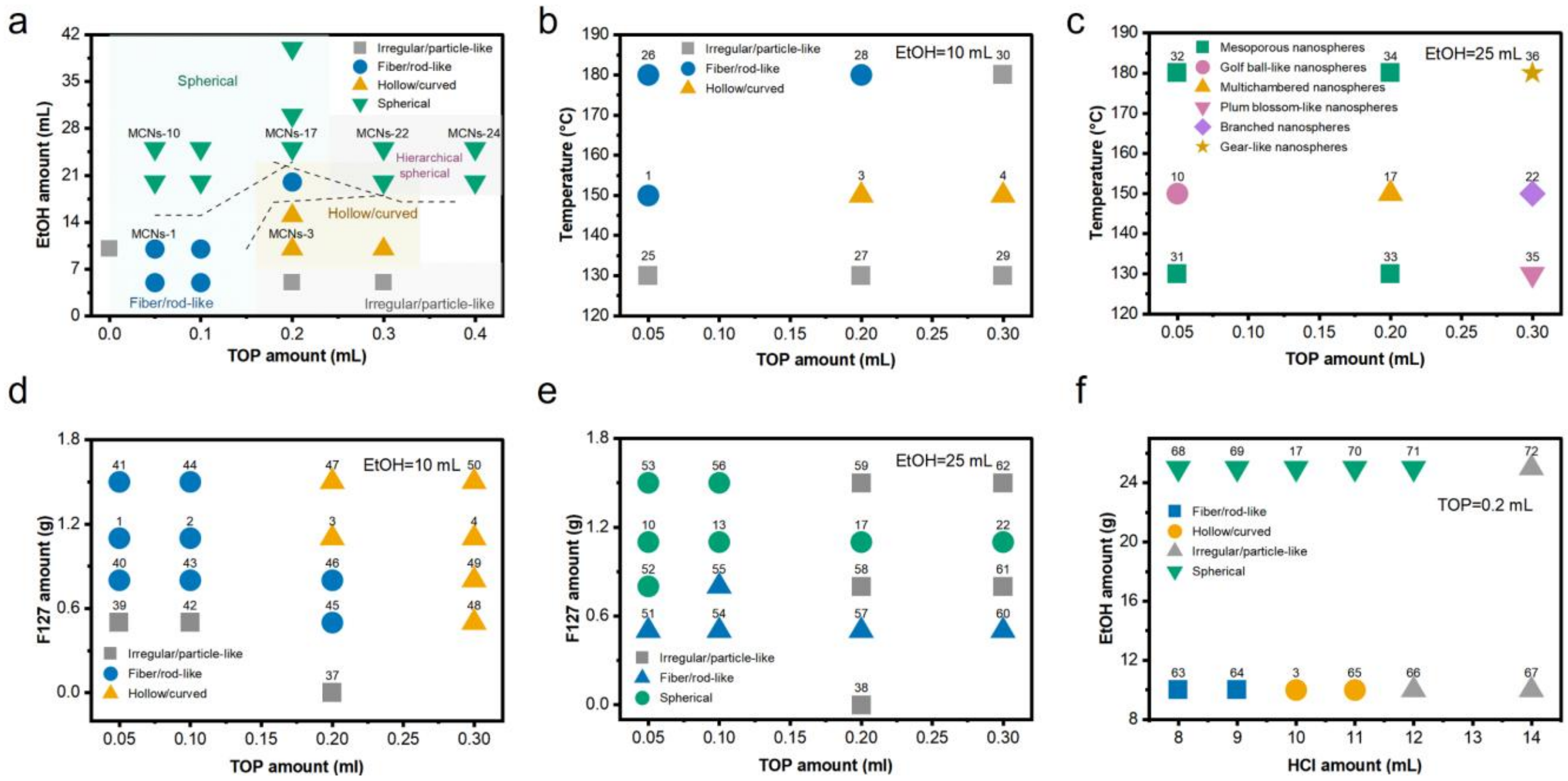

Supplementary Fig. 55. Experimental formulation-morphology map of MCNs. (a) TOP-EtOH morphology map,(b) TOP-temperature map at EtOH = 10 mL, (c) TOP-temperature map at EtOH = 25 mL, (d) TOP-F127 map at EtOH = 10 mL, (e) TOP-F127 map at EtOH = 25 mL, (f) HCl-EtOH map at TOP = 0.2 mL. Each point represents an experimentally synthesized sample, and colors denote different morphology categories. Dashed boundaries indicate approximate transition zones inferred from adjacent experimental points rather than thermodynamic phase boundaries.

Supplementary Table 6. Summary of carbon nanoarchitecture and mixed ion-electron thermoelectric performance.

Samples	MCNs-1	MCNs-3	MCNs-10	MCNs-17
Key structural features	1D fibrous framework with ordered parallel mesochannels	1D hollow fibers with mesoporous shell and internal cavity	nanospheres with local cavities/rough surfaces	nanospheres with multichambered interiors
BET surface area/ (m^2 g^{-1})	1235	639	583	671
Pore volume/ (cm^3 g^{-1})	0.62	0.90	0.37	0.47
Ionic conductivity/ (S m^{-1})	0.248	0.361	1.44	1.10
Electronic Conductivity/ (S m^{-1})	7.00×10^{-7}	4.91×10^{-7}	6.02×10^{-15}	6.02×10^{-13}
Internal resistance/ (kΩ)	14.01	12.52	2.50	3.29
Thermopower/ (mV K^{-1})	25.6	26.8	23.8	24.4
Maximum P_{av}/ (W m^{-3})	63.01	68.32	10.67	11.38
Structure-property relationship	1D ordered framework enables efficient electronic percolation, while ordered mesochannels provide directional ion transport.	Hollow 1D architecture integrates continuous electronic pathways with enhanced ion-accessible interfaces, enabling balanced ion-electron transport.	High ion-accessible surface promotes ionic polarization, but discontinuous particle contacts limit electronic transport.	Reduced size and multichambered structure facilitate ion diffusion, yet insufficient electronic percolation restricts charge compensation.

Supplementary References

1. Wu, Z. et al. Kinetics-controlled synthesis of ordered mesoporous carbon single crystals from liquefied wood. *Adv. Funct. Mater.* **33**, 2213852 (2023).
2. Wu, Z. et al. Ordered mesoporous carbons from liquefied wood: morphological effects of nucleation and growth processes. *Chem. Eng. Sci.* **264**, 118094 (2022).
3. Li, Y. et al. Worm-like ordered mesoporous carbon from liquefied wood: morphological manipulation by varying hydrothermal temperature. *Aggregate* **5**, e570 (2024).
4. Peng, L. et al. One-dimensionally oriented self-assembly of ordered mesoporous nanofibers featuring tailorable mesophases via kinetic control. *Nat. Commun.* **14**, 8148 (2023).
5. Zhao, Z. et al. Constructing unique mesoporous carbon superstructures via monomicelle interface confined assembly. *J. Am. Chem. Soc.* **144**, 11767–11777 (2022).
6. Zhao, Z. et al. Modular super-assembly of hierarchical superstructures from monomicelle building blocks. *Sci. Adv.* **8**, eabo0283 (2022).
7. Peng, L., Peng, H., Li, W. & Zhao, D. Monomicellar assembly to synthesize structured and functional mesoporous carbonaceous nanomaterials. *Nat. Protoc.* **18**, 1155–1178 (2023).
8. Cheng, H. & Ouyang, J. Ultrahigh thermoelectric power generation from both ion diffusion by temperature fluctuation and hole accumulation by temperature gradient. *Adv. Energy Mater.* **10**, 2001633 (2020).
9. Fan, Z., Li, P., Du, D. & Ouyang, J. Significantly enhanced thermoelectric properties of PEDOT:PSS films through sequential post-treatments with common acids and bases. *Adv. Energy Mater.* **7**, 1602116 (2017).
10. Cheng, H., Yue, S., Le, Q., Qian, Q. & Ouyang, J. A mixed ion-electron conducting carbon nanotube ionogel to efficiently harvest heat from both a temperature gradient and temperature fluctuation. *J. Mater. Chem. A* **9**, 13588–13596 (2021).
11. Qian, Q. et al. A mixed ion/electron thermoelectric generator with ultrahigh steady thermopower by exploring both the hole tunneling and ion accumulations. *Adv. Energy Mater.* **15**, 2404522 (2025).
12. Kimberly, T. Q., Ciesielski, K. M., Qi, X., Toberer, E. S. & Kauzlarich, S. M. High thermoelectric performance in 2D Sb_2Te_3 and Bi_2Te_3 nanoplate composites enabled by energy carrier filtering and low thermal conductivity. *ACS Appl. Electron. Mater.* **6**, 2816–2825 (2024).

13. Martin, J., Wang, L., Chen, L. & Nolas, G. S. Enhanced Seebeck coefficient through energy-barrier scattering in PbTe nanocomposites. *Phys. Rev. B* **79**, 115311 (2009).

14. Zhang, Z., Wu, Y., Zhang, H., Zeng, Z. & Hu, Z. Enhancement of Seebeck coefficient in Sb-rich Sb_2Te_3 thin film. *J. Mater. Sci. Mater. Electron.* **26**, 1619–1624 (2015).

15. Gogoc, S. & Data, P. Organic thermoelectric materials as the waste heat remedy. *Molecules* **27**, 1016 (2022).

16. Petsagkourakis, I. et al. Improved performance of organic thermoelectric generators through interfacial energetics. *Adv. Sci*. **10**, 2206954 (2023).

17. Dalton, N., Lynch, R. P., Collins, M. N. & Culebras, M. Thermoelectric properties of electrospun carbon nanofibres derived from lignin. *Int. J. Biol. Macromol.* **121**, 472–479 (2019).

18. Haldolaarachchige, N. et al. Thermoelectric properties of intermetallic semiconducting $RuIn_3$ and metallic $IrIn_3$. *J. Appl. Phys.* **113**, 083709 (2013).

19. Kumar, A., Kumari, K., Jayachandran, B., Sivaprahasam, D. & Thakur, A. D. Thermoelectric properties of $(1-x)LaCoO_3 \cdot xLa_{0.7}Sr_{0.3}MnO_3$ composite. *J. Alloys Compd.* **749**, 1092–1097 (2018).

20. Hasan, M. N., Wahid, H., Nayan, N. & Mohamed Ali, M. S. Inorganic thermoelectric materials: a review. *Int. J. Energy Res.* **44**, 6170–6222 (2020).

21. Huang, Y. et al. Engineering nanoscale solid networks of ionogel for enhanced thermoelectric power output and excellent mechanical properties. *Chem. Eng. J.* **456**, 141156 (2023).

22. Yang, X. et al. High-performance ionic thermoelectric supercapacitor for integrated energy conversion-storage. *Energy Environ. Mater.* **5**, 954–961 (2022).

23. Zhao, W. et al. Tailoring intermolecular interactions towards high-performance thermoelectric ionogels at low humidity. *Adv. Sci.* **9**, 2201075 (2022).

24. Chen, X. et al. Enhancing the thermoelectric performance of sustainable cellulose-based ionogels through water content regulation. *Small* **21**, 2412336 (2025).

25. Li, X. et al. Balancing mechanical-thermal-electrical properties in cellulose ionogels via crystallization-induced molecular assembly. *Nat. Commun.* **16**, 9970 (2025).
