# Supplementary material for: Programmable mesoporous carbon architectures from liquefied wood via reactive-emulsion-mediated self-assembly
Source: Nat Commun. 2026 Aug 29;17:10307. doi: 10.1038/s41467-026-77269-7 (PMC13619605; doi:10.1038/s41467-026-77269-7)
Supplement: Supplementary file 2 [file 41467_2026_77269_MOESM2_ESM.pdf]

nature portfolio

Peer Review File

Programmable mesoporous carbon architectures from liquefied wood via reactive-emulsion-mediated self-assembly

Corresponding Author: Dr Shouxin Liu

This file contains all reviewer reports in order by version, followed by all author rebuttals in order by version.

Version 0:

Reviewer comments:

Reviewer #1

(Remarks to the Author)
The manuscript reports an emulsion-mediated self-assembly strategy to synthesize mesoporous carbon architectures with tunable morphologies and pore structures from liquefied wood. By varying the amount of trioctyl phosphate (TOP) and ethanol, the authors claim to achieve continuous structural evolution and propose a mechanism based on interfacial energy ($\Delta\sigma$) and micelle packing parameter (p). While the experimental scope is broad and the structural diversity is interesting, the conceptual advance is limited. The mechanistic framework largely relies on established concepts (e.g., packing parameter and interfacial self-assembly), which are used here primarily in a descriptive manner rather than enabling new understanding. In addition, key mechanistic claims are not supported by direct experimental evidence. Overall, the manuscript does not yet meet the standard of conceptual novelty and mechanistic study required for Nature Communications.
1. The manuscript repeatedly invokes packing parameter (p) to explain structural transitions (spherical-cylindrical-lamellar). However, no quantitative estimation or calculation of p is provided, no independent measurement of micelle geometry is shown. As such, p is used for qualitative explanatory, rather than prediction. This significantly weakens the claimed mechanistic depth.
2. A key claim is the transition from homogeneous self-assembly to interfacial anisotropic self-assembly. This transition underpins the entire mechanistic framework. However, there is no direct experimental validation of micelle localization (bulk vs interface), assembly dynamics, and interfacial adsorption processes.
3. The introduction of $\Delta\sigma$ as a governing parameter is conceptually reasonable, but currently unsupported, because no interfacial tension measurements are provided and the correlation between $\Delta\sigma$ and structural transitions is unclear.
4. The manuscript emphasizes “continuous structural evolution” within a single system. However, no continuous mapping or phase diagram is provided. Moreover, the transition boundaries are not defined.
5. The use of liquefied wood is presented as a key feature, yet its functional role is not clearly demonstrated. Does it influence self-assembly kinetics? Does it alter interfacial interactions? Is it essential for achieving the reported structures?

Reviewer #2

(Remarks to the Author)
In this manuscript, the authors developed a synthesis platform for preparing mesoporous carbon materials with versatile morphologies. The underlying mechanism involves surfactant self-assembly and surface tension at the oil/water interface. Admittedly, the microscopy images were high quality showing ordered structures at both nanoscale as well as particle/fiber scale. The concept/work itself might be interesting, but there are several concerns regarding this work:
1) Most characterization are only focused on the final carbon materials. The nanostructure evolution occurs in both solution assembly phase and polymerization process. However, there is no mesoscale characterization on these intermediate products, which limits the scope and impact of this study. Because of this, the discussions and mechanistic explanation are not well supported. For example, will polymerization induce further phase separation or phase change? At least SAXS and microscopy images should be provided at different process stages.
2) It was not clear that these self-assembled structures are at thermodynamic equilibrium state or kinetically arrested. This is important to understand especially towards more rational control in the phase morphology when scaling up.
3) What's the key benefits to attain these different morphologies? In many cases, the pore size are very comparable. Are the changes to their particle size/shape critical to influence their performance in intended applications?

4) In Figure 3, the corresponding pore size distribution seems to be different from what you would expect from the sorption isotherms. A sharp increase in p/p0=0.9 range should correspond to pore size at least larger than 10 nm, according to the BJH model as authors claimed to use. This part needs further examination.
5) Why the structure development is sensitive to the reaction temperature? The authors tried to explain this in the manuscript, but it was still not clear. This suggests there are more factors (either thermodynamic or kinetics) involved in controlling final morphologies than the content of TOP, surfactant ct, ethanol, etc. What's the polymerization kinetics at these temperatures?

Reviewer #3

(Remarks to the Author)
I co-reviewed this manuscript with one of the reviewers who provided the listed reports. This is part of the Nature Communications initiative to facilitate training in peer review and to provide appropriate recognition for Early Career Researchers who co-review manuscripts.

Reviewer #4

(Remarks to the Author)
This manuscript reports an interesting emulsion-mediated self-assembly approach for converting biomass into a diverse of mesoporous carbon nanoarchitectures. In particular, the tunable of nanofiber architecture from ordered multichannel structures to hollow nanofibers within a single synthetic platform is novel. Furthermore, the micelle structure and interfacial assembly behavior can be delicately adjusted by altering trioctyl phosphate and ethanol content. This is an original and well-designed study, supported by substantial experimental effort and extensive supplementary data. The results are convincing and should be of broad interest to the community working on biomass-derived carbon materials and porous nanoarchitectures. However, authors need to carefully consider the following comments before consideration for publication.

1. It would be great if the authors could provide more information of the LW carbon precursor (chemical constitution or polydispersity).
2. Some key structure labels are not fully aligned between the main text and the Supplementary Information. For example, the 0.3 mL TOP sample is described as “bowl-like nanospheres” in the main text, while MCNs-4 is listed as “vesicle-like” in Table S1; similarly, the copolymer-template section assigns hollow spheres to P108 in the text, whereas the figure caption and Table S1 assign them to P84. It should be carefully checked throughout for typographical, grammatical, and formatting errors.
3. How unambiguous is the separation between interfacial effects and kinetic effects in the proposed mechanism? The manuscript states that ethanol affects both interfacial tension and reaction kinetics, and HCl controls the kinetic matching window. Since TOP, ethanol, HCl, and surfactant composition all likely influence several coupled variables simultaneously, is the current mechanistic partitioning too clean relative to the complexity of the system?
4. Although differences in specific surface area, pore volume, and pore size distribution among nanofibers, multicavity spheres, and gear-like spheres are listed, there is no systematic correlation with ion transport pathways, electronic percolation thresholds, or thermoelectric performance. The structure-property relationship remains incomplete.
5. In the thermoelectric measurements, only a limited number of samples are tested, leading to the simplified conclusion that hollow carbon fibers exhibit the best performance. A comprehensive investigation of thermoelectric behavior across the continuously tunable morphology library is lacking.
6. The manuscript states that the interfacial self-assembly process is “consistent with the classical Frank–van der Merwe growth mode.” This is a rather specific growth concept that is usually used in the context of epitaxial thin-film growth. In the present case, the system involves soft composite micelles, an emulsion interface, and polymerization-coupled assembly. Is this analogy sufficiently supported, or does it risk overstating the mechanistic precision of the proposed growth pathway?
7. At several sections, external morphology, hollow internal architecture, and mesopore geometry are discussed together, which makes the interpretation less clear. Are these three structural levels being used independently, or are they intended to describe a single coupled evolution process?
8. The work emphasizes the thermopower advantage of the as-prepared materials, but does not provide a systematic comparison with similar ionic-electronic hybrid thermoelectric materials or other biomass-derived carbon-based thermoelectrics.
9. The sentence referring to “TEP with longer alkyl chains” is clearly inaccurate, since TEP has the shortest alkyl chain among the phosphate esters compared in this section. This should be corrected, and the surrounding discussion should be checked carefully for similar imprecision.
10. Reproducibility is a big issue in MCNs for its practical applications. How about the synthesis strategy’s versatility? It is better to add more comments.
11. XPS analysis indicates co-doping with N, P, and O; however, no control experiments (e.g., undoped or single-element-doped samples) are conducted to disentangle the individual contributions of heteroatoms to electronic conductivity, ionic thermal diffusion, and interfacial interactions.
12. The sample labeling is inconsistent. Some TEM/SEM images lack clear scale bars, and cross-sectional statistical analysis is insufficient. Key parameters such as fiber diameter, wall thickness, and particle size distribution are not quantitatively analyzed, weakening the claim of programmable morphology.

Version 1:

Reviewer comments:

Reviewer #1

(Remarks to the Author)
The authors have made substantial efforts to address the concerns raised in my previous review. I appreciate the extensive revisions to both the manuscript and the Supplementary Information, including the addition of systematic control experiments, coarse-grained simulations, mechanistic analyses, and a much more balanced discussion of the proposed assembly mechanism.
The authors have revised the presentation of the packing parameter by introducing a formulation-dependent packing tendency descriptor rather than claiming a direct determination of the classical packing parameter. The added simulations and analyses provide useful complementary support for the proposed structural evolution. Although direct in situ observation of the assembly process remains technically challenging, the combination of control experiments, precursor characterization, and interfacial simulations provides a stronger basis for the proposed transition from homogeneous assembly to interface-associated anisotropic organization. Importantly, the authors now present these mechanistic interpretations more cautiously and appropriately acknowledge their limitations.
The discussion of the effective interfacial-energy framework has also been significantly improved. The revised manuscript clearly distinguishes between experimentally observed phenomena and the proposed mechanistic interpretation, making the overall conclusions more convincing.
Overall, I believe the authors have satisfactorily addressed my major concerns. While some aspects of the proposed mechanism remain supported primarily by indirect evidence, this limitation is now appropriately acknowledged and does not diminish the significance of the work. The revised manuscript is substantially strengthened in both scientific rigor and clarity, and I have no further major concerns.

Reviewer #2

(Remarks to the Author)
The authors tried to improve the manuscript by including additional data and analysis.

Reviewer #3

(Remarks to the Author)
I co-reviewed this manuscript with one of the reviewers who provided the listed reports. This is part of the Nature Communications initiative to facilitate training in peer review and to provide appropriate recognition for Early Career Researchers who co-review manuscripts.

Reviewer #4

(Remarks to the Author)
After checking this revised version, the authors reasonably addressed my comments; therefore, I would like to recommend considering for publication.

Open Access This Peer Review File is licensed under a Creative Commons Attribution 4.0 International License, which permits use, sharing, adaptation, distribution and reproduction in any medium or format, as long as you give appropriate credit to the original author(s) and the source, provide a link to the Creative Commons license, and indicate if changes were made.
In cases where reviewers are anonymous, credit should be given to 'Anonymous Referee' and the source.
The images or other third party material in this Peer Review File are included in the article's Creative Commons license, unless indicated otherwise in a credit line to the material. If material is not included in the article's Creative Commons license and your intended use is not permitted by statutory regulation or exceeds the permitted use, you will need to obtain permission directly from the copyright holder.
To view a copy of this license, visit https://creativecommons.org/licenses/by/4.0/

REVIEWER COMMENTS

Response to Reviewer #1 Page 1 to 42
Response to Reviewer #2 Page 42 to 58
Response to Reviewer #3 Page 58
Response to Reviewer #4 Page 59 to 80

Reviewer #1:

General comments: ***The manuscript reports an emulsion-mediated self-assembly strategy to synthesize mesoporous carbon architectures with tunable morphologies and pore structures from liquefied wood. By varying the amount of trioctyl phosphate (TOP) and ethanol, the authors claim to achieve continuous structural evolution and propose a mechanism based on interfacial energy (Δσ) and micelle packing parameter (p). While the experimental scope is broad and the structural diversity is interesting, the conceptual advance is limited. The mechanistic framework largely relies on established concepts (e.g., packing parameter and interfacial self-assembly), which are used here primarily in a descriptive manner rather than enabling new understanding. In addition, key mechanistic claims are not supported by direct experimental evidence. Overall, the manuscript does not yet meet the standard of conceptual novelty and mechanistic study required for Nature Communications.***

General response: We sincerely thank you for the careful evaluation of our manuscript. We appreciate your recognition of the broad experimental scope and the interest in structural diversity achieved in our work. We agree that packing-parameter regulation and interfacial self-assembly are indeed well-established concepts in mesoporous materials during soft-template synthesis. These comments have been very helpful for us to substantially revise both the manuscript and the Supplementary Information.

Traditional soft templates or emulsion-mediated methods for preparing mesoporous carbon materials are usually well-defined precursors, such as dopamine or phenolic resin. Biomass-derived precursors are attractive for carbon-material synthesis because of their renewability, low cost, scalable availability and abundant oxygen-/phenolic-containing functional groups. However, their compositional complexity makes it difficult to coordinate precursor solvation, template interaction,

interfacial participation, and polymerization within a controllable soft-template assembly process. No research has been reported to apply biomass to the emulsion-mediated system to synthesize mesoporous carbon materials. In this work, **we first address this challenge by establishing a biomass-based emulsion-mediated self-assembly platform and proposing a liquefied-wood-enabled reactive-emulsion assembly chemistry mechanism framework,** in which liquefied wood is transformed from a passive carbon source into an active assembly precursor. **Importantly, the renewable, low-cost and scalable nature of biomass wood makes these programmable mesoporous carbon architectures more promising for practical production and industrial translation.**

Moreover, this mechanistic framework extends the established micelle-packing and interfacial self-assembly concepts and applies to a more complex biomass-enabled reactive emulsion system, in which micelle-curvature regulation, interfacial-affinity-biased localization and polymerization-induced structural fixation operate cooperatively, enabling control over both morphology and mesopore architecture in biomass-derived carbon materials. Among these, **we proposed a new regulatory approach and evolution rule for controlling micelle curvature and assembly modes.** Compared with previous homogeneous soft-template systems that mainly tune pore size or mesophase, and emulsion/droplet-directed systems based largely on model precursors such as dopamine or phenolic resins, **our system couples liquefied-wood precursor chemistry, TOP-regulated micelle swelling, ethanol-dependent solvation, oil/water interfacial localization and framework fixation within one platform.** This coupling enables programming of external morphology and internal mesopore architecture, giving access to ordered mesoporous carbon nanofibers, hollow carbon nanofibers, bowl-like structures and diverse mesoporous carbon nanospheres**, most of which are rarely reported.**

To strengthen the mechanistic basis, **we introduced a contact-derived packing-tendency index based on coarse-grained calculations, preserving conceptual continuity with the classical packing-parameter theory** while providing a formulation-specific descriptor for understanding micelle-curvature evolution and structural transition. We further added interfacial coarse-grained simulations to support TOP-induced micelle/precursor localization and anisotropic organization at the TOP/water interface, thereby strengthening the mechanistic basis for the interfacial assembly transition.

We reorganized control experiments to connect the proposed mechanism more directly with

experimental observations, including TOP content, ethanol content, F127 concentration, oil-chain structure, surfactant composition, HCl concentration. The TOP-dependent series supports the transition from homogeneous micellar assembly toward interface-associated anisotropic growth; the ethanol-dependent series demonstrates the regulation of solvation and micelle organization; the oil-structure-dependent controls clarify the importance of hydrophobic/oil-like interactions; and the surfactant-related controls further support the role of block-copolymer-directed soft templating. These results demonstrate that this emulsion-mediated system follows a cooperative thermodynamic-kinetic process, in which effective interfacial affinity and micelle-packing tendency guide the assembly pathway, while polymerization kinetics fixes or disrupts the evolving mesostructure depending on the kinetic matching condition.

In addition, we extended biomass-derived programmable mesoporous carbon architectures into the field of mixed ion–electron thermoelectrics. To the best of our knowledge, the direct use of biomass-derived carbon materials, particularly hollow carbon nanofibers with tunable pore architectures, for mixed ion–electron thermoelectric generators has not been reported. This application is not merely an additional performance demonstration, but represents an important conceptual extension of biomass-derived mesoporous carbons toward thermoelectric energy conversion and confirms the functional value of the programmable morphology/pore-architecture design.

Accordingly, we have substantially revised the manuscript and Supplementary Information to present the conceptual contribution more clearly and cautiously. The revised version refines the packing-parameter and interfacial-affinity discussions, incorporates coarse-grained simulation analyses, reorganizes the control experiments and clarifies the active role of liquefied wood. We hope that these revisions better demonstrate how this work extends established soft-template and emulsion-assembly principles into a biomass-derived reactive assembly system for programmable mesoporous carbon architectures. Considering the novelty and significance, this unique study has implications for a host of researchers in diverse fields related to material science, biomass-based materials, nanotechnology, electrochemistry, and self-assembly chemistry.

Comment 1: The manuscript repeatedly invokes packing parameter (p) to explain structural transitions (spherical-cylindrical-lamellar). However, no quantitative estimation or calculation of p is provided, no independent measurement of micelle geometry is shown. As such, p is used for

qualitative explanatory, rather than prediction. This significantly weakens the claimed mechanistic depth.

Response: We sincerely thank you for this important comment. We agree that the original wording may have overstated the predictive role of *p*. In the revised manuscript, we have avoided presenting *p* as an independently measured or quantitatively predictive parameter. Building on the physical logic of the classical packing-parameter framework, we introduced a contact-derived packing-tendency proxy, $p_{contact}$, and its normalized form p_{local}, to capture the same physical tendency in a formulation-dependent manner: the relative competition between hydrophobic core association and polar interfacial solvation. Thus, p_{local} retains conceptual continuity with the classical packing-parameter framework, but it is used only as a normalized comparative index of local packing tendency, not as an absolute classical packing parameter.

We appreciate your concern that direct measurement of micelle geometry would provide stronger mechanistic evidence. We fully agree that in situ characterization of the reaction process and its dynamic evolution would be highly valuable for gaining a deeper understanding of the structure-evolution mechanism and its relationship with material properties. This is also an important direction that we aim to pursue in our future studies. **However, in the present multicomponent emulsion system, direct in situ observation of composite micelles during the sealed high-temperature/high-pressure hydrothermal polymerization process is technically challenging. Such measurements may disturb the emulsion state, solvent composition, interfacial environment, and polymerization kinetics, thereby altering the assembly pathway that we intend to observe.** More importantly, the classical expression $p=V/a_0l$ was originally developed for relatively well-defined surfactant assemblies. Our system is not a single-component surfactant system, but a dynamically evolving multicomponent assembly composed of F127, TOP, ethanol, water, liquefied-wood-derived phenolic oligomers, melamine/formaldehyde-derived resin species, and hydrogen-bonding interactions. There is no unique molecular entity for which a single hydrophobic volume, headgroup area, and chain length can be unambiguously defined. As a result, assigning a single value of v, a_0, or l would be misleading, because these quantities are not fixed molecular constants but formulation-dependent effective parameters in the present reactive emulsion system. Nevertheless, the packing-parameter concept remains useful as a qualitative curvature framework to rationalize the experimentally observed structural evolution.

The final mesopore geometry is commonly related to the packing manner of the micelles, because the polymer framework polymerizes around the assembled template and the removal of the template leads to the formation of mesopores (from PPO segments) and micropores (from PEO segments). In recent soft-template and micelle-directed syntheses of mesoporous polymeric and carbonaceous materials, the geometry, curvature and interfacial organization of surfactant/block-copolymer micelles have been widely used to rationalize the resulting pore architectures(Chem. Soc. Rev. 2020, 4681; Chem. Mater. 2006, 4447; J. Am. Chem. Soc. 2005, 13508). **Our previous studies and Zhao's work** have demonstrated that spherical, cylindrical, lamellar or interface-confined micellar assemblies can be translated into distinct replicated mesopore geometries after precursor condensation and template removal (*Nat. Protoc.* 2025, 1310; *Sci. Adv.* 2021, eabi7403; *Sci. Adv.* 2022, eabo0283; *J. Am. Chem. Soc.* 2024, 17866). Cylindrical micelles usually lead to channel-like mesopores or ordered mesochannels, while spherical micelles tend to generate spherical or cage-like mesopores. Vesicular or lamellar assemblies can produce hollow, layered, or low-curvature pore architectures. Similarly, the incorporation of hydrophobic swelling agents into the hydrophobic domains of block-copolymer micelles is widely known to enlarge the hydrophobic core and alter the preferred micelle curvature, thereby affecting the resulting pore size and mesophase geometry. **Thus, in soft-template-derived mesoporous carbons, changes in the replicated pore structure can provide useful, although indirect, information about the evolution of micelle packing and curvature** (*J. Am. Chem. Soc*. 2024, 17866; *Chem. Rev.* 2021, 14349; *J. Am. Chem. Soc.* 2023, 27708). In our present system, Pluronic F127, TOP and LWMF form composite TOP/F127/LWMF micelles that co-assemble with the polymerizing LWMF precursor. The PPO-rich domains of F127 and the incorporated TOP molecules constitute the hydrophobic template domains, while the PEO segments interact with the LWMF precursor through hydrogen bonding and other interfacial interactions. After carbonization, removal of the F127/TOP domains leaves mesopores in the carbon framework. **Therefore, the observed evolution from ordered cylindrical mesochannels to hollow/lower-curvature structures and spherical mesopores provides reasonable evidence for changes in the packing and curvature of the composite micelles.**

To provide additional support while avoiding perturbation of the hydrothermal process, we performed TEM observations of the pre-assembled micelle/emulsion system under ambient conditions before hydrothermal treatment (Fig. R1). These ambient-condition TEM observations provide

supportive evidence for the formation and organization of composite micellar/aggregate species in the pre-assembly stage. We have clarified this limitation in the revised manuscript and now present these results as complementary evidence.

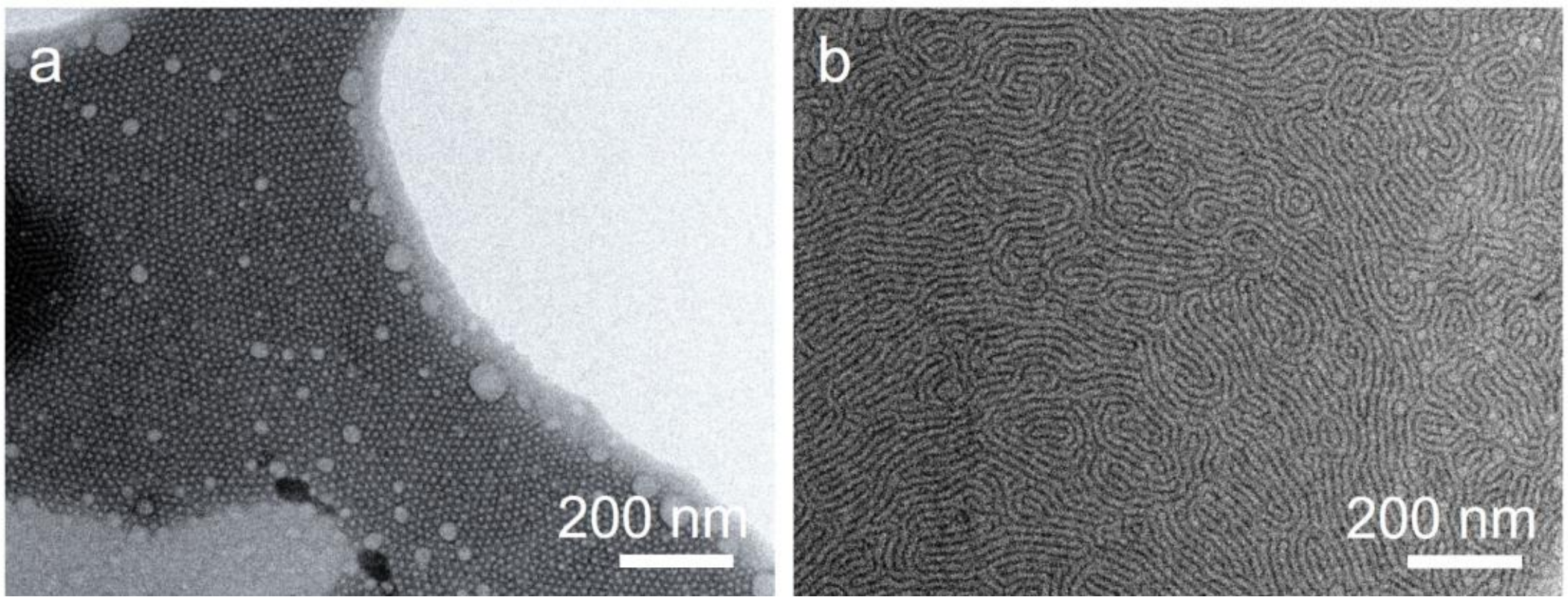

Fig. R1. TEM images of micelle/emulsion system under ambient conditions before hydrothermal treatment. (a) without TOP, (b) 0.2 mL TOP.

We have added formulation-guided coarse-grained molecular dynamics simulations in the revised Supplementary Information. Because the present precursor system is a multicomponent F127/TOP/EtOH/LW assembly rather than a single-component surfactant system, the classical expression $p = v/(a_0 l)$ cannot be unambiguously calculated from a single hydrophobic tail volume, headgroup area and chain length. Nevertheless, the physical concept underlying the classical packing parameter of the balance between hydrophobic core volume/association and polar interfacial area/solvation remains useful for rationalizing micelle-curvature evolution. We therefore defined a contact-derived packing-tendency proxy, $p_{contact}$, to compare the relative formulation-dependent packing tendency among different composition-variation models. Specifically, three coarse-grained models were constructed: P3-high EtOH, P1-base and P2-high TOP. P1-base was used as the reference model, P2-high TOP was constructed by increasing the TOP content, and P3-high EtOH was constructed by increasing the ethanol content while keeping the F127M, LW and water components constant. This design allowed us to compare how TOP and ethanol contents influence precursor-stage soft-template self-assembly. The contact-derived packing-tendency proxy was defined from the ratio between hydrophobic contact contributions and polar interfacial contact contributions. The hydrophobic contacts include LWOH-C–TOP, LWOH-C–PPO and TOP-PPO contacts, whereas the polar interfacial contacts include LWOH-OH–PEO, LWOH-OH–ETHX and TOP–PEO contacts. **To facilitate comparison with the classical micelle-curvature/packing**

framework, $p_{contact}$ was further normalized relative to the P1-base formulation to obtain a dimensionless packing-tendency index, p_{local}, which is used only as a comparative local packing-tendency index. The assignment of the P1-base value is therefore a normalization convention for displaying relative trends on a classical-packing-parameter-inspired scale, not an independent determination of the absolute classical *p*. The normalized contact-derived packing-tendency proxy increased from 0.295 ± 0.019 for P3-high EtOH to 0.400 ± 0.010 for P1-base and 0.538 ± 0.024 for P2-high TOP. This trend indicates that increasing TOP content enhances hydrophobic contact contributions and shifts the local organization toward a higher packing tendency, whereas higher ethanol content increases polar contact contributions and favors a lower local packing tendency. This trend is further supported by independent shape descriptors of the largest precursor core. The axis ratio axis1/axis3 increased from 1.217 ± 0.071 for P3-high EtOH to 1.352 ± 0.079 for P1-base and 1.400 ± 0.073 for P2-high TOP. Meanwhile, the relative shape anisotropy κ^2 increased from 0.0167 ± 0.0092 to 0.0284 ± 0.0098 and 0.0447 ± 0.0174, respectively. These results suggest a transition from more isotropic/high-curvature precursor aggregates under high-ethanol conditions toward more elongated and lower-curvature precursor organization under high-TOP conditions.

We have also added supplementary contact-proxy decomposition, convex-hull-based aggregate-scale compactness descriptors and additional shape descriptors, including axis2/axis3, asphericity and acylindricity. These analyses further support the formulation-dependent packing interpretation and help ensure that the conclusion is not based on a single descriptor. The corresponding model construction, simulation conditions, local packing proxy definition, representative structures and quantitative analyses are now provided in Supplementary Note 1.5, Supplementary Table 4 and Figure 51,52. We would like to emphasize that these CG simulations are not intended to reproduce the full hydrothermal curing, carbonization, template removal or framework shrinkage processes, nor are they used to claim absolute numerical values of the classical packing parameter. Instead, they provide complementary comparative evidence for precursor-stage soft-template organization under controlled TOP and ethanol variations. Together with the replicated mesopore structures and systematic control experiments, the simulations support the revised interpretation that micelle packing/curvature plays an important role in regulating the observed mesostructural evolution.

Figure-to-conclusion correspondence for Reviewer Comment 1:

Conclusion or mechanistic point	Supporting	How the figure supports the conclusion

	figure/table	
Controlled formulation variation was constructed to isolate the effects of TOP and EtOH contents.	Table S4; Fig. S51a-c	P3-highEtOH, P1-base and P2-highTOP use controlled changes in ETHX or TOP while keeping F127M, LWOH and water constant, allowing composition-dependent comparison.
A relative contact-derived proxy was introduced to compare formulation-dependent local packing tendency.	Eqs. S1–S2; Supplementary Table S4; Fig. S51g	The contact-derived packing-tendency proxy is derived from hydrophobic and polar contact contributions and normalized to the base formulation for relative comparison..
Increasing TOP content shifts the local packing tendency toward a larger p_{local} value.	Fig. S51g	p_{local} increases from P3-highEtOH to P1-base and P2-highTOP, showing a composition-dependent trend.
The morphology evolves from more isotropic/high-curvature aggregates toward more elongated aggregates.	Fig. S51d–f; Fig. S51h–i	Representative local aggregates and quantitative axis1/axis3 and κ^2 values show increasing anisotropy from P3-highEtOH to P2-highTOP.
The hydrophobic and polar contributions underlying p_{local} are explicitly decomposed.	Fig. S52a	Hydrophobic and polar contact proxies show how TOP/PPO/LWOH-C and PEO/ETHX/LWOH-OH contacts contribute to the packing-tendency proxy.
Additional geometric descriptors verify that the conclusion is not based on one parameter only.	Fig. S52b–e	Aggregate-scale p_{eff} and supplementary shape descriptors, including axis2/axis3, asphericity and acylindricity, provide independent support for morphology changes.

Added text in the revised supplementary information:

1.5 Coarse-grained simulation of formulation-dependent contact-derived packing tendency and precursor morphology evolution

1.5.1 Model construction and composition-variation design

To support the analysis of precursor-stage organization under different formulation conditions, three formulation-guided coarse-grained (CG) models were constructed, namely P3-high EtOH, P1-base and P2-high TOP. These models were designed to compare the influence of two compositional variables, TOP content and EtOH content, on the local organization of the F127/TOP/EtOH/LWOH precursor system. The key model names, bead labels and quantitative descriptors used in the CG simulations are defined in Supplementary Table 3 to avoid ambiguity between experimental components, CG residue names and analysis descriptors.

P1-base was used as the reference model, corresponding to the baseline condition with TOP=0.1 mL and EtOH=10 mL. P2-high TOP was constructed by increasing the number of TOP molecules from 40 to 120 while keeping the number of ETHX beads unchanged relative to P1-base. P3-high EtOH was constructed by increasing the number of ETHX beads from 9000 to 22500 while keeping the number of TOP molecules unchanged relative to P1-base. The numbers of F127M, LWOH and water beads were kept constant in the three models to allow a controlled comparison of the effects of TOP and

EtOH contents.

The EtOH/H_2O ratios in the CG models were mapped according to the corresponding experimental EtOH/H_2O molar ratios, considering that one Martini water bead represents approximately four water molecules. Thus, the ETHX/W ratios in P1-base and P3-high EtOH correspond to the experimental EtOH/H_2O ratios for the 10 mL and 25 mL EtOH formulations, respectively. The TOP variation was represented by a threefold increase in the number of TOP molecules, consistent with the experimental change from 0.1 mL to 0.3 mL TOP.

LWOH was used as a phenolic-rich precursor surrogate to represent hydroxyl-containing aromatic units in the precursor system. The CG models were not intended to reproduce the full experimental composition on an absolute mass basis or to explicitly include all chemical reactions occurring during curing and carbonization. Instead, they were used as comparative models to examine how controlled variations in TOP and EtOH contents affect precursor-stage soft-template organization. The corresponding CG compositions and key morphology descriptors are summarized in Supplementary Table 4.

1.5.2 Simulation conditions and analysis protocol

All coarse-grained (CG) molecular dynamics simulations were performed using GROMACS with Martini-type CG interaction parameters. The topology files used in the simulations included martini_v3.0.0.itp, martini_v3.0.0_solvents_v1.itp, martini_v3.0.0_ions_v1.itp, F127M_cg.itp, TOP_cg.itp, ETHX_cg.itp and LWOH.itp. F127M was represented by PEO-type polar beads and PPO-type hydrophobic beads. LWOH was used as a phenolic-rich precursor surrogate and was represented by hydrophobic/aromatic carbon beads (LWOH-C) and polar hydroxyl-rich beads (LWOH-OH). ETHX was used as the CG representation of ethanol, and Martini water beads were used as the solvent.

For each CG model, the initial structure was first subjected to energy minimization, followed by stepwise equilibration. A short NVT initialization step was performed with newly generated velocities to stabilize the minimized structure. Subsequent NVT soft-equilibration, gradual relaxation and NPT soft-equilibration steps were then performed before the production simulation. Periodic boundary conditions were applied in all three directions. The production simulation was performed for 15 ns in total, including an initial 2.5 ns run followed by a 12.5 ns continuation run.

For non-bonded interactions, the Verlet cutoff scheme was used. Electrostatic interactions were treated using the reaction-field method with a cutoff of 1.1 nm and a relative dielectric constant of 15. The van der Waals cutoff was set to 1.1 nm. Temperature coupling was performed using the velocity-rescaling thermostat. The main equilibration and production simulations used a time step of 0.01 ps, while the short NVT initialization step used a smaller time step of 0.002 ps to improve stability during the early relaxation stage. The simulations were used to compare the relative precursor-stage organization under different composition-variation conditions, rather than to explicitly reproduce the polymerization process.

Quantitative analyses were performed using the last three representative frames. Specifically, the frames extracted at 7500, 10000 and 12500 ps from the 12.5 ns continuation trajectory correspond to cumulative simulation times of approximately 10.0, 12.5 and 15.0 ns, respectively. Contact analyses

were performed using a 1.0 nm cutoff. The template core was defined using PPO and TOP beads, while the precursor core was defined using PPO, TOP and LWOH-C beads. The largest precursor core was used for shape-descriptor analysis.

1.5.3 Definition of the contact-derived packing-tendency proxy

Because the present system is a multicomponent reactive system rather than a single-component surfactant assembly, the classical molecular packing parameter cannot be directly calculated from uniquely defined hydrophobic volume, headgroup area and chain length. Nevertheless, the physical concept underlying the classical packing parameter of the balance between hydrophobic core volume/association and polar interfacial area/solvation remains useful for rationalizing micelle-curvature evolution. To extend this concept to the present multicomponent system, we defined a contact-derived packing-tendency proxy, $p_{contact}$, to compare the relative balance between hydrophobic and polar interfacial contributions among different formulations.

$$p_{contact} = (\mathrm{CLWOH\text{-}C–TOP+CLWOH\text{-}C–PPO+CTOP–PPO})/(\mathrm{CLWOH\text{-}OH–PEO+CLWOH\text{-}OH–ETHX +CTOP–PEO}) \quad (S1)$$

where CLWOH-C–TOP, CLWOH-C–PPO and CTOP–PPO represent the hydrophobic contact contributions from the phenolic/aromatic carbon beads of LWOH, TOP and the PPO-rich hydrophobic domains of F127. CLWOH-OH–PEO, CLWOH-OH–ETHX and CTOP–PEO represent the polar interfacial contact contributions from hydroxyl-rich LWOH beads, PEO segments and ETHX beads. This contact-based definition was used to compare the relative local packing tendency among different composition-variation models. $p_{contact}$ is not equivalent to the classical molecular packing parameter. Instead, it is a contact-derived descriptor that translates the physical balance considered in the classical packing-parameter framework into a form applicable to the present multicomponent reactive emulsion. A larger $p_{contact}$ indicates a stronger contribution from hydrophobic association relative to polar interfacial solvation, which is expected to favor a higher local packing tendency and lower preferred curvature. Conversely, a smaller $p_{contact}$ indicates stronger polar interfacial solvation relative to hydrophobic association, which is expected to favor a lower local packing tendency and higher preferred curvature.

To facilitate comparison with the classical micelle-curvature/packing framework, $p_{contact}$ was further normalized relative to the P1-base formulation to obtain a dimensionless packing-tendency index, p_{local}. The P1-base formulation was used only as an internal reference for normalization. Therefore, the resulting p_{local} values should be interpreted as relative, formulation-dependent indices rather than absolute classical packing parameters. They are used to compare whether TOP or ethanol shifts the precursor aggregates toward relatively higher or lower packing tendencies.

For presentation on a classical-packing-parameter-inspired scale, P1-base was assigned a reference value of p_{local}=0.40, a value chosen only as an internal normalization convention to represent an intermediate packing-tendency state, which is placed near the commonly discussed $1/3<p<1/2$ region for ease of qualitative comparison. The normalized local packing proxy was calculated as:

$$p_{local} = 0.4 \times [p_{contact} / p_{contact(P1\text{-}base)}] \quad (S2)$$

1.5.4 Shape descriptors and aggregate-scale compactness descriptor

To evaluate the morphology of precursor aggregates, the largest precursor core was identified from PPO, TOP and LWOH-C beads using a 1.0 nm cutoff. The shape of the largest precursor core was quantified using the eigenvalues of the gyration tensor. The three principal eigenvalues were denoted as $\lambda1$, $\lambda2$ and $\lambda3$, with $\lambda1 \geq \lambda2 \geq \lambda3$.

The axis ratio axis1/axis3 was calculated from the square roots of the largest and smallest eigenvalues and was used to describe the elongation degree of the aggregate:

$$\text{axis1/axis3} = \sqrt{\lambda1}/\sqrt{\lambda3} \quad \text{(S3)}$$

The relative shape anisotropy κ^2 was used to quantify the deviation from an isotropic spherical shape. A lower axis1/axis3 and κ^2 indicate a more isotropic or high-curvature aggregate, whereas higher values indicate a more elongated and anisotropic precursor assembly. Additional shape descriptors, including axis2/axis3, asphericity and acylindricity, were also calculated and are shown in Supplementary Fig. 52. In addition, a convex-hull-based aggregate-scale p_{eff} was calculated as an auxiliary compactness descriptor. The template core was defined as PPO+TOP, while the precursor core was defined as PPO+TOP+LWOH-C. The aggregate-scale p_{eff} was calculated as:

$$p_{eff} = V_{core}/(A_{hull} R_{\_core,90}) \quad \text{(S4)}$$

where V_{core} is the effective core volume, A_{hull} is the convex-hull surface area and $R_{core,90}$ is the 90th percentile distance of core beads from the core center. This descriptor reflects the geometric compactness of the aggregate and is simultaneously affected by core volume, surface area and aggregate dimension. Therefore, p_{eff} was used only as a supplementary aggregate-scale compactness descriptor rather than as a direct substitute for the classical molecular packing parameter.

1.5.5 Results and interpretation

The normalized local packing proxy showed a clear composition-dependent trend among the three CG models. As summarized in Supplementary Table 4, p_{local} increased from 0.295±0.019 for P3-highEtOH to 0.400±0.010 for P1-base and 0.538±0.024 for P2-highTOP. The lower p_{local} value in P3-highEtOH was associated with a higher polar contact contribution, whereas the higher p_{local} value in P2-highTOP was associated with an increased hydrophobic contact contribution. These results indicate that the contact-defined packing-tendency proxy was sensitive to the changes in EtOH and TOP contents.

The morphology descriptors further supported the variation in precursor aggregate organization. P3-highEtOH showed the lowest axis1/axis3 value of 1.217±0.071 and the lowest κ^2 value of 0.0167±0.0092, indicating a more isotropic and high-curvature precursor aggregate. P1-base exhibited intermediate values, with axis1/axis3=1.352±0.079 and κ^2=0.0284±0.0098. P2-highTOP showed the highest anisotropy, with axis1/axis3=1.400±0.073 and κ^2=0.0447±0.0174, suggesting a tendency toward more elongated and lower-curvature precursor assemblies.

The representative CG snapshots shown in Supplementary Fig. 51 are consistent with these quantitative descriptors. P3-highEtOH exhibited a more compact and high-curvature local aggregate morphology, P1-base showed an intermediate organization, and P2-highTOP displayed a more elongated precursor aggregate. These results support the use of the contact-derived p_{local} and shape descriptors to compare precursor-stage organization under different composition-variation conditions.

The supplementary quantitative analyses shown in Supplementary Fig. 52 further substantiate the contact-derived packing interpretation and the aggregate morphology descriptors. The hydrophobic and polar contact proxies shown in Supplementary Fig. 52a decompose the numerator and denominator of p_{local}, confirming that the increase in p_{local} for P2-highTOP originates from enhanced hydrophobic contacts, whereas P3-highEtOH is associated with stronger polar contact contributions. The convex-hull-based compactness descriptors shown in Supplementary Fig. 52b provide an aggregate-scale geometric cross-check of the local packing tendency for both template and precursor cores. The additional shape descriptors shown in Supplementary Fig. 52c-e, including axis2/axis3, asphericity and acylindricity, further support the morphology evolution inferred from axis1/axis3 and κ^2.

It should be noted that the CG simulations describe precursor-stage soft-template organization rather than the final carbonized mesoporous structure. Processes such as M/F condensation, curing, carbonization, template removal and framework shrinkage were not explicitly simulated. Therefore, the CG results are interpreted as comparative mechanistic evidence for precursor-stage organization, rather than as a direct reproduction of the final mesoporous carbon morphology.

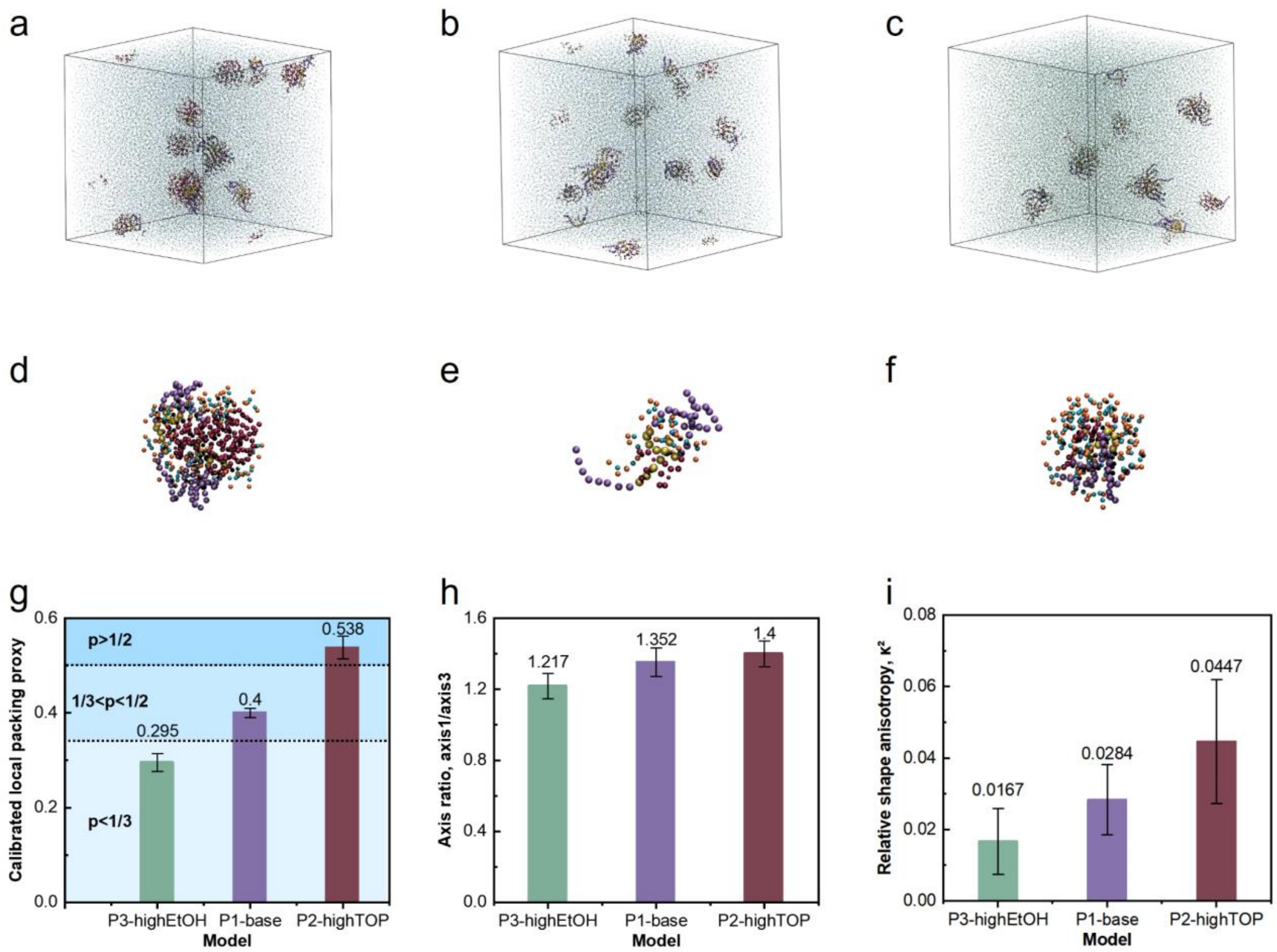

Supplementary Fig. 51. CG simulation of composition-dependent local packing tendency and precursor morphology evolution. (a–c) Representative full-system snapshots of P3-highEtOH, P1-base and P2-highTOP, respectively. Water molecules were omitted for clarity. (d–f) Representative

local precursor aggregates extracted from the corresponding systems. (g) Normalized contact-derived packing-tendency proxy, p_{local}. The shaded background regions schematically indicate the commonly discussed classical packing-parameter ranges only for qualitative reference; the plotted p_{local} values should not be interpreted as absolute classical p values or strict regime assignments. (h) Axis ratio axis1/axis3 of the largest precursor core. (i) Relative shape anisotropy κ^2 of the largest precursor core. Error bars represent the standard deviation over the last three representative frames.

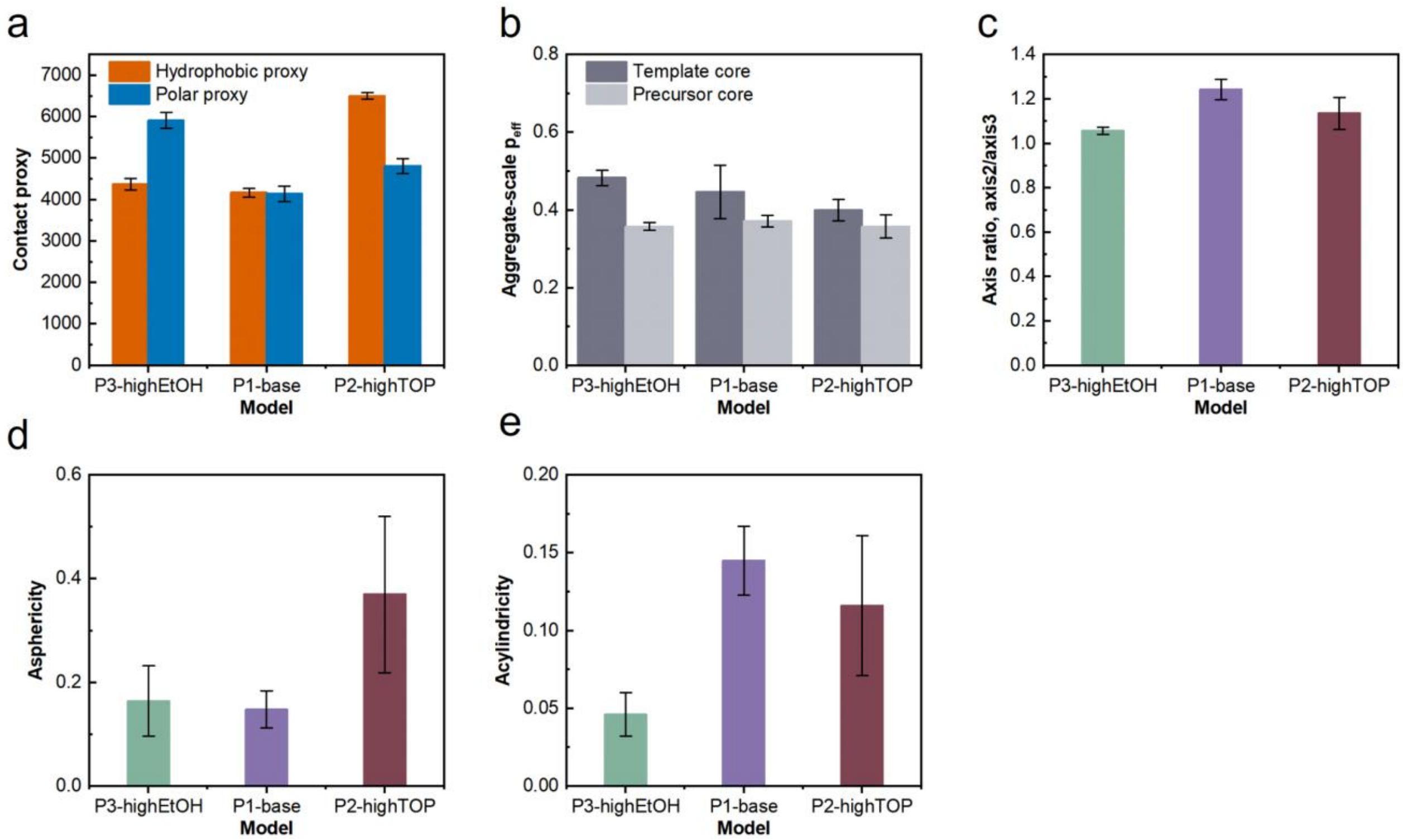

Supplementary Fig. 52. Supplementary quantitative analysis of contact-derived packing contributions, aggregate-scale compactness descriptors and additional shape parameters in P3-highEtOH, P1-base and P2-highTOP systems. (a) Hydrophobic and polar contact proxies are used to define the local packing tendency. The hydrophobic proxy was calculated from LWOH-C–TOP, LWOH-C–PPO and TOP–PPO contacts, whereas the polar proxy was calculated from LWOH-OH–PEO, LWOH-OH–ETHX and TOP–PEO contacts. (b) Convex-hull-based aggregate-scale compactness descriptors, p_{eff}, were calculated for template and precursor cores. The template core was defined as PPO+TOP, while the precursor core was defined as PPO+TOP+LWOH-C. (c–e) Supplementary shape descriptors of the largest precursor core, including axis2/axis3, asphericity and acylindricity. Error bars represent the standard deviation over the last three representative frames.

Supplementary Table 3. Explanation of key terms and abbreviations used in the CG simulations.

Term/abbreviation	Meaning in this work	Use and interpretation
F127	Pluronic F127, a PEO-PPO-PEO triblock copolymer.	Soft-template polymer in precursor self-assembly.
F127M	Residue/model name of the Martini CG representation of F127 in topology and coordinate files.	Not a new chemical component; discussion can refer to F127.
PEO	Hydrophilic poly(ethylene oxide) blocks of F127, represented by E-type beads.	Forms the polar corona and interacts with water, ETHX and hydroxyl-rich

		LW sites.
PPO	Hydrophobic poly(propylene oxide) block of F127, represented by P-type beads.	Forms the hydrophobic domain and is preferentially anchored by TOP-rich regions.
TOP	Hydrophobic TOP component in the experimental formulation and CG model.	Forms TOP-rich domains/slabs and tunes the hydrophobic interfacial environment.
EtOH/ETHX	EtOH denotes ethanol in the experiment; ETHX denotes the Martini CG ethanol bead.	Represents ethanol-related polar solvent effects in the CG models.
W	Martini water bead; one W bead represents approximately four water molecules.	Aqueous solvent in all CG models.
LW	Liquefied wood, treated as a polyhydroxyl-containing phenolic precursor/carbon source.	General term for precursor chemistry and interfacial role.
LWOH	Residue/model name of the CG representation of LW containing aromatic carbon-rich beads and hydroxyl-rich polar beads.	Used in topology/coordinate files and bead-level contact analysis; equivalent to the modeled LW component.
LW-C	Aromatic/carbon-rich beads of the CG LW model.	Hydrophobic/aromatic contact contributions, e.g., LW-C-TOP and LW-C-PPO.
LW-OH	Hydroxyl-rich polar beads of the CG LW model.	Polar contact contributions, e.g., LW-OH-PEO and LW-OH-ETHX.
P-series models	Formulation-guided CG models including P3-highEtOH, P1-base and P2-highTOP.	Compare local packing tendency and precursor morphology under different EtOH/TOP contents.
Ea-series models	Actual-ratio long-box TOP-rich/water interfacial models.	Validate interfacial localization, PPO anchoring and anisotropic organization.
TOP-rich slab	Continuous TOP-rich interfacial domain identified in the long-box models.	Stability is evaluated by TOP cluster number and largest TOP-cluster fraction.
Aggregate	F127/LW-containing precursor aggregate or selected bead group used for shape/orientation analysis.	Evaluates elongation, anisotropy and interfacial orientation.
C_A-B or N(A-B)	Number of bead-bead contacts between groups A and B within the selected cutoff.	C or N denotes contact count, not carbon; contacts/bead is normalized by bead number.
p_{local}	Contact-derived local packing proxy normalized relative to P1-base.	Relative descriptor of local packing tendency, not an absolute classical packing parameter.
p_{eff}	Convex-hull-based aggregate-scale compactness descriptor.	Supplementary geometric support for aggregate compactness.
axis1/axis3	Axis ratio calculated from eigenvalues of the	Higher values indicate a more

	gyration tensor.	elongated aggregate or chain configuration.
kappa2 (κ^2)	Relative shape anisotropy calculated from the gyration tensor.	Higher values indicate stronger deviation from an isotropic spherical shape.
S	Orientation parameter calculated relative to the interface normal.	S=-0.5 ideal in-plane alignment; S=0 random; S=1 perpendicular alignment.

Supplementary Table 4. Formulation mapping, coarse-grained composition and key morphology descriptors of P3-highEtOH, P1-base and P2-highTOP systems.

Model	P3-highEtOH	P1-base	P2-highTOP
Experimental condition	TOP=0.1 mL, EtOH=25 mL	TOP=0.1 mL, EtOH=10 mL	TOP=0.3 mL, EtOH=10 mL
F127M	16	16	16
TOP	40	40	120
ETHX	22500	9000	9000
LWOH	120	120	120
W	36538	36538	36538
Calibrated p_{local}	0.295 ± 0.019	0.400 ± 0.010	0.538 ± 0.024
axis1/axis3	1.217 ± 0.071	1.352 ± 0.079	1.400 ± 0.073
κ^2	0.0167 ± 0.0092	0.0284 ± 0.0098	0.0447 ± 0.0174

Normalized p_{local} was obtained by normalizing the contact-derived local packing proxy to P1-base and assigning P1-base a reference value of 0.40. The axis1/axis3 and κ^2 values describe the shape anisotropy of the largest precursor core. All values are reported as mean ± standard deviation over the last three representative frames.

Revised text in manuscript:

Revised text ① (Page 3, line 49): The structural modulation is predominantly restricted to adjustments of the micelle curvature, swelling behavior or nucleation kinetics[19–24]. These factors are often optimized separately, making it difficult to achieve coupled control over both morphology and mesostructure within a single synthetic system to fabricate multi-mode mesoporous carbon materials.

Revised text ② (Page 14, line 251: Under a TOP volume of 0.2 mL, increasing ethanol content drove a transition from one-dimensional fibers to nanospheres, accompanied by a change in mesopore geometry from cylindrical to spherical pores.

Revised text ③ (Page 14, line 260: The evolution of mesostructures reveals that increasing ethanol content improves the solvation of PEO segments and hydroxyl-rich LWMF fragments, thereby favoring more dispersed or isotropic precursor organization. At the same time, ethanol increases the effective polar/interfacial contribution of the composite micelles and shifts the preferred micelle curvature toward higher-curvature organizations.

Revised text ④ (Page 19, line 349 a higher F127 concentration increases the micelle population density and enhances steric repulsion among micelles, which increases the effective polar/interfacial contribution and favors higher-curvature assemblies. In contrast, increasing TOP content swells the PPO-rich hydrophobic domains and increases the effective hydrophobic contribution, driving the mesostructure toward lower-curvature cylindrical, hollow or lamellar-like configurations. (Supplementary Fig. 36). These results confirm that F127 defines the soft-template assembly window. A sufficient amount of F127 is required to generate composite micelles and preserve ordered mesopores, while the final morphology is further selected by TOP-mediated interfacial affinity and ethanol-regulated solvation/curvature effects.

Revised text ⑤ (Page 20, line 368): Such regulation changes the preferred micelle curvature and induces distinct self-assembly pathways, including homogeneous assembly, vesicle formation, and interfacial overgrowth.

Revised text ⑥ (Page 31, line 600): Qualitative micelle-curvature/packing framework used to rationalize the observed pore-architecture evolution.

Revised text ⑦ (Page 31, line 603: Coarse-grained simulations provide additional support for this coupled mechanism (Supplementary Table 3). For formulation-dependent micelle packing, the classical packing parameter cannot be uniquely calculated in this reactive multicomponent system. A contact-derived packing-tendency proxy and aggregate-shape descriptors were used to compare relative packing tendencies (Supplementary Table 4). The increase in the normalized local packing proxy and aggregate anisotropy under high-TOP conditions supports a shift from more isotropic/high-curvature aggregates toward elongated or lower-curvature organizations (Supplementary Fig. 51,52).

Comment 2: A key claim is the transition from homogeneous self-assembly to interfacial anisotropic self-assembly. This transition underpins the entire mechanistic framework. However, there is no direct experimental validation of micelle localization (bulk vs interface), assembly dynamics, and interfacial adsorption processes.

Response: We sincerely thank you for your insightful and constructive comments. In the revised manuscript, we have substantially revised this section and now present the homogeneous-to-interfacial transition as a mechanistic interpretation supported by complementary experimental observations, systematic control experiments and interfacial coarse-grained simulations.

We agree with you that the proposed mechanism must be supported by evidence specific to our own system. However, in the present multicomponent emulsion-polymerization system, direct in situ visualization of transient TOP/F127/LWMF composite micelles during hydrothermal treatment is technically challenging. The assembly process occurs in a sealed high-temperature/high-pressure autoclave and is simultaneously coupled with LWMF polymerization. **Opening the system, introducing optical or electron-beam probes, diluting the emulsion, or transferring the system for cryogenic observation would inevitably disturb the emulsion state, TOP/water interface, solvent composition, pressure, micelle self-assembly and polymerization kinetics**. Therefore, direct real-time tracking of micelle migration from the bulk aqueous phase to the oil/water interface under the actual hydrothermal reaction conditions is not experimentally accessible without substantially perturbing the pathway that we aim to examine. This is an important challenge that we are actively considering, and it will be a major focus of our future work, for example through the development of less perturbative in situ or quasi-in situ characterization approaches capable of capturing micelle/interfacial evolution under conditions closer to the actual emulsion-polymerization process.

To further address the specific concern regarding micelle localization and interfacial adsorption, we added actual-ratio long-box TOP-rich/water interfacial CG simulations in the revised Supplementary Information. The low-TOP and high-TOP interfacial models used the same molecular compositions as the corresponding P-series models, except for the TOP content. Both models maintained a single TOP-rich slab, with the largest TOP-cluster fraction of 1.0 over the final 2 ns. Increasing TOP content from 40 to 120 molecules substantially enhanced the interfacial coupling between TOP and the F127/LWOH aggregate. The normalized PPO-TOP contact density increased from 2.27 to 8.48 contacts per bead, while the PPO-water contact density decreased from 1.33 to 0.081 contacts per bead. The normalized LWOH-TOP contact density also increased from 1.22 to 3.48 contacts per bead, while LWOH retained substantial water contact, supporting its location in the TOP-rich/water interfacial transition region. Furthermore, TOP enrichment induced stronger in-plane anisotropic organization. The aggregate orientation parameter shifted from -0.275 to -0.476, and the F127 orientation parameter shifted from -0.071 to -0.441, approaching the theoretical value of -0.5 for ideal in-plane alignment. These results provide molecular-level computational evidence that TOP enrichment promotes PPO anchoring and anisotropic organization at the TOP-rich/water interface. The

corresponding analysis has been added as Supplementary Note 1.6, Supplementary Table 5 and Supplementary Fig. 53. We note that these interfacial simulations are not intended to reproduce the complete hydrothermal curing, carbonization or template-removal process. They are also not used to claim direct real-time observation of long-range micelle migration from a far-away bulk aqueous phase. Instead, they were designed to test whether the actual formulation components can form stable TOP-rich/water interfaces, whether F127 and LWOH can localize at such interfaces, and whether increasing TOP content enhances interfacial anchoring and anisotropic organization.

To further address this concern, **we have strengthened the mechanistic support using multiple complementary experiments of evidence,** as detailed below. First, the initial emulsion observation confirms the presence of TOP/water interfaces before hydrothermal polymerization. After stirring, the reaction mixture turns into a milk-like emulsion, and optical microscopy reveals uniform TOP droplets in the system. This observation confirms that the introduction of TOP creates well-defined oil/water interfaces, which are absent in the TOP-free system. These interfaces provide a physically plausible location for interfacial micelle/precursor co-assembly. The TOP-free control experiment provides a direct comparison between homogeneous aqueous self-assembly and interface-mediated assembly. In the absence of TOP, no oil/water interface is introduced, and the product forms conventional dodecahedral carbon nanoparticles with ordered mesopores, which is consistent with homogeneous micelle self-assembly in a single aqueous phase. By contrast, after TOP is introduced, fibrous and hollow architectures appear. This comparison indicates that TOP does not merely act as a passive swelling agent, but changes the assembly environment by introducing oil/water interfaces. The TOP-dependent structural evolution supports the redirection of the assembly pathway. As the TOP amount increases, the products evolve from ordered mesoporous nanofibers to hollow nanofibers and bowl-like nanostructures. Such a systematic transition cannot be fully explained by conventional homogeneous aqueous micelle packing alone. Instead, it is consistent with the increasing contribution of TOP-rich interfaces, which promote interfacial adsorption, directional organization and anisotropic overgrowth of TOP/F127/LWMF composite micelles. Time-dependent intermediate observations provide process-level evidence for polymerization-coupled assembly. The polymeric precursor collected at early hydrothermal stages shows aggregated or partially elongated intermediates, whereas fibrous frameworks with hollow/mesoporous features become evident at later stages. Prolonging the reaction does not lead to obvious further morphological transformation or coarsening. These

observations suggest that the hollow fibrous architecture is generated during the hydrothermal assembly/polymerization process and then kinetically fixed by precursor condensation, rather than being produced only during post-treatment or carbonization. Fifth, the as-made polymer precursors already exhibit the key anisotropic and hollow/mesoporous features before carbonization. SEM images show fibrous polymeric precursors with pronounced surface striations, while TEM images reveal hollow mesochannels in the polymeric nanofibers. These results demonstrate that the anisotropic fibrous framework and hollow mesostructure are established at the precursor stage. Therefore, the observed architecture is not a secondary artifact caused by carbonization shrinkage or template removal, but is associated with the emulsion-mediated assembly/polymerization process. Sixth, oil-phase control experiments further support the role of interfacial interactions. When TOP was replaced by trialkyl phosphate oils with different alkyl-chain lengths and hydrophobicities, the products showed chain-length-dependent structural evolution. Shorter-chain and less hydrophobic phosphate oils favored dodecahedral particles with ordered mesopores, indicating a dominant homogeneous assembly pathway. In contrast, longer-chain and more hydrophobic phosphate oils promoted more anisotropic and hollowing structures. These results indicate that oil-phase hydrophobicity and interfacial coupling are important factors in directing the transition from homogeneous assembly to interface-mediated anisotropic growth.

Zhao's work on asymmetric mesoporous hemispheres at liquid-liquid interfaces has directly shown that droplet surfaces can serve as preferential nucleation and growth sites for micelle anisotropic self-assembly; the self-assembly behavior could be switched from isotropic to anisotropic modes through an interface-energy-mediated nucleation and growth process (*J. Am. Chem. Soc.* 2022, 15754). This provides a direct precedent for our proposal that the TOP-rich/water interface can change the assembly locus of F127/TOP/LWMF composite micelles from the bulk aqueous phase to an interfacial region. This interfacial-assembly rationale is further supported by droplet-directed carbon systems. In the synthesis of semifootball-like carbon nanoparticles, emulsion droplets were shown to direct anisotropic growth of carbonaceous precursors at the droplet interface, and the particle morphology could be changed by adjusting the oil-phase polarity (*Adv. Funct. Mater.* 2024, 2400503). This is mechanistically relevant to our work because TOP is not treated only as a pore-swelling additive; it also creates a hydrophobic liquid domain and an oil/water interface that can alter precursor/micelle adsorption and directional growth. Similarly, emulsion-confined environments can tailor hollow

carbon architectures, supporting the idea that carbonaceous mesostructures can be generated through emulsion-mediated precursor organization rather than only through homogeneous solution assembly (*JACS Au* 2024, 2666). **Our previous dual-emulsion-directed micelle assembly work provides another important precedent.** Emulsion interfaces guided micelle organization into asymmetric monolayer mesoporous nanosheets with semi-opened pores and two-sided surface anisotropy (*J. Am. Chem. Soc.* 2023, 27708). This demonstrates that an emulsion interface can impose directional constraints on micelle organization and pore opening. In addition, Zhao' emulsion-oriented assembly of Janus double-spherical mesoporous nanoparticles showed that emulsion interfaces can spatially separate and guide different mesoporous domains within one particle (*Nat. Chem.* 2023, 832). These previous studies establish three points that are relevant to our mechanism: first, liquid-liquid interfaces can become the sites for micelle/precursor nucleation and growth; second, interfacial energy and droplet chemistry can switch assembly from isotropic or homogeneous growth to anisotropic interfacial organization; and third, emulsion-confined assembly can generate hollow, asymmetric and hierarchical carbonaceous mesostructures. Therefore, in the present TOP/F127/LWMF system, it is reasonable to propose that increasing TOP content not only changes micelle packing, but also creates stronger TOP-rich/water interfacial coupling, thereby redirecting F127/TOP/LWMF composite micelles or micellar aggregates from homogeneous aqueous self-assembly toward interface-mediated anisotropic organization. In the present TOP/F127/LWMF system, we do not simply transfer these mechanisms by analogy. Instead, **we use these previous studies as conceptual precedents and combine them with system-specific evidence, including TOP-free controls, TOP-dependent morphology evolution, time-dependent intermediate observations, polymer precursor characterization, oil-phase control experiments and actual-ratio TOP-rich/water interfacial CG simulations. Together, these results support the proposed transition from homogeneous aqueous self-assembly to TOP-rich/water interface-mediated anisotropic organization.**

Taken together, the initial emulsion observation, TOP-free homogeneous assembly, TOP-dependent structural evolution, time-dependent intermediate characterization, pre-carbonization polymer precursor morphology, oil-phase control experiments and actual-ratio TOP-rich/water interfacial simulations now provide mutually consistent evidence for the transition from homogeneous aqueous self-assembly to TOP-rich/water interface-mediated anisotropic organization. We have revised the manuscript to present this transition more cautiously as a mechanism supported by complementary

experimental and computational evidence, rather than as a directly visualized real-time single-micelle migration process. We hope that these additions and revisions adequately address your concern regarding micelle localization, assembly dynamics and interfacial adsorption processes.

Figure-to-conclusion correspondence for your Comment 2:

Conclusion or mechanistic point	Supporting figure/table	How the figure supports the conclusion
The actual-ratio long-box models form stable TOP-rich slabs.	Table S5; Fig. S53a-b	Both $Ea1_{TOP40}$ and $Ea2_{TOP120}$ show $n_{TOP\ clusters}$=1 and largest TOP-cluster fraction=1.0 over the final 2 ns.
Increasing TOP content shifts F127/LWOH components toward the TOP-rich interfacial region.	Fig. S53e-f; Table S5	z-density profiles and z-mean values show that PPO is positioned closer to the TOP-rich slab in $Ea2_{TOP120}$ than in $Ea1_{TOP40}$.
High TOP content suppresses PPO-water contact and promotes PPO relocation toward the TOP-rich slab.	Fig. S53g,h; Table S5	PPO-water contact density decreases from 1.33 to 0.081 contacts/bead from $Ea1_{TOP40}$ to $Ea2_{TOP120}$.
High TOP content induces stronger in-plane anisotropic organization of the F127/LWOH aggregate.	Fig. S53i-j; Table S5	Aggregate S shifts from -0.275 to -0.476 and F127 S shifts from -0.071 to -0.441, approaching the ideal in-plane value of S=-0.5.

Added text in the revised supplementary information:

1.6 Actual-ratio long-box TOP-rich/water interfacial simulations for validating TOP-induced interfacial localization and anisotropic organization

1.6.1 Model construction and simulation protocol

To further validate the proposed transition from homogeneous hydrated association to TOP-rich/water interfacial organization, actual-ratio long-box interfacial CG models were constructed using the same molecular compositions as the corresponding P-series simulations. The low-TOP model, denoted as Ea1TOP40, contained 16 F127M molecules, 40 TOP molecules, 9000 ETHX beads, 36538 water beads and 120 LWOH molecules. The high-TOP model, denoted as Ea2TOP120, contained 16 F127M molecules, 120 TOP molecules, 9000 ETHX beads, 36538 water beads and 120 LWOH molecules. Thus, the two models differed only in the TOP content, allowing a controlled comparison of TOP-content-dependent interfacial organization.

The simulation box was constructed with a reduced interfacial area and elongated z dimension of 10×10×60 nm to increase the effective TOP surface density and stabilize a TOP-rich slab. The F127/LWOH components were initially placed near the TOP-rich/water interfacial region to examine interfacial adsorption, segmental partitioning and anisotropic organization. After energy minimization, NVT equilibration and z-only semi-isotropic NPT equilibration, 20 ns production simulations were performed. The x and y dimensions were kept fixed to maintain the interfacial area, while the z dimension was allowed to relax during NPT and production simulations. The last 2 ns of each trajectory were used for quantitative analysis.

1.6.2 Analysis descriptors

TOP-rich slab stability was evaluated by calculating the number of TOP clusters and the fraction of TOP beads in the largest TOP cluster using a 0.8 nm cutoff. Segment-resolved contact analysis was performed between PEO, PPO or LWOH and TOP, water or ETHX using the same cutoff. The normalized contact density was obtained by dividing the total number of contacts by the number of corresponding beads. z-directional normalized density profiles were calculated over the final 2 ns to examine the spatial distributions of TOP, PEO, PPO and LWOH along the interface normal. Shape and orientation descriptors were calculated from the eigenvalues and eigenvectors of the gyration tensor. The axis1/axis3 ratio and relative shape anisotropy κ2 were used to describe elongation and shape anisotropy, respectively. The orientation parameter S was calculated relative to the z-axis, which corresponds to the normal direction of the TOP-rich/water interface. S=-0.5 represents ideal in-plane alignment, S=0 represents random orientation and S=1 represents perpendicular alignment.

1.6.3 TOP-slab stability and segment-selective interfacial anchoring

The quantitative descriptors summarized in Supplementary Table 5 first confirm that both actual-ratio long-box models maintained a stable TOP-rich slab over the final 2 ns. In both Ea1TOP40 and Ea2TOP120, the number of TOP clusters remained 1 and the largest TOP-cluster fraction remained 1.0. Therefore, the difference between the two systems mainly reflects the TOP-content-dependent interfacial coupling strength rather than the presence or absence of a TOP-rich slab.

The normalized contact densities summarized in Supplementary Table 5 and plotted in Supplementary Fig. 53 show that increasing the TOP content from 40 to 120 molecules substantially enhanced the segment-selective anchoring of F127. The normalized PPO-TOP contact density increased from 2.27 to 8.48 contacts per bead, whereas the normalized PEO-TOP contact density increased from 0.51 to 0.97 contacts per bead. Therefore, PPO-TOP contacts were much stronger than PEO-TOP contacts in both models, confirming that TOP preferentially anchors the PPO hydrophobic segment rather than uniformly interacting with the whole F127 chain. Meanwhile, the normalized PPO-water contact density decreased sharply from 1.33 to 0.081 contacts per bead, indicating that TOP enrichment effectively excludes PPO from the aqueous region and drives PPO toward the TOP-rich slab.

1.6.4 Spatial distribution and anisotropic interfacial organization

The z-directional normalized density profiles shown in Supplementary Fig. 53e,f further confirm the TOP-content-dependent interfacial localization. In Ea1TOP40, the mean z positions of PEO and PPO were 34.69, 34.71 nm, respectively, which were below the TOP-rich slab centered at 35.63 nm. In Ea2TOP120, the corresponding meaning z positions shifted to 36.17 and 36.15 , respectively, close to the TOP-rich slab centered at 35.96 nm.

The shape and orientation descriptors summarized in Supplementary Table 5 and shown in Supplementary Fig. 53i,j further support the TOP-induced anisotropic interfacial organization. The aggregate axis1/axis3 ratio increased from 1.96 in Ea1TOP40 to 2.30 in Ea2TOP120, while the relative shape anisotropy kappa2 increased from 0.141 to 0.272. More importantly, the aggregate orientation parameter shifted from -0.275 to -0.476, approaching the theoretical value of -0.5 for ideal in-plane alignment. A similar trend was observed for F127 chains: the F127 orientation parameter shifted from -0.071 in Ea1TOP40 to -0.441 in Ea2TOP120. These results demonstrate that TOP enrichment

enhances PPO-TOP anchoring and induces in-plane anisotropic growth of the aggregate at TOP-rich/water interface.

It should be noted that the long-box interfacial models were designed to validate interfacial adsorption and anisotropic organization under actual formulation ratios. Because the F127/LWOH components were initially placed near the TOP-rich/water interfacial region, these simulations are interpreted as direct evidence for interfacial coupling, segmental partitioning and anisotropic organization, rather than as a measurement of the long-range migration kinetics from a far-away bulk aqueous phase.

The correspondence between the conclusions, Supplementary Table 5 and Supplementary Fig. 53 is therefore as follows: TOP-cluster descriptors support slab stability and contact-density descriptors support PPO-selective anchoring ; z-density profiles support spatial localization; and shape/orientation descriptors support in-plane anisotropic organization.

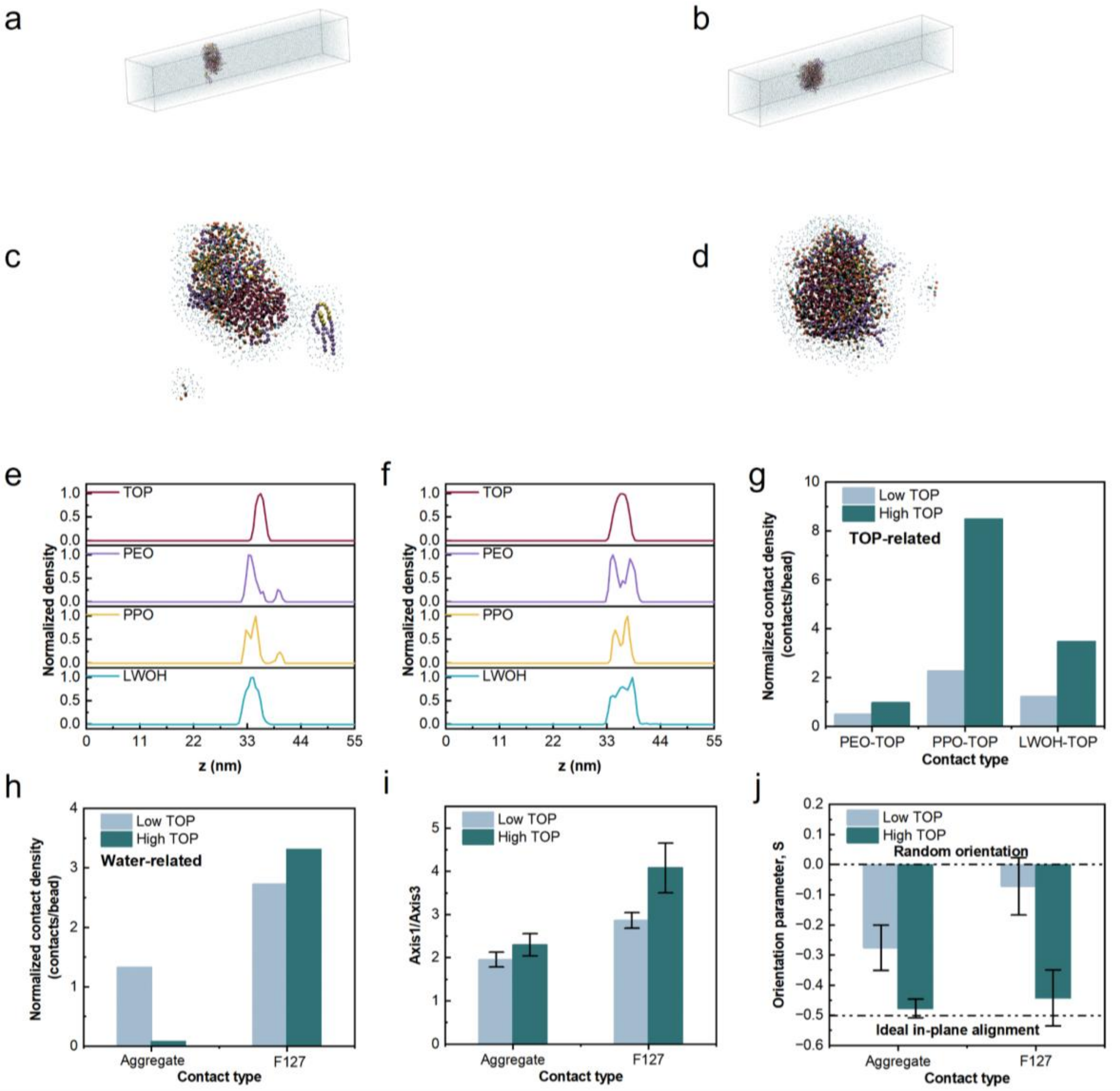

Supplementary Fig. 53. Actual-ratio long-box TOP-rich/water interfacial CG simulations validating TOP-content-dependent interfacial localization and anisotropic organization. (a, b) Representative full-system snapshots of $Ea1_{TOP40}$ and $Ea2_{TOP120}$, respectively. (c, d) Local interfacial views of the corresponding systems. Water beads were omitted or shown only as a dilute background for clarity. The representative CG snapshots shown in Supplementary Fig. 53a-d are consistent with

the quantitative descriptors summarized in Supplementary Table 5, showing stronger interfacial coupling in Ea2$_{TOP120}$ than in Ea1$_{TOP40}$. (e, f) z-directional normalized density profiles of TOP, PEO, PPO and LWOH in Ea1$_{TOP40}$ and Ea2$_{TOP120}$. (g) Normalized contact densities between PEO, PPO or LWOH and TOP. (h) Normalized water-contact densities. (i) Axis1/axis3 ratios of the F127/LWOH aggregate and F127 chains. (j) Orientation parameter S of the aggregate and F127 chains relative to the normal interface. S=-0.5 indicates ideal in-plane alignment, S=0 indicates random orientation and S=1 indicates perpendicular alignment. Error bars represent the standard deviation over the final 2 ns.

Supplementary Table 5. Quantitative descriptors from actual-ratio long-box TOP-rich/water interfacial CG simulations.

Descriptor	Ea1$_{TOP40}$	Ea2$_{TOP120}$
$n_{TOP\ clusters}$	1.0	1.0
Largest TOP-cluster fraction	1.0	1.0
PEO-TOP contacts/bead	0.507	0.974
PPO-TOP contacts/bead	2.270	8.484
LWOH-TOP contacts/bead	1.220	3.477
PEO-water contacts/bead	3.438	3.309
PPO-water contacts/bead	1.327	0.081
LWOH-water contacts/bead	2.728	3.314
TOP z_{mean} (nm)	35.63	35.96
PEO z_{mean} (nm)	34.69	36.17
PPO z_{mean} (nm)	34.71	36.15
LWOH z_{mean} (nm)	34.01	36.46
Aggregate axis1/axis3	1.96 ± 0.17	2.30 ± 0.26
Aggregate κ^2	0.141 ± 0.028	0.272 ± 0.072
Aggregate S	-0.275 ± 0.075	-0.476 ± 0.031
F127 axis1/axis3	2.87 ± 0.18	4.08 ± 0.57
F127 κ^2	0.398 ± 0.069	0.446 ± 0.063
F127 S	-0.071 ± 0.095	-0.441 ± 0.093

The values were averaged over the final 2 ns of the 20 ns production simulations. The normalized contact density was calculated by dividing the total number of contacts by the number of corresponding beads. The TOP-rich slab was identified using a 0.8 nm cutoff for TOP bead clustering. The descriptors listed in Supplementary Table 5 correspond to the structural snapshots, density profiles, contact-density plots and shape/orientation analyses shown in Supplementary Fig. 53.

Revised text in manuscript:

Revised text ① (Page 2, line 26): This synthesis features the introduction of trioctyl phosphate (TOP) as a hydrophobic swelling agent for composite micelles and an interfacial modifier, shifting the preferred micelle curvature/packing tendency and biasing the assembly pathway from homogeneous aqueous self-assembly toward interface-associated anisotropic organization. This coupled regulation progressively reduces the density of ordered mesochannels within the fibrous framework and promotes the evolution from ordered mesoporous nanofibers to hollow nanofibers and bowl-like architectures.

Revised text ② (Page 3, line 54): oil molecules can simultaneously swell the hydrophobic blocks of micelles and construct oil/water interfaces for the self-assembly and growth process of micelles[25–30]. The relative affinity among precursor species, surfactant micelles, oil phase and solvent can be described by an effective interfacial-energy/interfacial-affinity framework, which is useful for rationalizing whether composite micelles preferentially remain in the bulk aqueous phase or become enriched at oil/water interfaces[31–36]. Although packing-parameter regulation and interfacial micelle assembly have been widely used in soft-template synthesis, most reported systems rely on model synthetic precursors such as dopamine or other well-defined monomers[37–40]. In these systems, the carbon precursor generally serves mainly as a framework-forming component, whereas the simultaneous programming of external morphology and internal mesopore architecture from a renewable biomass-derived precursor remains rarely explored[41–46]. Therefore, establishing a reactive emulsion platform in which biomass can participate in micelle organization, interfacial localization and polymerization-induced structural fixation would provide a distinct route beyond conventional homogeneous soft-template or droplet-directed assembly.

Revised text ③ (Page 4, line 82): we develop a reactive-emulsion-mediated self-assembly platform to synthesize mesoporous carbon architectures from liquefied wood. By systematically varying the TOP and ethanol content, we gain coupled control over micelle curvature and interfacial interactions, biasing the self-assembly pathway from homogeneous toward interface-associated anisotropic self-assembly of composite micelles and enabling the evolution of carbon nanofibers from parallel-ordered channels to hollow architectures.

Revised text ④ (Page 10, line 182): the assembly mainly proceeds through a homogeneous aqueous pathway without an oil phase (Supplementary Fig. 7a,b). After TOP was introduced, the system evolved from a single aqueous assembly environment into a reactive emulsion containing oil/water interfaces. Upon gradually increasing the TOP amount from 0.05 to 0.3 mL, the macroscopic product changed from soft and collapsible columns to a self-supporting monolith, and finally to compact blocks deposited at the bottom

Revised text ⑤ (Page 13, line 246): which is probably because excessive TOP disrupts the kinetic matching between micelle organization and precursor condensation under low-ethanol conditions.

Revised text ⑥ (Page 15, line 287): These results define an experimentally sampled EtOH–TOP composition window in which ethanol mainly shifts the system from anisotropic fibrous/hollow

architectures toward isotropic or quasi-isotropic nanospheres, while TOP further regulates the internal pore architecture and surface mesostructure within each morphology regime.

Revised text ⑦ (Page 22, line 419): The use of different trialkyl phosphates led to distinct mesostructural evolution, which may be attributed to the fact that longer alkyl-chain phosphate oils strengthen PPO/oil association and improve compatibility with the hydrophobic/aromatic fragments of LWMF, thereby increasing the tendency of composite micelles to organize at oil/water interfaces. At the same time, stronger hydrophobic swelling shifts the preferred micelle curvature toward lower-curvature configurations, promoting hollow or multi-chambered architectures. In contrast, shorter-chain oils provide weaker hydrophobic/interfacial interactions and retain a more homogeneous assembly pathway. These oil-phase controls demonstrate that the transition is not caused solely by the chemical identity of TOP, but by the combined hydrophobic swelling ability and interfacial affinity of the oil phase. Longer alkyl-chain phosphate oils strengthen PPO/oil association and interfacial coupling, thereby favoring anisotropic or hollow architectures, whereas shorter-chain oils provide weaker hydrophobic/interfacial interactions and retain a more homogeneous assembly pathway.

Revised text ⑧ (Page 32, line 611): Interfacial simulation models further show that F127/TOP/LWOH aggregates organize near the TOP-rich oil/water interfacial region, where TOP/PPO-rich domains provide hydrophobic anchoring while PEO/LWOH hydroxyl-rich regions maintain polar contacts with the solvent (Supplementary Table 5). These results support the interpretation that increasing TOP content enhances interfacial coupling and anisotropic organization of F127/resin/TOP micellar domains (Supplementary Fig. 53).

Comment 3: The introduction of Δσ as a governing parameter is conceptually reasonable, but currently unsupported, because no interfacial tension measurements are provided and the correlation between Δσ and structural transitions is unclear.

Response: We sincerely thank you for this important and constructive comment. We fully agree that, in the original manuscript, the physical meaning of Δσ and its correlation with the observed structural transitions were not described in sufficient detail. We also appreciate your recognition that introducing Δσ as an interfacial-energy parameter is conceptually reasonable. In the revised manuscript, we have revised the discussion to clarify what Δσ represents in our dynamic multicomponent assembly system, why it is appropriate to use Δσ as an effective descriptor, and how the experimentally observed

structural transitions correlate with the proposed Δσ-mediated interfacial assembly pathway.

The Δσ discussed in our manuscript does not refer to the static interfacial tension of a simple binary liquid/liquid interface, such as pure TOP/water or TOP/ethanol–water. Instead, Δσ represents the effective change in interfacial energy associated with the adsorption, nucleation, and anisotropic growth of F127/resin/TOP co-assembled micelles at the TOP-containing oil/solvent interface. **During this process, the original oil/solvent interface is partially replaced by two newly generated interfaces, namely the micelle/resin–solvent interface and the micelle/resin–oil interface.** Therefore, in the revised manuscript, we define this term more explicitly as an effective interfacial-energy change:

$$\Delta\sigma_{eff}=\sigma_{M-S}^{eff}+\sigma_{M-O}^{eff}-\sigma_{O-S}^{eff}$$

where M denotes the F127/resin/TOP co-assembled micelles, O denotes the TOP-rich oil phase, and S denotes the ethanol/water solvent phase. Here, σ_{M-S}^{eff}, σ_{M-O}^{eff}, and σ_{O-S}^{eff} represent the effective micelle/resin–solvent, micelle/resin–oil, and oil–solvent interfacial-energy terms, respectively. A positive $\Delta\sigma_{eff}$ favors spreading or more homogeneous isotropic assembly, whereas a more negative or less positive $\Delta\sigma_{eff}$ disfavors complete spreading and promotes localized island-like nucleation and anisotropic growth at the interface.

This Δσ-based treatment is not unique to this system, but follows an interfacial-energy framework used in related interfacial micelle assembly systems established in our and Zhao's previous work. For example, Zhao's group used an interfacial-energy-mediated model to explain the transition of micelle self-assembly from interface-layer assembly to interface-island assembly and then to interface-separated assembly by changing the ethanol/water volume fraction, in which Δσ was used to describe the energetic balance among the PDA/solvent, PDA/TMB, and TMB/solvent interfaces, and the morphology transition was correlated with the ethanol-dependent change in this effective interfacial-energy balance (*J. Am. Chem. Soc*. 2022, 54). Similarly, they proposed that the droplet-directed anisotropic assembly of semifootball-like carbon nanoparticles is governed by the interfacial-energy balance of the emulsion system; by changing oil-phase polarity, TMB content, and water/oil ratio, morphology evolves from nonporous spheres to anisotropic semifootball-like nanoparticles and further to channel-containing structures (*Adv. Funct. Mater.* 2024 2400503). Beyond these carbon-based systems, Zhao further developed engine–trailer-structured mesoporous nanotrucks through an

interfacial-energy-mediated anisotropic growth strategy and reported the anisotropic island nucleation and growth of dual-compartment Janus mesoporous silica nanoparticles, which has been summarized as a representative example of interfacial-energy-mediated anisotropic growth of composite micelles (*J. Am. Chem. Soc.* 2014, 15086; *Chem* 2020, 1097). All these precedents support the use of Δσ as an effective thermodynamic descriptor for dynamic interfacial micelle assembly and anisotropic domain growth, although the individual interfacial-energy terms are difficult to measure independently under reactive polymerization conditions.

We also agree with you that direct interfacial tension measurements were not provided in the original manuscript. However, in our system, direct in situ visualization of transient TOP/F127/LWMF composite micelles during hydrothermal treatment is technically challenging. Moreover, the assembly process involves several coupled and time-dependent events: partitioning of TOP into the hydrophobic PPO domains of F127 micelles, adsorption of F127/resin/TOP co-assembled domains at the oil/solvent interface, ethanol-dependent solvation of both TOP and F127, and progressive resin polymerization/cross-linking. **A pendant-drop or spinning-drop measurement of the initial TOP/ethanol–water interface would mainly probe σ_{O-S}^{eff}, but it would not directly capture σ_{M-S}^{eff}or σ_{M-O}^{eff}, which are generated only after the micelle/resin/TOP assemblies nucleate and reorganize at the interface.** Therefore, we did not present Δσ as an independently measured static interfacial tension. We have revised the manuscript to define it explicitly as an effective interfacial-energy change associated with the dynamic replacement of the oil/solvent interface by micelle/resin-containing interfaces.

Different experimental variables contribute to $\Delta\sigma_{eff}$ through different interfacial terms. For the TOP-dosage-dependent experiments, TOP partitions into the hydrophobic PPO domains of F127, increases the TOP-associated hydrophobic volume inside the F127/resin/TOP co-assembled micelle domains, and strengthens PPO–TOP contacts, which increase the compatibility between the micelle domains and the TOP-rich oil phase, thereby decreasing σ_{M-O}^{eff}. At the same time, TOP incorporation increases the hydrophobic character and swelling degree of the micelle domains, making full exposure of PPO/TOP-rich regions to the ethanol/water phase less favorable; this changes σ_{M-S}^{eff} by increasing the energetic penalty for micellar domains to remain fully dispersed in the aqueous-rich continuous

phase. Therefore, the TOP-dependent morphology evolution is mainly attributed to a decrease in σ_{M-O}^{eff} relative to σ_{M-S}^{eff}, which promotes preferential localization of F127/resin/TOP micellar domains at the TOP-rich interface. This is consistent with the observed transition from poorly developed or more homogeneous micellar products under TOP-deficient conditions to well-defined hollow carbon nanofibers under optimized TOP-containing conditions. When the TOP/micelle balance is further perturbed, excessive swelling or altered micellar organization can disrupt the optimized interfacial localization and anisotropic growth, leading to further morphology variation.

For the ethanol-content-dependent experiments, ethanol directly changes the solvent composition and therefore has a strong influence on σ_{O-S}^{eff}. Increasing ethanol content improves the compatibility between the hydrophobic TOP-rich phase and the surrounding ethanol/water medium, thereby decreasing σ_{O-S}^{eff}. Ethanol also changes σ_{M-S}^{eff}, because it modifies the solvation environment of the PEO and PPO blocks of F127 and alters the stability of F127/resin/TOP micellar domains in the continuous phase. At moderate ethanol content, the decrease in σ_{O-S}^{eff} reduces the excessive oil/solvent interfacial penalty and allows controlled interfacial nucleation. Meanwhile, the micelles still maintain sufficient hydrophobic segregation and local packing to support anisotropic growth. Thus, ethanol mainly decreases σ_{O-S}^{eff} and simultaneously alters σ_{M-S}^{eff}, explaining why ethanol variation can shift the system between dispersed or weakly anisotropic assembly, optimized interfacial anisotropic growth, and disrupted micellar organization.

To further strengthen the correlation between $\Delta\sigma_{eff}$ and the structural transitions, we added a more detailed discussion based on systematic control experiments. The interfacial simulations provide molecular-level support for this descriptor by examining whether TOP/F127/LW-derived precursor assemblies preferentially localize and organize at the TOP/water interface under different formulation conditions. These simulations show that TOP promotes the enrichment of composite micelle/precursor species at the oil/water interface and supports their anisotropic interfacial organization, providing a direct mechanistic link between the proposed decrease in effective interfacial penalty and the experimentally observed transition from homogeneous micellar products to hollow carbon nanofibers. In our synthesis, TOP content and ethanol content both regulate the effective interfacial-energy balance, but through different physical pathways. Increasing TOP content enhances the interaction between the

hydrophobic PPO domains of F127 and the TOP-rich oil phase, thereby promoting the localization of F127/resin/TOP assemblies at the interface. Experimentally, this is reflected by the transition from homogeneous micelle products to hollow carbon nanofibers under optimized TOP-containing conditions. Excessive perturbation of the oil/micelle balance disrupts the optimized anisotropic assembly and leads to further morphological variation. These results indicate that TOP regulates both the effective interfacial affinity between the micellar assemblies and the oil-rich interface and acting as swelling agent. Ethanol provides another important handle for modulating $\Delta\sigma_{eff}$. Ethanol modifies the compatibility between TOP and the surrounding solvent, and influences the stability and localization of micelles at the oil/solvent interface. Increasing ethanol content decreases σ_{O-S}^{eff} by improving oil/solvent compatibility. This interpretation is consistent with the experimentally observed ethanol-dependent morphology evolution (from hollow nanofiber to mesoporous sphere).

Accordingly, we have revised the manuscript in three ways. First, we have added a clear definition of Δσ as an effective interfacial-energy change, $\Delta\sigma_{eff}$, associated with dynamic F127/resin/TOP micellar assembly at the TOP-containing interface. Second, we have added literature precedents for the use of Δσ-based models in liquid–liquid interfacial micelle assembly systems. Third, we have expanded the discussion of TOP-, ethanol-, oil-chain-, and F127-dependent control experiments, together with the coarse-grained simulation results, to clarify the correlation between $\Delta\sigma_{eff}$-mediated interfacial localization and the observed structural transitions. We hope that these revisions address your concern.

Revised text in manuscript (Page 28, line 535)**:**

In this system, TOP partitions into the hydrophobic PPO-rich domains of F127-based composite micelles and acts as a hydrophobic swelling agent. Besides, it forms a TOP-rich oil phase and creates oil/solvent interfacial regions for precursor–template organization. F127, TOP and LWMF species are proposed to form F127/resin/TOP co-assembled micellar domains. The coexistence of nanoscale micellar species and micron-sized TOP-rich droplets provide both bulk solvent domains and TOP-rich oil/solvent interfacial regions for assembly. Depending on the formulation, these composite micelle domains can mainly organize in the continuous ethanol/water solvent phase or become enriched near the TOP-rich oil/solvent interface. To describe the competition between homogeneous aqueous assembly and interface-associated organization, we use an effective interfacial-energy/interfacial-

affinity descriptor:

$$\Delta\sigma_{eff}=\sigma_{M-S}^{eff}+\sigma_{M-O}^{eff}-\sigma_{O-S}^{eff}$$

where M denotes the F127/resin/TOP co-assembled micellar domain, O denotes the TOP-rich oil phase, and S denotes the ethanol/water solvent phase. Here, σ_{M-S}^{eff}, σ_{M-O}^{eff} and σ_{O-S}^{eff} represent the effective micelle/resin–solvent, micelle/resin–oil and oil–solvent interfacial-energy terms, respectively. This descriptor does not represent the static interfacial tension of a simple binary liquid/liquid interface. Instead, it describes the effective interfacial-energy change associated with the dynamic replacement of the original oil/solvent interface by micelle/resin-containing interfaces during reactive emulsion assembly. In this framework, a positive $\Delta\sigma_{eff}$ favors spreading or more homogeneous isotropic assembly, whereas a more negative or less positive $\Delta\sigma_{eff}$ disfavors complete spreading and promotes localized island-like nucleation and anisotropic growth at the interface. Increasing TOP content strengthens PPO–TOP and resin/TOP hydrophobic interactions, thereby improving the compatibility between the F127/resin/TOP micellar domains and the TOP-rich oil phase and decreasing σ_{M-O}^{eff}. At the same time, TOP incorporation increases the hydrophobic character and swelling degree of the micellar domains, making full exposure of PPO/TOP-rich regions to the ethanol/water solvent less favorable and altering σ_{M-S}^{eff}. The relative decrease in σ_{M-O}^{eff} with respect to σ_{M-S}^{eff} promotes the enrichment of F127/resin/TOP micellar domains at the TOP-rich interfacial region, thereby facilitating interface-associated anisotropic organization. This interpretation is consistent with the TOP-dependent morphology and pore-architecture evolution. In the absence of TOP, the system lacks a TOP-rich oil phase and mainly follows a homogeneous soft-template assembly pathway in the solvent phase, producing three-dimensional particles with ordered mesopores. Introducing a small amount of TOP creates a reactive emulsion while maintaining a strong contribution from homogeneous micelle organization, leading to ordered mesoporous nanofibers with multiple parallel mesochannels. Increasing TOP content enhances hydrophobic swelling and strengthens the contribution of interface-associated organization, which reduces the density of ordered mesochannels and promotes the formation of hollow carbon nanofibers. At higher TOP content, excessive swelling or an altered oil/micelle balance further changes the interfacial assembly behavior, giving rise to bowl-like nanospheres or irregular lamellar aggregates.

Importantly, this interfacial-affinity balance contributes to the assembly-locus selection by biasing where micelle organization preferentially occurs, but does not independently determine the final morphology and mesostructure. Micelle packing provides another useful but qualitative framework for understanding the internal pore-architecture evolution. In the present F127/TOP/ethanol/water/LWMF reactive system, a single classical packing parameter $p = V/a_0l_c$ cannot be unambiguously calculated because the hydrophobic volume, effective headgroup area and hydrophobic chain length are all formulation-dependent and dynamically altered during TOP partitioning, ethanol solvation and resin polymerization. Therefore, the packing-parameter concept is used here as a qualitative micelle-curvature framework rather than a quantitatively predictive parameter. TOP incorporation into PPO-rich domains increases the effective hydrophobic volume and favors lower-curvature micellar organizations. The observed evolution from ordered channels to hollow, curved and lamellar-like architectures is therefore consistent with a formulation-dependent shift in preferred micelle curvature/packing tendency.

Ethanol further regulates the assembly pathway by modifying both the oil/solvent interfacial term and the micelle/solvent interactions. Increasing ethanol content improves the compatibility between the oil phase and the surrounding ethanol/water medium, thereby decreasing $\Delta\sigma_{eff}$. Ethanol also alters σ_{M-S}^{eff} by changing the solvation environment of PEO and PPO blocks and the stability of F127/resin/TOP micellar domains in the continuous phase. At moderate ethanol content, the reduced oil/solvent interfacial penalty and retained hydrophobic segregation allow controlled interfacial nucleation and anisotropic growth. A higher ethanol content improved solvent compatibility and enhanced micelle solvation weaken the contribution of interface-associated anisotropic organization and shift the products toward isotropic or quasi-isotropic nanospheres.

Comment 4: The manuscript emphasizes "continuous structural evolution" within a single system. However, no continuous mapping or phase diagram is provided. Moreover, the transition boundaries are not defined.

Response: We sincerely thank you for this important and constructive comment. **To address this concern, we have added a systematic morphology/mesostructured map based on the synthetic matric summarized in Supplementary Table 1.**

The products are classified into four major experimentally distinguishable structural regimes: irregular/particle-like structures, fiber/rod-like structures, hollow/curved structures, and spherical-like architectures. The sphere-like region includes several subtypes with different internal or surface mesostructures, such as mesoporous nanospheres, golf ball-like nanospheres, multichambered nanospheres, plum blossom-like nanospheres, branched nanospheres and gear-like nanospheres. We intentionally treat these sphere-based products as one broad structural regime in the main morphology map, because they share an isotropic or quasi-isotropic spherical external morphology, while their internal mesostructural differences are further distinguished in a separate high-ethanol map.

The newly added TOP–EtOH map serves as the primary composition–structure map because TOP and ethanol are the two most important variables controlling the main anisotropic-to-spherical structural transition This map directly shows the structural evolution across the main synthetic parameter space. At low ethanol content and low TOP amount, the products mainly appear as fiber/rod-like structures. Increasing TOP content under low-to-moderate ethanol conditions drives the system toward hollow/curved architectures. Further increasing ethanol content shifts the products into the sphere-like region. Therefore, the structural evolution from fiber/rod-like structures to hollow/curved structures and then to sphere-like architectures is now explicitly mapped rather than inferred from isolated examples. The transition boundaries are also defined from the experimental map. The boundary between the fiber/rod-like region and the hollow/curved region occurs at intermediate TOP content, especially around the condition where the fibrous morphology is retained but the internal mesostructure evolves from ordered mesochannels to hollow or curved architectures. The boundary between isotropic nanofibers and anisotropic assembly is controlled by TOP content. The boundary between anisotropic fibrous/hollow structure and sphere-like architecture is mainly controlled by ethanol content. Increasing ethanol content shifts the dominant assembly from anisotropic fibrous or hollow architectures toward isotropic nanospheres. These boundaries are indicated as empirical transition regions in the morphology map.

To clarify that the observed structural evolution is not controlled only by TOP and ethanol, we further constructed additional parameter-section maps. The TOP–temperature maps at EtOH = 10 mL and EtOH = 25 mL show that temperature changes the accessible structural window. At EtOH = 10 mL, low temperature leads to irregular/particle-like products, an appropriate temperature enables fiber/rod-like or hollow/curved structures depending on TOP content, whereas excessive temperature

can again disrupt the ordered assembly pathway. In contrast, at EtOH = 25 mL, the products remain within the sphere-like regime over a broad temperature range, but the detailed sphere-like morphology varies from mesoporous nanospheres to golf ball-like, multichambered, branched or gear-like nanospheres. This result further confirms that TOP and ethanol content determines the main anisotropic-to-spherical transition, while temperature regulates the kinetic accessibility and detailed mesostructure within the selected structural regime.

The TOP–F127 maps at EtOH = 10 mL and EtOH = 25 mL further define the role of the soft template. At EtOH = 10 mL, increasing TOP content shifts the morphology from fiber/rod-like structures to hollow/curved structures when sufficient F127 is present. Insufficient F127 leads to irregular/particle-like products, confirming that F127 is necessary for constructing ordered soft-template mesostructures. At EtOH = 25 mL, the system is biased toward sphere-like architectures, while F127 amount controls whether the products are well-developed or become irregular. These maps show that F127 defines the soft-template assembly window, whereas TOP and ethanol determine the dominant structural regime.

The HCl–EtOH map at fixed TOP = 0.2 mL further shows that the transition boundaries are also affected by polymerization kinetics. At low ethanol content, increasing HCl amount drives the products from 1D fiber/rod-like structures to hollow/curved structures and then to irregular/particle-like products, indicating that excessive polymerization disrupts the kinetic matching between micelle assembly and precursor fixation. At high ethanol content, the products remain mainly in the sphere-like regime, except under overly strong acid conditions, where irregular structures appear. This result confirms that the morphology map is governed by coupled interfacial assembly, micelle packing and polymerization kinetics.

We would like to show that this newly added map is not intended to be a strict equilibrium thermodynamic phase diagram. To avoid overstatement, we have revised the wording in the manuscript. The structural evolution is now described as a progressive and experimentally mapped morphology evolution across the sampled synthetic parameter space, rather than as a mathematically continuous phase transition. The present system is a reactive emulsion-mediated self-assembly system involving emulsion formation, micelle organization, LWMF polymerization, interfacial adsorption and hydrothermal fixation. Therefore, the boundaries should be regarded as experimentally sampled transition regions rather than infinitely sharp equilibrium phase boundaries. Nevertheless, the map now clearly defines the parameter windows for obtaining irregular/particle-like products, 1D fiber/rod-like

structures, hollow/curved architectures and sphere-like architectures.

Revised text (Page 32, line 623)**:** The morphology/mesostructure maps further clarify the structural windows accessible in this reactive emulsion system (Supplementary Fig. 55). The products can be classified into irregular/particle-like products, fiber/rod-like structures, hollow/curved architectures and sphere-like architectures. In the TOP–EtOH map, low-EtOH/low-TOP conditions mainly favor fiber/rod-like structures, intermediate TOP promotes hollow/curved architectures, whereas high ethanol content shifts the products toward sphere-like morphologies. These boundaries are empirical transition regions inferred from adjacent experimental points, rather than sharp equilibrium phase boundaries, because the present system involves coupled emulsion formation, micelle organization, precursor condensation and hydrothermal fixation. Additional TOP–temperature, TOP–F127 and HCl–EtOH maps further show that temperature, template concentration and polymerization kinetics regulate the accessibility and stability of each structural regime.

Added Supplementary Figures

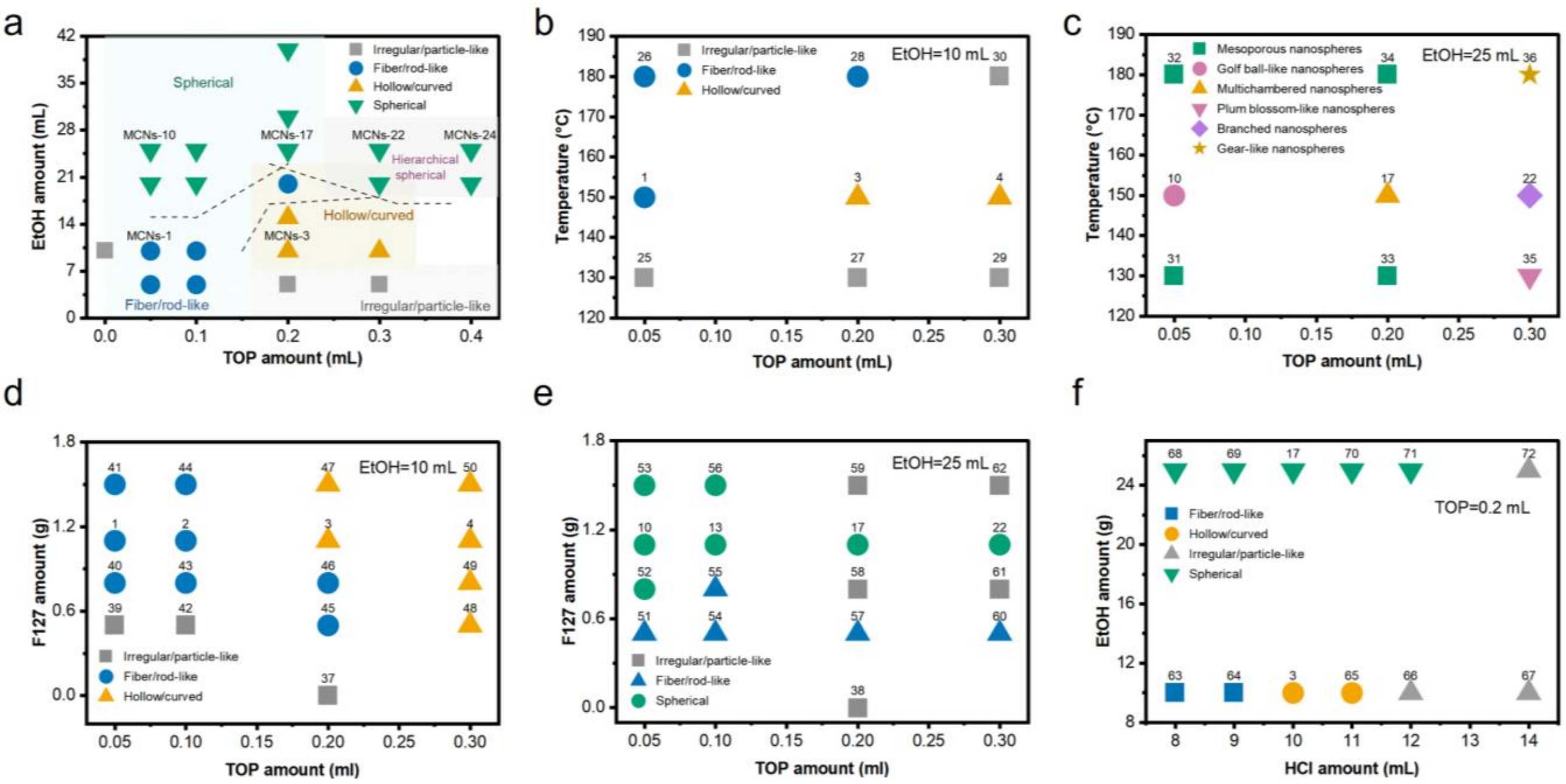

Supplementary Fig. 55. Experimental formulation-morphology map of MCNs. (a) TOP-EtOH morphology map, (b) TOP-temperature map at EtOH=10 mL, (c) TOP-temperature map at EtOH=25 mL, (d) TOP-F127 map at EtOH=10 mL, (e) TOP-F127 map at EtOH=25 mL, (f) HCl-EtOH map at TOP=0.2 mL. Each point represents an experimentally synthesized sample, and colors denote different morphology categories. Dashed boundaries indicate approximate transition zones inferred from adjacent experimental points rather than thermodynamic phase boundaries.

Comment 5: The use of liquefied wood is presented as a key feature, yet its functional role is not clearly demonstrated. Does it influence self-assembly kinetics? Does it alter interfacial interactions? Is it essential for achieving the reported structures?

Response: We sincerely thank you for this important and constructive question. As one of the key innovations, we agree that the functional role of liquefied wood should be more clearly demonstrated. Liquefied wood is not merely a carbon source; rather, wood-derived LWMF acts as a framework-forming carbon precursor, an interfacial participating precursor, and a polymerization-fixation component that collectively enable the formation of mesoporous carbon architectures.

First, **liquefied wood participates in the polymerization-coupled self-assembly process.** The liquefaction of wood converts wood into a mixture of phenolic/aromatic fragments and polydroxy oligomers, containing abundant hydroxyl groups and reactive aromatic sites, which can react with formaldehyde and melamine to form liquefied wood-melamine-formaldehyde resins (LWMF). LWMF is incorporated into the composite micelles through precursor–template interactions, including hydrogen bonding, hydrophobic association, and interfacial compatibility. Therefore, liquefied wood forms the final carbon framework after hydrothermal polymerization and carbonization. Without such a polymerizable precursor, the oil/surface soft template would not be converted into a stable mesoporous carbon architecture. The TEM image of products (as follows) obtained from the additional experiment without adding the liquefied wood shows only solid carbon spheres are exist, which is due to the fact that, unlike larch wood phenol liquefies, which have a large number of hydroxyl groups, the triazine group of melamine interacts weakly with F127 and cannot form composite micelles and undergo the self-assembly process.

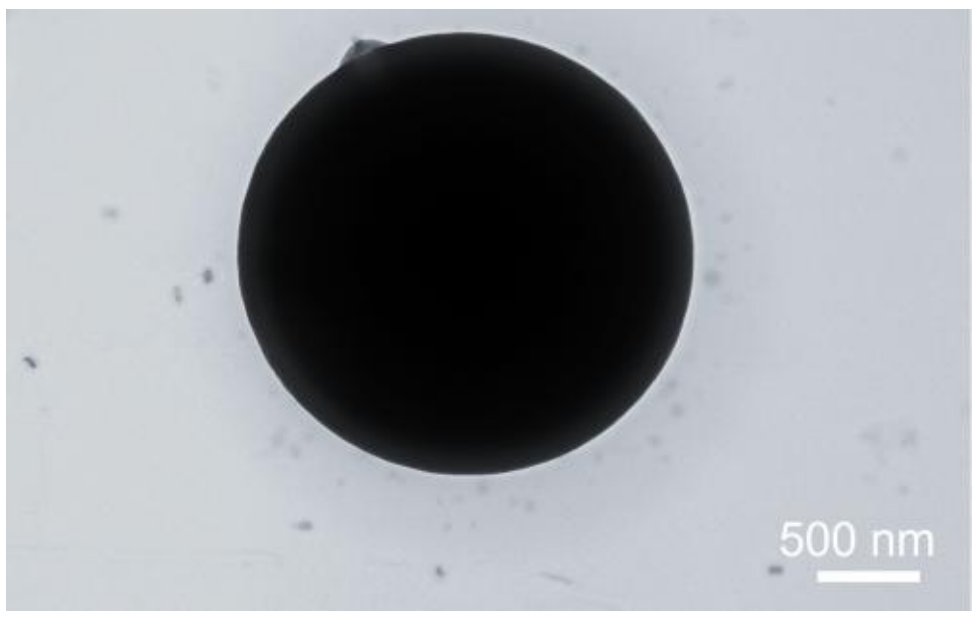

Fig. R2. TEM images of products without adding LW.

Second, to examine the interfacial role of liquefied wood, **we added coarse-grained simulations, including an actual-ratio TOP-rich/water interfacial model and an LWOH-removal control**

simulation. It should be noted that the simulation did not aim to reproduce the full chemical structure of LWMF resin. LWOH was used as a coarse-grained surrogate of the liquefied-wood-derived phenolic/polyhydroxy fragments, allowing us to probe the intrinsic interfacial affinity of liquefied-wood-derived phenolic/polyhydroxy moieties that are concurrently involved in LWMF formation, composite micelle assembly, and precursor-stage interfacial organization.

We respectfully note that direct molecular-level measurement of transient interactions among LWMF, TOP, F127, ethanol/water, and the evolving polymeric precursor under sealed hydrothermal conditions is technically challenging. The reaction involves simultaneous emulsion evolution, precursor condensation, interfacial adsorption, and framework solidification. Direct in situ probing of molecular contacts at the TOP-rich/water interface would likely disturb the emulsion state, solvent composition, interfacial organization, and polymerization process. Therefore, we used actual-ratio coarse-grained simulations as a complementary, non-perturbative approach to examine precursor-stage interfacial organization under controlled composition.

After removing LWOH from the equilibrated actual-ratio TOP120 interfacial configuration, the TOP-rich slab remained stable, and the F127/TOP contacts were largely retained. The normalized PPO–TOP contact density was 9.173 contacts per bead, comparable to that in the LWOH-containing TOP120 model, 8.484 contacts per bead, while the normalized PEO–TOP contact density decreased slightly from 0.974 to 0.803 contacts per bead. These results indicate that the intrinsic hydrophobic association between TOP and the F127 hydrophobic domain can be maintained without LWOH, whereas **LWOH is mainly responsible for incorporating the carbonizable precursor into the interfacial soft-template assembly and modulating the local interfacial organization.**

The interfacial affinity of the LW-derived phenolic/polyhydroxy component is critical for enabling the carbonizable LWMF precursor to participate in the interfacial soft-template assembly. In the LWOH-containing TOP120 model, LWOH simultaneously interacted with the TOP-rich and water-rich environments, with normalized LWOH–TOP and LWOH–water contact densities of 3.477 and 3.314 contacts per bead, respectively (Supplementary Table 5). **This indicates that LWOH is primarily located in the TOP-rich/water interfacial transition region, rather than being fully partitioned into either phase. Therefore, LW-derived phenolic/polyhydroxy fragments can participate in the interfacial region while remaining connected to the polar precursor environment.**

Importantly, removing LWOH affects the mode of interfacial organization. The normalized PPO–water contact density increased from 0.081 contacts per bead in the LWOH-containing model to 0.805 contacts per bead after LWOH removal, while the PEO–water contact density increased from 3.309 to 4.241 contacts per bead. These changes suggest that LW-derived species help stabilize the local interfacial environment and reduce the re-exposure of the template-rich domain to water. In addition, the orientation descriptor changed markedly after LWOH removal. The F127 orientation parameter shifted from −0.441 in the LWOH-containing model, close to the value expected for in-plane interfacial alignment, to 0.918 after LWOH removal, indicating a strong tendency toward alignment along the normal interface. These results show that LW influences not only precursor incorporation, but also the mode of interfacial organization of the soft-template assembly. TOP–PPO interaction provides the primary hydrophobic anchoring force, while the **LW-derived component acts as a carbonizable amphiphilic interfacial precursor that bridges the TOP/PPO-rich domain and the PEO/EtOH/water-rich polar environment, stabilizes the local interfacial environment and promotes in-plane interfacial organization.** The corresponding analysis has been added in Supplementary Note 1.7 and Supplementary Fig. 54.

Third, regarding your question on the self-assembly kinetics influence, we add LW-amount and hydrothermal-time dependent experiments to evaluate how liquefied wood affects the apparent polymerization process. The optical photographs show that increasing the LW amount accelerates the appearance of precipitation within the same hydrothermal time, indicating faster apparent polymerization of LWMF precursor at higher LW concentration (Fig. R3). We noted that the experiment is not intended to extract elementary rate constant for individual steps, because the present system involves simultaneous emulsion formation, precursor-template self-assembly, interfacial organization and LWMF polymerization. The time-dependent optical observation provides kinetic evidence under identical conditions. This kinetic effect is important for mesostructure formation: insufficient LW slows framework development and weakens the preservation of micelle-derived structures, whereas excessive LW accelerates the polymerization which mismatches the self-assembly of composite micelles. Therefore, **LW affects the kinetic matching between precursor condensation and soft-template assembly.**

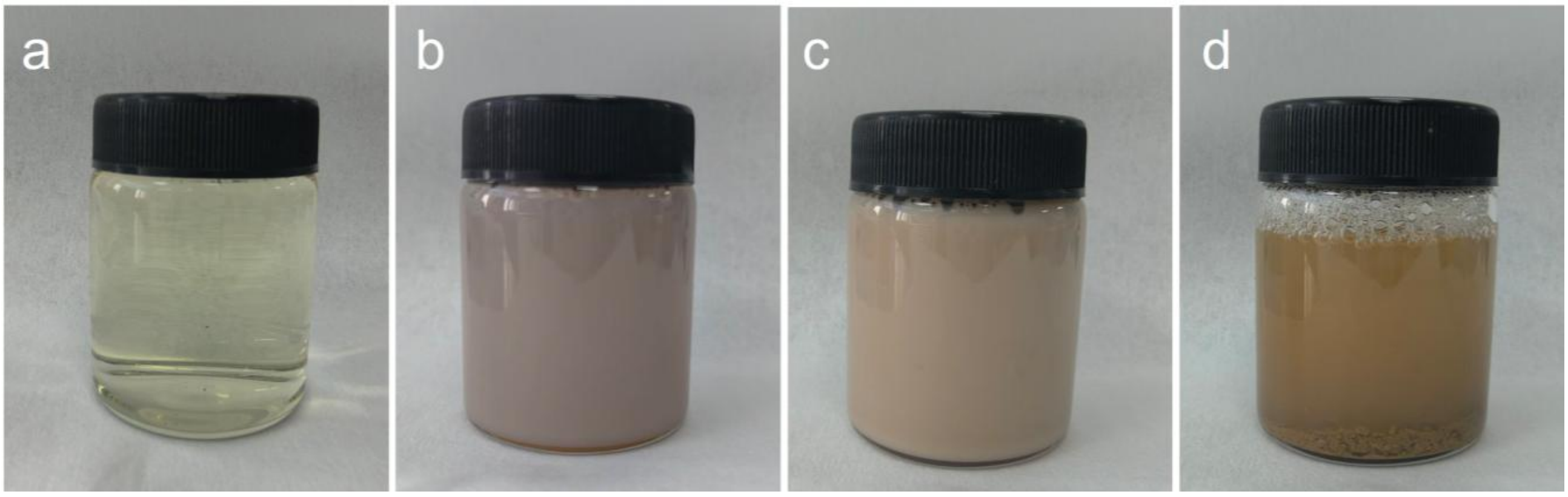

Fig. R3. Optical photographs of the solution with adding different amounts of LW after hydrothermal reaction for 30 min. (a) 0 g, (b) 0.1 g, (c) 0.3 g, (d) 0.5 g.

Finally, we want to clarify that the exact botanical origin of larch wood is not uniquely essential, but the liquefied biomass chemistry is essential for this synthesis platform. The critical requirement is a liquefaction-derived polyhydroxyl-containing phenolic/aromatic component that can be converted into polymerizable LWMF resin species and thereby provide framework-forming carbon chemistry, precursor–template interactions, interfacial participation, and polymerization-based fixation. In this sense the selection of biomass feedstock rich in lignin and cellulose is also one of our important innovations. **To verify the universality of the proposed method, we performed the synthesis using coconut shell and corn straw as alternative biomass resources** (Added Supplementary Fig. 40). These raw biomasses were converted into liquefied biomass precursors using a similar liquefaction strategy. Under representative synthetic conditions, regular morphologies and mesoporous structures were obtained. Moreover, varying TOP and ethanol in these liquefied-biomass systems produced structural evolution trends similar to those observed for liquefied wood. These results indicate that the functional role arises from the chemistry of liquefied biomass precursors.

Added text in the supplementary information:

1.7 Functional role of LWOH in TOP-rich/water interfacial organization

To clarify the functional role of LW in precursor-stage interfacial organization, an LWOH-removal control simulation was performed by removing LWOH from the equilibrated actual-ratio TOP120 long-box interfacial configuration while keeping F127M, TOP, ETHX and water unchanged (Supplementary Fig. 54).

After LWOH removal, the TOP-rich slab remained stable, with one TOP cluster and a largest TOP-cluster fraction of 1.0. The F127/TOP contacts were also retained. The normalized PPO–TOP contact density was 9.173 contacts per bead after LWOH removal, comparable to that in the LWOH-containing TOP120 model, 8.484 contacts per bead. The normalized PEO–TOP contact density was 0.803

contacts per bead after LWOH removal, slightly lower than that in the LWOH-containing model, 0.974 contacts per bead.

In the LWOH-containing TOP120 model, LWOH simultaneously contacted the TOP-rich and water-rich environments, with normalized LWOH–TOP and LWOH–water contact densities of 3.477 and 3.314 contacts per bead, respectively. This simultaneous association indicates that LW is primarily located in the TOP-rich/water interfacial transition region rather than being fully partitioned into either phase. Removing LWOH also increased the water exposure of F127. The normalized PPO–water contact density increased from 0.081 contacts per bead in the LWOH-containing model to 0.805 contacts per bead after LWOH removal, while the PEO–water contact density increased from 3.309 to 4.241 contacts per bead. These results suggest that LW contributes to stabilizing the local interfacial environment and suppressing the re-exposure of the PPO-rich domain to water, although TOP–PPO hydrophobic anchoring itself can still be maintained.

The shape and orientation descriptors further reveal that LW influences the mode of interfacial organization. The F127 axis1/axis3 ratio remained relatively high after LWOH removal, 3.717, compared with 4.076 in the LWOH-containing model, indicating that the assembly remained anisotropic. However, the orientation parameter changed markedly. In the LWOH-containing model, the F127 orientation parameter was -0.441, close to the value expected for in-plane interfacial alignment. After LWOH removal, this value shifted to 0.918, indicating a strong tendency toward alignment along the interface normal. This result shows that LW is important not only for precursor incorporation but also for maintaining the in-plane interfacial organization of the soft-template assembly.

Taken together, these results demonstrate that LW can serve as a carbonizable amphiphilic interfacial precursor that bridges the TOP/PPO-rich domain and the PEO/EtOH/water-rich polar environment, stabilizes the local interfacial environment and promotes the in-plane interfacial organization of F127.

Added Supplementary Fig. 40:

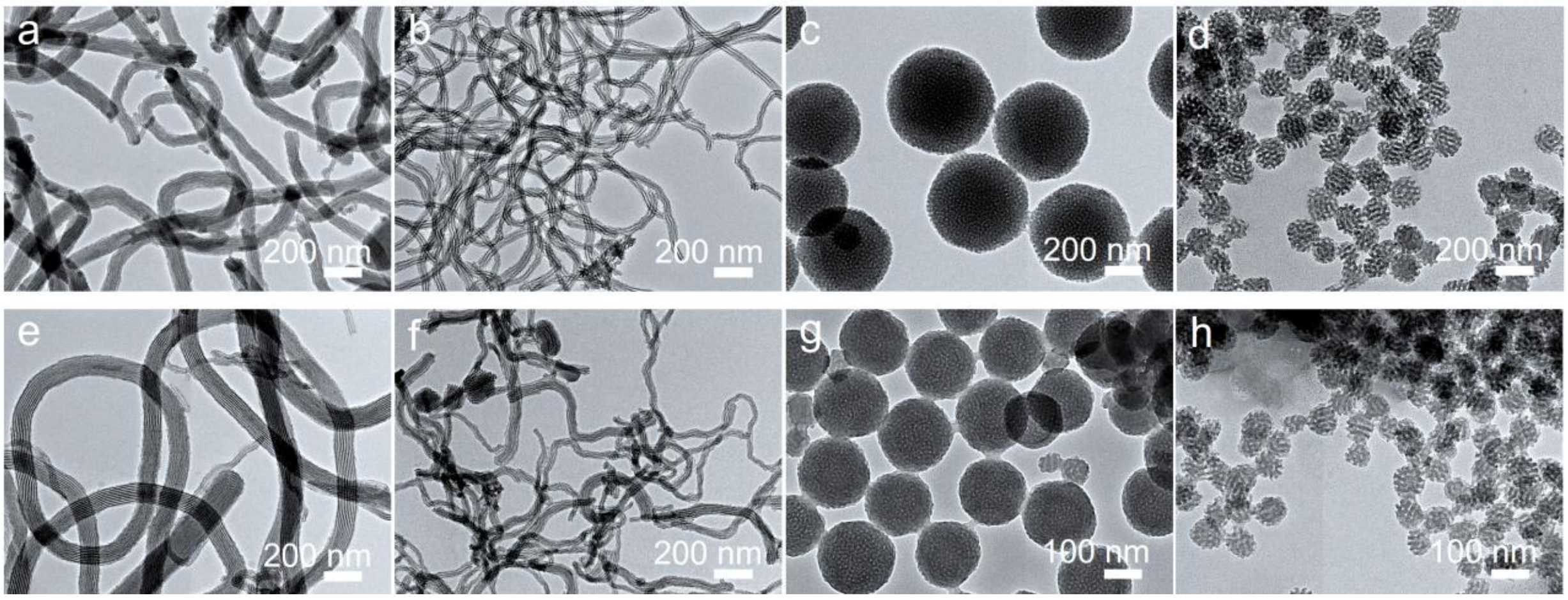

Supplementary Fig. 40. TEM images of MCNs synthesized from coconut shell (a–d) and corn straw (e–h). The synthesis condition was similar to the nanofibers and nanospheres. (a, e) 10 mL ethanol and 0.05 mL TOP. (b, f) 10 mL ethanol and 0.2 mL TOP. (c, g) 25 mL ethanol and 0.05 mL TOP. (d, h) 25 mL ethanol and 0.2 mL

TOP.

Added Supplementary Fig. 54:

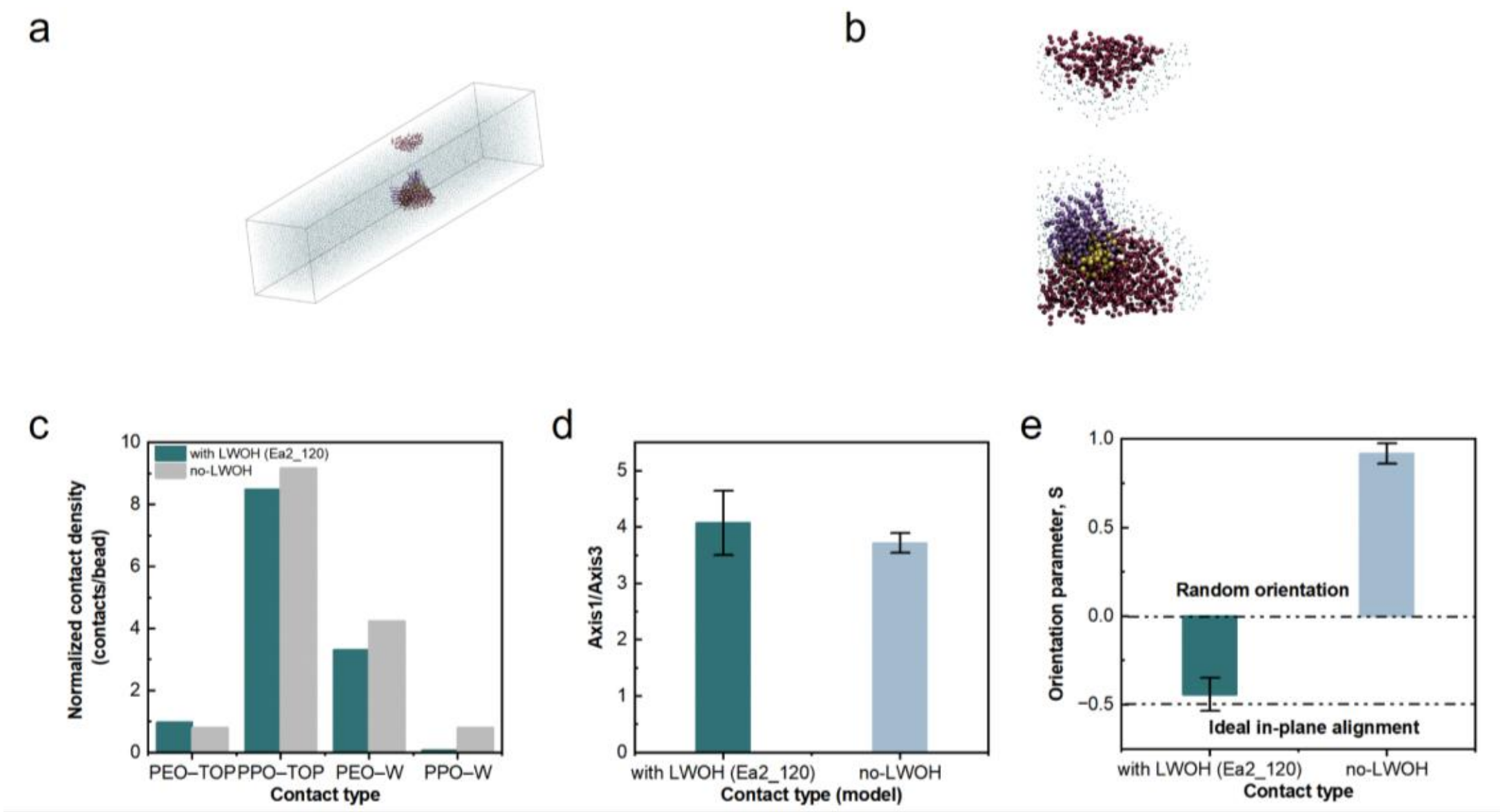

Supplementary Fig. 54. LWOH-removal control simulation clarifying the functional role of LW in interfacial self-assembly and organization. (a, b) Representative final configurations of the LWOH-containing TOP120 model and the LWOH-removal model, respectively. In the LWOH-removal model, LWOH was removed from the equilibrated TOP120 interfacial configuration and the trajectory was continued to examine the retained interfacial organization. Water and ETHX beads are omitted for clarity. (c) Normalized contact densities of PEO–TOP, PPO–TOP, PEO–water and PPO–water. LWOH removal retains F127–TOP contacts but increases the water exposure of F127, especially the PPO segment. (d) F127 axis1/axis3 ratio in the LWOH-containing and LWOH-removal models. (e) F127 orientation parameter S relative to the normal interface. S = -0.5 corresponds to ideal in-plane alignment, S = 0 to random orientation and S = 1 to perpendicular alignment. Error bars represent the standard deviation over the final 2 ns of the corresponding production trajectories.

Revised text ① (Page 2, line 22): Developing biomass-derived programmable self-assembly systems for precise control of carbon morphology and architecture remains challenging, primarily because of the intrinsic heterogeneity that hinders controllable assembly.

Revised text ② (Page 4, line 75): However, owing to the compositional complexity and rapid polymerization rate, it is difficult to coordinate interfacial participation, template interaction and polymerization within a controllable soft-template assembly process. The self-assembly involved occurs exclusively confined in a single aqueous phase environment through homogeneous self-assembly, and strategies for regulating structure remain limited to micelle structure and nucleation kinetics.

Revised text ③ (Page 32, line 616): In addition, LWOH-removal simulations indicate that liquefied-wood-derived species bridge hydrophobic TOP/PPO-rich domains and polar PEO/ethanol/water-rich environments, supporting the active role of liquefied wood in precursor–template interaction, interfacial organization and framework fixation (Supplementary Fig. 54). Together, these simulations support the mechanism proposed in Fig. 7, in which TOP and ethanol regulate micelle curvature, TOP/water interfaces define favorable assembly loci and liquefied wood couples interfacial assembly with carbon-framework formation.

Revised text ④ (Page 23, line 431): The generality of this emulsion-mediated synthetic approach was also examined, in which coconut shell and corn straw were selected as the raw biomass resources and converted into liquefied biomass precursors (Supplementary Fig. 40). Despite the different origins and compositions from wood, regular morphology and mesoporous structures were obtained. Moreover, varying TOP and ethanol in these biomass-derived systems produced structural evolution trends similar to those observed for liquefied wood, supporting the generality of their regulatory roles in emulsion-mediated self-assembly.

Once again, we sincerely appreciate your thoughtful and constructive comments. They have been very helpful in guiding us to better highlight the novelty of this work and to strengthen the mechanistic basis of the revised manuscript.

Reviewer #2:

General comments: In this manuscript, the authors developed a synthesis platform for preparing mesoporous carbon materials with versatile morphologies. The underlying mechanism involves surfactant self-assembly and surface tension at the oil/water interface. Admittedly, the microscopy images were high quality showing ordered structures at both nanoscale as well as particle/fiber scale. The concept/work itself might be interesting, but there are several concerns regarding this work:.

General response: Thanks for your positive comments and careful evaluation of our manuscript. We are also grateful for the constructive and insightful comments which help us substantially improve the clarity and rigor of the manuscript. In the revised manuscript, we have added and clarified time-dependent observations of the polymerization process, including intermediate-stage photographs and

TEM images, and revised the mechanism as a coupled thermodynamic-kinetic process. We have also clarified the functional relevance of the different morphologies by discussing the relationship between carbon nanoarchitecture and mixed ion-electron thermoelectric behavior. Moreover, we have added coarse-grained simulations to provide complementary support for precursor-stage organization. In addition, we re-examined the pore-size analysis and revised the related discussion to distinguish the primary mesopores observed by TEM from larger voids or interparticle spaces contributing to high-relative-pressure adsorption. We have also refined the discussion on the temperature-dependent structural evolution by explicitly considering polymerization kinetics and kinetic matching during micelle assembly. Detailed responses to each comment are provided below.

Comment 1: Most characterization are only focused on the final carbon materials. The nanostructure evolution occurs in both solution assembly phase and polymerization process. However, there is no mesoscale characterization on these intermediate products, which limits the scope and impact of this study. Because of this, the discussions and mechanistic explanation are not well supported. For example, will polymerization induce further phase separation or phase change? At least SAXS and microscopy images should be provided at different process stages.

Response: We sincerely thank you for this important comment. We agree that the nanostructure evolution in the present emulsion-mediated system should not be inferred only from the final carbonized products. The formation of these architectures involves several coupled stages, including precursor/micelle organization in solution, emulsion-interface-assisted assembly, LWMF polymerization, structural fixation, carbonization and template removal. In the revised manuscript and Supplementary Information, we have reorganized the mechanistic discussion and added intermediate-stage observations to clarify when and how the mesoscale structures are formed.

We have added time-dependent TEM observations of polymeric precursors collected at different hydrothermal stages for the hollow carbon nanofiber-forming system. As shown in Supplementary Fig. 2a–d, the early products obtained at 30 and 60 min are mainly aggregated or partially elongated intermediates. After further hydrothermal treatment, fibrous frameworks with hollow/mesoporous features become evident. With prolonged treatment, well-defined hollow fibrous networks are formed, and further prolonging the reaction does not lead to obvious morphological transformation or

coarsening. These observations indicate that the hollow fibrous architecture is progressively established during the hydrothermal assembly/polymerization process and then fixed by precursor condensation, rather than being generated only during the subsequent carbonization process. Then, we have added time-dependent SAXS measurements of products obtained at different hydrothermal durations. As shown in Supplementary Fig. 2e, the early-stage products show relatively weak and broad scattering features, consistent with incompletely developed micelle-derived polymer aggregates. With increasing hydrothermal time, the mesoscale scattering feature becomes more discernible, supporting the progressive development of micelle-derived mesostructured polymer frameworks. Importantly, no new sharp higher-order peaks or additional scattering peak series appear upon prolonged hydrothermal treatment. This result suggests that extended polymerization does not induce an independent late-stage ordered mesophase transition. Instead, the SAXS evolution supports that the micelle-derived mesoscale organization is gradually established and subsequently fixed during hydrothermal polymerization.

We have further revised the discussion of characterization of the as-made resin polymer products before carbonization. The SEM and TEM images of the polymeric intermediates show self-supporting fibrous architectures with pronounced surface striations and hollow/mesoporous features (Supplementary Fig. 3). These structural features are consistent with those observed in the final carbon nanofibers after carbonization, although the framework undergoes shrinkage during thermal treatment. This comparison demonstrates that the key mesoscale morphology and internal hollow/mesoporous architecture already exist at the polymer precursor stage. Therefore, carbonization and template removal mainly convert the polymeric framework into carbon and generate open porosity, rather than creating the fibrous/hollow architecture from a featureless precursor.

Regarding whether polymerization induces further phase separation or phase change, we have now clarified this point in the revised manuscript. **Polymerization plays an active and indispensable role in the structure-evolution process; it should not be regarded as an independent late-stage macrophase separation or a separate equilibrium phase transition occurring after a preformed micellar structure has already been established.** Before hydrothermal treatment, the precursor system was already a multicomponent emulsion containing TOP droplets, F127/TOP/LWMF composite micelles, ethanol/water solvent and reactive LWMF oligomeric species. During hydrothermal treatment, micelle organization, interfacial localization and LWMF condensation occur

cooperatively. The polymerization of LWMF gradually increases the molecular weight and crosslinking degree of the precursor species, decreases their mobility and solubility, and strengthens the spatial coupling between LWMF-rich framework domains and F127/TOP-rich template domains. This process converts dynamic micelle/oligomer aggregates into a solid polymer framework and arrests the evolving mesostructure. In this sense, polymerization can promote local nanoscale microphase organization between polymer-rich framework regions and template-rich domains, which is intrinsic to soft-template-directed mesopore formation. However, this process is different from an uncontrolled macrophase separation or a late-stage phase change that independently generates the final morphology. The time-dependent TEM and SAXS results show that the hollow/mesoporous fibrous framework is gradually established during the coupled assembly/polymerization stage and then structurally fixed. The absence of newly emerged SAXS peak series upon prolonged hydrothermal treatment further indicates that no additional independent mesophase transition occurs during extended polymerization. Based on these results, we have revised the mechanistic description to a cooperative thermodynamic-kinetic assembly process. Effective interfacial affinity and micelle curvature/packing tendency guide the preferred organization pathway, while LWMF polymerization progressively fixes the evolving mesostructure.

This interpretation is also consistent with kinetic-control experiments. Changing the hydrothermal temperature or HCl concentration significantly affects the final morphology and mesostructural definition, because both parameters regulate the polymerization rate of LWMF. If polymerization is too slow, the micelle/emulsion-derived assemblies cannot be fixed efficiently and may evolve into poorly defined or aggregated structures. If polymerization is too fast, the system can be prematurely arrested before cooperative micelle organization and interfacial assembly are sufficiently developed, also leading to disrupted or irregular structures.

In addition to these experimental observations, we have added coarse-grained simulations to provide complementary support for precursor-stage organization. The formulation-dependent simulations show that increasing TOP content enhances hydrophobic contributions and shifts the local organization toward higher packing tendency and greater aggregate anisotropy, whereas higher ethanol content increases polar contact contributions and favors lower local packing tendency. Interfacial simulations further show that F127/TOP/LWOH aggregates can organize near the TOP-rich/water interfacial region, where TOP/PPO-rich domains provide hydrophobic anchoring and PEO/LWOH

hydroxyl-rich regions maintain polar contacts with the solvent. These simulations do not replace direct in situ observation of hydrothermal polymerization, but they provide complementary molecular-level support for the experimentally observed formulation-dependent precursor organization.

Importantly, we find that this comment is closely related to the concerns raised in Reviewer #4 Comment 3. Accordingly, we have revised the manuscript to describe the structure evolution as a cooperative thermodynamic-kinetic process rather than a simple equilibrium micelle phase transition. Effective interfacial affinity and micelle curvature/packing tendency guide the preferred assembly pathway, whereas polymerization kinetics fix or disrupt the evolving mesostructure depending on the kinetic matching condition.

The added detailed coarse-grained simulations can be seen in the revised supplementary Information (Page 68–77).

Revised text ① (Page 6, line 122): Time-dependent TEM observations were further performed to track the formation of hollow nanofibers (Supplementary Fig. 2). Early products obtained at 30 min and 60 min show aggregated or partially elongated intermediates, whereas fibrous frameworks with hollow/mesoporous features become evident after 180 min. Prolonging the hydrothermal treatment to 10 h yields well-defined hollow nanofibers, while further extension to 24 h does not induce obvious morphological transformation or coarsening. The corresponding time-dependent SAXS patterns show the gradual development of a broad mesoscale scattering feature during hydrothermal treatment. The broad feature around $q \approx 0.7\ nm^{-1}$ corresponds to a characteristic length of approximately 9 nm. No new sharp higher-order peaks or additional peak series appear upon prolonged hydrothermal treatment, indicating that extended polymerization does not induce an independent late-stage ordered mesophase transition. These observations suggest that the hollow fibrous architecture is established through polymerization-coupled micelle/emulsion assembly and subsequently fixed by precursor condensation, rather than being generated by a separate phase transition or by carbonization alone.

Revised text ② (Page 8, 143): Scanning electron microscopy (SEM) images reveal that the as-prepared mesostructured resin polymers exhibit a uniform fibrous morphology, with a representative cross-sectional diameter of 40 nm and striped grooves along the nanofiber surface, which are consistent with the radial growth of composite TOP/F127/LWMF micelles during interfacial self-assembly (Supplementary Fig. 3a,c). Transmission electron microscopy (TEM) images show that the polymeric nanofibers possess well-defined hollow mesochannels of ~12 nm (Supplementary Fig. 3b,d). After

carbonization in N_2 atmosphere, fibrous morphology is retained while shrinking to a representative outer diameter of 30 nm (Fig. 1b). The hollow interior and porous wall are preserved after carbonization, with a wall thickness of ~8 nm as observed from the magnified TEM image (Fig. 1c,d).

Revised text ③ (Page 33, line 638): Therefore, the observed morphology evolution should be interpreted as a cooperative thermodynamic-kinetic process, in which effective interfacial-affinity-biased locus selection and micelle-curvature regulation guide the assembly pathway, while polymerization kinetics fixes or disrupts the evolving mesostructure depending on the kinetic matching condition.

Added Supplementary Fig. 2

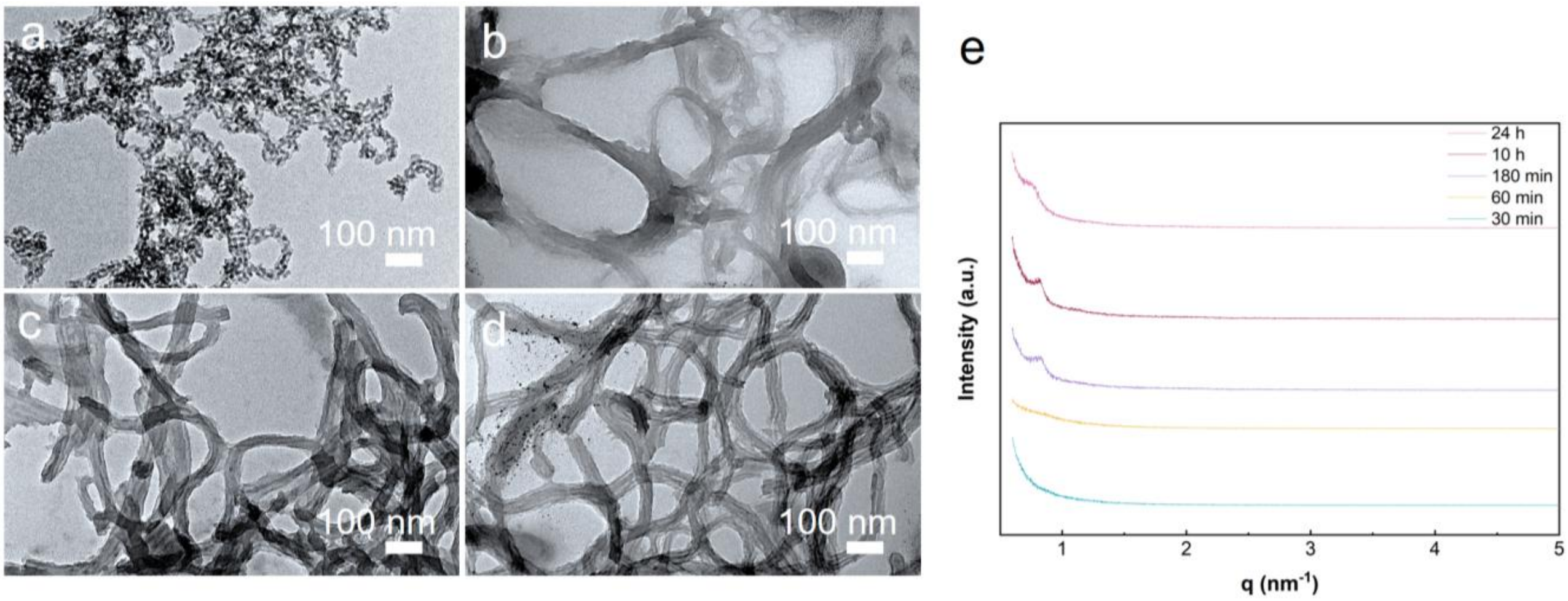

Supplementary Fig. 2. TEM image of polymeric precursor of MCNs-3 at different hydrothermal times. (a) 30 min, (b) 60 min, (c) 180 min, (d) 24 h, (e) SAXS patterns of the corresponding products collected after 30 min, 60 min, 180 min, 10 h and 24 h of hydrothermal treatment. The absence of newly emerged sharp higher-order peaks upon prolonged hydrothermal treatment indicates that extended polymerization does not induce an additional independent ordered mesophase transition.

Revised Supplementary Fig. 3:

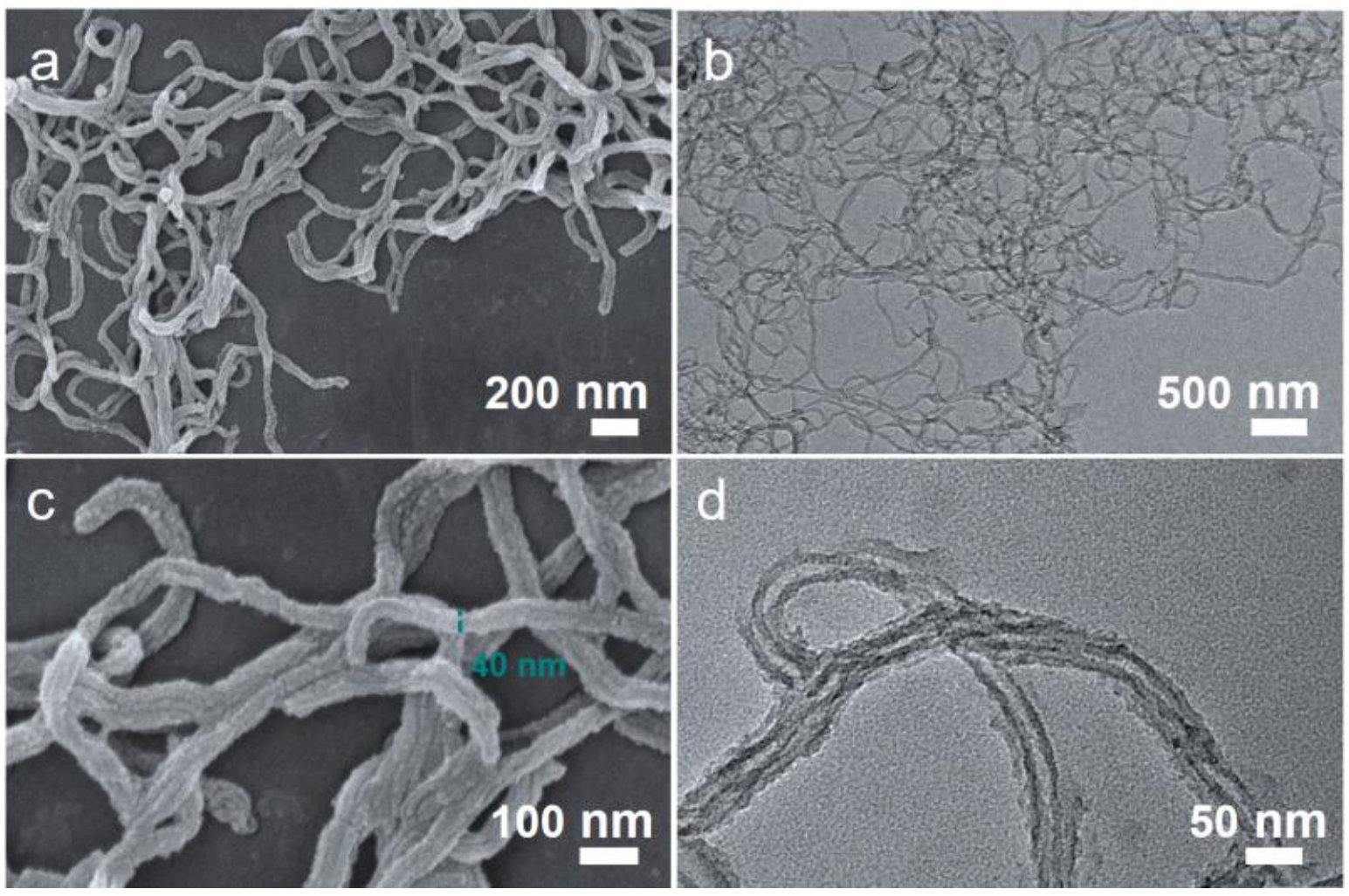

Supplementary Fig. 3. Structural characterization of the as-made resin polymers. (a, c) SEM images and (b, d) TEM images of the as-made resin polymers prepared by the emulsion-mediated self-assembly approach. The as-made resin polymers were prepared by using F127 as the soft-template, TOP as the swelling agent and interface mediation agent, and LW and melamine as the carbon source and nitrogen source.

Comment 2: It was not clear that these self-assembled structures are at thermodynamic equilibrium state or kinetically arrested. This is important to understand especially towards more rational control in the phase morphology when scaling up.

Response: Thanks for your insightful comment. We agree that distinguishing thermodynamic equilibrium structures from kinetically arrested architectures is essential for understanding and controlling this emulsion-mediated self-assembly process, particularly when considering scale-up.

In the present reaction system, we do not consider the final polymer architecture to be thermodynamic equilibrium. Thermodynamic equilibrium state implies that the micelles and polymeric precursors can continuously aggregate toward the lowest-energy morphology under the given conditions. However, in our synthesis, the self-assembly of TOP/F127/LWMF micelles occurs simultaneously with the liquefied wood-melamine-formaldehyde precursor. Therefore, although interfacial affinity and micelle curvature guide the early assembly pathway and structure, the assembled intermediate structures are progressively fixed by the precursor growth and polymerization. The final assembled structures are thus more appropriately described as polymerization-fixed,

kinetically arrested architectures formed within a thermodynamic-kinetic matching window.

We have performed additional time-dependent hydrothermal experiments for the hollow nanofiber-forming system and added the corresponding Supplementary Fig. 2. At the early stages, the products evolve from small aggregated/elongated intermediates to fibrous frameworks, indicating the progressive assembly and polymerization of TOP/F127/LWMF composite micelles. With increasing hydrothermal time, the hollow fibrous architecture and mesoporous shell become increasingly defined. After 10 h, well-defined hollow carbon nanofibers with clear mesoporous shells are already formed. Extending the hydrothermal treatment to 24 h does not lead to further morphological transformation or obvious coarsening. These results indicate that the hollow fibrous architecture is essentially established by approximately 10 h and then kinetically fixed by precursor condensation, rather than continuously evolving toward a thermodynamic equilibrium morphology. In addition, hydrothermal temperature and HCl concentration strongly influence the final morphology and mesostructure, further supporting the importance of kinetic matching between micelle assembly and precursor polymerization. The revised text is as follows:

Revised text (Page 6, line 122): Time-dependent TEM observations were further performed to track the formation of hollow nanofibers (Supplementary Fig. 2). Early products obtained at 30 min and 60 min show aggregated or partially elongated intermediates, whereas fibrous frameworks with hollow/mesoporous features become evident after 180 min. Prolonging the hydrothermal treatment to 10 h yields well-defined hollow nanofibers, while further extension to 24 h does not induce obvious morphological transformation or coarsening. The corresponding time-dependent SAXS patterns show the gradual development of a broad mesoscale scattering feature during hydrothermal treatment. The broad feature around $q \approx 0.7\ nm^{-1}$ corresponds to a characteristic length of approximately 9 nm. No new sharp higher-order peaks or additional peak series appear upon prolonged hydrothermal treatment, indicating that extended polymerization does not induce an independent late-stage ordered mesophase transition. These observations suggest that the hollow fibrous architecture is established through polymerization-coupled micelle/emulsion assembly and subsequently fixed by precursor condensation, rather than being generated by a separate phase transition or by carbonization alone.

Added Supplementary Fig. 2:

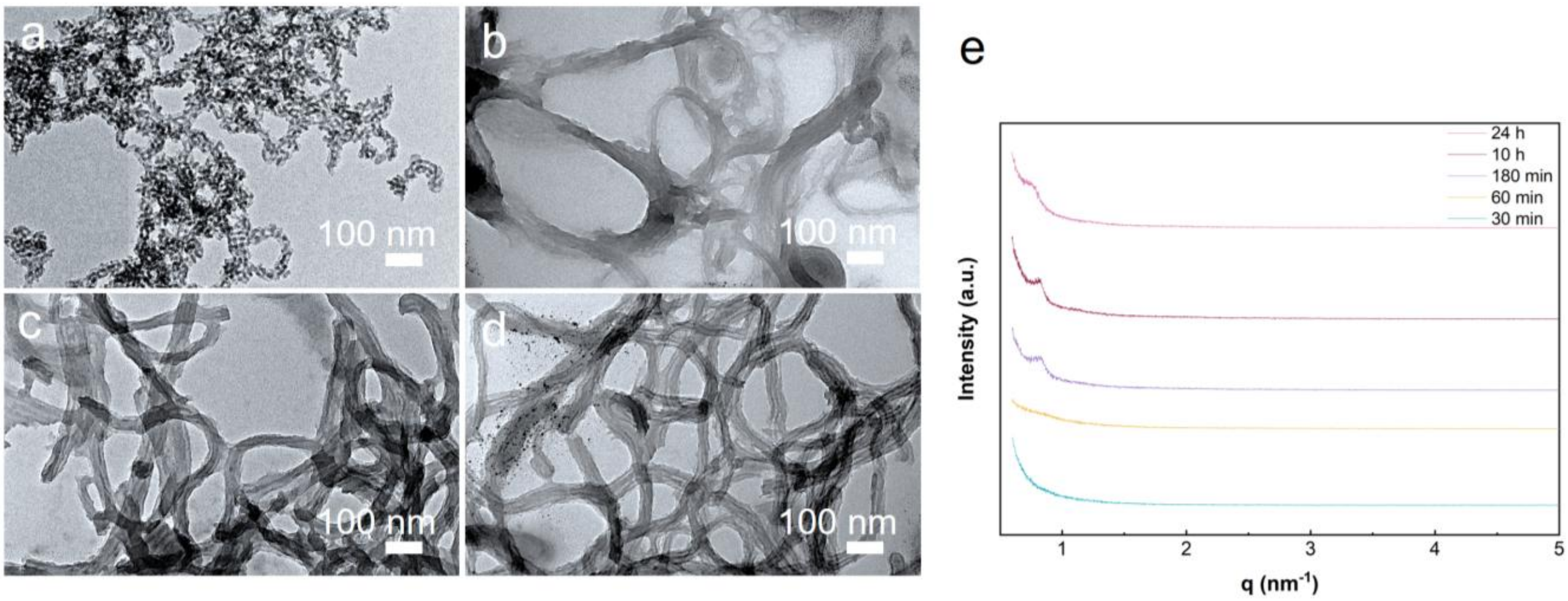

Supplementary Fig. 2. TEM image of polymeric precursor of MCNs-3 at different hydrothermal times. (a) 30 min, (b) 60 min, (c) 180 min, (d) 24 h, (e) SAXS patterns of the corresponding products collected after 30 min, 60 min, 180 min, 10 h and 24 h of hydrothermal treatment. The absence of newly emerged sharp higher-order peaks upon prolonged hydrothermal treatment indicates that extended polymerization does not induce an additional independent ordered mesophase transition.

Comment 3: What's the key benefits to attain these different morphologies? In many cases, the pore size are very comparable. Are the changes to their particle size/shape critical to influence their performance in intended applications?

Response: We sincerely thank you for the important question. We agree that the significance of attaining these different morphologies and structures should be clearly connected to both the synthetic strategy and the intended application rather than being presented only as structural diversity.

First, in many reported syntheses, different carbon morphologies often require different precursor systems, templates or synthetic routes, which makes systematic comparison difficult. In our work, diverse mesoporous carbon nanoarchitectures are obtained within one biomass liquefied wood-derived emulsion-mediated self-assembly system by adjusting key formulation parameters. **Therefore, the different morphologies are not presented only as isolated structures, but as evidence that the assembly pathway can be regulated within a single synthetic platform.**

Second, **for mixed ion-electron thermoelectric ionogels, the particle/fiber shape, dimensionality and internal architecture directly influence the formation of electronic pathways, ion redistribution, interfacial polarization and charge compensation under external loading** (*Adv. Energy Mater.* 2023, 202203692; *Nat. Mater.* 2019, 608). In mixed ionic–electronic thermoelectric

systems, electronically connected fillers are required to provide continuous charge-transport pathways, while the ionogel phase contributes ionic thermodiffusion and interfacial ion accumulation (*J. Mater. Chem. A.* 2021, 13588; *Energy Environ. Sci.* 2025, 1294). One-dimensional nanofibers can more readily interconnect within the ionogel matrix to form continuous electronic pathways. Such fibrous connectivity lowers the requirement for particle-to-particle contacts and enables long-range electron transport under an external load. Moreover, recent ionogel thermoelectric fibers further highlight the importance of aligned internal architectures for efficient thermoelectric response (*Nat. Commun.* 2017, 1215; *Nat. Commun.* 2025, 544). Ordered mesoporous carbon nanofibers provide an electronically connected backbone, while their aligned channels also facilitate ion transport along the fibrous framework. Hollow carbon nanofibers retain this one-dimensional electronic connectivity and additionally introduce a hollow interior and thin mesoporous shell. This architecture exposes both the outer and inner carbon surfaces to the ionogel, shortens radial ion-transport pathways, facilitates ion accumulation/redistribution under a temperature gradient, and allows electronic charge compensation during ionic thermodiffusion (*Chem. Eng. J.* 2025, 168809). By contrast, nanosphere-based architecture mainly contributes through local ionogel-carbon interfacial contact and internal compartments. These features can promote local ionic polarization and ion accumulation. However, their zero-dimensional morphology makes long-range electronic transport dependent on discrete interparticle contacts. Electron transport between separated nanospheres is therefore more likely governed by contact-limited tunneling or hopping pathways, which are less effective than the percolated fibrous network for sustained charge compensation under external loading (*Adv. Energy Mater.* 2025, 2404522). This explains why MCNs-10 and MCNs-17 can still generate ionic thermovoltage but deliver much lower steady output than hollow carbon nanofiber-based ionogels.

We also recognize that this comment is closely connected to the structure-property concern raised by Reviewer #4 (comments 4 and 5). We have added **Supplementary Table 6** to correlate these transport differences with dimensionality, internal architecture, internal resistance, and output performance. We also strengthened the discussion on the morphology/structure influence on intended thermoelectric application.

Revised text ① (Page 27, line 527): However, both systems remain predominantly ion-conducting, and the lack of a continuous electronic percolation network severely limits effective charge compensation under external loading. In this context, the reduced particle size of MCNs-17 may

facilitate faster ion redistribution under a temperature gradient, yet this advantage is insufficient to overcome the intrinsic limitation imposed by discontinuous electronic pathways.

Revised text ② (Page 33, line 642): The mixed ion-electron thermoelectric behavior of ionogels is commonly governed by the balance between electronic percolation and ionic transport within the composite network[57]. As summarized in Supplementary Table 6, the superior performance of MCNs-3 cannot be attributed solely to the surface area or pore volume, but rather to the coupling of one-dimensional electronic percolation, hollow/mesoporous ion-accessible interfaces, reduced ion-diffusion pathways and lower device-level internal resistance. One-dimensional ordered mesoporous carbon nanofibers provide continuous carbon backbones that facilitate long-range electronic connectivity, while their ordered mesoporous channels offer accessible ion-transport pathways along the fibrous framework. Hollow carbon nanofibers retain this one-dimensional electronic connectivity and introduce additional internal cavities and thin mesoporous shells, enabling ion access to both outer and inner carbon surfaces and shortening radial ion-diffusion pathways. This architecture is therefore more effective in coupling ionic polarization with electronic charge compensation under a temperature gradient[58,59]. By contrast, zero-dimensional nanosphere-based architectures, including golf ball-like and multichambered nanospheres, provide abundant local ion-accessible surfaces, internal compartments, and rough external interfaces, which are beneficial for ion accumulation and interfacial polarization[60]. However, their zero-dimensional particle morphology makes long-range electron transport more dependent on discrete interparticle contacts. Such discontinuous electronic pathways are less favorable for forming a percolated carbon network in the ionogel matrix, thereby limiting electronic charge compensation and steady power delivery under external loading[61]. These considerations explain why the hollow carbon nanofiber-based ionogels exhibit the most balanced mixed ion-electron transport among the representative architectures examined.

Supplementary Table 6. Summary of carbon nanoarchitecture and mixed ion-electron thermoelectric performance.

Samples	MCNs-1	MCNs-3	MCNs-10	MCNs-17
Key structural features	1D fibrous framework with ordered parallel mesochannels	1D hollow fibers with mesoporous shell and internal cavity	nanospheres with local cavities/rough surfaces	nanospheres with multichambered interiors
BET surface area/ (m^2	1235	639	583	671

g^{-1})				
Pore volume/ (cm^3 g^{-1})	0.62	0.90	0.37	0.47
Ionic conductivity/ (S m^{-1})	0.248	0.361	1.44	1.10
Electronic Conductivity/ (S m^{-1})	7.00×10^{-7}	4.91×10^{-7}	6.02×10^{-15}	6.02×10^{-13}
Internal resistance/ (kΩ)	14.01	12.52	2.50	3.29
Thermopower/ (mV K^{-1})	25.6	26.8	23.8	24.4
Maximum P_{av}/ (W m^{-3})	63.01	68.32	10.67	11.38
Structure-property relationship	1D ordered framework enables efficient electronic percolation, while ordered mesochannels provide directional ion transport.	Hollow 1D architecture integrates continuous electronic pathways with enhanced ion-accessible interfaces, enabling balanced ion-electron transport.	High ion-accessible surface promotes ionic polarization, but discontinuous particle contacts limit electronic transport.	Reduced size and multichambered structure facilitate ion diffusion, yet insufficient electronic percolation restricts charge compensation.

Comment 4: In Figure 3, the corresponding pore size distribution seems to be different from what you would expect from the sorption isotherms. A sharp increase in p/p0=0.9 range should correspond to pore size at least larger than 10 nm, according to the BJH model as authors claimed to use. This part needs further examination.

Response: We sincerely thank you for this important comment. We fully agree with you that the increase in the N_2 sorption isotherms near p/p_0 corresponds to relatively large mesopores. Your careful examination helped us recognize that our original BJH-based pore-size analysis was not sufficiently appropriate for describing the hierarchical pore structures of these MCNs, and could lead to a misleading interpretation of the high-relative-pressure uptake. Following your suggestions, we have retested the required samples and carefully examined the N_2 sorption data using a NLDFT pore-size distribution model to further evaluate the hierarchical pore structures of MCNs.

During the preparation of mesoporous carbon materials using the soft-template method, the

TOP/hydrophobic blocks form the mesoporous pores, and the hydrophilic blocks form micropores after carbonization of the LWMF resin, suggesting that our materials inherently possess a hierarchical pore structure. In addition, the hollow interiors, interparticle voids and structural cavities give rise to larger mesopores, which are responsible for the pronounced uptake at high relative pressure. Therefore, the pore structure of the MCNs should not be interpreted as a single uniform mesoporous system. Compared with the original BJH analysis, the NLDFT model provides a more appropriate description of this hierarchical pore system. In the revised Figure 3, the pore-size distributions have been replotted using the NLDFT model over a broader pore-size range. The revised results show that the smaller mesopores mainly originate from F127/TOP/LWMF composite micelle-templated mesochannels, whereas the sharp uptake near P/P_0~0.9 is associated with larger mesopores, hollow interiors, interparticle voids and/or textural porosity, which is more consistent with both the N_2 sorption isotherms and the TEM-observed hollow/mesoporous structures.

We are grateful to you for pointing out this issue, as this comment allowed us to improve the rigor and reliability of the pore-structure analysis. Accordingly, we have revised Figure 3, its caption, the pore-structure discussion in the manuscript, and the Methods section. We have also removed the previous BJH-based description to avoid over-assigning the high-pressure sorption uptake to the small primary mesopores.

Revised text ② (Page 11, line 205): The NLDFT-derived pore-size distributions reveal hierarchical porosity in these MCNs, including micropores, primary micelle-templated mesopores and broader larger-pore contributions, which might originate from hollow interiors, interparticle voids and structural cavities.

Revised Figure 3:

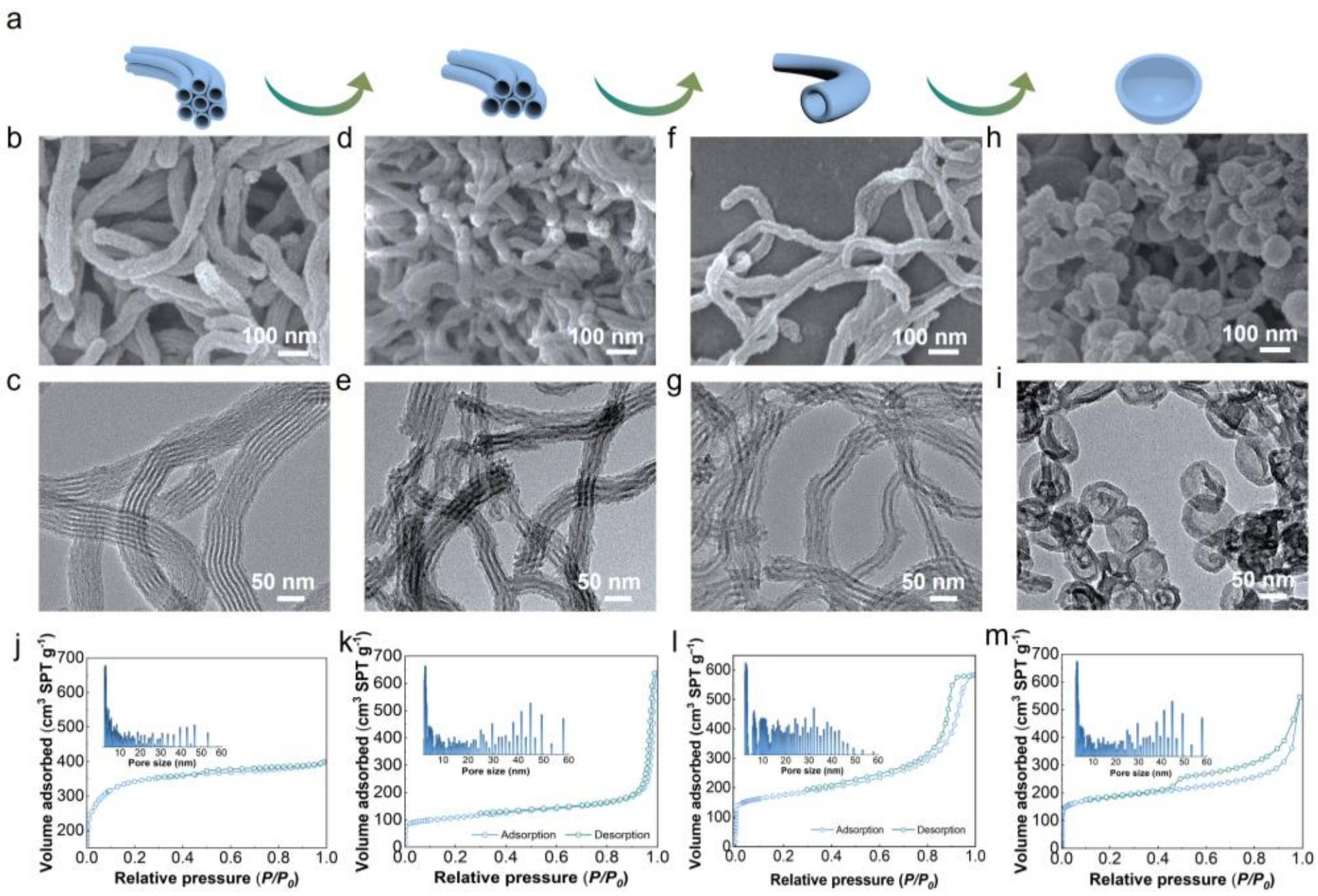

Fig. 3. Controllability of the emulsion-mediated micelle self-assembly strategy by tuning the TOP content. (a) Structural models depicting experimentally sampled TOP-dependent morphology/mesostructured evolution of mesoporous carbon nanomaterials from ordered mesochannel nanofibers to hollow nanofibers and ultimately to bowl-like nanospheres, (b–i) SEM and TEM images of the MCNs-x (x=1,2,3,4) prepared by adjusting the amount of TOP. (b, c) 0.05 mL, (d, e) 0.1 mL, (f, g) 0.2 mL, (h, i) 0.3 mL. (j–m) The corresponding N2 sorption isotherms and distribution histograms of pore sizes.

Comment 5: Why the structure development is sensitive to the reaction temperature? The authors tried to explain this in the manuscript, but it was still not clear. This suggests there are more factors (either thermodynamic or kinetics) involved in controlling final morphologies than the content of TOP, surfactant ct, ethanol, etc. What's the polymerization kinetics at these temperatures?

Response: We sincerely thank you for this insightful comment. We agree that the temperature-dependent structural evolution cannot be explained solely by the amounts of TOP, surfactant, or ethanol. In the revised manuscript, we have clarified that hydrothermal temperature acts as a coupled thermodynamic-kinetic parameter in this emulsion-mediated self-assembly system.

The reason why the structure development is sensitive to hydrothermal temperature is that the hydrothermal temperature can influence micelle hydration, effective hydrophobic-domain volume and preferred micelle curvature, thereby affecting the resulting mesostructure. Previous studies have demonstrated that increasing hydrothermal temperature decreases the hydration/hydrophilicity of the PEO segments and promotes the contraction of PEO chains toward the PPO-rich hydrophobic domain, which increases the effective hydrophobic volume and thereby reduces the micelle curvature

(*Aggregate* 2024, e570, *Small* 2025, 2502475). In the present emulsion-mediated system, a similar temperature-dependent micelle effect is expected, and it is further coupled with TOP swelling and interfacial assembly. **The increased temperature alters the preferred micelle curvature/packing tendency, mesopore geometry, and micelle-TOP interfacial affinity.** This explains why even under the same TOP, ethanol, surfactant, and HCl contents, changing hydrothermal temperature can still lead to different morphologies and mesostructures.

Second, **hydrothermal temperature also strongly affects the polymerization and nucleation kinetics of the LWMF precursor.** The formation of ordered mesoporous architectures requires kinetic matching between the self-assembly of TOP/F127/LWMF composite micelles and the polymerization/condensation of the LWMF precursor. If the temperature is too low, LWMF condensation and nucleation are too slow. Under this condition, the composite micelles cannot be effectively fixed during assembly, and the weak precursor-template interaction leads to aggregated or poorly defined mesostructures. When the temperature is appropriate, the precursor polymerization rate is matched with micelle self-assembly, allowing seed nuclei to form and to serve as sites for further micelle growth. If the temperature is too high, LWMF condensation and nucleation become too rapid, generating numerous small nuclei and prematurely fixing the structure before the composite micelles can reorganize into ordered mesophases. Excessively high temperature may also weaken hydrogen bonding between the LWMF and Pluronic segments, further disrupting cooperative assembly. As a result, irregular particles or defective mesostructures are obtained. This is supported by both our previous temperature-dependent study and our present work (*Adv. Funct. Mater.* 2023, 2213852, *Chem. Eng. Sci.* 2022, 118094). Increasing the hydrothermal temperature from 130 to 150 °C promotes the transition from aggregated/layer-like products to well-defined fibrous and bowl-shaped architectures, whereas further increasing the temperature to 180 °C results in irregular nanoparticles. Low temperature produces aggregated nanospheres with poorly defined mesostructures, while elevated temperature leads to more developed mesoporous spherical architectures. We have added photographs of the reaction solution after hydrothermal reactions for 40 min at 130, 150, and 180 °C to support this. These results indicate that temperature regulates the formation of MCNs through coupled effects on micelle curvature, TOP swelling/interfacial affinity, and LWMF polymerization kinetics. Regarding the "polymerization kinetics at these temperatures", we clarify that we do not aim to establish a full quantitative kinetic model with rate constants at each temperature. Instead, we use "polymerization

kinetics" to describe the apparent rate of precursor polymerization and nucleation, as evidenced by time-dependent quenching, temperature-dependent morphology, and HCl-concentration-dependent experiments.

Revised text ① (Page 16, line 299): Optical photograph of MCNs-3 precursor solution after 2 h hydrothermal treatment shows more pronounced solid formation at 180 °C, suggesting accelerated polymerization at elevated temperatures (Supplementary Fig. 22).

Revised text ② (Page 17, line 304): Further increasing the temperature to 180 °C led to irregular nanoparticles, suggesting that mismatch between the micelle self-assembly process and the polymerization kinetics of the carbon source under excessively high hydrothermal temperature, leading to disruption of the ordered assembly pathway.

Revised text ③ (Page 17, line 309): Aggregated nanospheres with poorly defined mesostructures were obtained at relatively low hydrothermal temperatures.

Revised text ④ (Page 17, line 314): Notably, the evolution in pore structure suggests that hydrothermal temperature affects the packing geometry of composite micelles. At elevated temperatures, the hydration of PEO segments is reduced, while the effective hydrophobic volume of the PPO/TOP domain increases. This change favors lower-curvature micellar configurations, thereby influencing the resulting mesopore geometry. These temperature-dependent results indicate that hydrothermal temperature affects both the kinetic matching between LWMF polymerization and soft-template assembly. Insufficient temperature slows precursor condensation and weakens framework fixation, whereas excessive temperature accelerates polymerization or disrupts micelle organization.

Revised text ⑤ (Page 33, line 631): Additional control experiments further support the coupled mechanism. Variations in hydrothermal temperature, F127 dosage, HCl concentration, surfactant composition and oil-phase chemistry all altered the final morphology and mesostructure (Supplementary Fig. 23–40), indicating that these parameters regulate the assembly pathway through coupled changes in interfacial energy, micelle curvature, precursor-template interactions and polymerization kinetics. Therefore, the observed morphology evolution should be interpreted as a cooperative thermodynamic-kinetic process, in which effective interfacial-affinity-biased locus selection and micelle-curvature regulation guide the assembly pathway, while polymerization kinetics fixes or disrupts the evolving mesostructure depending on the kinetic matching condition

Added Supplementary Fig. 22:

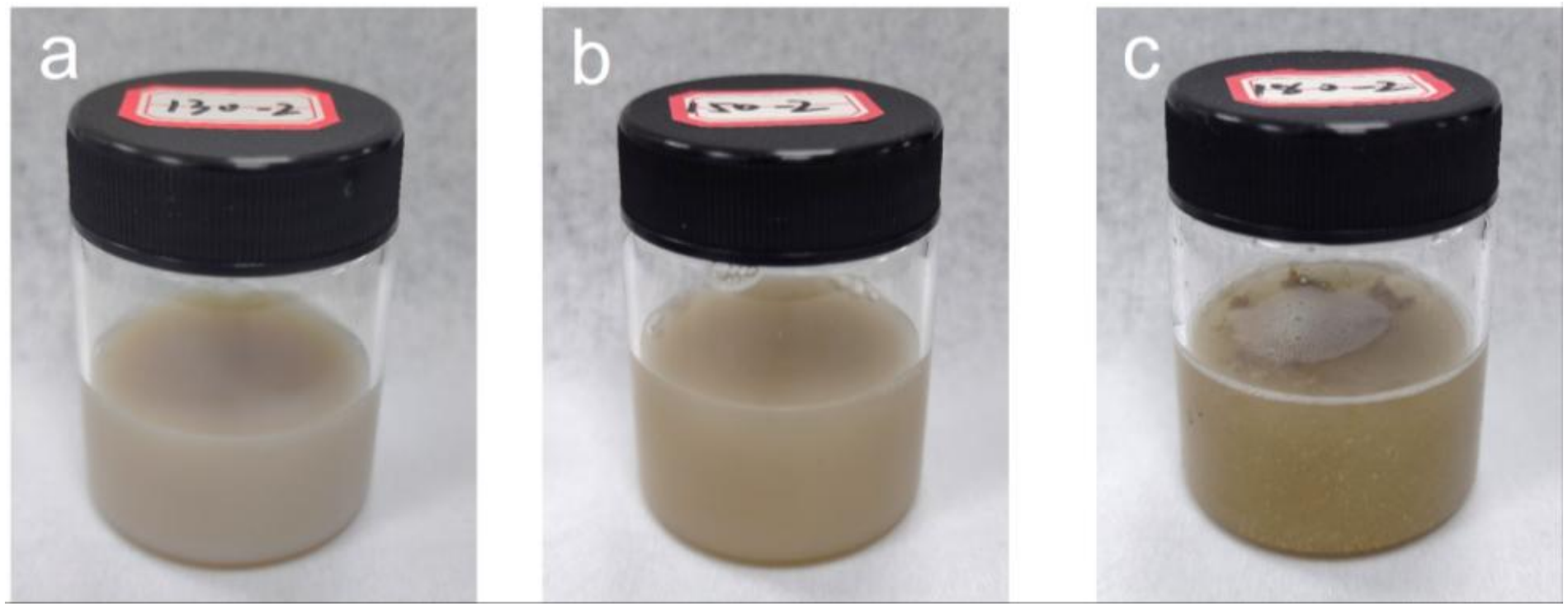

Supplementary Fig. 22. Photograph of the precursor solution after hydrothermal treatment for 40 min. (a) 130 °C, (b) 150 °C and (c) 180 °C.

We greatly appreciate your thoughtful and constructive comments, which have been very helpful in improving our manuscript. We have carefully considered all the suggestions and made corresponding revisions to strengthen the clarity and quality of our work.

Reviewer #3:

General comments: I co-reviewed this manuscript with one of the reviewers who provided the listed reports. This is part of the Nature Communications initiative to facilitate training in peer review and to provide appropriate recognition for Early Career Researchers who co-review manuscripts.

Response: We sincerely thank you for the time and effort you devoted to reviewing our manuscript. We greatly appreciate your careful reading, thoughtful consideration, and valuable comments, which help us improve the manuscript.

Reviewer #4:

General comments: ***This manuscript reports an interesting emulsion-mediated self-assembly approach for converting biomass into a diverse of mesoporous carbon nanoarchitectures. In particular, the tunable of nanofiber architecture from ordered multichannel structures to hollow nanofibers within a single synthetic platform is novel. Furthermore, the micelle structure and interfacial assembly behavior can be delicately adjusted by altering trioctyl phosphate and ethanol content. This is an original and well-designed study, supported by substantial experimental effort and extensive supplementary data. The results are convincing and should be of broad interest to the community working on biomass-derived carbon materials and porous nanoarchitectures. However,***

authors need to carefully consider the following comments before consideration for publication.

General response: Thanks for your positive comments about the originality, experimental design and potential impact of our work. We are also grateful for the constructive and insightful comments which help us substantially improve the clarity and rigor of the manuscript. We have examined the entire manuscript and Supplementary Information, corrected inconsistent sample descriptions and written errors, clarified the proposed self-assembly mechanism, weakened over-specific mechanistic analogies and added additional experiments and analyses. We have revised the discussion of the mechanism as an interfacial-energy/micelle-packing/kinetic-matching coupled framework rather than a fully separate effect of each variable and added more quantitative structure-property analysis for the thermoelectric performance and clarified the hierarchical structural terminology. Detailed responses to each comment are provided below.

Comment 1: ***It would be great if the authors could provide more information of the LW carbon precursor (chemical constitution or polydispersity).***

Response: We appreciate your interest in our work! In this synthesis system, LW serves as an important carbon source, of which chemical constitution and molecular-weight distribution are important for understanding its condensation behavior with formaldehyde/melamine and its interaction with composite micelles.

In our previous studies, the preparation, chemical composition and structural characteristics of LW were systematically investigated and reported in detail. The LW used here was prepared following the similar protocol as our previous studies; therefore, previous characterization provides representative compositional information. Briefly, the liquefaction process converts the wood components into a complex mixture of phenolic/aromatic fragments and polyhydroxy oligomers containing abundant hydroxyl groups, which can further react with formaldehyde/melamine to form LWMF resins and participate in hydrogen-bonding interactions with the PEO segments of F127 during the self-assembly. The data on the characterizations of LW in our previously published works are provided as follows:

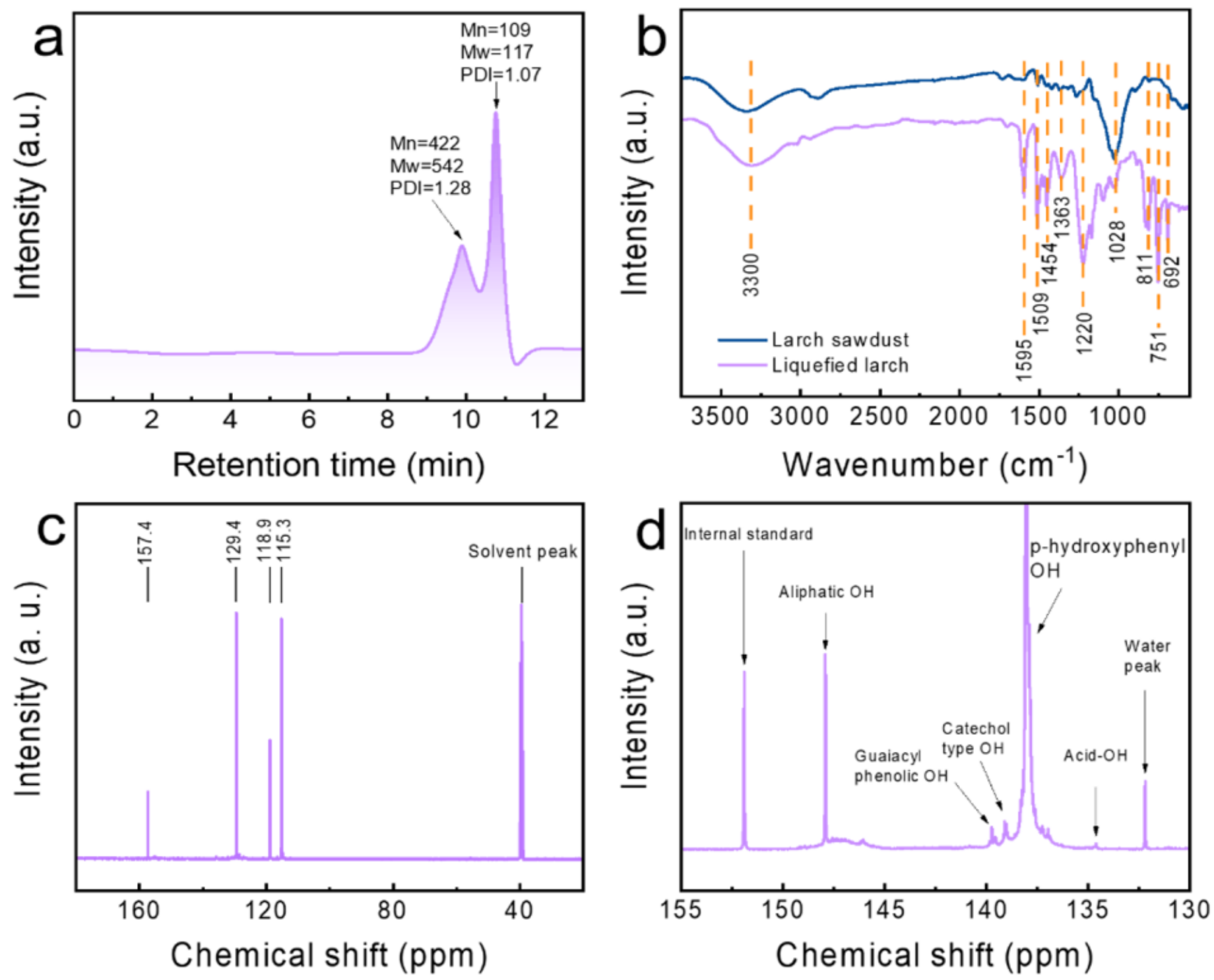

(a) GPC curve of LW. (b) FTIR spectra of larch sawdust and LW. (c) ^{13}C-NMR spectrum and (d) ^{31}P-NMR spectrum of LW.

The GPC curve of LW exhibits two narrow peaks, indicating the presence of two different compositions in LW. The first peak, with a low molecular weight of 542 g mol^{-1} is attributed to decomposed wood fragments generated during liquefaction, whereas the second peak is assigned to residual free phenol from the liquefaction process. FTIR analysis shows that, after liquefaction, the –OH stretching band at 3300 cm^{-1} becomes more pronounced, while the C–O stretching band of cellulose and lignin at 1028^{-1} decreases. The peak at 1220 cm^{-1} is assigned to Ar–O–C stretching vibrations. The bands at approximately 1595, 1509, 1456 cm^{-1} correspond to C=C stretching vibrations of the benzene ring, while those between 559 and 692 cm^{-1} arise from out-of-plane Ph–H bending vibrations. The 13C-NMR spectrum of LW displays four well-resolved signals, which are assigned to C–OH (157.4 ppm), meta-carbon (129.4 ppm), para-carbon (118.9 ppm) and ortho-carbon (115.3 ppm) in phenolic rings. The ^{31}P NMR spectrum further confirms the presence of various hydroxyl groups in LW, including aliphatic OH (3.76 mmol g^{-1}, 150.5–145.4 ppm), guaiacyl OH (0.67 mmol g^{-1}, 140.5–139.4 ppm), catechol-type OH (1.39 mmol g^{-1}, 139.4–138.5 ppm), p-hydroxyphenyl OH (22.48 mmol g^{-1}, 138.5–136.7 ppm) and carboxylic acid OH (0.30 mmol g^{-1}, 136.0–134.0 ppm).

The complete citation is [Z. Wu, K. Zhang, J. Sun, C. Ma, S. Luo, W. Li, S. Liu, Adv. Funct. Mater.

2023, 33, 2213852.], [Z. Wu, J. Sun, Y. Miao, L. E, Y. Liu, Z. Xu, K. Zhang, C. Ma, S. Luo, W. Li, S. Liu, Chem. Eng. Sci. 2022, 264, 118094.] and [Y. Li, Z. Wu, Y. Liu, K. Zhang, S. Luo, W. Li, S. Liu, Aggregate 2024, 5, e570.]

We have now added the corresponding description and references in the revised Supplementary Information to guide readers to the detailed characterization of LW.

Revised text in Supplementary Information: The LW used in this work is a biomass-derived oligomeric mixture rather than a single molecular compound. As reported in our previous studies, the liquefaction of larch sawdust in phenol under acid catalysis converts the rigid lignocellulosic structure into soluble phenolic-rich oligomers containing abundant hydroxyl and aromatic units. These chemical features allow LW to participate in subsequent melamine–formaldehyde co-polymerization and to interact with Pluronic F127 through hydrogen bonding and hydrophobic/aromatic interactions. Therefore, in the present reactive-emulsion-mediated assembly system, LW serves not only as a carbon precursor, but also as an active assembly precursor that contributes to precursor–template interaction, interfacial affinity and polymerization-induced structural fixation. The detailed molecular-weight distribution and spectroscopic characterization of LW, including GPC, FTIR, 13C NMR and 31P NMR analyses, have been reported in our previous studies [1–3].

Comment 2: ***Some key structure labels are not fully aligned between the main text and the Supplementary Information. For example, the 0.3 mL TOP sample is described as "bowl-like nanospheres" in the main text, while MCNs-4 is listed as "vesicle-like" in Table S1; similarly, the copolymer-template section assigns hollow spheres to P108 in the text, whereas the figure caption and Table S1 assign them to P84. It should be carefully checked throughout for typographical, grammatical, and formatting errors.***

Response: Thank you for carefully pointing out these inconsistencies. We agree that inconsistencies in structural labeling and terminology significantly affect the clarity of the manuscript. In the revised manuscript, we have performed comprehensive and systematic corrections. Beyond these specific corrections, we also thoroughly checked the naming and morphological description of all MCNs samples across the manuscript. This includes consistent use of sample names (MCNs-x), alignment with Table S1, and unified terminology for morphology and mesostructure. We have carefully revised figure captions, annotations, and formatting to ensure internal consistency throughout the manuscript.

The revised text is as follows:

Revised text (Page 19, line 362)**:** Upon partial replacement of F127 with P123, uniform vesicle-like nanospheres with well-defined internal cavities were generated (Fig. 5b,e). Further replacing Pluronic F127 with P84, well-defined hollow carbon spheres could be obtained (Fig. 5c,f).

Comment 3: ***How unambiguous is the separation between interfacial effects and kinetic effects in the proposed mechanism? The manuscript states that ethanol affects both interfacial tension and reaction kinetics, and HCl controls the kinetic matching window. Since TOP, ethanol, HCl, and surfactant composition all likely influence several coupled variables simultaneously, is the current mechanistic partitioning too clean relative to the complexity of the system?***

Response: Thanks for this insightful and important comment. We would like to clarify that the central aim of our mechanistic discussion is to highlight the dominant roles of interfacial interactions and micelle structural regulation in directing the programmable evolution of MCNs. These two aspects are emphasized because they most directly explain the transition from homogeneous aqueous self-assembly to interface-biased anisotropic growth, as well as the evolution of mesopore geometry from ordered channels to hollow or spherical architectures. At the same time, we fully agree that kinetic effects cannot be ignored. In the revised manuscript, **we emphasize that the structural evolution observed in this system originated from a coupled thermodynamic-kinetic interplay, rather than from independently acting variables.** More specifically: (i) Effective interfacial affinity ($\Delta\sigma_{eff}$) contributes to biasing the preferred assembly locus (bulk aqueous phase *vs.* oil-water interface), thereby influencing the relative contribution of predominantly homogeneous organization and interface-associated anisotropic organization. (ii) Micelle curvature/packing tendency provides a qualitative framework for rationalizing local mesophase geometry. (iii) Nucleation rate determines whether the self-assembly rate of micelles matches the polymerization rate of the carbon source, which is crucial for the formation of mesoporous structures.

Importantly, experimental variables such as TOP, ethanol, HCl, hydrothermal temperature and surfactant composition do not map one-to-one onto these effects: (i) TOP simultaneously affects the hydrophobic core swelling, interfacial energy and micelle stability. (ii) Ethanol influences solvent polarity, interfacial compatibility, headgroup area, and reaction kinetics. (iii) HCl regulates polymerization rate, but also indirectly affects micelle-precursor interactions. (iv) Hydrothermal

temperature affects the volume of the hydrophobic segment but also directly regulates the reaction kinetics. (v) F127 concentration modifies micelle population density and steric repulsion, thus affecting both packing and assembly dynamics.

Accordingly, in the revised manuscript, we have described the mechanism as a coupled framework in which interfacial interactions and micelle curvature/packing tendency are the primary structural-regulating factors, while kinetic matching acts as an additional regulating factor that influences structural fixation and ordering degree.

Revised text ① (Page 11, line 206): These results indicate that TOP acts not only as a hydrophobic swelling agent, but also as an interfacial modifier that changes the relative contribution of homogeneous aqueous assembly and interface-associated anisotropic organization. Low TOP content favors ordered mesochannel formation, intermediate TOP content promotes hollow fibrous architectures, and excessive TOP shifts the system toward low-curvature or lamellar-like assemblies that no longer preserve ordered fibrous frameworks.

Revised text ② (Page 29, line 570): Importantly, this interfacial-affinity balance contributes to the assembly-locus selection by biasing where micelle organization preferentially occurs, but does not independently determine the final morphology and mesostructure.

Revised text ③ (Page 34, line 631): Additional control experiments further support the coupled mechanism. Variations in hydrothermal temperature, F127 dosage, HCl concentration, surfactant composition and oil-phase chemistry all altered the final morphology and mesostructure (Supplementary Fig. 23–40), indicating that these parameters regulate the assembly pathway through coupled changes in interfacial energy, micelle curvature, precursor-template interactions and polymerization kinetics. Therefore, the observed morphology evolution should be interpreted as a cooperative thermodynamic-kinetic process, in which effective interfacial-affinity-biased locus selection and micelle-curvature regulation guide the assembly pathway, while polymerization kinetics fixes or disrupts the evolving mesostructure depending on the kinetic matching condition.

Comment 4: ***Although differences in specific surface area, pore volume, and pore size distribution among nanofibers, multicavity spheres, and gear-like spheres are listed, there is no systematic correlation with ion transport pathways, electronic percolation thresholds, or thermoelectric performance. The structure-property relationship remains incomplete.***

Response: We sincerely thank you for this constructive comment. In the revised manuscript, we have substantially expanded this part by correlating the structural characteristics of representative MCNs with their mixed ion-electron transport behavior and MTEG performance. A new structure-thermoelectric performance discussion has been added to the thermoelectric performances section. We also added a summary table comparing morphology, internal architecture, pore parameters, ionic/electronic conductivities, internal resistance, and thermopower of representative MCNs-based ionogels (MCNs-1/3/10/17) to clarify that the superior performance of hollow carbon nanofibers originates from the balanced coupling of one-dimensional electronic percolation and hollow/mesoporous ion-accessible pathways, rather than only from surface area or pore volume alone. Relevant literature on carbon-based mixed ion-electron thermoelectric systems has been cited to support the discussion.

The thermoelectric performance of MCNs-based ionogels is governed by the combined effects of several structure-dependent factors. First, a larger ion-accessible interfacial area can provide more contact sites for ion-carbon interfacial interactions, which enhance ionic polarization and thermovoltage generation. Second, shortened ion diffusion pathways, enabled by open mesopores and hollow interiors, facilitate faster ion redistribution under a temperature gradient. Third, efficient electronic percolation is essential for continuous power output under external loading. Previous studies on mixed ion-electron thermoelectric converters based on carbon nanotube/ionogel systems have shown that one-dimensional carbon networks can provide electronically conductive pathways, while ionogels provide ionic transport channels, enabling coupled ion-electron thermoelectric conversion. Fourth, balanced ion/electron charge compensation is critical because ionic thermodiffusion generates the thermovoltage, whereas electronic transport allows the polarized charges to be continuously compensated rather than merely stored capacitively. Finally, device-level internal resistance and its matching with the external load directly determine the extractable voltage and power density.

Based on this, we have further clarified the structure-dependent transport differences among the representative architectures, including nanofibers, golf ball-like nanospheres and multichambered nanospheres. For ordered mesoporous carbon nanofibers (MCNs-1), the one-dimensional high-aspect-ratio morphology is favorable for constructing continuous carbon pathways in the ionogel matrix. Such fibrous connectivity can lower the electronic percolation threshold and promote long-range electron

transport under external loading. Meanwhile, the ordered mesoporous channels aligned along the fiber axis provide directional ion diffusion pathways and accessible pore surfaces. Hollow carbon nanofibers retain the one-dimensional fibrous morphology but introduce an additional hollow interior and mesoporous shell. This architecture provides ion-accessible interfaces on both the outer and inner shell surfaces, shortens radial ion diffusion pathways, and facilitates ion accumulation/redistribution under a temperature gradient. More importantly, the hollow nanofibers still preserve fibrous electronic connectivity, allowing efficient electronic charge compensation during ionic thermodiffusion. Thus, hollow carbon nanofibers combine the electronic percolation advantage of one-dimensional nanofibers with the ion-accessible advantage of hollow/mesoporous architectures, leading to a more balanced ion/electron transport environment.

Nanosphere-based architectures mainly contribute to ion transport through their developed mesoporous surfaces, internal cavities, and rough external features. Golf ball-like nanospheres provide compartmentalized internal spaces, which can increase ion-accessible regions and promote local ion accumulation. Similarly, multichambered nanospheres possesses smaller particle size, and rough external morphology, may further enhance ion–carbon interfacial contact and electrolyte penetration. These structural features are beneficial for ionic thermodiffusion and interfacial polarization. However, unlike one-dimensional nanofibers, zero-dimensional nanospheres cannot readily provide an effective electron-transport network for persistent output, because charge transfer between particles relies only on discrete interparticle contacts and limited short-range tunneling junctions. This weak electronic connectivity is insufficient for sustained charge compensation under an external load, even though ionic thermovoltage can still be generated. Therefore, compared with hollow carbon nanofibers, nanosphere-based architectures are more prone to showing ionic thermoelectric behavior but less capable of delivering stable and continuous thermoelectric output.

Therefore, the comparison among these architectures indicates that different structural levels contribute differently to mixed ion-electron thermoelectric conversion. Zero-dimensional nanoparticles are advantageous for increasing ion-accessible interfaces and local ionic polarization, whereas one-dimensional nanofiber architectures are more effective in establishing continuous electronic pathways. Among the representative samples examined, hollow carbon nanofibers show the best performance because they combine both advantages: fibrous electronic connectivity and hollow/mesoporous ion-accessible transport pathways. This analysis explains why the structure–

property relationship cannot be evaluated solely by specific surface area, pore volume, or pore size distribution, but should instead be understood from the coupled perspective of ionic transport, electronic percolation, charge compensation, internal resistance and load-matched thermoelectric output. We have strengthened this part in the revised manuscript. We hope this can address your concerns.

Revised text (Page 33, line 640)**:** The mixed ion-electron thermoelectric behavior of ionogels is commonly governed by the balance between electronic percolation and ionic transport within the composite network[57]. As summarized in Supplementary Table 6, the superior performance of MCNs-3 cannot be attributed solely to the surface of pore volume, but rather to the coupling of one-dimensional electronic percolation, hollow/mesoporous ion-accessible interfaces, reduced ion-diffusion pathways and lower device-level internal resistance. One-dimensional ordered mesoporous carbon nanofibers provide continuous carbon backbones that facilitate long-range electronic connectivity, while their ordered mesoporous channels offer accessible ion-transport pathways along the fibrous framework. Hollow carbon nanofibers retain this one-dimensional electronic connectivity and introduce additional internal cavities and thin mesoporous shells, enabling ion access to both outer and inner carbon surfaces and shortening radial ion-diffusion pathways. This architecture is therefore more effective in coupling ionic polarization with electronic charge compensation under a temperature gradient[58,59]. By contrast, three-dimensional nanosphere-based architectures, including golf ball-like and multichambered nanospheres, provide abundant local ion-accessible surfaces, internal compartments, and rough external interfaces, which are beneficial for ion accumulation and interfacial polarization[60]. However, their zero-dimensional particle morphology makes long-range electron transport more dependent on discrete interparticle contacts. Such discontinuous electronic pathways are less favorable for forming a percolated carbon network in the ionogel matrix, thereby limiting electronic charge compensation and steady power delivery under external loading[61]. These explain why the hollow carbon nanofiber-based ionogels exhibit the most balanced mixed ion-electron transport among the representative architectures examined.

[57] Cho, C.; Kim, B.; Park, S.; Kim, E. High thermoelectric conversion through an optimal contribution of electronic carriers in polymeric mixed ionic–electronic conducting films. *Energy Environ. Sci.* **18**, 1294–1309 (2025).
[58] Schütt, F.; Signetti, S.; Krüger, H.; Röder, S.; Smazna, D.; Kaps, S.; Gorb, S.N.; Mishra, Y.-K.; Pugno, N.-M.; Adelung, R. Hierarchical self-entangled carbon nanotube tube networks. *Nat. Commun.*

8, 1215 (2017).
[59] Cao, L.; Sun, T.; Zhao, H.; Shang, M.; Wang, L.; Jiang, W. An actuatable ionogel thermoelectric fiber with aligned mesogens-induced thermopower for four-dimensional dynamically adaptive heat harvesting. *Nat. Commun.* **16**, 5445 (2025).
[60] Horike, S.; Wei, Q.; Akaike, K.; Kirihara, K.; Mukaida, M.; Koshiba, Y.; Ishida, K. Bicyclic-ring base doping induces n-type conduction in carbon nanotubes with outstanding thermal stability in air. Nat. Commun. 13, 3517 (2022).
[61] Cheng, H.; Yue, S.; Le, Q.; Qian, Q.; Ouyang, J. A mixed ion-electron conducting carbon nanotube ionogel to efficiently harvest heat from both a temperature gradient and temperature fluctuation. *J. Mater. Chem. A* **9**, 13588–13596 (2021).

Added Supplementary Table 6:

Supplementary Table 6. Summary of carbon nanoarchitecture and mixed ion-electron thermoelectric performance.

Samples	MCNs-1	MCNs-3	MCNs-10	MCNs-17
Key structural features	1D fibrous framework with ordered parallel mesochannels	1D hollow fibers with mesoporous shell and internal cavity	nanospheres with local cavities/rough surfaces	nanospheres with multichambered interiors
BET surface area/ (m^2 g^{-1})	1235	639	583	671
Pore volume/ (cm^3 g^{-1})	0.62	0.90	0.37	0.47
Ionic conductivity/ (S m^{-1})	0.248	0.361	1.44	1.10
Electronic Conductivity/ (S m^{-1})	7.00×10^{-7}	4.91×10^{-7}	6.02×10^{-15}	6.02×10^{-13}
Internal resistance/ (kΩ)	14.01	12.52	2.50	3.29
Thermopower/ (mV K^{-1})	25.6	26.8	23.8	24.4
Maximum P_{av}/ (W m^{-3})	63.01	68.32	10.67	11.38
Structure-property relationship	1D ordered framework enables efficient electronic percolation, while ordered mesochannels provide directional ion transport.	Hollow 1D architecture integrates continuous electronic pathways with enhanced ion-accessible interfaces, enabling balanced ion-electron transport.	High ion-accessible surface promotes ionic polarization, but discontinuous particle contacts limit electronic transport.	Reduced size and multichambered structure facilitate ion diffusion, yet insufficient electronic percolation restricts charge compensation.

Comment 5: ***In the thermoelectric measurements, only a limited number of samples are tested, leading to the simplified conclusion that hollow carbon fibers exhibit the best performance. A comprehensive investigation of thermoelectric behavior across the continuously tunable morphology library is lacking.***

Response: We truly appreciate you for this important comment, which is closely related to **Comment 4**. We agree that a full thermoelectric screening of the entire morphology library would be valuable, but it is beyond the scope of the present mechanistic materials-synthesis study. We have revised the manuscript to avoid overgeneralizing the conclusion and now present the thermoelectric measurements as representative proof-of-concept comparisons among selected architectures. Based on the existing research, we analyze the influence on thermoelectric performance of carbon materials from particle size and mesostructure, to elucidate the relationship between structural characteristics and corresponding properties. We used these as proof of concept to demonstrate the feasibility of the proposed synthesis strategy in the field of thermoelectric biomass-based porous carbon materials, rather than exhaustive performance optimization.

Dimensionality determines the nature of electronic transport within the ionogel matrix. One-dimensional nanofibers (MCNs-1 and MCNs-3) can form continuous conductive pathways, enabling efficient long-range electron transport with low percolation thresholds. In contrast, zero-dimensional nanospheres (MCNs-10 and MCNs-17) rely on discrete interparticle contacts, where electron transport is dominated by tunneling or hopping processes. This represents a transition from percolation-controlled transport to contact-limited transport, which is widely recognized in carbon-based mixed ion-electron conductors.

Within the 0D nanosphere systems, we further differentiate based on the size. Specifically, MCNs-10 exhibits a larger particle size (~170 nm), while MCNs-17 possesses a reduced size (~80 nm). From a transport perspective, the characteristic diffusion time scales with the square of the transport length (L^2), indicating that a reduction in particle size can accelerate ion redistribution kinetics. Therefore, the smaller particle size of MCNs-17 is expected to facilitate faster ion response under a temperature gradient. In addition, MCNs-17 features an interconnected multichambered structure, which introduces additional internal interfaces and may further influence ion distribution and interfacial polarization. We have strengthened this part in the revised manuscript.

Revised text ① (Page 23, line 444): Thermoelectric performance of MCNs-based ionogels is

governed by the coupled transport of ions and electrons, which is intrinsically influenced by the structural characteristics of the carbon frameworks. To demonstrate their functional potential, four representative architectures including ordered mesoporous carbon nanofibers (MCNs-1), hollow carbon nanofibers (MCNs-3), golf ball-like nanospheres (MCNs-10) and multichambered nanospheres (MCNs-17) were incorporated into ionogels to construct mixed ion–electron thermoelectric generators (MTEGs) (Figure 6c).

Revised text ② (Page 27, line 522): Moreover, the higher electronic conductivity of MCNs-17 ionogels ($\sigma_e = 6.02 \times 10^{-13}$ S m^{-1}) than MCNs-10 ionogels ($\sigma_e = 6.02 \times 10^{-15}$ S m^{-1}) indicates that reducing particle size and introducing multichambered structures can partially enhance electronic coupling between carbon domains. However, both systems remain predominantly ion-conducting, and the lack of a continuous electronic percolation network severely limits effective charge compensation under external loading. In this context, the reduced particle size of MCNs-17 may facilitate faster ion redistribution under a temperature gradient, yet this advantage is insufficient to overcome the intrinsic limitation imposed by discontinuous electronic pathways.

We respectfully note that this point is closely connected with Comment 4, as both comments concern the relationship between the structural diversity of MCNs and their thermoelectric behavior. Therefore, we hope you may consider our response to this comment together with our response to Comment 4. Together these revisions clarify both the rationale for selecting representative samples and the structural origin of the superior performance of hollow carbon nanofibers. We hope this can address your concerns.

Comment 6: ***The manuscript states that the interfacial self-assembly process is "consistent with the classical Frank–van der Merwe growth mode." This is a rather specific growth concept that is usually used in the context of epitaxial thin-film growth. In the present case, the system involves soft composite micelles, an emulsion interface, and polymerization-coupled assembly. Is this analogy sufficiently supported, or does it risk overstating the mechanistic precision of the proposed growth pathway?***

Response: Thanks for raising this important point. We agree that the original text was too specific for the present self-assembly system and could lead to an overly precise interpretation of the growth

pathway. We intended to describe the gradual adsorption and radial overgrowth of composite micelles at the TOP/water interface, rather than to claim a strict crystallographic or epitaxial growth model to this process. We have removed this terminology and replaced it with a more cautious description in the revised manuscript. We have described it as progressive interfacial adsorption and radial anisotropic overgrowth of composite micelles instead of directly assigning it to the classical Frank-van der Merwe model. The revised text is as follows:

Revised text (Page 6, line 117**:** Notably, directional interactions between composite micelles and TOP droplets promote progressive interfacial adsorption and radial anisotropic overgrowth.

Comment 7: ***At several sections, external morphology, hollow internal architecture, and mesopore geometry are discussed together, which makes the interpretation less clear. Are these three structural levels being used independently, or are they intended to describe a single coupled evolution process?***

Response: We appreciate you for the useful suggestion, which helped us improve the clarity of the structural discussion. These three structural levels are not to describe the same structural features, nor are they entirely independent descriptors. Specifically, external morphology refers to the overall particle shape, such as nanofibers, nanospheres, bowl-like particles, nanoribbons, and branched particles. Internal architecture describes the inner structural state, including solid, hollow, vesicle-like, and multichambered structures. Mesopore geometry refers to the local pore arrangement within the carbon framework, including cylindrical channels, strip-like channels, spherical mesopores, worm-like mesopores, and lamellar mesostructures. In our synthesis, changes in TOP content, ethanol content, F127 dosage, HCl concentration, and hydrothermal temperature simultaneously influence all three structural levels, although to different extents.

Therefore, the final MCNs architectures were described by combining these three hierarchical levels rather than by relying on only one. At the same time, we would like to clarify that different synthetic parameters do not necessarily affect these three structural levels to the same extent. For example, TOP content more strongly influences micelle swelling, interfacial assembly tendency, and the transition from ordered channels to hollow/bowl-like architectures, whereas ethanol content has a more pronounced effect on the transition from one-dimensional fibrous morphology to three-dimensional nanosphere-based morphology and on the evolution of mesopore geometry. Similarly, F127 dosage,

HCl concentration, and hydrothermal temperature may preferentially affect micelle packing density, polymerization kinetics, or structural fixation. For this reason, some sections of the manuscript emphasize one structural descriptor more than the others, depending on which level is most strongly affected by the parameter being discussed. However, this does not mean that the three descriptors are independent or interchangeable. Rather, external morphology, internal architecture, and mesopore geometry jointly describe the final MCNs structure from outside to inside and evolve in a coupled but not necessarily identical manner during emulsion-mediated self-assembly.

We have improved the morphology and structural descriptions of different samples in Table S1, and the revised text in the manuscript is as follows:

Revised text ① (Page 10, line 177): The detailed parameter matrix and morphology/structure map are summarized in Supplementary Table 1.

Revised text ② (Page 10, line 186): Correspondingly, the resulting mesoporous carbon nanomaterials (MCNs) showed a systematic TOP-dependent evolution: the external morphology evolved from one-dimensional nanofibers to bowl-like nanospheres, while the internal architecture changed from ordered mesochannels to hollow structures with a progressive decrease in ordered channel density.

Revised text ③ (Page 14, line 251): Under a TOP volume of 0.2 mL, increasing ethanol content drove a transition from one-dimensional fibers to nanospheres, accompanied by a change in mesopore geometry from cylindrical to spherical pores.

Revised text ④ (Page 17, line 302): …induced a pronounced structure transition from aggregated nanostructures or layer-like structures to uniform fibrous and bowl-shaped architectures.

Comment 8: The work emphasizes the thermopower advantage of the as-prepared materials, but does not provide a systematic comparison with similar ionic-electronic hybrid thermoelectric materials or other biomass-derived carbon-based thermoelectrics.

Response: Thank you for this valuable suggestion! We have added a new comparison Supplementary Table 2 to the revised Supplementary Information. We compared the MCNs-3/ionogel with representative mixed ion-electron conductor materials, conventional electronic thermoelectric materials, inorganic ionic materials, ionogels and biomass-derived ionic thermoelectric materials.

Compared with conventional electronic thermoelectric materials, MCNs-3/ionogel exhibits a much

higher thermopower. Compared with purely ionic materials and many ionogels, MCNs-3/ionogel is advantageous because it can sustain output under external loading, which is enabled by electronic charge compensation through the carbon network. Compared with other mixed ion-electron systems such as CNT/ionogel and rGO/ionogel, the hollow carbon nanofiber architecture provides both one-dimensional electronic connectivity and ion-accessible mesoporous/hollow interfaces, leading to a high steady-state thermopower under a relatively large external load.

We also note that biomass-derived materials have rarely been applied in thermoelectric, most of which are cellulose-based ionic hydrogels/ionogels, which can show high ionic thermopower but do not show mixed ion-electron transport with continuous load output. To our knowledge, few studies have been designed and synthesized tunable mesoporous carbon nanostructures from biomass for thermoelectric applications. This is also one of the novelties of our work. The added text in the revised text is as follows. We believe this comparison better highlights the novelty of our work.

Revised text (Page 27, line 514)**:** Under an external load of 40 kΩ, MCNs-3 MTEG delivered a steady thermopower of 12.33 mV K^{-1}, which is higher than those of many representative electronic, ionic and hybrid thermoelectric materials summarized in Supplementary Table 2.

The added Supplementary Table 2:

Supplementary Table 2. Comparison of the steady thermovoltage of the ionogels with the MCNs-3 loading of 0.3 wt% in this work with the thermopowers (*S*) of some representative mixed ion-electron Conductor materials, electronic materials, inorganic ionic materials, ionogels, at room temperature.

Materials	Steady-State Thermopower/ (mV/K)	Continuous power supply	External Load Resistance (kΩ)	Reference
**Mixed ion-electron Conductor materials**				
MCNs-3/ionogel	12.33	yes	40	This work
CNT/ionogel	0.05	yes	1	[11]
rGO/ionogel	7	yes	25	[12]
**Electronic TE materials**				
Bi_2Te_3	0.21	yes	-	[13]
PbTe	0.33	yes	-	[14]
Sb/Sb_2Te_3	0.54	yes	-	[15]
Iodine-doped Pentacene	0.07	yes	-	[16]
PEDOT:Tos/Au	0.021	yes	-	[17]
Lignin/PAN-derived carbon nanofibres	0.01	no	-	[18]

**Inorganic ionic materials**				
$RuIn_3$	0.4	no	-	[19]
$IrIn_3$	0.02	no	-	[19]
$LaCoO_3$	0.5	no	-	[20]
CdS	-0.14	no	-	[21]
Cu_2S	0.15	no	-	[21]
MnS	0.3	no	-	[21]
**Ionogels**				
PVA/IL ionic hydrogels	-	no	2	[22]
NaCl–PMSC Ionogel	-	no	-	[23]
PEO-IL Ionogels	-	no	200	[24]
Cellulose-based ionogel	-	no	0.01	[25]
Cellulose-based ionogel	6	yes	-	[26]

CNT: Carbon Nanotubes; rGO: Reduced Graphene Oxide; PEDOT:Tos: Poly(3,4-ethylenedioxythiophene):p-toluenesulfonate; PVA: Poly(vinyl alcohol); PMSC: Poly(methanesulfonic acid cellulose); PEO: Poly(ethylene oxide); PAM-P407: Polyacrylamide-Poloxamer 407.

Added references:

[11] Cheng, H.; Yue, S.; Le, Q.; Qian, Q.; Ouyang, J. A mixed ion-electron conducting carbon nanotube ionogel to efficiently harvest heat from both a temperature gradient and temperature fluctuation. *J. Mater. Chem. A* **9**, 13588–13596 (2022).

[12] Qian, Q.; Cheng, H.; Xie, H.; Wu, Y.; Fang, Y.; Le, Q.; Yue, S.; Ouyang, J. A Mixed ion/electron thermoelectric generator with ultrahigh steady thermopower by exploring both the hole tunneling and ion accumulations. *Adv. Energy Mater.* **15**, 2404522 (2025).

[13] Kimberly, T. Q.; Ciesielski, K. M.; Qi, X.; Toberer, E. S.; Kauzlarich, S. M. High thermoelectric performance in 2D Sb_2Te_3 and Bi_2Te_3 nanoplate composites enabled by energy carrier filtering and low thermal conductivity. *ACS Appl. Electron. Mater.* **6**, 2816–2825 (2023).

[14] Martin, J.; Wang, L.; Chen, L.; Nolas, G. S. Enhanced Seebeck coefficient through energy-barrier scattering in PbTe nanocomposites. *Phys. Rev. B* **79**, 115311 (2009).

[15] Zhang, Z.; Wu, Y.; Zhang, H.; Zeng, Z.; Hu, Z. Enhancement of Seebeck coefficient in Sb-rich Sb_2Te_3 thin film. *J. Mater. Sci.: Mater. Electron.* **26**, 1619–1624 (2015).

[16] Gogoc,, S.; Data, P. Organic thermoelectric materials as the waste heat remedy. *Molecules* **27**, 1016 (2022).

[17] Petsagkourakis, I.; Riera-Galindo, S.; Ruoko, T.-P.; Strakosas, X.; Pavlopoulou, E.; Liu, X.; Braun, S.; Kroom, R.; Kim, N.; Lienemann, S. Gueskine, V.; Hadziioannou, G.; Berggren, M.; Fahlman, M.; Fabiano, S.; Tybrandt, K.; Crispin, X. Improved performance of organic thermoelectric generators through interfacial energetics. *Adv. Sci*. **10**, 2206954 (2023).

[18] Dalton, N.; Lynch, R. P.; Collins, M. N.; Culebras, M. Thermoelectric properties of electrospun carbon nanofibres derived from lignin. *Int. J. Biol. Macromol.* **121**, 472–479 (2019).

[19] Haldolaarachchige, N.; Phelan, W. A.; Xiong, Y. M.; Jin, R.; Chan, J. Y.; Stadler, S.; Young, D. P. Thermoelectric properties of intermetallic semiconducting $RuIn_3$ and metallic $IrIn_3$. *J. Appl. Phys.* **113**, 083709 (2013).

[20] Kumar, A.; Kumari, K.; Jayachandran, B.; Sivaprahasam, D.; Thakur, A. D. Thermoelectric properties of (1−x)$LaCoO_3$·x$La_{0.7}Sr_{0.3}MnO_3$ composite. *J. Alloys Compd.* **749**, 1092–1097 (2018).

[21] Hasan, M. N.; Wahid, H.; Nayan, N.; Mohamed Ali, M. S. Inorganic thermoelectric materials: a review. *Int. J. Energy Res.* **44**, 6170–6222 (2020).

[22] Huang, Y.; Zhao, X.; Ke, J.-L.; Zha, X.-J.; Yang, J.; Yang, W. Engineering nanoscale solid networks of ionogel for enhanced thermoelectric power output and excellent mechanical properties. *Chem. Eng. J.* **456**, 141156 (2023).

[23] Yang, X.; Tian, Y.; Wu, B.; Jia, W.; Hou, C.; Zhang, Q.; Li, Y.; Wang, H. High-performance ionic thermoelectric supercapacitor for integrated energy conversion-storage. *Energy Environ. Mater.* **5**, 954–961 (2022).

[24] Zhao, W.; Sun, T.; Zheng, Y.; Zhang, Q.; Huang, A.; Wang, L.; Jiang, W. Tailoring intermolecular interactions towards high-performance thermoelectric ionogels at low humidity. *Adv. Sci.* **9**, 2201075 (2022).

[25] Chen, X.; Lin, Y.; Chen, B.; Duan, R.; Zhou, Z.; Lu, C. Enhancing the thermoelectric performance of sustainable cellulose-based ionogels through water content regulation. *Small* **21**, 2412336 (2025).

[26] Li, X.; Tong, Z.; Qiu, Y.; Zheng, Z.; Zeng, S.; Zhao, D.; Yu, H. Balancing mechanical-thermal-electrical properties in cellulose ionogels via crystallization-induced molecular assembly. *Nat. Commun.* **16**, 9970 (2025).

Comment 9: The sentence referring to "TEP with longer alkyl chains" is clearly inaccurate, since TEP has the shortest alkyl chain among the phosphate esters compared in this section. This should be corrected, and the surrounding discussion should be checked carefully for similar imprecision.

Response: We apologize for this inaccurate description. We fully agree that TEP (triethyl phosphate) has the shortest alkyl chain among the phosphate esters compared in this section, whereas TOP (trioctyl phosphate) has the longest alkyl chain. We have corrected the inaccurate phrase "TEP with longer alkyl chains" and revised the corresponding discussion in the manuscript and the caption/discussion related to Supplementary Fig. 39.

Revised text ① (Page 21, line 402): TOP was systematically replaced by trialkyl phosphate oils with different alkyl-chain lengths and hydrophobicity, including triethyl phosphate (TEP), tributyl phosphate (TBP) and triamyl phosphate (TAP), while keeping the remaining synthetic parameters unchanged.

Revised text ② (Page 22, line 417): The use of different trialkyl phosphates led to distinct mesostructural evolution…

Revised text ③ (Page 22, line 410): Notably, when TAP with longer alkyl chains was used…

Revised caption of Supplementary Fig. 39: TEM images of mesoporous carbon nanomaterials synthesized by replacing TOP with trialkyl phosphate oils of different alkyl-chain lengths and hydrophobicity. Oil phase: (a, d) TEP, (b, e) TBP and (c, f) TAP.

Comments 10: Reproducibility is a big issue in MCNs for its practical applications. How about the synthesis strategy's versatility? It is better to add more comments.

Response: Thank you for raising this important concern. We agree that reproducibility and versatility are critical for MCNs. Considering the biomass-derived systems where the chemical composition of the precursor may vary depending on the biomass source, we have performed additional experiments using other two different biomass resources, coconut shell and corn straw as carbon precursors. After liquefication, the obtained biomass-derived liquid precursors were used in the provided emulsion-mediated self-assembly strategy to prepare mesoporous carbon materials. The SEM and TEM images show that regular morphology and mesoporous carbon architecture can be obtained, indicating that the proposed strategy is not limited to wood as a biomass source, and it also shows promising versatility for converting various biomass into mesoporous carbon materials with controlled morphologies and architectures. In addition to testing different biomass sources, we repeated the representative hollow nanofiber synthesis under the optimized condition, and similar morphology/mesostructure was obtained, supporting the reproducibility of the synthetic protocol.

Moreover, we further investigated the TOP and ethanol effects in these biomass-derived synthesis systems by using the same synthesis method as MCNs-1/3/10/17. The results show that TOP and ethanol remain effective in regulating the morphology and mesostructure of the resulting carbon materials. We have added these new results and corresponding discussion in the revised manuscript and Supplementary Information to strengthen the reproducibility and generality of the proposed method. We sincerely hope that these additional experiments and revisions can address your concern and better demonstrate the potential of our synthesis strategy. The revised text is as follows:

Revised text (Page 23, line 429)**:** The generality of this emulsion-mediated synthetic approach was also examined, in which coconut shell and corn straw were selected as the raw biomass resources and converted into liquefied biomass precursors (Supplementary Fig. 40). Despite the different origins and compositions from wood, regular morphology and mesoporous structures were obtained. Moreover, varying TOP and ethanol in these biomass-derived systems produced structural evolution trends similar to those observed for liquefied wood, supporting the generality of their regulatory roles in emulsion-mediated self-assembly.

Added Supplementary Fig. 40

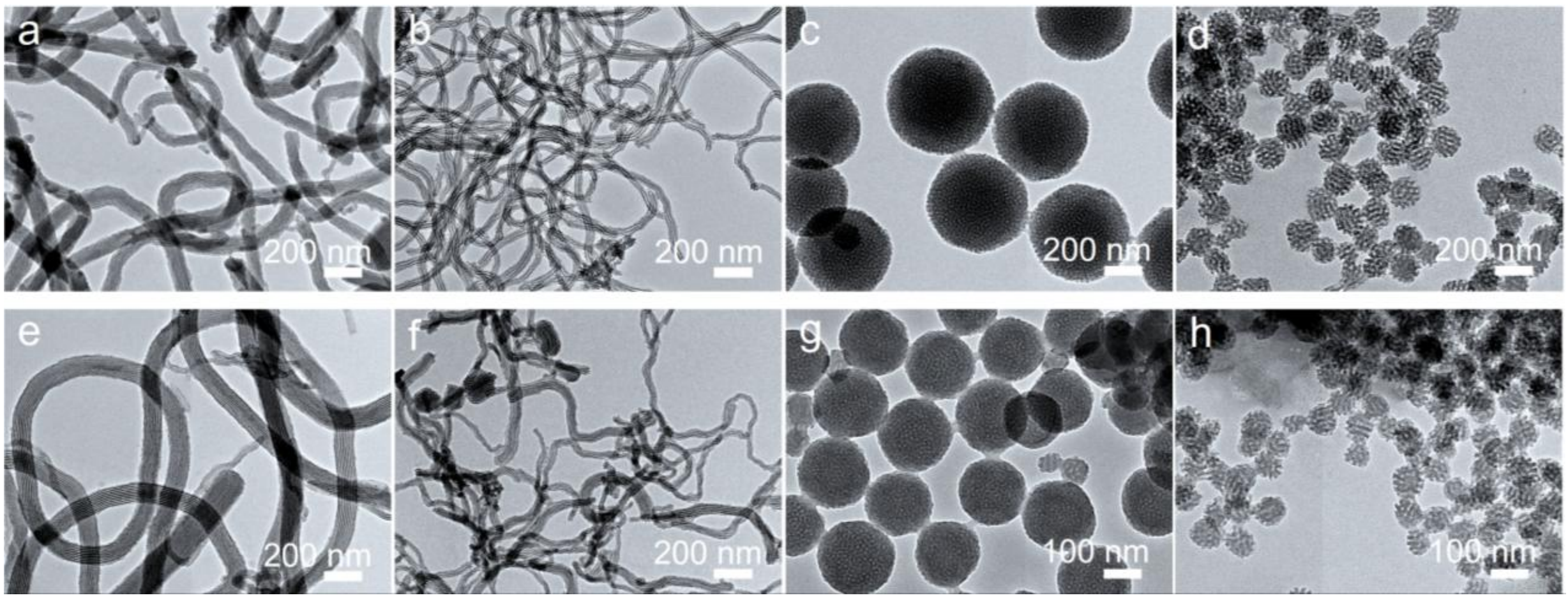

Supplementary Fig. 40. TEM images of MCNs synthesized from coconut shell (a–d) and corn straw (e–h). The synthesis condition was similar to the nanofibers and nanospheres. (a, e) 10 mL ethanol and 0.05 mL TOP. (b, f) 10 mL ethanol and 0.2 mL TOP. (c, g) 25 mL ethanol and 0.05 mL TOP. (d, h) 25 mL ethanol and 0.2 mL TOP.

Comment 11: XPS analysis indicates co-doping with N, P, and O; however, no control experiments (e.g., undoped or single-element-doped samples) are conducted to disentangle the individual contributions of heteroatoms to electronic conductivity, ionic thermal diffusion, and interfacial interactions.

Response: Thanks for this important and constructive suggestion. We agree that the possible contributions of N-, P- and O-containing species to electronic conductivity, ionic thermodiffusion, and interfacial ion-carbon interactions should be evaluated carefully. We agree that complete disentanglement of individual heteroatom effects is difficult because removing the dopant source also changes precursor chemistry and nanoarchitecture. We have performed six additional component-removal control experiments by omitting melamine, TOP or both under representative synthetic conditions of hollow nanofiber and nanosphere. SEM/TEM characterization, electronic conductivity measurements and thermovoltage curves were collected for these control samples.

The melamine-omitted samples are largely retained original morphology but show decreased electronic conductivity, suggesting that N species may partially contribute to improving the electronic conductivity, likely through local electronic structure and defect modulation. For ionic thermodiffusion and thermovoltage generation, the N/P/O-containing surface chemistry may also influence local ion-

carbon interactions, surface polarity, and interfacial ionic polarization under a temperature gradient. The thermovoltage curves of the component-removal samples show that the thermal voltage response changes after removing N- and/or P-related components. However, these changes are not solely determined by heteroatom composition, because morphology, pore accessibility, diffusion pathways, and electronic percolation are also altered to different extents.

Removing TOP strongly changes the particle size, morphology and pore structures of the obtained carbon materials. Therefore, the influence of the P-containing component on electronic conductivity and thermovoltage response is mainly associated with its role in regulating carbon nanoarchitecture, ion-accessible interfaces, and transport pathways, rather than being assigned simply to a direct P-doping effect. By contrast, the melamine-removal controls, where the morphology is better retained, suggest a relatively clearer contribution of N-containing species to electronic transport and possibly to local ion–carbon interfacial interactions. Accordingly, we have revised the manuscript to discuss the thermoelectric response as a combined effect of surface chemistry, ion-accessible hollow/mesoporous architecture and electronic percolation, rather than attributing it to individual heteroatoms alone.

Revised text ① (Page 25, line 484): Notably, the MCNs-3 ionogel exhibits the highest thermopower of 26.8 mV K^{-1}, suggesting that abundant ion-accessible interfaces and shortened ion-diffusion pathways provided by the hollow/mesoporous fibrous architecture facilitate ionic thermodiffusion and interfacial polarization.

Revised text ② (Page 26, line 497): Component-removal controls show that omitting melamine largely preserves the morphology but decreases electronic conductivity (Supplementary Fig. 46), suggesting a partial contribution of N-containing species to electronic transport. In contrast, TOP removal markedly alters particle size, pore architecture and carbon connectivity, indicating that the P-containing component mainly contributes through its structure-regulating role. The thermoelectric response is therefore attributed to the combined effects of surface chemistry, ion-accessible hollow/mesoporous architecture and electronic percolation.

Revised text ③ (Page 34, line 670): The fabricated hollow carbon nanofiber-based MTEGs deliver a high thermopower of 12.33 mV K^{-1} and a sustained average power density of 68.32 W m^{-3} under an external load of 40 kΩ, while maintaining stable operation over extended thermal cycling.

Added Supplementary Fig. 46:

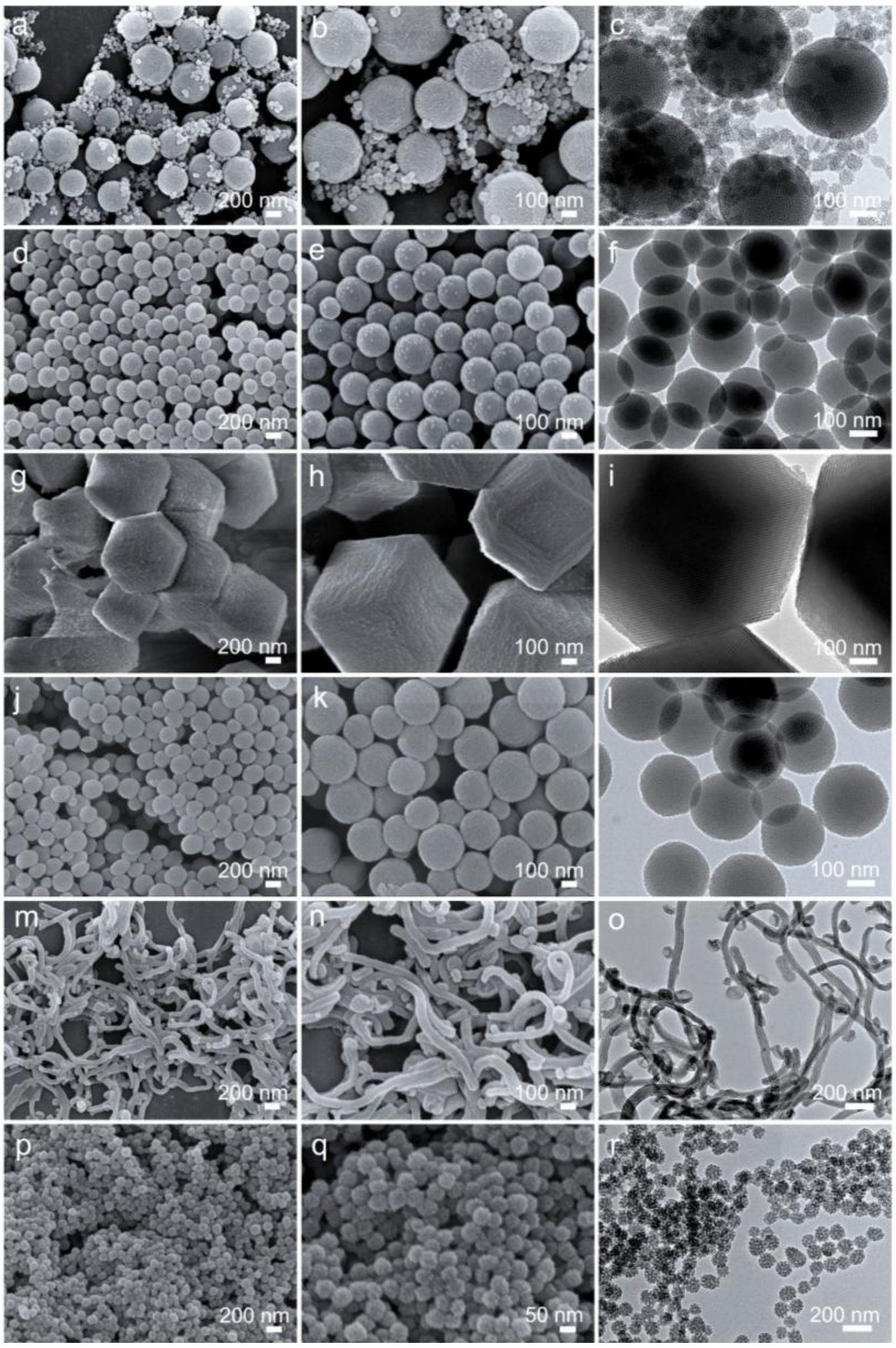

Supplementary Fig. 46. SEM and TEM images of MCNs prepared with similar process of hollow nanofibers (a–c, g–i and m–o) and multichambered nanospheres (d–f, j–l and p–r). The prepared process was performed similarly to Supplementary except (a–f) without adding melamine and TOP, (g–l) without adding TOP and (m–r) without adding melamine.

Added Supplementary Fig. 47:

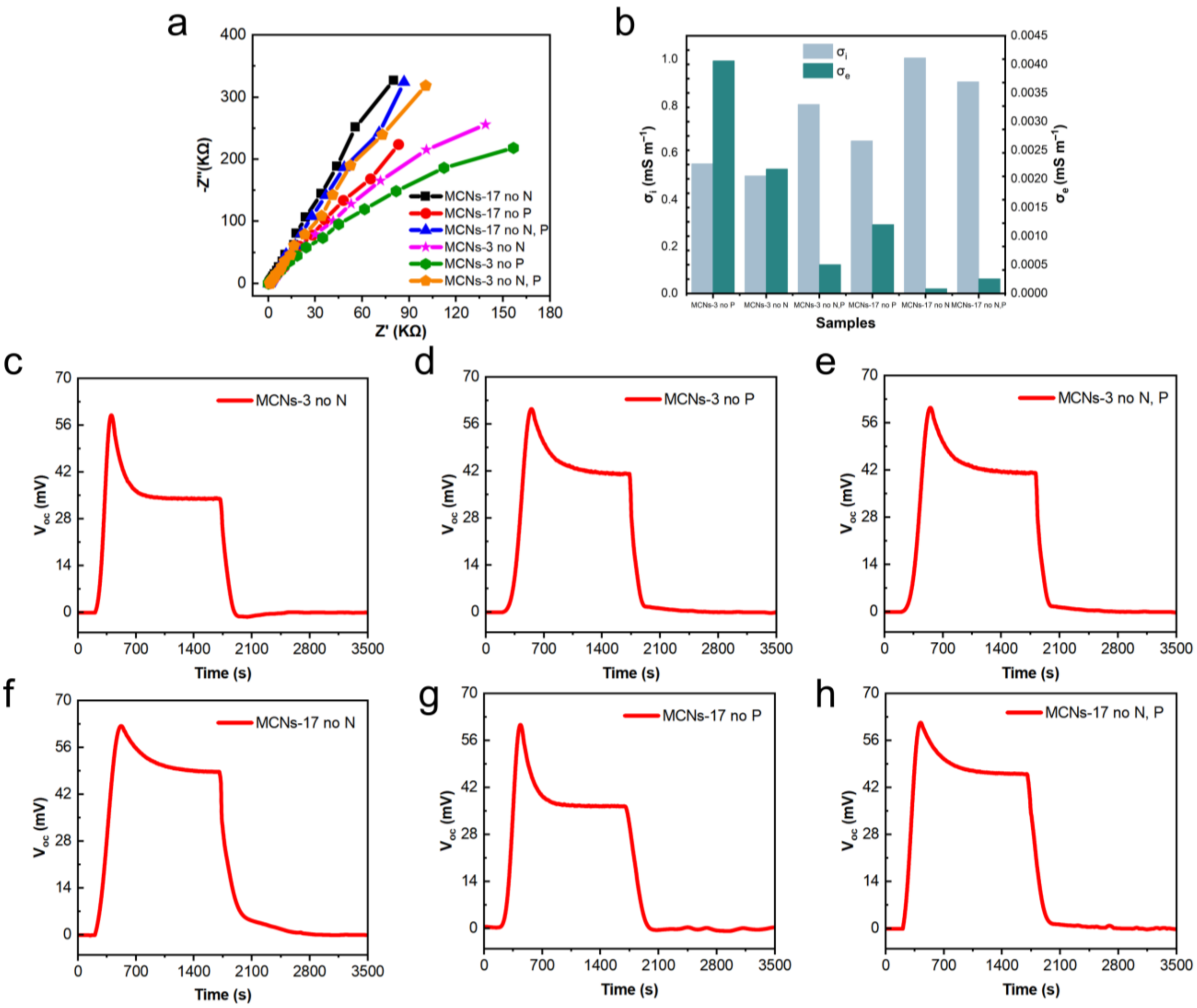

Supplementary Fig. 47. Electrochemical performance of the samples in N/P control experiments. (a) Nyquist plots. (b) Variations of the ionic and electronic conductivities of MCNs ionogels. (c–h) Voc response under the temperature of 3 K. The heating rate is 0.4 K min^{-1}, and the relative humidity (RH) is 70%.

Comment 12: The sample labeling is inconsistent. Some TEM/SEM images lack clear scale bars, and cross-sectional statistical analysis is insufficient. Key parameters such as fiber diameter, wall thickness, and particle size distribution are not quantitatively analyzed, weakening the claim of programmable morphology.

Response: Thank you for this careful and important comment. We agree that consistent sample labeling, clear scale bars and quantitative dimensional analysis are essential for supporting the claim of controllable morphology.

In the revised manuscript and supplementary information, we have systematically checked the sample labels, morphology descriptions, figure captions and Tables. We checked the SEM and TEM images and added scale bars where necessary to improve the readability and reliability of the structural characterization. Moreover, we have refined the discussion of the representative nanofiber samples MCNs-1, MCNs-2 and MCNs-3 in fiber diameter, pore size and wall/shell thickness estimated from the corresponding SEM/TEM images. The revised text is as follows:

Revised text ① (Page 8, line 141): Scanning electron microscopy (SEM) images reveal that the as-prepared mesostructured resin polymers exhibit a uniform fibrous morphology, with a representative cross-sectional diameter of 40 nm and striped grooves along the nanofiber surface, which are consistent with the radial growth of composite TOP/F127/LWMF micelles during interfacial self-assembly (Supplementary Fig. 3a,c). Transmission electron microscopy (TEM) images show that the polymeric nanofibers possess well-defined hollow mesochannels of ~12 nm (Supplementary Fig.3b,d). After carbonization in N_2 atmosphere, fibrous morphology is retained while shrinking to a representative outer diameter of 30 nm (Fig. 1b). The hollow interior and porous wall are preserved after carbonization, with a wall thickness of ~8 nm as observed from the magnified TEM image (Fig. 1c,d).

Revised text ② (Page 10, line 189): uniform carbon nanofibers with a diameter of ~80 nm were generated with multiple linear protrusions observed on their surface (Fig. 3b). TEM images further reveal that these nanofibers possess multiple ordered mesoporous channels (~3 nm) packed in a hexagonal arrangement and aligned parallel to the one-dimensional fiber axis, suggesting that homogeneous micelle organization remains dominant under limited TOP swelling (Fig. 3c).

Revised text ③ (Page 10, line 193): the fibrous morphology was retained, but showed a reduced diameter of ~50 nm, accompanied by similar mesopore size and substantial decrease in ordered mesochannels density.

Thanks again for your constructive and thoughtful comments, which have helped us improve the clarity and quality of our manuscript.
